# Prognostic signatures associated with high infiltration of Tregs in bone metastatic prostate cancer

**DOI:** 10.18632/aging.203234

**Published:** 2021-07-06

**Authors:** Fanjing Meng, Xu Han, Zhixue Min, Xuehui He, Sha Zhu

**Affiliations:** 1 Key Laboratory of Tumor Immunity, Department of Immunology, School of Basic Medical Sciences, Zhengzhou University, Zhengzhou, China; 2 Department of Pathology, The Third People’s Hospital of Zhengzhou, Zhengzhou, China

**Keywords:** mCRPC, DEGs, bone metastasis, immune infiltration, biomarkers

## Abstract

Metastatic cancer especially bone metastasis (BM) is the lethal end-stage of castration-resistant prostate cancer (CRPC). To understand the possible molecular mechanisms underlying the development of the distant metastasis is of potential clinical value. We sought to identify differentially expressed genes between patient-matched primary and bone metastatic CRPC tumors. Functional enrichment, protein-protein interaction networks, and survival analysis of DEGs were performed. DEGs with a prognostic value considered as candidate genes were evaluated, followed by genetic analysis of tumor infiltrating immune cells based on Wilcoxon test and immunofluorescence identification. Expression profiles analysis showed that 381 overlapping genes were screened as differentially expressed genes (DEGs), of which 16 DEGs were randomly selected to be validated and revealed that most of these genes showed a transcriptional profile similar to that seen in the datasets (Pearson’s r = 0.76). Six core genes were found to be involved in regulation of extracellular matrix receptor interaction and chemotactic activity, and four of them were significantly correlated with the survival of PCa patients with bone metastases. Immune infiltration analysis showed that the expressions levels of COL3A1, RAC1, FN1, and SDC2 in CD4+T cells were significantly higher than those in tumor cells, especially regulatory T cell infiltration was significantly increased in BM tumors. We analyzed gene expression signatures specifically associated with the development of bone metastases of CRPC patients. Characterization of genes associated with BM of mCRPC is critical for identification of predictive biomarkers and potential therapeutic targets.

## INTRODUCTION

Prostate cancer (PCa) is one of the most common malignant tumors in men worldwide and is ranked in the top five in terms of morbidity and mortality [1, 2]. Although progress has been made in screening and early detection of prostate cancer, a large proportion of men still develop advanced prostate cancer. Androgen deprivation therapy (ADT) is the standard treatment for advanced or metastatic prostate cancer, but it progresses to castration-resistant prostate cancer (CRPC) within 2-3 years after the initiation of ADT [3]. The prognosis of men with CRPC is worse than that those who are of hormone-sensitive, and the risk of metastasis is particularly higher [4, 5]. Patients with metastatic CRPC generally develop metastasis to the bones, which is the lethal end-stage; this poses a formidable therapeutic challenge. It is estimated that more than 80% of patients with mCRPC have skeletal metastases [6]. Although mCRPC treatment options have increased over the past decade, the mortality and 5-year survival rates have remained unacceptable.

Bone metastasis leads to bone metabolic disorders, which can lead to bone -related events, such as spinal cord compression, pathological fractures, and severe pain, which usually require bone tumor-related surgical intervention and external radiotherapy. However, study demonstrated that these therapeutic methods not only reduce the overall survival time and life quality of the patients but also increase the burden of treatment [7]. The development of future treatments as well as the selection of therapies in individual patients requires a deeper understanding of the biological signature of bone metastasis. Due to tissue availability, genome-wide studies of biomarkers related to cancer-specific deaths are mainly based on observations of primary tumors, except in the more lethal metastatic lesions.

In this study, we aimed to identify the putative targets for BM PCa by integrated regulatory network and survival analysis, which provide a valuable resource for further investigations carried out to better understand the mechanisms and potentially guide therapy decisions. On most occasions, the combination of screened biomarkers and clinical information has been proven to provide a diagnostic basis for the accurate assessment of suspicious bone metastases.

## MATERIALS AND METHODS

### Data acquisition and DEGs identification

Primary and bone metastatic CRPC tumor gene expression profile datasets, GSE32269 and GSE77930, were obtained from an international public repository, NCBI-GEO (http://www.ncbi.nlm.nih.gov/geo/). Raw data in different datasets were converted to expression measures and normalized using the data processed package downloaded from the open source software for bioinformatics Bioconductor in R [8]. The limma package [9] was used for the identification of genes with aberrant expression profiles in primary tumors compared with bone metastatic CRPC samples. DEGs were defined using threshold |logFC| >1 and FDR <0.05 as criteria for comparison. Finally, the overlapping genes between the DEGs were considered as significant genes associated with bone metastatic CRPC. Bone metastasis and primary prostate cancer samples used in the real-time quantitative PCR and immunohistochemical analysis of this study were collected from CRPC patients who had not received chemotherapy or radiotherapy (Table 1) after obtaining informed consent in accordance with the protocol approved by the Ethics Committee of Affiliated Cancer Hospital and the First Affiliated Hospital of Zhengzhou University.

**Table 1:** Demographics and characteristics of the study group (n=42).

**Variable**	**Number or characteristics**
Mean age at diagnosis	67.5
Median age at diagnosis	61
Standard deviation	7.26
Stage (AJCC8^th^)	
IIIB: T3-4, N0, M0	21
IVA: Any T, N1, M0,	12
IVB: Any T, N1, M1	9
Neoadjuvant chemotherapy	Not received

### Functional and pathway enrichment analysis

DAVID (Database for Annotation, Visualization and Integrated Discovery) [10] was used for gene ontology (GO) analysis. The DEGs in primary and bone metastatic CRPC tumors were screened for functional enrichment. GO analysis was used to evaluate the potential functions and degree of enrichment of the DEGs in biological processes (BP), cellular components (CC), and molecular functions (MF). Kyoto Gene and Genome Encyclopedia (KEGG) database with a P-value of <0.05 was used to systematically analyze the differences in gene function [11]. In addition, gene set enrichment analysis (GSEA) was used to determine the statistically significant and concordant differences between the two biological states of primary and bone metastatic CRPC tumors. The false discovery rate was adjusted to 0.05, and FDR <0.05 was considered the cut-off criterion.

### Integration of PPI network and identification of hub genes

As an online database, the Search Tool for the Retrieval of Interacting Genes (STRING) [12] (version 10.0, http://string-db.org) was use to retrieve gene interactions providing experimental and predictive PPI (protein-protein interaction) information. Cytoscape software (version 3.4.0, http://www.cytoscape.org) was utilized to construct PPI networks [13] to visualize the interaction of the DEGs. Subsequently, the CytoHubba plug-in [14] software was used to calculate the degree centrality of the nodes and hub proteins with higher degrees of centrality were identified [15]. Additionally, the KEGG pathway enrichment analysis of the nodes in the significant modules was performed using Multifaceted Analysis Tool for Human Transcriptome [16].

### Validation of hub genes

To verify the above analysis results, real-time quantitative PCR was used to detect the expression patterns of 6 related genes using theQPK-201 SYBR Green master mix (Toyobo, Osaka, Japan) and the ABI 7300 system from Applied Biosystems. The thermocycling protocol was set as an RT step at 50° C for 20 min, DNA polymerase activation step at 95° C for 2 min, and a total of 35 PCR cycles (95° C for 20 s, 60° C for 30 s). The primers used in this study were synthesized from Invitrogen (Beijing China). All reactions were performed in triplicate, and all samples were standardized with GAPDH. A comparative CT method was used to calculate the fold change of expression in each gene. Expression data are described by a log-ratio calculated by comparing ΔCq from the BM tumor cells with ΔCq from the primary controls. The 2^-ΔΔCq^ method was used for expression level analysis.

### Immunohistochemistry and immunofluorescence evaluation

Immunohistochemistry (IHC) was carried out with 3μm thick sections of formalin-fixed, paraffin-embedded, bone-metastasis and primary tumor tissues from CRPC patients who had undergone surgical resection before radiochemical therapy. After de-paraffinization and rehydration of tissue sections, antigen retrieval was performed by microwave sterilization in 10 mM citrate buffer (pH 6.0). Primary antibody (anti-COL3A1, anti-PTPRF, and anti-SDC2, Beijing Zhongshan Golden Bridge Biotechnology Co., Ltd., Beijing, China) was used at a 1:50 dilution. Anti-rabbit, peroxidase-conjugated secondary antibody was then applied at a 1:500 dilution. Diaminobenzidine (DAB) was used to visualize the labeling and hematoxylin was used as the section counterstain. For immunofluorescence staining, Cy3 or FITC (BioLegend, USA) was used as a secondary antibody (1.5 μg/mL) for one hour, and nuclei were counterstained by 4′-6-diamidino-2-phenylindole (DAPI; Sigma, USA) for 5 min. After mounting, the sections were observed under an Olympus BX51 microscope at 200× magnification.

### Nonparametric estimation

Kaplan-Meier curve and survival estimation are a frequently used method to display time-to-event outcomes, especially for patients with different lengths of follow-up. Hub genes may play an important role in the progression of bone metastasis of CRPC for their centrality in the co-expression networks. In this study, based on the datasets from cBioportal, a database that collects and processes the Cancer Genome Atlas (TCGA) datasets the screened hub genes were analyzed using Kaplan-Meier to identify their specific association with the overall survival (OS) of PCa patients (Supplementary Table 1). According to cBioportal survival analyzing confidence interval, the screened hub genes were analyzed to identify the correlation of their specific altered expression with overall survival of PCa patients. P < 0.05 was considered as to be statistically significant.

### Statistics

The mean ± SD was used to present standard descriptive statistics. P values for experimental data were generated using a two-tailed Student’s t-test with unequal variance. A *P*-value or FDR of less than 5% was considered significant.

### Data availability statement

Publicly available datasets were analyzed in this study. This data can be found here: https://www.ncbi.nlm.nih.gov/geo/.

### Ethics statement

Samples were collected from patients after obtaining informed consent in accordance with a protocol approved by the Ethics Committee of Affiliated Cancer Hospital and the First Affiliated Hospital of Zhengzhou University (Zhengzhou, China).

## RESULTS

### Identification of DEGs in PCa patients with BM

**Figure 1:**
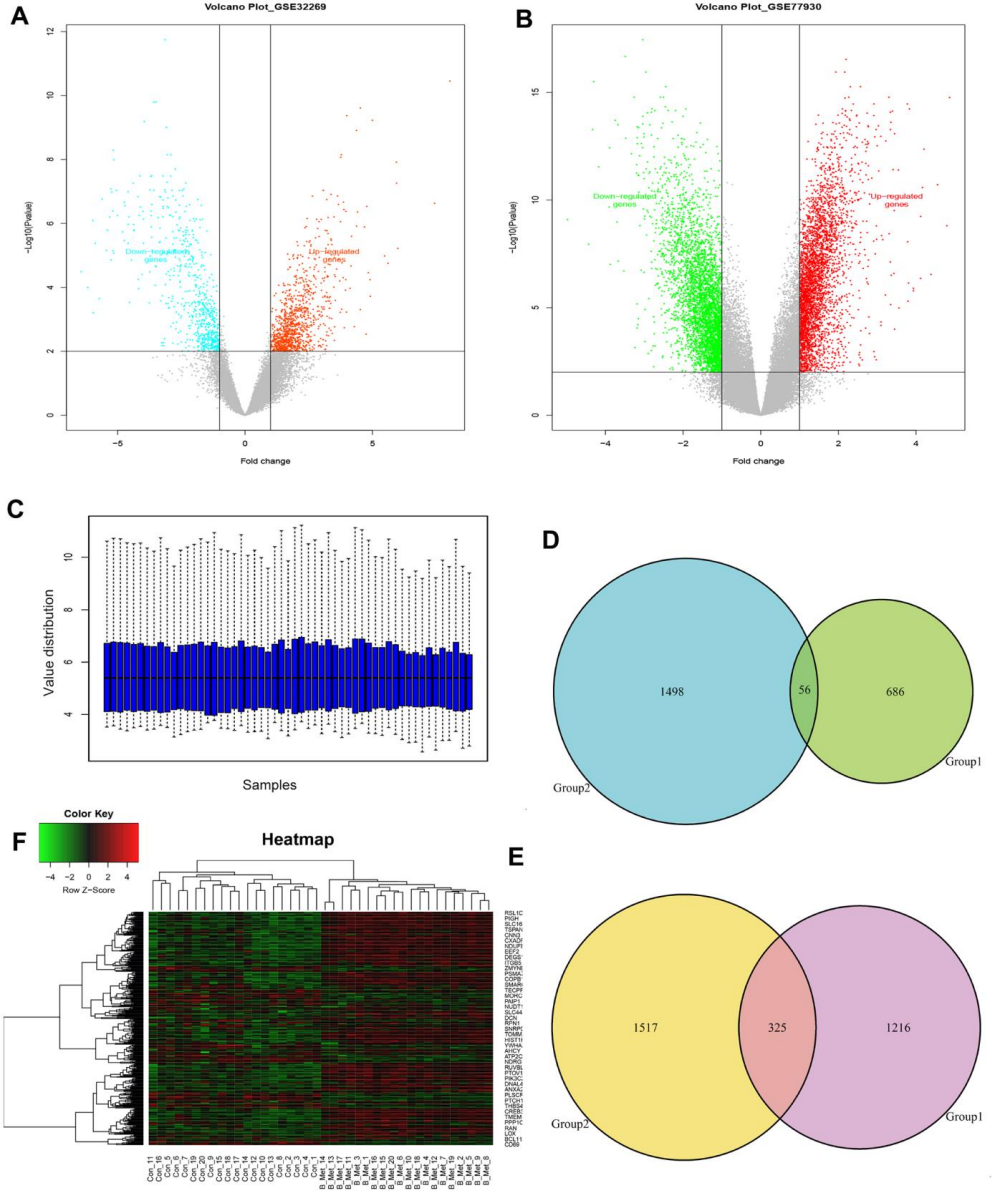
**Identification of the overlapping DEGs in GSE32269 and GSE77930.** Volcano plots of DEGs from analyzed microarray data of GSE32269 (**A**) and GSE77930 (**B**). (**C**) The gene expression distribution after normalization. Venn plots for the overlapping downregulated (**D**) and upregulated (**E**) DEGs. (**F**) The dendrogram of overlapping DEGs. Red represents higher expression and green represents lower expression. The criteria used to select DEGs were P <0.05 and |log2 (fold-change)| >1. DEGs, differentially expressed genes.

To identify the genes differentially expressed between bone metastasis and primary tumors, threshold |logFC| >1 and FDR <0.05 were used as cut-off criteria for comparative analysis. A total of 2283 DEGs were identified in GSE32269, while 3396 DEGs were identified in GSE77930 (Figure 1A, 1B and Supplementary Tables 2, 3). Among those, 742 (GSE32269) and 1554 (GSE77930) were downregulated, and 1541 (GSE32269) and 1842 (GSE77930) were upregulated. The gene expression distribution after normalization are shown in Figure 1C. We selected ten overlapping upregulated and downregulated genes, according to their biological function and log-ratio expression values (Table 2). A comparison of the DEGs between the two datasets revealed that 381 genes were overlapped, including 56 co-downregulated and 325 co-upregulated genes (Figure 1D, 1E). Thereafter, the overlapping DEGs were clustered to differentiate BM tumors from the primary samples and presented in the heatmaps (Figure 1F).

**Table 2:** Ten up- and down-regulated genes in bone metastasis tumors of PCa.

**Primary accession**	**Gene symbol**	**Log2 ratio**	**Main function**
NM_001040058	SPP1	4.793	Involved in the attachment of osteoclasts to the mineralized bone matrix. Probably important to cell-matrix interaction.
NM_001568	EIF3E	3.829	Component of the eukaryotic translation initiation factor 3 (eIF-3) complex, which is required for several steps in the initiation of protein synthesis.
NM_005971	FXYD3	3.786	This gene encodes a cell membrane protein that may regulate the function of ion-channels and may also play a role in tumor progression.
NM_00020	COL3A1	3.431	A fibrillar collagen that is found in extensible connective tissues such as skin, lung, uterus and the vascular system.
NM_000090	COL11A1	3.363	May play an important role in fibrillogenesis by controlling lateral growth of collagen II fibrils.
NM_207042	ENSA	3.342	The protein encoded by this gene belongs to a highly conserved cAMP-regulated phosphoprotein family.
NM_005561	LAMP1	2.972	A kind of membrane glycoprotein which provides selectins with carbohydrate ligands and may also play a role in tumor cell metastasis.
NM_002950	RPN1	2.912	This protein forms part of the regulatory subunit of the 26S proteasome and may mediate binding of ubiquitin-like domains to this proteasome.
NM_212482	FN1	2.776	This gene encodes fibronectin involved in cell adhesion and migration processes.
NM_001307	CLDN7	2.765	As a member of claudin family which are integral membrane proteins and components of tight junction strands.
NM_023111	FGFR1	-2.648	A member of the fibroblast growth factor receptor (FGFR) family where amino acid sequence is highly conserved.
NM_004456	EZH2	-2.663	Encodes a member of the Polycomb-group (PcG) family involved in maintaining the transcriptional repressive state.
NM_002409	MGAT3	-2.698	Glycosyltransferase involved in the synthesis of protein-bound and lipid-bound oligosaccharides.
NM_004762	CYTH1	-2.737	Mediate the regulation of protein sorting and membrane trafficking
NM_003248	THBS4	-2.868	Adhesive glycoproteins that mediate cell-to-cell and cell-to-matrix interactions.
NM_001024858	SPTB	-2.911	Actin crosslinking and molecular scaffold protein that links the plasma membrane to the actin cytoskeleton.
NM_001511	CXCL1	-3.153	Has chemotactic activity for neutrophils that signals through the G-protein coupled receptor.
NM_016612	SLC25A37	-3.155	Mitochondrial iron transporter that playing an essential role in heme biosynthesis.
NM_000298	PKLR	-3.785	Pyruvate kinase involved in a critical energy-producing process known as glycolysis.
NM_003245	TGM3	-3.814	Catalyzes the cross-linking of proteins and the conjugation of polyamines to proteins.

### Functional signaling pathway enrichment analysis

**Figure 2:**
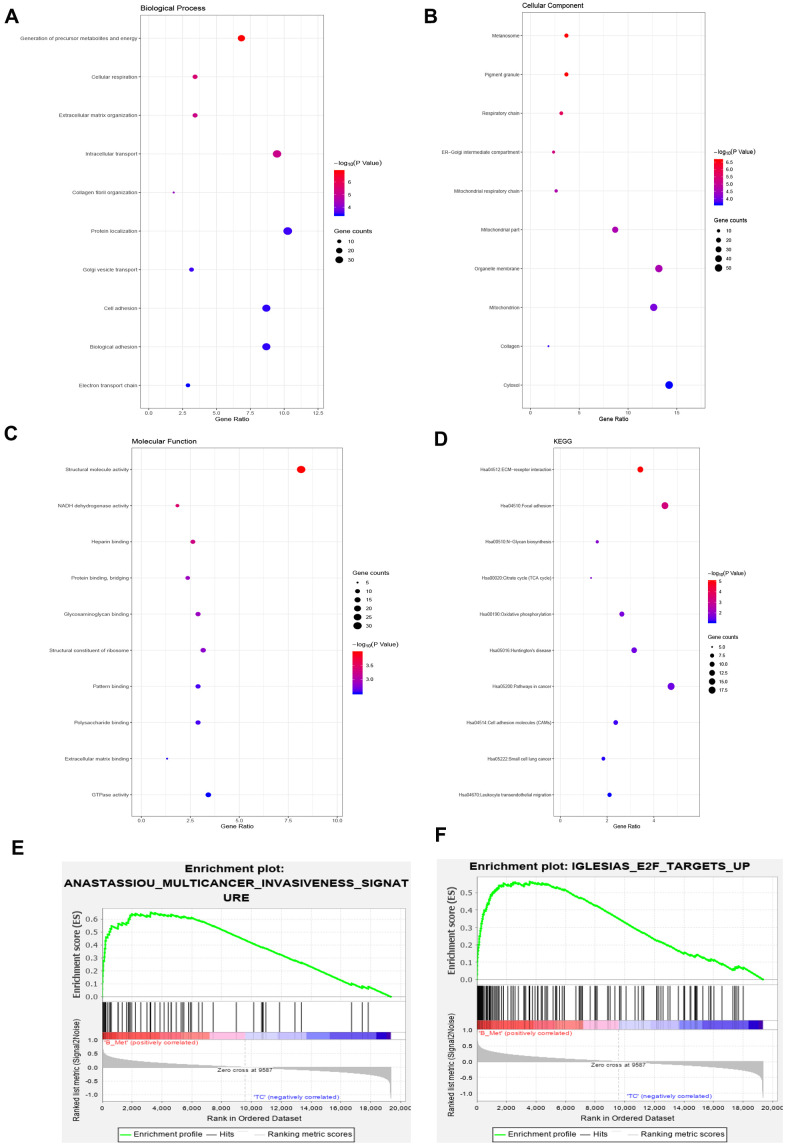
**Gene ontology, Kyoto Encyclopedia of Genes and Genomes and gene set enrichment analysis of the overlapping DEGs.** (**A**–**C**) Top 10 GO terms enriched in biological processes (**A**), cellular components (**B**), and molecular functions (**C**). (**D**) KEGG pathway analysis. (**E**) Enrichment of genes in ANASTASSIOU_MULTICANCER_INVA- SIVENESS_SIGNATURE. (**F**) Enrichment of genes in IGLESIAS_E2F_TARGETS_UP.

Functions and pathway enrichment analysis were performed, and the results demonstrated that the 381 overlapping genes are involved in several biological processes (BP), including generation of precursor metabolites and energy, cellular respiration, and extracellular matrix organization (Figure 2A). In terms of cellular components, DEGs were mostly enriched in the melanosome, pigment granule and respiratory chain (Figure 2B). Molecular function (MF) analysis revealed that the overlapping DEGs were mainly associated with structural molecule activity, NADH dehydrogenase activity and heparin binding (Figure 2C). Subsequential Kyoto Encyclopedia of Genes and Genomes (KEGG) pathway enrichment analysis showed that the common upregulated DEGs were primarily enriched in the extracellular matrix (ECM) -receptor interaction, focal adhesion, citrate cycle and N-glycan biosynthesis (Figure 2D). Moreover, Gene Set Enrichment Analysis (GSEA) was implemented between the BM and primary groups. Results revealed that gene sets were mainly enriched in the multicancer invasiveness signature and transcription factor E2F targets up (Figure 2E, 2F). These analyses reflect the biological processes associated with regulation of extracellular matrix receptor interaction and chemotactic activity. This significantly enriched gene ontology function GSEA analysis could help us further understand the roles of the overlapping DEGs, involved in the development of BM in CRPC.

### Modular analysis and candidate BM markers identification

**Figure 3:**
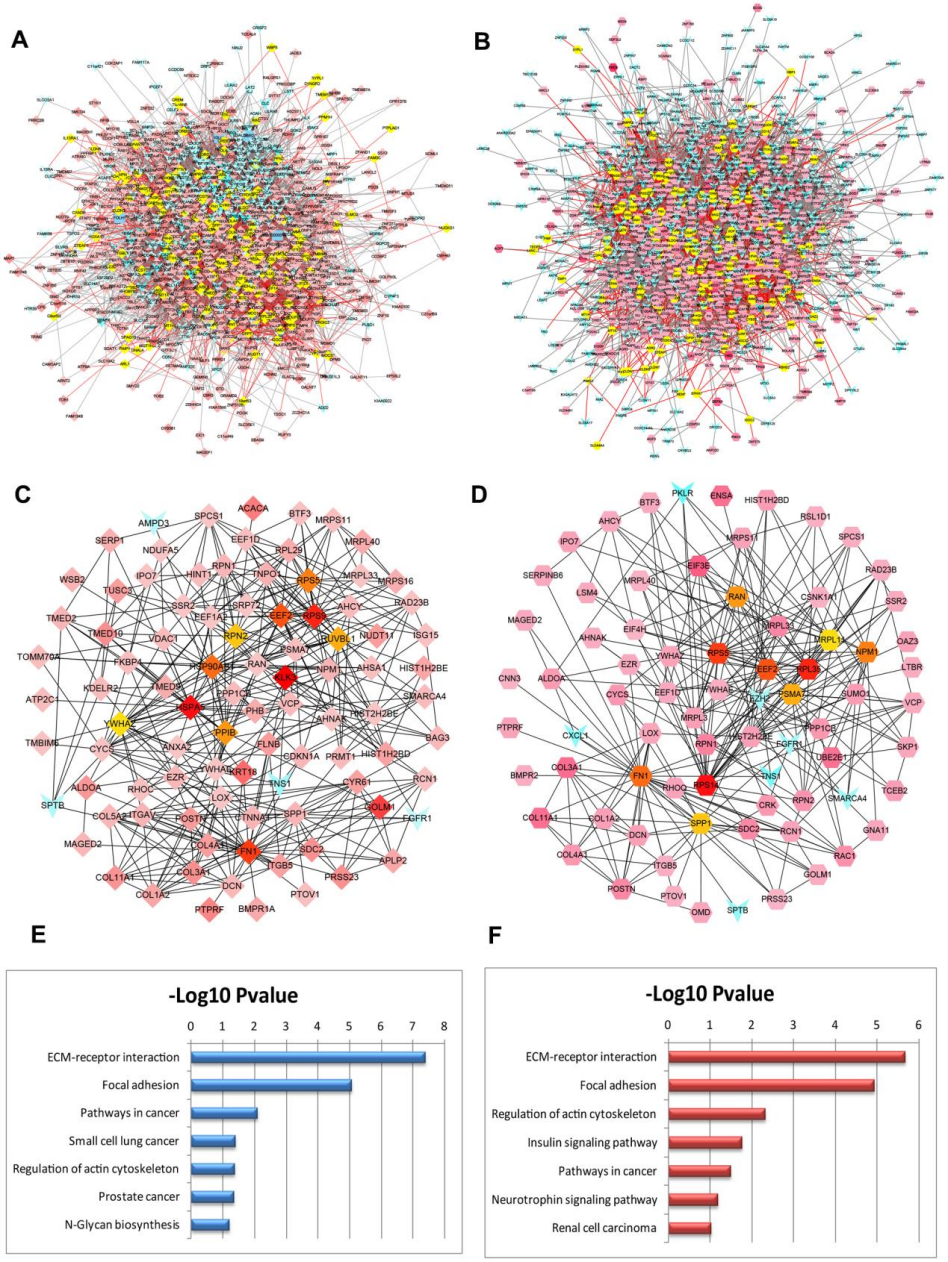
**PPI networks constructed by the DEGs from BM PCa and pathway function enrichment.** Deregulated gene networks of GSE32269 (**A**) and GSE77930 (**B**). (**C**) Network of significant hub proteins screened from GSE32269. (**D**) Network of significant hub proteins screened from GSE77930. Red and green intensities indicate the degree of upregulation and downregulation, respectively. (**E**) Pathway enrichment in network C. (**F**) Pathway enrichment in network D.

To further screen the hub genes of DEGs identified in the datasets, PPI networks were constructed using GSE32269 and GSE77930 significant proteins in which the overlapping nodes were highlighted in yellow (Figure 3A, 3B). To distinguish the hub genes based on the PPI networks, Edge Percolated Component (EPC) was chosen to identify candidate BM markers. The most significant modules composed of 10 nodes were screened out and highlighted with color from red to yellow according to their importance in the interactome network based on topological algorithms (Figure 3C, 3D). The EPC significant proteins consist of 93 nodes and 413 edges in the GSE32269 sub-network, 75 nodes and 273 edges in GSE77930 sub-network. By calculation of the value of the three features for each hub protein, the median values of “Closeness”, “Betweenness”, and “Degree” for GSE32269 and GSE77930 were 0.0043, 27.35, and 6.5 and 0.0051, 57.87, 3.5 respectively. Functional annotation and pathway analysis of the key nodes in the sub-networks are displayed in Figure 3E, 3F. This indicates that the ECM-receptor interaction pathway is significantly involved in the modules associated with the hub proteins.

### Validation and ROC analysis of the hub DEGs

**Figure 4:**
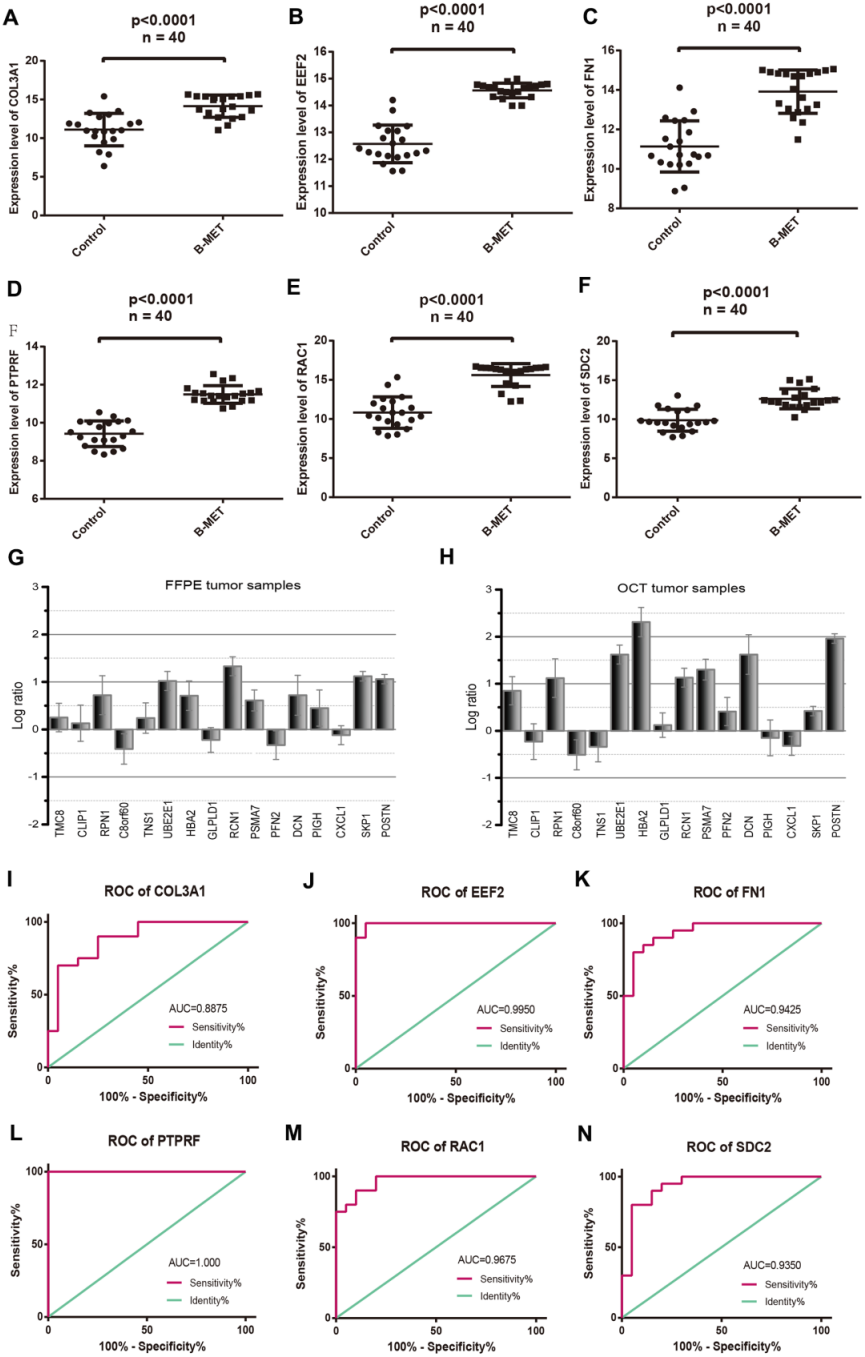
**Comparable evaluation of the expression patterns and ROC curves of six differentially expressed hub genes between PCa and controls.** Expression levels of COL3A1 (**A**), EEF2 (**B**), FN1 (**C**), PTPRF (**D**), RAC1 (**E**), and SDC2 (**F**). Detection of the expression values of the randomly selected DGEs in FFPE (**G**) and OCT (**H**) samples compared with the microarray data respectively. ROC curves of COL3A1 (**I**), EEF2 (**J**), FN1 (**K**), PTPRF (**L**), RAC1 (**M**), and SDC2 (**N**). (Means ± SEM FFPE n=5; OCT n=4).

The expression patterns of six DEGs including COL3A1, EEF2, FN1, PTPRF, SDC2, and RAC1, were evaluated by quantitative RT-PCR (Figure 4A–4F). The results showed upregulated and enhanced positive expression of these hub genes in BM tumors compared to the primary tumors of the OCT samples from CRPC patients. In addition, a panel of 16 DEGs with the lowest and highest expression range was also analyzed in BM tumors versus primary controls (OCT tumor samples, n=4; FFPE tumor samples, n=5) from patients with bone metastatic CRPC (Figure 4G, 4H). Results revealed that most of these genes displayed a transcriptional profile similar to that of the analyzed microarray data. The Pearson correlation coefficients between the microarray data and qRT-PCR of the 16 DEGs in OCT and FFPE samples were 0.83 and 0.78, respectively. Furthermore, receiver operating characteristic (ROC) analysis was performed for the hub genes (Figure 4I–4N), and the area under the curve (AUC) of COL3A1, EEF2, FN1, PTPRF SDC2, and RAC1 were 0.8875, 0.9950, 0.9425, 0.8675, 0.9250, and 0.9675, respectively (P< 0.01). The ROC curve represents the relationship between the true positive fraction and the false positive fraction resulting from a set of binary classification statistical tests of the expression data based on each possible decision threshold value [17].

### Evaluation of prognostic associated molecules

**Figure 5:**
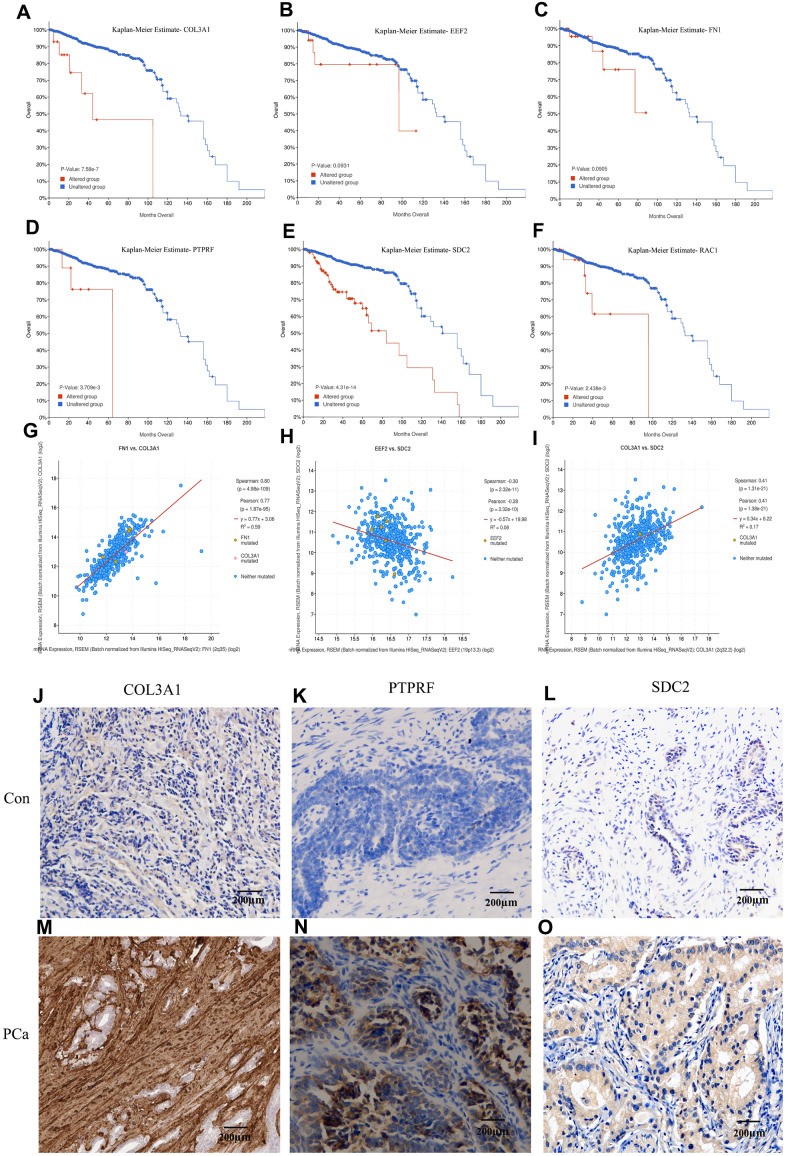
**Kaplan-Meier estimation of the overall survival and identification of the key molecules by immunohistochemistry in patients samples.** Overall survival analysis of COL3A1 (**A**), EEF2 (**B**), FN1 (**C**), PTPRF (**D**), SDC2 (**E**), and RAC1 (**F**). Expression linear correlations between FN1 vs. COL3A1 (**G**), EEF2 vs. SDC2 (**H**), and COL3A1 vs. SDC2 (**I**). (**J**–**O**) Detection of COL3A1, PTPRF, and SDC2 expression by immunohistochemistry in representative BM PCa and controls.

Kaplan–Meier survival analysis was performed to evaluate the prognostic values of the hub genes with respect to the overall survival (OS) of PCa patients. The results revealed that four hub proteins as independent risk factor, COL3A1, PTPRF, SDC2, and RAC1 were significantly associated with OS in the patients (Figure 5A–5F). The dataset from TCGA database were treated by cBioportal and the risk ratio and survival curve was drawn based on 6 key genes as independent risk factors. Result showed that COL3A1 and SDC2 were found to be the highest risk factors of prostate cancer, followed by RAC1 and PTPRF (Supplementary Figure 1). The expression signature of the ECM-receptor interaction associated molecules COL3A1, SDC2, and PTPRF were further identified by immunohistochemistry in clinical BM samples of CRPC patients. Results showed that the protein levels of COL3A1, SDC2, and PTPRF were particularly high in BM tumors than in the primary samples (Figure 5G–5I). Furthermore, we detected the expression levels of EEF2, FN1 and RAC1 in which we found that EEF2 is significantly high in tumors while the expression levels of FN1 and RAC1 were relatively low (Supplementary Figure 2). However, it is not clear whether these key genes are expressed in tumor cells or tumor microenvironment.

### Visualization and evaluation of tumor infiltration immune cells

**Figure 6:**
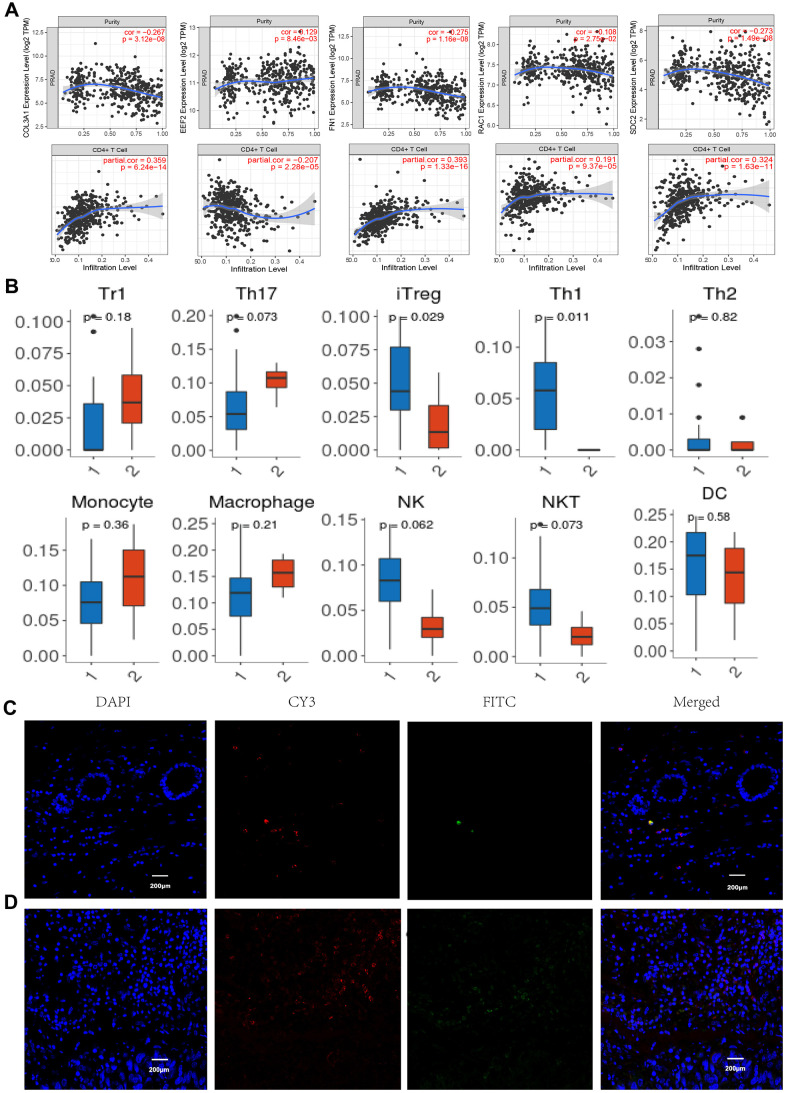
**Immune infiltration and immunofluorescence identification.** (**A**) Correlation of gene expression levels of COL3A1, EEF2, FN1, RAC1, and SDC2 with tumor purity and CD4+ T cells. (**B**) Tumor infiltrating lymphocytes profiles in the BM PCa (1. BM tumor, 2 normal tissues). Normal tissue (**C**) and BM tumor (**D**) immunofluorescence labeling for CD4 (red) and FOXP3 (green), as well as merged images. (n =6, P < 0.05, 200X).

Tumor Immune infiltration estimation showed that the expression levels of COL3A1, RAC1, FN1, and SDC2 were all negatively associated with tumor purity whereas positively correlated with CD4+ T cells (Figure 6A) in PCa, as indicated that these proteins may be expressed in this type of immune cells rather than in tumor cells. Cellular composition was evaluated by the Wilcoxon test based on standardized gene expression value, which showed the abundances of specific cell types. From the chart of Figure 6B, we identified a relatively high percentage of induced regulatory T cell (iTreg) and Th1 cell in BM PCa tissue (P< 0.05), which is consistence with the immune infiltration analysis. To further illustrate the immune cell profile, we detected the regulatory T cell by immunofluorescence staining of FOXP3 and observed that the Tregs are markedly increased in the BM tumor microenvironment (Figure 6C, 6D). Study indicated that increased infiltration of regulatory T cells in prostate cancer tissue is associated with a poor prognosis [18]. The aberrant immune cell infiltration especially the increased iTreg may have an important clinical value in prognostic and treatment of BM PCa.

## DISCUSSION

A bioinformatics method combined with high-throughput sequencing data was used to study the mechanism of prostate cancer, find the related genes and immune cell infiltration characteristics in BM prostate cancer, which provide the possibility of finding the target and related regulatory pathway in BM prostate cancer treatment. At present, the specific molecular mechanism of the metastasis of prostate cancer to the bone has not been fully elucidated. It involves the abnormal expression of many genes and the imbalance of related signaling pathways. In this study, we integrated two BM PCa datasets to comprehensively analyze and identify overlapping DEGs and immune cell infiltrating profile. Our research provides new insights into the molecular mechanism underlying the occurrence and development of mCRPC. The identification of genetic markers associated with PCa bone metastasis is essential for developing novel therapeutic targets.

Our gene expression analyses of the BM and primary PCa groups have shown that COL3A1, EEF2, FN1, PTPRF SDC2, and RAC1 act as hubs in the PPI networks, indicating their potentially significant roles. Functional enrichment results indicated that these overlapping genes were mainly involved in extracellular matrix organization, protein localization, cell adhesion, and ECM-receptor interaction. Type III collagen (COL3A1) is a structural protein that is classified as one of the major fibrillar collagens [19], which provides integrity for many organs. Studies indicated that COL3A1 is highly correlated with tumor progression and metastasis [20, 21]. COL3A1 has been identified as a representative biomarker that is overregulated in the focal adhesion pathway, and plays an unfavorable role in the development and metastasis of ovarian and bladder cancer [22, 23]. A previous study demonstrated that the mRNA and protein levels of COL3A1 are not only present in colorectal cancer (CRC) cells, but also increased in the plasma of CRC patients, which is associated with clinicopathologic factors and poor survival [24]. A report indicated that COL3A1 gene has prognostic implications in breast cancer [25]. Our results revealed that COL3A1, SDC2 and FN1 are dramatically enriched in the ECM-receptor interaction pathway, which may play a key role in the process of tumor development and bone metastasis of prostate cancer.

As a member of the syndecan proteoglycan family and an integral transmembrane protein, syndecan-2 (SDC2) plays an important role in controlling cell survival, proliferation, differentiation, cell-matrix interaction, and migration via its receptor of ECM proteins [26, 27]. The ECM contains collagen, proteoglycan, and several other glycoproteins to provide the structure of supporting cells where surface receptors can transmit signals from the ECM to the cells. These signals are essential for maintaining normal homeostasis [28]. In epithelial cells, SDC2 acts as a cross-link between ECM and actin cytoskeleton through its interaction with the PDZ domain of the CASK protein [29]. Ectopic or over-expression of this proteoglycan is related to the carcinogenic properties and poor prognosis of various malignant tumors [30, 31]. Deregulation of SDC2 might alter the expression of E-cadherin and consequently destroy cell-cell interactions, thereby increasing cell motility via the PKCa-dependent signaling pathway [32]. In addition, the expression levels of fibronectin 1 (FN1) were also found upregulated in BM tumors. As a member of the FN family, FN1 is widely expressed in many cell types and participates in ECM changes in physiological and pathological processes through integrin transmembrane receptors [33, 34]. Studies have shown that the expression of FN1 protein is closely related to the occurrence and development of many types of malignant tumors, such as ovarian cancer, renal cell carcinoma, and thyroid cancer [35–37]. Further, with the identification and recognition of ECM macromolecules in the remodeling of the tumor microenvironment, understanding the role of the ECM-receptor interaction in regulating tumor metastasis is becoming increasingly important.

From the network, we found that the expression levels of EEF2 and PTPRF were markedly increased in BM tumors compared to the primary samples of CRPC patients. EEF2 plays an important role in peptidyl-tRNA translocation and polypeptide chain elongation during protein synthesis. However, deregulation of this protein has been shown to be associated with tumorigenicity and cancer cell progression in mouse xenotransplantation model [38]. Overexpression of EEF2 correlates with cancer cell growth, metastasis invasion, and poor prognosis in acute myeloid leukemia, breast, and lung cancer [39–41]. Report indicated that significantly higher incidence of tumor recurrence and worse prognosis were found in elevated EEF2 patients, whereas silencing of eEF2 expression increased mitochondrial elongation and cellular autophagy [42]. It is suggested that EEF2 may be a promising target molecule for targeted cancer therapy [43]. Protein tyrosine phosphatase receptor type F (PTPFR) plays an essential role in the disassembly and reassembly of cell-cell and cell-extracellular matrix adhesions during epithelial cell migration. It has been found that high expression of PTPRF is related to the recurrence and metastasis of breast cancer and urothelial carcinoma of the bladder, while knockdown of PTPRF decreases cancer cell invasion and migration [44]. In addition, it was observed in the present study that the upregulated expression of RAC1 is inversely correlated with the survival rate of CRPC patients. RAC1, as a GTPase, belongs to the Ras superfamily of small GTP-binding proteins [45]. Hyperactivation of RAC1 has been implicated in numerous aspects of solid tumor development and could be the consequence of abnormal upstream inputs from tyrosine-kinase receptors and/or anomalous intracellular localization [46]. RAC1 GTPase was also found to signal Wnt-beta-catenin pathway-mediated tumor cell phenotypes which are involved in proliferation, tumorigenesis, and metastatic events [47].

Immune infiltration and immunofluorescence results showed that induced regulatory T cell were significantly increased in the BM prostate tumors. A high proportion of bone marrow T cells with regulatory phenotype (CD4^+^CD25hiFoxP3^+^) were also found in bone metastatic Ewing sarcoma patients [48]. Study revealed the active Treg cells recruitment and expansion in bone marrow of prostate cancer patients with bone metastasis, as may tilt the balance between osteoclast and osteoblastic activity and contribute to osteoblastic bone lesions of the patients [49]. Investigation indicated that TGFβ promotes Treg cell conversion while IL-2 induces Treg cell expansion from naïve T cells [50, 51]. In tumor immunity, Treg cells participate in the occurrence and development of tumors by inhibiting anti-tumor immunity [52]. High infiltration of Treg cells in TME has been associated with poor survival in various types of cancer [53, 54].

In conclusion, six core genes including COL3A1, EEF2, FN1, PTPRF SDC2, and RAC1, and several cancer-related metabolic processes, signaling pathways, and overall survival rate of patients were identified in BM PCa. These can be used as potential markers for early diagnosis and treatment. These analyses help in understanding the overall gene expression characteristics of bone metastatic CRPC and provide ideas and a theoretical basis for the diagnosis and treatment by which to increase the effectiveness of the current therapies.

## Supplementary Material

Supplementary Figures

Supplementary Table 1

Supplementary Table 2

Supplementary Table 3
