# Supplementary figures and images for: Prognostic signatures associated with high infiltration of Tregs in bone metastatic prostate cancer
Source: Aging (Albany NY). 2021 Jul 6;13(13):17442–61. doi: 10.18632/aging.203234 (PMC8312432; doi:10.18632/aging.203234)

SUPPLEMENTARY FIGURES

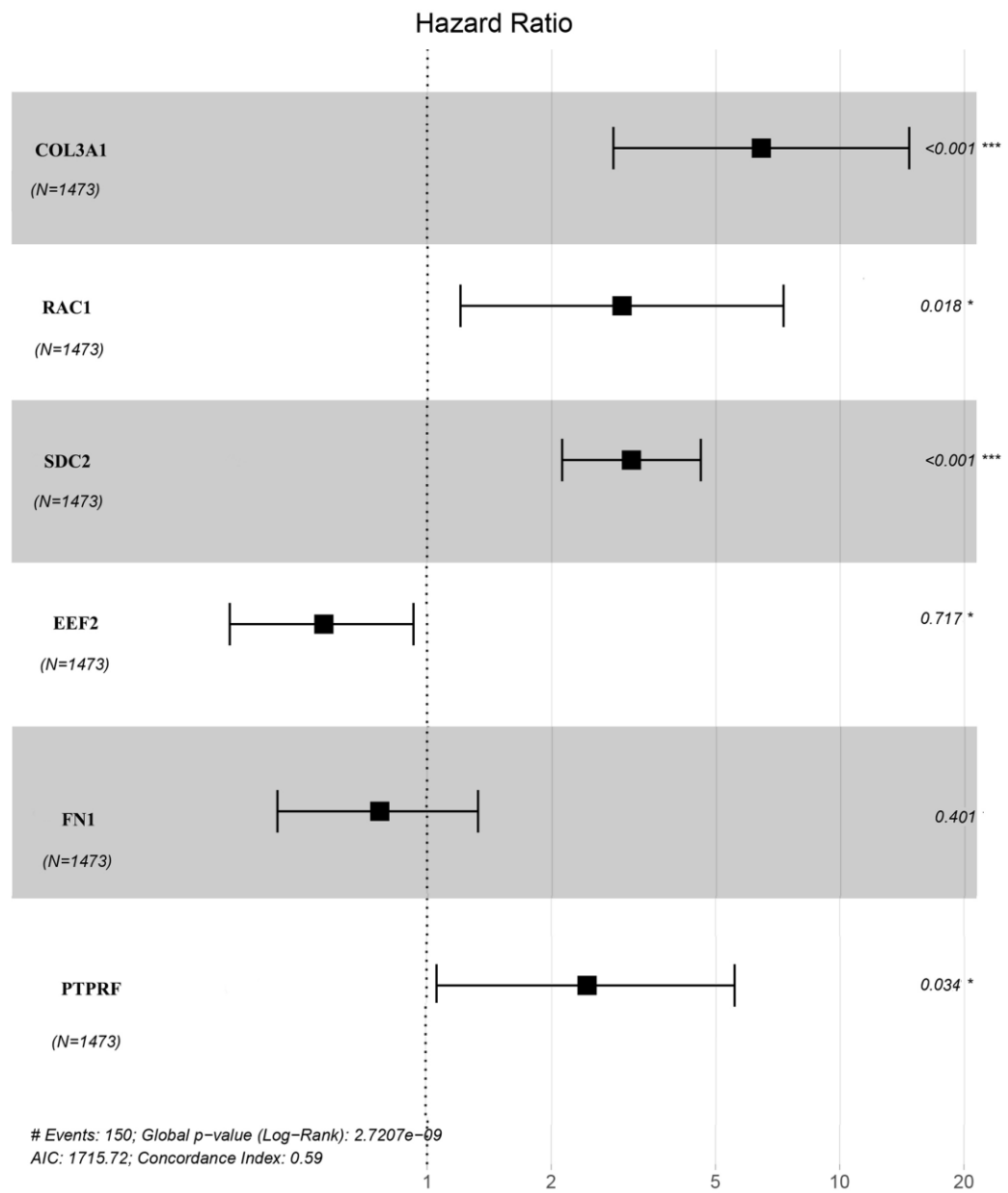

Supplementary Figure 1.

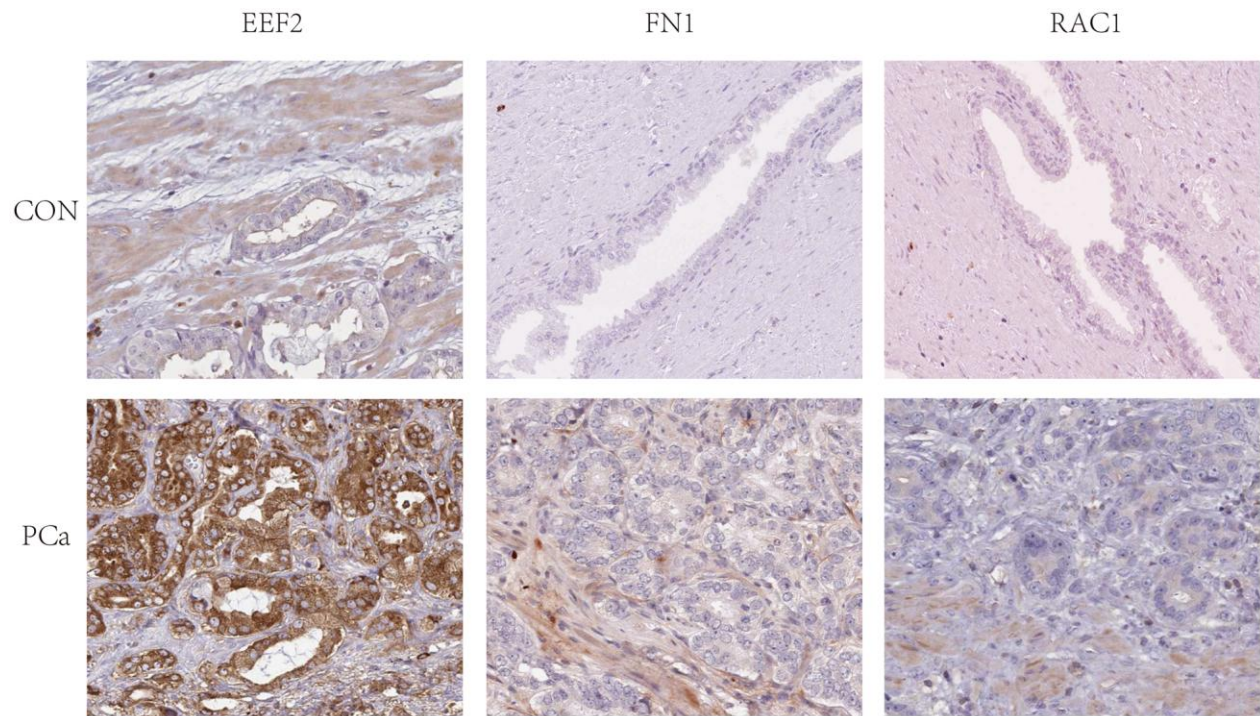

**Supplementary Figure 2.**

Supplement: Supplementary Figures [file aging-13-203234-s001.pdf]
