# Supplementary material for: Prognostic signatures associated with high infiltration of Tregs in bone metastatic prostate cancer
Source: Aging (Albany NY). 2021 Jul 6;13(13):17442–61. doi: 10.18632/aging.203234 (PMC8312432; doi:10.18632/aging.203234)
Supplement: Supplementary Table 1 [file aging-13-203234-s002.pdf]

## SUPPLEMENTARY TABLE

**Supplementary Table 1. Datasets used in overall survival analysis.**

| Datasets |
|---|
| Prostate Adenocarcinoma (CPC-GENE, Nature 2017) |
| Metastatic Prostate Adenocarcinoma (MCTP, Nature 2012) |
| Prostate Adenocarcinoma (MSKCC, Cancer Cell 2010) |
| Prostate Adenocarcinoma (MSKCC, PNAS 2014) |
| Metastatic Prostate Adenocarcinoma (SU2C/PCF Dream Team, PNAS 2019) |
| Prostate Adenocarcinoma (TCGA, Firehose Legacy) |
| Prostate Adenocarcinoma (TCGA, PanCancer Atlas) |
