# Supplementary material for: Prognostic signatures associated with high infiltration of Tregs in bone metastatic prostate cancer
Source: Aging (Albany NY). 2021 Jul 6;13(13):17442–61. doi: 10.18632/aging.203234 (PMC8312432; doi:10.18632/aging.203234)
Supplement: Supplementary Table 2 [file aging-13-203234-s003.pdf]

Supplementary Table 2. Differentially expressed genes in GSE32269.

ID_REF	Gene	logFC	AveExpr	P.Value	adj.P.Val
204582_s_at	KLK3	8.037370941	10.40322089	1.93E-18	3.53E-14
209854_s_at	KLK2	7.439211174	9.764322446	7.49E-10	2.36E-07
202018_s_at	LTF	-6.168537036	5.933372238	2.17E-06	0.000100542
207269_at	DEFA4	-5.973919773	6.580152077	4.57E-09	8.11E-07
202917_s_at	S100A8	-5.954904438	7.622300131	2.29E-05	0.000617885
217771_at	GOLM1	5.936176455	8.504235882	1.13E-11	1.22E-08
203535_at	S100A9	-5.877918077	5.978122669	3.81E-08	4.17E-06
206834_at	HBD	-5.715854552	7.46127039	5.88E-06	0.000224351
211303_x_at	FOLH1B	5.612676205	8.295171557	2.47E-07	1.77E-05
210244_at	CAMP	-5.602137916	4.724837693	4.89E-10	1.75E-07
211560_s_at	ALAS2	-5.43965268	4.870821144	7.24E-08	6.94E-06
203949_at	MPO	-5.396276033	6.002019544	1.08E-08	1.62E-06
210254_at	MS4A3	-5.309729492	5.058994202	3.10E-09	6.36E-07
206676_at	CEACAM8	-5.260268374	4.654279178	1.33E-10	8.36E-08
205592_at	SLC4A1	-5.255785026	5.4527752	1.89E-07	1.38E-05
204959_at	MNDA	-5.228435761	4.876136601	2.32E-10	1.04E-07
205863_at	S100A12	-5.185885307	6.387312937	1.66E-09	4.10E-07
205950_s_at	CA1	-5.184876775	7.096374952	7.56E-08	7.20E-06
210746_s_at	EPB42	-5.169386408	4.326183119	3.12E-12	5.20E-09
214146_s_at	PPBP	-5.149288005	5.533566935	9.86E-08	8.51E-06
212531_at	LCN2	-5.147394835	4.957622348	9.01E-12	1.03E-08
211743_s_at	PRG2	-5.046289345	4.468690237	1.79E-10	9.28E-08
206111_at	RNASE2	-5.011168133	5.460420957	2.54E-09	5.67E-07
209706_at	NKX3-1	4.998370408	10.13276801	6.55E-17	5.98E-13
202489_s_at	FXYD3	4.916497449	7.323390673	4.81E-06	0.000190938
216804_s_at	PDLIM5	4.837771814	7.299595295	5.81E-07	3.47E-05
204393_s_at	ACPP	4.77673178	9.792987521	1.09E-09	2.93E-07
209173_at	AGR2	4.752803609	7.249918589	0.00017565	0.002912325
211821_x_at	GYPA	-4.740591545	4.448583834	6.74E-10	2.16E-07
206871_at	ELANE	-4.73800018	4.486333754	2.95E-08	3.45E-06
219672_at	AHSP	-4.712706837	5.798658883	9.86E-10	2.82E-07
205653_at	CTSG	-4.589247341	5.568067063	3.86E-08	4.18E-06
206207_at	CLC	-4.588409007	5.370869082	2.17E-10	1.02E-07
214407_x_at	GYPB	-4.584109716	5.518673674	6.24E-09	1.07E-06
213975_s_at	LYZ	-4.532778247	6.940698481	8.36E-06	0.000296117
201596_x_at	KRT18	4.526832868	9.462386505	9.43E-14	2.46E-10
209114_at	TSPAN1	4.523835777	7.426593728	1.66E-05	0.000494253
209301_at	CA2	-4.455620309	5.520968582	1.92E-07	1.40E-05
210946_at	PPAP2A	4.415333421	9.987300504	1.90E-09	4.40E-07
210586_x_at	RHD	-4.389967847	4.227618824	8.13E-11	5.51E-08

205542_at	STEAP1	4.373987012	8.853356283	7.36E-09	1.22E-06
206177_s_at	ARG1	-4.372951289	5.427514809	6.36E-09	1.08E-06
202088_at	SLC39A6	4.369158806	10.47138062	6.80E-13	1.24E-09
206390_x_at	PF4	-4.35973938	5.695672723	1.05E-08	1.60E-06
206937_at	SPTA1	-4.351701465	5.407947347	1.17E-08	1.68E-06
201858_s_at	SRGN	-4.194102628	5.927343284	1.04E-06	5.56E-05
201689_s_at	TPD52	4.178041458	7.058228456	1.97E-08	2.54E-06
206851_at	RNASE3	-4.17578055	5.544300232	2.40E-06	0.000109931
205593_s_at	PDE9A	4.158659006	6.622045062	3.21E-07	2.15E-05
206145_at	RHAG	-4.146054764	5.086338894	2.56E-09	5.67E-07
212768_s_at	OLFM4	-4.126021536	4.082679534	3.42E-11	3.29E-08
209930_s_at	NFE2	-3.999855841	4.336510126	1.77E-09	4.24E-07
202023_at	EFNA1	3.994024625	6.695391009	9.37E-16	4.28E-12
202363_at	SPOCK1	3.980180925	7.37162779	2.18E-05	0.000597361
214404_x_at	SPDEF	3.959985884	6.751146435	1.72E-09	4.20E-07
217973_at	DCXR	3.952998914	7.117064548	1.09E-06	5.75E-05
212186_at	ACACA	3.942293427	7.746671422	2.70E-06	0.000122066
212741_at	MAOA	3.892812825	8.658106063	9.62E-05	0.001823064
222258_s_at	SH3BP4	3.877522706	7.359416513	1.44E-09	3.66E-07
200606_at	DSP	3.861341971	8.516959331	6.70E-10	2.16E-07
201951_at	ALCAM	3.84867211	6.673858227	9.15E-07	4.99E-05
218186_at	RAB25	3.816046666	6.849095024	9.19E-08	8.16E-06
201662_s_at	ACSL3	3.803684604	8.849882802	2.38E-07	1.71E-05
219476_at	C1orf116	3.793365748	6.69338285	1.59E-05	0.000481538
218211_s_at	MLPH	3.779733333	9.929979166	5.16E-12	7.12E-09
205936_s_at	HK3	-3.760733334	4.288025052	8.75E-06	0.000305507
201464_x_at	JUN	3.752857071	7.102141342	7.00E-12	8.54E-09
221627_at	TRIM10	-3.731298835	6.092402985	1.51E-10	8.90E-08
204466_s_at	SNCA	-3.724930463	6.618720737	1.30E-07	1.05E-05
205922_at	VNN2	-3.69405623	4.212822269	4.28E-11	3.40E-08
204446_s_at	ALOX5	-3.690943509	5.954758824	1.65E-09	4.10E-07
220001_at	PADI4	-3.686748427	4.487092356	2.62E-09	5.70E-07
203879_at	PIK3CD	-3.679172283	4.84966143	7.97E-09	1.29E-06
200636_s_at	PTPRF	3.666419681	8.002089558	8.36E-10	2.55E-07
206515_at	CYP4F3	-3.664181766	4.673060854	3.96E-11	3.29E-08
220811_at	PRG3	-3.663955319	4.825147343	2.14E-08	2.71E-06
213587_s_at	ATP6V0E2	3.662303169	8.354418878	1.17E-07	9.74E-06
205786_s_at	ITGAM	-3.646965942	5.865418614	7.17E-08	6.93E-06
201839_s_at	EPCAM	3.631596072	8.981131963	3.42E-10	1.39E-07
211110_s_at	AR	3.592248149	7.740881471	4.20E-07	2.68E-05
203662_s_at	TMOD1	-3.577234077	3.94799955	5.24E-14	1.60E-10
210297_s_at	MSMB	3.54508374	6.566350378	0.001424076	0.012979934
203215_s_at	MYO6	3.544510248	7.120736177	6.92E-06	0.00025551
200947_s_at	GLUD1	3.541222548	7.039131486	8.26E-07	4.63E-05

203757_s_at	CEACAM6	-3.518603501	5.245940847	3.20E-07	2.15E-05
209555_s_at	CD36	-3.516752935	5.908070917	1.80E-06	8.68E-05
217775_s_at	RDH11	3.513234313	8.334388742	6.86E-07	4.05E-05
201162_at	IGFBP7	3.512606158	8.402531776	8.95E-05	0.001723866
205709_s_at	CDS1	3.511648979	6.152708131	1.53E-07	1.19E-05
219455_at	C7orf63	3.505478807	6.066297794	0.000325865	0.004538777
213555_at	RWDD2A	3.490847427	6.807241378	3.00E-09	6.28E-07
215047_at	TRIM58	-3.485188351	4.573973956	3.41E-07	2.26E-05
206385_s_at	ANK3	3.483758911	8.645862669	2.11E-06	9.80E-05
210504_at	KLF1	-3.40841802	5.107653452	3.32E-09	6.59E-07
212085_at	SLC25A6	3.401768416	8.077578518	4.02E-05	0.000946334
213050_at	COBL	3.383963549	6.275356912	1.14E-07	9.54E-06
214765_s_at	NAAA	3.380777862	6.531690138	2.83E-08	3.38E-06
212095_s_at	MTUS1	3.375339554	6.886916671	2.85E-10	1.24E-07
201131_s_at	CDH1	3.371942084	9.124807076	2.53E-08	3.06E-06
221577_x_at	GDF15	3.36466719	6.287773109	0.000121478	0.002199594
202458_at	PRSS23	3.364159205	7.463991818	9.36E-06	0.000319271
207384_at	PGLYRP1	-3.355935362	3.73695332	3.84E-10	1.46E-07
220723_s_at	CWH43	3.341615012	6.934278809	4.99E-06	0.000196314
203786_s_at	TPD52L1	3.333801619	7.833702456	2.78E-07	1.94E-05
207072_at	IL18RAP	-3.331868914	4.897262641	3.35E-08	3.76E-06
200929_at	TMED10	3.316525967	7.249400016	7.40E-06	0.000268695
211161_s_at	COL3A1	3.311112564	8.51294374	3.79E-05	0.000905269
205391_x_at	ANK1	-3.308850013	4.76745535	7.17E-06	0.000262113
206283_s_at	TAL1	-3.306219163	5.49321277	3.99E-10	1.49E-07
220942_x_at	FAM162A	3.303536911	8.883944374	5.39E-06	0.000209033
212158_at	SDC2	3.298229925	6.801728939	0.000100257	0.001894112
206461_x_at	MT1H	3.271945912	6.846013897	0.000205846	0.00325369
202457_s_at	PPP3CA	3.266509079	8.929277676	2.65E-07	1.87E-05
204235_s_at	GULP1	3.260330564	6.778708166	4.46E-08	4.63E-06
204862_s_at	NME3	3.258724988	6.771350731	3.26E-09	6.55E-07
211596_s_at	LRIG1	3.256556078	7.158867826	7.27E-06	0.000265378
205353_s_at	PEBP1	3.255556383	6.794235252	1.55E-08	2.09E-06
206697_s_at	HP	-3.240527816	5.513227263	4.51E-06	0.000182033
200770_s_at	LAMC1	3.229673662	6.771717492	7.60E-07	4.33E-05
200755_s_at	CALU	3.228924095	6.461680527	4.99E-06	0.000196314
214084_x_at	NCF1	-3.22134188	4.020954406	3.82E-11	3.29E-08
202286_s_at	TACSTD2	3.220522558	9.349218101	5.28E-06	0.000205977
221734_at	PRRC1	3.218545444	6.763751609	1.19E-08	1.69E-06
209389_x_at	DBI	3.217882487	9.457605957	3.25E-05	0.000805831
214181_x_at	LST1	-3.217511348	4.049749902	5.56E-10	1.85E-07
202499_s_at	SLC2A3	-3.214543218	5.107186062	1.31E-09	3.39E-07
201005_at	CD9	3.213922508	8.87827991	9.32E-08	8.19E-06
209395_at	CHI3L1	-3.212064665	3.814930254	1.06E-09	2.93E-07

37892_at	COL11A1	3.209967095	7.242201532	0.000141338	0.0024743
211719_x_at	FN1	3.203446842	10.20917051	0.000195165	0.003122647
219682_s_at	TBX3	3.202267638	6.02460726	3.38E-06	0.000144732
211689_s_at	TMPRSS2	3.199043131	6.202864977	0.00055294	0.006622895
204007_at	FCGR3B	-3.197866072	6.473929987	9.18E-08	8.16E-06
209624_s_at	MCCC2	3.178451967	6.09307994	0.001001116	0.010021024
203917_at	CXADR	3.175892346	8.843609026	7.67E-06	0.000276542
202890_at	MAP7	3.172700154	7.002151233	6.15E-08	6.08E-06
208581_x_at	MT1X	3.165051921	7.026120946	0.000344345	0.004689043
208998_at	UCP2	-3.155576916	5.514083968	0.000276937	0.004060172
220615_s_at	FAR2	-3.146234812	4.061051034	1.47E-10	8.90E-08
200966_x_at	ALDOA	3.139736321	7.557009864	0.00128456	0.012128049
204882_at	ARHGAP25	-3.134138709	3.757270186	2.97E-16	1.81E-12
218100_s_at	IFT57	3.132855981	8.142715348	3.70E-07	2.39E-05
213868_s_at	DHRS7	3.13229441	6.237725592	0.000832085	0.008801138
208612_at	PDIA3	3.131309893	7.052095956	5.42E-07	3.28E-05
203557_s_at	PCBD1	3.122992126	6.921170584	9.21E-07	5.02E-05
203954_x_at	CLDN3	3.107463752	6.730682187	1.60E-05	0.00048429
206077_at	KEL	-3.102123331	4.386235009	2.32E-08	2.86E-06
200616_s_at	MLEC	3.09571719	7.689231686	1.20E-06	6.25E-05
210130_s_at	TM7SF2	3.094278275	6.895688656	1.28E-05	0.000412361
207332_s_at	TFRC	-3.084735754	8.586522939	6.47E-07	3.85E-05
204430_s_at	SLC2A5	-3.082748056	3.803089738	4.93E-13	1.00E-09
209094_at	DDAH1	3.074412355	8.46864292	1.67E-10	9.28E-08
217820_s_at	ENAH	3.068316132	8.085623362	1.88E-10	9.28E-08
203180_at	ALDH1A3	3.066628692	7.178411071	0.000305304	0.004351836
210215_at	TFR2	-3.060928783	3.996521543	6.84E-11	5.21E-08
205268_s_at	ADD2	-3.059954067	5.050553118	1.09E-07	9.27E-06
220116_at	KCNN2	3.057503772	6.093158764	0.001330366	0.012368951
208117_s_at	LAS1L	3.056062076	8.135653793	0.000301605	0.004316262
207677_s_at	NCF4	-3.055711165	3.808142382	5.45E-12	7.12E-09
203946_s_at	ARG2	3.051468265	7.342951289	0.000502715	0.006186847
207857_at	LILRA2	-3.049129065	4.591530111	1.08E-09	2.93E-07
201849_at	BNIP3	3.045861792	7.922461224	9.79E-07	5.28E-05
208712_at	CCND1	3.045569206	6.934901332	1.56E-05	0.000474094
205379_at	CBR3	3.034048681	5.633751141	3.01E-05	0.000763632
213572_s_at	SERPINB1	-3.027162513	5.210110911	4.38E-10	1.60E-07
207341_at	PRTN3	-3.024782512	4.661847282	8.34E-08	7.75E-06
209083_at	CORO1A	-3.009860267	4.258333291	5.24E-07	3.19E-05
206352_s_at	PEX10	3.006145402	6.322993773	1.28E-05	0.000411019
210044_s_at	LYL1	-3.002619606	4.359269653	1.23E-09	3.21E-07
218270_at	MRPL24	2.997877399	7.532638102	2.76E-06	0.000124162
213892_s_at	APRT	2.996525219	6.415589757	2.15E-05	0.000591895
214063_s_at	TF	-2.994948808	5.37653379	1.41E-05	0.000445165

203355_s_at	PSD3	2.98993773	6.446839235	4.08E-09	7.46E-07
214575_s_at	AZU1	-2.989771393	4.062025559	5.37E-06	0.00020861
213888_s_at	TRAF3IP3	-2.987352597	4.486415644	9.02E-08	8.08E-06
208309_s_at	MALT1	2.986006372	5.969430398	0.000648972	0.007344311
208890_s_at	PLXNB2	2.982910826	6.751397857	3.35E-05	0.000821919
209498_at	CEACAM1	-2.974026031	5.494709351	1.34E-05	0.000426552
200643_at	HDLBP	2.97010634	7.253374278	0.000266931	0.003949535
210809_s_at	POSTN	2.964805855	8.29902473	0.001852486	0.015619302
209949_at	NCF2	-2.961925852	6.364382612	7.45E-07	4.28E-05
201744_s_at	LUM	2.961061033	7.958337413	0.000573935	0.006815666
221732_at	CANT1	2.957734362	6.142993436	7.18E-05	0.001474855
218454_at	PLBD1	-2.95533724	7.079003723	2.40E-09	5.48E-07
36830_at	MIPEP	2.952664903	5.721731749	0.001501522	0.013462215
207329_at	MMP8	-2.949851292	4.747228169	9.29E-08	8.19E-06
205237_at	FCN1	-2.938848582	5.571015585	1.08E-06	5.75E-05
219819_s_at	MRPS28	2.929962343	6.219116272	2.72E-05	0.000707177
213603_s_at	RAC2	-2.925349965	6.728974942	2.57E-09	5.67E-07
219855_at	NUDT11	2.920568229	6.208750896	0.000538364	0.00651595
206522_at	MGAM	-2.920541709	3.828194515	3.78E-11	3.29E-08
208613_s_at	FLNB	2.9123985	5.987985094	2.90E-05	0.000745322
200632_s_at	NDRG1	2.91210602	9.864247305	1.46E-07	1.15E-05
209228_x_at	TUSC3	2.907648261	6.638726019	2.01E-08	2.57E-06
217716_s_at	SEC61A1	2.907299131	6.757815406	0.002253254	0.017877445
211980_at	COL4A1	2.906539796	8.53708084	1.95E-05	0.000548214
208820_at	PTK2	2.904165616	7.648342125	5.06E-09	8.82E-07
209230_s_at	NUPR1	2.892044312	7.665159729	8.49E-05	0.001666954
203478_at	NDUFC1	2.88214524	7.329750468	0.000370153	0.004948368
200968_s_at	PPIB	2.880329974	8.155295013	5.20E-07	3.18E-05
219087_at	ASPN	2.879508028	6.841017398	0.000585011	0.006889039
205513_at	TCN1	-2.879149496	5.708485707	7.60E-06	0.00027457
208950_s_at	ALDH7A1	2.875631323	6.834938507	0.000103717	0.00195141
211423_s_at	SC5D	2.871302187	8.145957561	1.25E-05	0.00040331
201135_at	ECHS1	2.869503016	9.367364846	4.24E-05	0.000983365
219890_at	CLEC5A	-2.868412044	5.159572995	1.84E-10	9.28E-08
209138_x_at	IGLC1	-2.864238002	8.854246467	0.00982674	0.049823684
203130_s_at	KIF5C	2.854897448	8.666046395	9.46E-09	1.45E-06
217875_s_at	PMEPA1	2.845849496	5.468363978	2.00E-05	0.000558894
210140_at	CST7	-2.842451224	4.952718346	4.77E-06	0.000190109
206371_at	FOLR3	-2.835655351	4.090271591	1.30E-08	1.81E-06
210262_at	CRISP2	-2.833771476	5.111683022	0.000443303	0.005591127
204563_at	SELL	-2.830177632	5.564942387	4.22E-08	4.49E-06
203666_at	CXCL12	-2.827027919	6.990249196	2.88E-09	6.20E-07
220751_s_at	FAXDC2	-2.821735191	3.80795687	3.33E-10	1.39E-07
203760_s_at	SLA	-2.813520211	4.644751153	5.98E-05	0.001294096

209163_at	CYB561	2.80615121	7.232729741	4.01E-09	7.41E-07
200681_at	GLO1	2.794132766	10.24701198	2.88E-07	1.99E-05
210041_s_at	PGM3	2.787471136	5.584796719	4.52E-05	0.001032966
208438_s_at	FGR	-2.779670115	5.694427674	5.00E-07	3.09E-05
214020_x_at	ITGB5	2.779459915	6.759706302	1.01E-06	5.41E-05
201061_s_at	STOM	-2.778090983	7.931412953	2.33E-08	2.86E-06
219014_at	PLAC8	-2.773908952	5.73899072	6.80E-08	6.68E-06
217028_at	CXCR4	-2.773066931	6.557027746	1.23E-05	0.000399429
202371_at	TCEAL4	2.768304446	8.656021512	2.28E-08	2.84E-06
209796_s_at	CNPY2	2.760843335	6.669683193	1.78E-07	1.32E-05
201289_at	CYR61	2.752066612	5.954192644	3.29E-06	0.000141599
209917_s_at	TP53TG1	2.738185974	5.563595668	3.74E-05	0.000896705
211382_s_at	TACC2	2.734279083	5.79758597	3.57E-07	2.32E-05
208764_s_at	ATP5G2	2.728987373	8.081498968	8.66E-07	4.79E-05
211742_s_at	EVI2B	-2.728289804	5.065545823	7.12E-08	6.93E-06
222067_x_at	HIST1H2BD	2.728034711	6.831258182	0.00019471	0.003118091
221748_s_at	TNS1	-2.727809352	6.510755609	1.43E-07	1.14E-05
202893_at	UNC13B	2.719276234	8.1583817	1.68E-07	1.28E-05
206881_s_at	LILRA3	-2.718094613	3.705598928	5.53E-10	1.85E-07
209531_at	GSTZ1	2.717051063	5.656358147	0.001710982	0.014780556
220000_at	SIGLEC5	-2.712890006	4.519722937	1.90E-09	4.40E-07
212592_at	IGJ	-2.712025634	4.993494435	0.000107255	0.001997424
200654_at	P4HB	2.699909344	9.930120717	6.94E-05	0.001442102
201825_s_at	SCCPDH	2.699599389	6.567227643	9.57E-08	8.33E-06
219736_at	TRIM36	2.699113582	5.890148536	1.64E-06	8.15E-05
201196_s_at	AMD1	2.697094115	8.310628825	5.77E-05	0.001258741
210510_s_at	NRP1	2.695714733	5.394012557	0.004377354	0.028645051
218123_at	C21orf59	2.695275772	6.802792605	2.97E-05	0.000757948
207540_s_at	SYK	-2.695205293	4.429442693	2.14E-05	0.00059006
214735_at	IPCEF1	-2.69302507	4.449575018	2.22E-08	2.80E-06
213793_s_at	HOMER1	2.692473368	5.418858376	7.69E-07	4.37E-05
202722_s_at	GFPT1	2.691786715	7.891869952	7.48E-07	4.28E-05
218723_s_at	RGCC	-2.687478414	6.01798562	5.59E-05	0.001229321
218261_at	AP1M2	2.687351152	5.953456059	4.74E-05	0.001073037
212115_at	HN1L	2.67672141	9.102991742	3.81E-06	0.000159801
218333_at	DERL2	2.673897711	6.93167422	1.62E-05	0.000487145
213032_at	NFIB	2.662405985	5.5443611	6.14E-06	0.000231821
212013_at	PXDN	2.652621348	6.766719014	0.000651794	0.007371682
205258_at	INHBB	2.649649386	6.694618256	5.58E-06	0.000215689
37986_at	EPOR	-2.648313252	4.893563642	3.19E-07	2.15E-05
221880_s_at	FAM174B	2.64794203	6.000633761	3.54E-10	1.41E-07
201116_s_at	CPE	2.643007531	6.627345668	0.000872606	0.009124197
201288_at	ARHGDIB	-2.632163335	6.574456874	8.90E-06	0.000308174
217977_at	MSRB1	-2.629236806	6.797414551	0.000469655	0.005878884

202404_s_at	COL1A2	2.628831935	9.846269673	0.003787388	0.025820578
204174_at	ALOX5AP	-2.616934823	6.072674216	1.08E-08	1.62E-06
201616_s_at	CALD1	2.616663137	5.266957783	8.00E-05	0.001594085
209146_at	MSMO1	2.607033184	6.508283159	3.44E-06	0.000146711
209759_s_at	ECI1	2.605603101	6.481356431	7.26E-05	0.001482836
212166_at	XPO7	-2.598032663	5.662039014	2.73E-05	0.000708116
203430_at	HEBP2	2.596388334	8.921294122	0.00034109	0.004673367
214669_x_at	IGKC	-2.59185423	6.561685132	0.006122051	0.036023187
202148_s_at	PYCR1	2.588304883	7.21801353	4.48E-08	4.63E-06
208527_x_at	HIST1H2BE	2.587993021	5.338549528	0.000225221	0.003487581
219266_at	ZNF350	2.586659493	6.038084905	5.47E-05	0.001207583
205830_at	CLGN	2.586542404	6.258285527	0.000141385	0.0024743
211928_at	DYNC1H1	2.58574191	7.659407021	1.27E-06	6.56E-05
205990_s_at	WNT5A	2.584437973	6.035083168	0.004979284	0.031238814
202597_at	IRF6	2.584364091	8.071550533	5.09E-08	5.14E-06
219546_at	BMP2K	-2.583665481	4.010330562	1.70E-07	1.29E-05
209803_s_at	PHLDA2	2.581009835	5.434206622	0.001831865	0.015495443
222074_at	UROD	-2.575909403	3.959909889	1.93E-10	9.28E-08
210395_x_at	MYL4	-2.575464346	5.955832748	3.71E-09	7.14E-07
203765_at	GCA	-2.573715761	6.691895452	1.17E-08	1.68E-06
213988_s_at	SAT1	2.572956361	6.642514599	2.46E-05	0.000653069
217901_at	DSG2	2.571666891	7.108209203	3.93E-05	0.000930901
212448_at	NEDD4L	2.571170108	6.631968213	0.005877669	0.035001892
202620_s_at	PLOD2	2.561133969	6.809678414	0.000365379	0.004902459
212328_at	LIMCH1	2.56075476	7.389504096	4.27E-06	0.000175736
203397_s_at	GALNT3	2.557045972	7.473807916	0.000303064	0.004328567
211207_s_at	ACSL6	-2.556103816	4.775456877	3.13E-09	6.36E-07
205270_s_at	LCP2	-2.555100919	4.388000403	5.15E-07	3.16E-05
213416_at	ITGA4	-2.550974745	4.983673749	1.36E-06	6.93E-05
206698_at	XK	-2.549225395	5.107332631	1.27E-06	6.56E-05
209538_at	ZNF32	2.549072096	6.196258531	3.03E-08	3.50E-06
218584_at	TCTN1	2.54776543	6.415533823	9.68E-06	0.000328398
214875_x_at	APLP2	2.54748449	7.173388317	7.71E-05	0.001548761
202933_s_at	YES1	2.54736245	7.158268571	3.69E-07	2.39E-05
222154_s_at	SPATS2L	2.547257426	7.877766924	2.35E-06	0.000107999
200812_at	CCT7	2.546637076	8.104025782	0.000355262	0.00481262
210119_at	KCNJ15	-2.546048377	4.161203907	2.03E-07	1.47E-05
202157_s_at	CELF2	-2.544642703	6.537596388	3.44E-07	2.27E-05
220864_s_at	NDUFA13	2.540747945	8.340534199	0.000732554	0.008026934
211373_s_at	PSEN2	2.540562825	5.962586022	9.85E-08	8.51E-06
221505_at	ANP32E	-2.536685856	6.45254817	4.31E-07	2.74E-05
207697_x_at	LILRB2	-2.533256403	4.764880841	2.05E-06	9.69E-05
208579_x_at	H2BFS	2.528156675	6.142740086	0.00512734	0.031839999
209262_s_at	NR2F6	2.527411909	5.889290352	0.000526408	0.006409418

203186_s_at	S100A4	-2.525062871	6.084499214	0.00013042	0.002326948
216667_at	ECRP	-2.523514401	4.415636547	3.01E-07	2.06E-05
218654_s_at	MRPS33	2.522705699	8.208019725	3.14E-06	0.000136745
213817_at	IRAK3	-2.521380802	4.003305288	9.03E-09	1.41E-06
202241_at	TRIB1	2.520421123	8.807832947	1.17E-06	6.08E-05
212104_s_at	RBFOX2	2.511818501	6.58029501	3.44E-08	3.84E-06
206214_at	PLA2G7	2.511809212	7.644723188	0.000225872	0.003494705
204798_at	MYB	-2.511797371	6.23778627	2.90E-07	1.99E-05
208822_s_at	DAP3	2.505309488	8.392688613	9.37E-05	0.001782263
210108_at	CACNA1D	2.50387045	5.68808356	0.002919288	0.021458176
212812_at	SERINC5	2.499200458	8.39271176	1.98E-06	9.36E-05
203470_s_at	PLEK	-2.498559604	4.081586842	3.13E-05	0.000785456
205160_at	PEX11A	2.497098336	6.685897219	1.54E-07	1.20E-05
200806_s_at	HSPD1	2.496330989	7.945292885	3.70E-05	0.000889809
215051_x_at	AIF1	-2.495775546	5.149468408	0.00039151	0.00513521
212417_at	SCAMP1	2.494187608	5.238345406	0.000227786	0.003515401
204674_at	LRMP	-2.490728481	4.810421222	9.99E-06	0.00033509
218025_s_at	ECI2	2.487657955	7.785151668	0.000435658	0.00552518
210715_s_at	SPINT2	2.482943656	7.978091835	3.78E-05	0.000904055
204832_s_at	BMPR1A	2.481242128	5.647067516	1.74E-06	8.50E-05
201704_at	ENTPD6	2.481047825	6.243215657	2.98E-05	0.000757948
210036_s_at	KCNH2	-2.480139152	6.595867572	4.19E-08	4.49E-06
204102_s_at	EEF2	2.476787783	9.433331207	3.96E-06	0.000163797
218375_at	NUDT9	2.476141409	7.86847421	3.19E-06	0.000138468
203791_at	DMXL1	2.474674716	7.657974061	2.37E-05	0.000634731
202600_s_at	NRIP1	2.474129484	6.631679279	2.04E-05	0.000568395
201937_s_at	DNPEP	2.473952391	5.454069977	0.000395143	0.005165387
218308_at	TACC3	-2.469067177	5.202870751	0.000976987	0.009838729
214598_at	CLDN8	2.462486713	6.288388519	0.001338311	0.01242935
212956_at	TBC1D9	2.461536913	7.087345633	1.44E-05	0.000448432
202756_s_at	GPC1	2.460606264	5.612423621	0.000180696	0.002958434
206589_at	GFI1	-2.460232706	3.629067692	1.85E-10	9.28E-08
219806_s_at	SMCO4	2.456403171	8.338139653	1.62E-05	0.000487732
209693_at	ASTN2	2.453650728	6.027319422	1.54E-05	0.000471691
204220_at	GMFG	-2.451702042	6.301936904	5.89E-06	0.000224351
209374_s_at	IGHM	-2.449028844	5.734351757	0.008340876	0.044523624
216905_s_at	ST14	2.446501367	5.117421683	7.53E-05	0.001520129
209116_x_at	HBB	-2.440844134	11.18158864	0.004974983	0.031222543
221215_s_at	RIPK4	2.439376034	5.805466023	0.001286518	0.012140269
214835_s_at	SUCLG2	2.438452738	7.207827292	2.61E-06	0.00011796
201681_s_at	DLG5	2.437907938	7.296232761	0.000237486	0.003616277
205286_at	TFAP2C	2.435704899	6.178934558	3.59E-09	7.05E-07
201417_at	SOX4	2.435467805	6.701668715	9.28E-05	0.001769868
204068_at	STK3	2.434608797	6.484991892	1.42E-05	0.000447438

203875_at	SMARCA1	2.43405063	5.782182075	0.000123317	0.002221822
205547_s_at	TAGLN	2.432531308	6.599992219	8.60E-05	0.001681698
207008_at	CXCR2	-2.432225321	3.959244323	3.42E-10	1.39E-07
206060_s_at	PTPN22	-2.431143176	3.830081578	1.33E-08	1.82E-06
212640_at	PTPLB	2.430216792	8.059745085	0.000383157	0.005070314
203152_at	MRPL40	2.429772145	6.815294608	0.002483768	0.0191578
206858_s_at	HOXC6	2.429551874	8.110931631	0.000117029	0.002140232
203343_at	UGDH	2.429011557	7.409369	1.65E-06	8.16E-05
212624_s_at	CHN1	2.427230184	6.341743505	0.002093152	0.016960372
206493_at	ITGA2B	-2.426734559	5.16388028	1.89E-08	2.45E-06
219109_at	SPAG16	2.424037525	5.971578382	5.03E-07	3.09E-05
219405_at	TRIM68	2.423970483	6.028636108	0.000992902	0.009949722
208731_at	RAB2A	2.423648359	7.615804759	3.29E-06	0.000141599
200658_s_at	PHB	2.42177851	7.640454227	0.000172997	0.002886644
213306_at	MPDZ	2.421052046	5.954425038	0.002123879	0.017100369
213506_at	F2RL1	2.420304829	6.172738317	0.001858888	0.015651633
214774_x_at	TOX3	2.418097141	5.41070432	0.002894632	0.0213112
204875_s_at	GMDS	2.417570923	5.618860632	0.001988082	0.016392262
59625_at	NOL3	2.41672069	5.950145293	3.22E-08	3.68E-06
201614_s_at	RUVBL1	2.412041309	5.416329932	0.000880506	0.009175325
207877_s_at	NVL	2.411712669	5.205252812	0.005774665	0.034602582
208737_at	ATP6V1G1	2.409151097	9.083964991	1.32E-06	6.70E-05
220161_s_at	EPB41L4B	2.408699235	8.006519009	1.78E-05	0.000520433
220560_at	C11orf21	-2.408084443	5.453918502	2.88E-08	3.40E-06
201911_s_at	FARP1	2.404759306	5.8510357	7.62E-09	1.25E-06
213733_at	MYO1F	-2.404677473	5.199937837	1.19E-05	0.000390001
204720_s_at	DNAJC6	-2.403966145	4.11972679	1.89E-08	2.45E-06
207738_s_at	NCKAP1	2.40186706	7.166797863	8.63E-06	0.000302823
204784_s_at	MLF1	2.400261191	5.654296949	8.67E-05	0.001690081
205896_at	SLC22A4	-2.39971176	5.573248936	1.28E-10	8.36E-08
217963_s_at	NGFRAP1	2.396775266	10.37241294	1.43E-05	0.000448413
205597_at	SLC44A4	2.392515784	5.834885523	2.59E-06	0.000117565
219121_s_at	ESRP1	2.392499614	5.594648701	0.000567248	0.006758199
213309_at	PLCL2	-2.386262751	4.878064592	1.64E-05	0.000490438
222075_s_at	OAZ3	2.384802369	5.570815848	5.19E-06	0.000202943
201193_at	IDH1	2.384525472	9.287312046	1.35E-05	0.000430138
201415_at	GSS	2.38395446	7.171329286	1.04E-06	5.54E-05
205273_s_at	PITRM1	2.382140636	7.339867184	2.12E-05	0.000588153
217919_s_at	MRPL42	2.381399926	5.532235622	0.000358756	0.004843802
217786_at	PRMT5	2.380774152	6.058277449	0.00630634	0.036779222
217165_x_at	MT1F	2.379587317	5.547561826	0.007140423	0.040155
218487_at	ALAD	-2.375862956	6.416704502	1.33E-07	1.07E-05
208757_at	TMED9	2.374979081	7.561317007	0.000817566	0.008662604
222209_s_at	TMEM135	2.373201052	5.961542583	0.000580227	0.006867882

200823_x_at	RPL29	2.373174584	8.928380967	0.000108339	0.002012858
AFFX-HUMGAPDH/M33197_M_at	GAPDH	2.372722544	10.36538384	0.000803423	0.008576204
221824_s_at	8-Mar	-2.368367373	6.014309471	3.27E-08	3.72E-06
201194_at	SEPW1	2.367087853	8.363150487	6.16E-05	0.001323751
209873_s_at	PKP3	2.367022641	5.684857664	4.87E-07	3.02E-05
217534_at	FAM49B	-2.366202979	4.6265511	3.16E-07	2.14E-05
221504_s_at	ATP6V1H	2.363340731	6.779151171	4.37E-05	0.001005435
205504_at	BTK	-2.35921226	4.464430426	1.11E-05	0.000366181
221942_s_at	GUCY1A3	2.357696264	8.557168722	2.84E-06	0.000126306
200837_at	BCAP31	2.356224012	8.81750409	0.000108881	0.002019487
218531_at	TMEM134	2.353217729	7.119077661	8.61E-09	1.36E-06
212110_at	SLC39A14	2.35210869	6.951564643	2.00E-05	0.000558894
203845_at	KAT2B	-2.351624402	6.042819172	1.78E-09	4.24E-07
218046_s_at	MRPS16	2.35009606	5.923252839	0.000659887	0.007435625
212698_s_at	10-Sep	2.344709693	7.17160903	3.73E-06	0.000157359
219990_at	E2F8	-2.342913659	4.454826406	3.13E-06	0.000136433
222162_s_at	ADAMTS1	2.340619682	5.940843098	0.007050149	0.039806458
212062_at	ATP9A	2.33775499	7.116750636	2.21E-05	0.000602806
212915_at	PDZRN3	2.335857104	5.185017282	1.42E-05	0.000447438
201428_at	CLDN4	2.332643082	5.02574186	2.07E-06	9.72E-05
218069_at	DCTPP1	2.332089491	7.665793099	2.65E-05	0.000693615
203116_s_at	FECH	-2.331475849	7.142524588	1.11E-08	1.64E-06
209459_s_at	ABAT	2.330498235	5.986380732	3.46E-05	0.000840926
218816_at	LRRC1	2.32692908	5.379240472	9.64E-05	0.001825819
218546_at	C1orf115	2.32635125	6.875346648	3.23E-05	0.000805831
201889_at	FAM3C	2.325928742	6.623688611	1.94E-05	0.0005479
212588_at	PTPRC	-2.325507436	6.177395745	0.000405558	0.005245533
203590_at	DYNC1LI2	2.324255407	6.873905111	1.76E-06	8.54E-05
213160_at	DOCK2	-2.324205325	4.33022506	8.60E-06	0.000302455
208206_s_at	RASGRP2	-2.320715597	3.811434169	9.50E-08	8.31E-06
213150_at	HOXA10	2.319871818	6.022793678	7.91E-05	0.001578079
201413_at	HSD17B4	2.318194107	9.208662274	0.001636439	0.014312384
201022_s_at	DSTN	2.316888227	9.983281104	5.67E-07	3.40E-05
220416_at	ATP8B4	-2.316645245	4.807422529	0.000700592	0.007781614
205180_s_at	ADAM8	-2.314994513	3.854234996	1.40E-08	1.89E-06
220496_at	CLEC1B	-2.311986448	4.711320857	2.60E-07	1.85E-05
203038_at	PTPRK	2.307922968	7.653604275	4.51E-06	0.000182033
219580_s_at	TMC5	2.306242747	6.30049638	0.00284382	0.021081384
201674_s_at	AKAP1	2.304684876	7.575122207	2.52E-06	0.000114798
36711_at	MAFF	2.304646163	5.508626804	0.000438567	0.005546694
215380_s_at	GGCT	2.304644871	9.358494175	0.000133438	0.00236793
205862_at	GREB1	2.302742058	5.07256023	0.002088641	0.01694896
202974_at	MPP1	-2.299152732	6.646921452	7.81E-08	7.36E-06
209171_at	ITPA	2.298791626	6.955608733	0.000600257	0.006989198

218187_s_at	C8orf33	2.294980199	6.709136083	9.30E-06	0.000319223
205856_at	SLC14A1	-2.291305415	3.986896409	5.22E-08	5.24E-06
202201_at	BLVRB	-2.288262225	7.444183721	0.002255671	0.017888492
203591_s_at	CSF3R	-2.286528331	3.956859185	8.15E-09	1.31E-06
205133_s_at	HSPE1	2.286020579	8.870431902	3.05E-05	0.000772147
206167_s_at	ARHGAP6	2.283121716	7.239727084	6.60E-05	0.001386069
216268_s_at	JAG1	2.283056661	7.912531205	0.000872322	0.009124197
202704_at	TOB1	2.280772788	7.397031796	6.95E-06	0.00025578
55872_at	ZNF512B	2.278961991	5.789939424	0.00014002	0.002459565
212680_x_at	PPP1R14B	2.274641325	5.959157392	0.000540361	0.006524824
204992_s_at	PFN2	2.274307721	8.443209117	1.14E-06	5.97E-05
200970_s_at	SERP1	2.272554104	7.871144459	0.000334718	0.004619862
200832_s_at	SCD	2.269088879	7.437541411	0.00633799	0.036901991
221802_s_at	KIAA1598	2.268228853	7.218532162	4.97E-06	0.000196314
201732_s_at	CLCN3	2.2663864	5.267529557	0.000118551	0.002157278
211979_at	GPR107	2.263701971	5.4784023	6.95E-06	0.00025578
212873_at	HMHA1	-2.262861413	5.213153408	0.000301865	0.004316262
209806_at	HIST1H2BK	2.262060229	8.572896182	8.70E-05	0.00169439
209364_at	BAD	2.261029464	5.305966322	0.000863165	0.009077377
203711_s_at	HIBCH	2.256543446	5.630590286	2.23E-05	0.00060668
205632_s_at	PIP5K1B	-2.254683965	5.488328094	3.22E-08	3.68E-06
221024_s_at	SLC2A10	2.25453395	6.438549631	0.005508261	0.033477928
209193_at	PIM1	-2.251068381	6.287211616	7.22E-08	6.94E-06
204343_at	ABCA3	2.244680937	5.056560626	9.28E-07	5.04E-05
201563_at	SORD	2.242075239	7.84517335	0.004803703	0.030466269
209226_s_at	TNPO1	2.241141126	5.515121352	0.000192037	0.003091526
203438_at	STC2	2.239080787	5.24272818	0.000782316	0.008415881
207339_s_at	LTB	-2.236090367	4.706053386	1.88E-07	1.38E-05
203789_s_at	SEMA3C	2.234948666	6.31182783	0.002762048	0.020667894
212411_at	IMP4	2.234522814	6.6054729	3.59E-05	0.000868994
218086_at	NPDC1	2.230894391	5.789994076	7.60E-05	0.001532587
212686_at	PPM1H	2.227915035	7.058832698	3.62E-05	0.000873711
209121_x_at	NR2F2	2.224072521	6.14403558	4.28E-06	0.000175791
220918_at	RUNX1-IT1	-2.222357193	4.284862825	2.34E-05	0.000629377
202990_at	PYGL	-2.222277155	5.402379299	6.96E-07	4.08E-05
220617_s_at	ZNF532	2.22016653	5.446009869	0.002023266	0.016602776
201438_at	COL6A3	2.219775177	8.740525783	0.001531342	0.013641105
215946_x_at	IGLL3P	-2.21842854	7.302628275	0.000974042	0.009825304
204485_s_at	TOM1L1	2.217955286	6.344294448	4.41E-06	0.000180898
200782_at	ANXA5	2.217818898	8.800944424	1.72E-05	0.000505433
203962_s_at	NEBL	2.21688722	5.969424338	1.78E-05	0.000520433
219577_s_at	ABCA7	-2.216317109	5.061102386	8.51E-06	0.000299865
205927_s_at	CTSE	-2.214670638	5.919182497	3.45E-07	2.27E-05
203642_s_at	COBLL1	2.212701227	7.011683365	1.89E-05	0.000540005

208416_s_at	SPTB	-2.209035586	4.802488495	8.17E-05	0.001619496
205107_s_at	EFNA4	2.205991699	4.998048495	0.00012577	0.0022572
202591_s_at	SSBP1	2.205776484	7.664092321	0.00011518	0.002108968
212829_at	PIP4K2A	-2.203114055	6.982592454	8.85E-08	8.01E-06
212848_s_at	C9orf3	2.202743897	5.643571898	0.00068166	0.007615272
203394_s_at	HES1	2.202572237	7.2381759	8.99E-07	4.95E-05
209369_at	ANXA3	-2.199219627	5.860195133	0.000577263	0.006841852
204019_s_at	SH3YL1	2.198020226	8.280089467	9.46E-05	0.001799264
218573_at	MAGEH1	2.197372934	6.680562654	0.000181127	0.002961839
219938_s_at	PSTPIP2	-2.195609792	4.693575672	5.24E-08	5.24E-06
203741_s_at	ADCY7	-2.195201879	4.982839463	8.35E-08	7.75E-06
214359_s_at	HSP90AB1	2.194445829	6.632220003	0.00030731	0.004366817
206687_s_at	PTPN6	-2.194432544	6.350009972	1.70E-08	2.24E-06
217871_s_at	MIF	2.190467916	7.574578404	2.82E-05	0.000728366
201204_s_at	RRBP1	2.189298269	5.649057298	1.92E-05	0.000547315
219388_at	GRHL2	2.183760929	6.89823339	1.17E-07	9.74E-06
204667_at	FOXA1	2.183736245	6.957794872	1.42E-06	7.18E-05
200872_at	S100A10	2.182745	8.068366697	0.000387366	0.005092843
218214_at	ATG101	2.182510566	6.644638772	2.81E-05	0.000724646
201760_s_at	WSB2	2.181649106	7.433348572	3.25E-05	0.000805831
207556_s_at	DGKZ	-2.175940703	4.808999709	3.31E-05	0.000817664
38964_r_at	WAS	-2.172507063	7.281966733	8.70E-10	2.61E-07
220005_at	P2RY13	-2.172151157	4.06364588	2.68E-07	1.88E-05
209478_at	STRA13	2.17170537	7.396833898	0.000297979	0.0042909
219905_at	ERMAP	-2.171510386	4.302439001	0.000102611	0.001932699
204172_at	CPOX	-2.169697282	6.105106664	1.15E-05	0.000377744
206452_x_at	PPP2R4	2.168271099	5.507712427	0.000450545	0.005670725
210074_at	CTSV	2.161852928	5.52354181	0.002249427	0.017862579
202381_at	ADAM9	2.161629536	7.308683204	6.67E-05	0.001394275
204295_at	SURF1	2.160644853	7.126642073	1.63E-05	0.000488383
213413_at	STON1	2.159727087	6.755919156	0.00135289	0.012527418
202351_at	ITGAV	2.159314763	8.007003042	1.37E-05	0.000434909
200692_s_at	HSPA9	2.158719531	7.941684847	4.48E-06	0.000182033
218689_at	FANCF	2.158223322	4.864086431	0.00194466	0.016165424
203407_at	PPL	2.157849494	4.899124288	0.00202729	0.016625597
205668_at	LY75	-2.155595558	4.308399051	4.13E-08	4.44E-06
218477_at	TMEM14A	2.154439611	7.253181224	0.000224134	0.003479587
218568_at	AGK	2.149429185	5.644955188	1.45E-05	0.000451055
211005_at	LAT	-2.148832255	4.47905793	1.88E-07	1.38E-05
210844_x_at	CTNNA1	2.143262182	8.55466508	0.000712261	0.007870589
220014_at	PRR16	2.141957134	4.974360415	0.006505175	0.037552598
221691_x_at	NPM1	2.140969446	8.906534143	0.000229765	0.003536993
210094_s_at	PARD3	2.140591176	5.550251886	9.60E-07	5.19E-05
202382_s_at	GNPDA1	2.140498811	6.413561155	0.000473309	0.005915238

205573_s_at	SNX7	2.138812692	6.496785101	0.000987911	0.009924459
213342_at	YAP1	2.138016901	5.198447843	0.000170964	0.002868432
203496_s_at	MED1	2.137462011	5.227044861	0.005108754	0.031762433
204600_at	EPHB3	2.137047209	5.807195734	4.50E-06	0.000182033
209503_s_at	PSMC5	2.134218292	7.60981968	0.000691543	0.007702154
203997_at	PTPN3	2.131987739	6.286404067	0.00023039	0.003542841
203335_at	PHYH	2.131577569	7.604551027	0.000177311	0.002925686
213508_at	SPTSSA	2.129736323	6.726221994	2.86E-07	1.99E-05
210944_s_at	CAPN3	-2.129302539	5.813884174	4.32E-08	4.54E-06
200913_at	PPM1G	2.127101134	6.374738006	0.000172745	0.002886644
218180_s_at	EPS8L2	2.126499339	5.613350886	7.34E-05	0.001493729
211297_s_at	CDK7	2.126018139	6.041068392	1.92E-05	0.000546516
209008_x_at	KRT8	2.125083162	7.1337671	0.000177299	0.002925686
200758_s_at	NFE2L1	2.124605213	6.167412698	0.000153673	0.002636376
218176_at	MAGEF1	2.119037248	5.861356387	6.48E-05	0.0013722
200087_s_at	TMED2	2.118297025	9.712682357	3.25E-06	0.000140504
209875_s_at	SPP1	2.116442169	9.563712781	0.001153311	0.011201142
35820_at	GM2A	2.116136245	6.176290548	5.66E-05	0.001241123
214214_s_at	C1QBP	2.116037903	6.3314683	7.15E-05	0.001473415
203518_at	LYST	-2.114357776	5.34536645	4.55E-08	4.68E-06
201830_s_at	NET1	2.111383744	6.196851295	0.000391852	0.00513521
216449_x_at	HSP90B1	2.111191147	7.4495935	1.50E-05	0.000462795
217758_s_at	TM9SF3	2.109771794	8.024476317	9.25E-05	0.001767098
220030_at	STYK1	2.108485769	5.311707962	0.000318127	0.004461715
221019_s_at	COLEC12	2.10566059	7.895522266	0.000550364	0.006604365
213939_s_at	RUFY3	2.105572771	7.049242951	0.000235599	0.003599533
213475_s_at	ITGAL	-2.104006019	3.964625823	3.55E-07	2.32E-05
217979_at	TSPAN13	2.103965395	9.202887724	1.48E-06	7.46E-05
203367_at	DUSP14	2.103449221	6.552606457	7.04E-06	0.000258093
202769_at	CCNG2	2.10279267	8.55391344	0.000526129	0.006409418
204081_at	NRGN	-2.101650508	3.681853815	1.29E-07	1.05E-05
201011_at	RPN1	2.100213929	6.899870362	0.000442822	0.005588908
201432_at	CAT	-2.097206442	8.995656072	9.89E-06	0.000332509
208696_at	CCT5	2.096866591	7.059649577	0.003055599	0.022096003
204167_at	BTD	2.096621986	5.451562234	0.008825076	0.046178254
203647_s_at	FDX1	2.095946605	6.248924346	0.001558022	0.013804799
201113_at	TUFM	2.09493304	7.900406406	0.000591063	0.006913021
218910_at	ANO10	2.092010769	5.350976218	0.00139878	0.012815427
221210_s_at	NPL	-2.091072288	4.392104176	4.76E-05	0.001076405
201831_s_at	USO1	2.090910849	5.815294597	0.000927976	0.009512798
209829_at	FAM65B	-2.09035122	4.53274854	2.91E-05	0.000746834
203767_s_at	STS	2.089074171	5.271606198	0.000253927	0.003822066
219329_s_at	ATRAID	2.085539173	8.220358418	1.86E-05	0.000535797
205110_s_at	FGF13	2.085461522	6.054329957	0.00032214	0.004497168

217975_at	WBP5	2.085016124	8.290253492	3.60E-05	0.000870609
208623_s_at	EZR	2.084049722	6.277661908	6.12E-05	0.001319311
204547_at	RAB40B	2.079540139	5.625556314	1.19E-07	9.86E-06
217761_at	ADI1	2.077691616	9.309761498	0.00016961	0.0028508
218189_s_at	NANS	2.077207958	7.947484676	0.000638051	0.007288369
210473_s_at	GPR125	2.075718712	5.852125243	4.84E-08	4.94E-06
201485_s_at	RCN2	2.075576681	5.832048779	0.003312974	0.02339292
218313_s_at	GALNT7	2.07362066	8.181403667	0.001147586	0.011157395
208799_at	PSMB5	2.070298446	8.562663523	0.000600395	0.006989198
202188_at	NUP93	2.069327968	6.031230089	0.007713238	0.042284083
207957_s_at	PRKCB	-2.069022363	3.693588508	1.22E-08	1.72E-06
217683_at	HBE1	-2.067962662	4.549871023	0.004846772	0.030654401
202284_s_at	CDKN1A	2.067622853	5.66404977	0.000737071	0.008057114
212231_at	FBXO21	2.0668109	7.394215706	1.93E-05	0.000547514
206116_s_at	TPM1	2.064026423	6.019358486	0.002679115	0.020229418
215482_s_at	EIF2B4	2.062598601	7.17081485	7.43E-05	0.001505846
211795_s_at	FYB	-2.061364742	4.71508475	8.37E-06	0.000296117
201178_at	FBXO7	-2.060894152	7.575985738	1.36E-07	1.09E-05
218662_s_at	NCAPG	-2.06011964	6.028561024	3.65E-05	0.000880315
220934_s_at	TMEM223	2.058806713	6.926063009	1.48E-07	1.16E-05
213357_at	GTF2H5	2.058719893	8.138118872	6.28E-05	0.001340732
218163_at	MCTS1	2.057820004	6.005712356	0.003377553	0.023720698
217803_at	GOLPH3	2.057818354	8.547701548	1.39E-05	0.000439367
202124_s_at	TRAK2	-2.057693311	6.094057761	2.16E-07	1.56E-05
219539_at	GEMIN6	2.05742588	6.276418985	0.000511811	0.006275019
217851_s_at	SLMO2	2.056988125	5.82057859	0.000339927	0.004670606
201709_s_at	NIPSNAP1	2.054951212	7.958293006	1.63E-07	1.25E-05
221274_s_at	LMAN2L	2.05462535	6.8181382	0.00026178	0.003899056
218686_s_at	RHBDF1	2.053106552	6.60163163	3.21E-05	0.000803507
219327_s_at	GPRC5C	2.053082369	4.826974028	0.000824057	0.008721267
200862_at	DHCR24	2.051973767	7.388984426	7.17E-05	0.001474855
201568_at	UQCRQ	2.051941801	9.12557791	0.000369157	0.004942267
219373_at	DPM3	2.05168733	6.893623446	0.003638503	0.025100317
220558_x_at	TSPAN32	-2.051280674	5.122576979	3.28E-05	0.00081074
203636_at	MID1	2.04869621	5.870808576	0.002077908	0.016904258
221730_at	COL5A2	2.046427491	6.008642081	0.003296454	0.023303264
208682_s_at	MAGED2	2.045479549	7.601129856	0.003590751	0.024902411
206827_s_at	TRPV6	2.044102173	4.763798148	0.000635257	0.007270865
211038_s_at	CROCCP2	-2.04383101	6.447802416	3.11E-05	0.000784157
202043_s_at	SMS	2.042804338	7.631757427	0.000981806	0.009876381
212068_s_at	PRRC2B	2.038552445	7.071788666	0.001250323	0.011866067
205422_s_at	ITGBL1	2.037859971	5.574044338	0.002603339	0.019826986
202257_s_at	CD2BP2	2.035948242	6.387674975	0.002965198	0.021647724
218793_s_at	SCML1	2.034434904	5.545028476	0.000119449	0.002169307

219191_s_at	BIN2	-2.034026461	3.777430052	1.46E-07	1.15E-05
213523_at	CCNE1	-2.033214202	5.63004081	7.32E-07	4.26E-05
209892_at	FUT4	-2.031174886	5.277073345	1.65E-07	1.27E-05
200897_s_at	PALLD	2.031112691	7.767646283	0.000156244	0.002670463
201962_s_at	RNF41	2.030971549	5.572155457	0.00370926	0.025454012
205613_at	SYT17	2.030324325	5.523188454	0.004427777	0.028827054
221249_s_at	FAM117A	-2.029144143	7.128507023	1.70E-08	2.24E-06
211135_x_at	LILRB3	-2.027053447	3.673890013	2.24E-08	2.81E-06
206558_at	SIM2	2.026817851	6.685176115	1.05E-06	5.56E-05
209250_at	DEGS1	2.0261956	8.033993531	0.001726591	0.014866244
202178_at	PRKCZ	2.025792268	6.734633288	4.07E-05	0.000953317
207854_at	GYPE	-2.023442905	3.70465836	3.65E-09	7.09E-07
205880_at	PRKD1	2.022724797	6.749946846	1.95E-05	0.000548826
200022_at	RPL18	2.017064837	10.98071876	6.40E-05	0.001359971
202243_s_at	PSMB4	2.013351325	7.948874491	0.000762126	0.008256964
210183_x_at	PNN	2.013060744	9.13165979	0.007283746	0.040582347
205898_at	CX3CR1	-2.01285428	4.571634613	2.10E-05	0.000583253
213011_s_at	TPI1	2.012484181	8.32798403	0.000377481	0.005024287
209031_at	CADM1	2.010454309	6.948791453	0.001163056	0.011256383
218578_at	CDC73	2.009938341	6.159589535	0.002330274	0.018345265
220925_at	NAA35	2.00951465	6.778078202	1.05E-05	0.00035107
204205_at	APOBEC3G	-2.009366881	4.943281192	0.000667413	0.007498263
218510_x_at	FAM134B	2.00896118	6.102156305	1.66E-05	0.000492509
212322_at	SGPL1	2.007998536	7.416203063	1.43E-06	7.23E-05
209059_s_at	EDF1	2.006351582	8.380416347	0.000379489	0.005038425
211896_s_at	DCN	2.006308201	8.11263311	0.008545392	0.045219368
205844_at	VNN1	-2.00552308	3.889692277	2.65E-07	1.87E-05
205003_at	DOCK4	2.005245363	6.238751865	1.31E-06	6.68E-05
212727_at	DLG3	2.004865747	6.702843551	7.37E-07	4.27E-05
44654_at	G6PC3	2.004432447	6.029435252	1.36E-05	0.000431007
204613_at	PLCG2	-2.004277192	6.194325476	2.88E-06	0.000126741
204199_at	RALGPS1	2.004120576	5.277069539	0.000879247	0.009167429
204852_s_at	PTPN7	-2.000839825	4.820995876	0.00017627	0.00291995
214709_s_at	KTN1	1.99803212	8.407862977	7.83E-05	0.001565531
217911_s_at	BAG3	1.996217552	6.433541667	6.31E-06	0.000236821
215945_s_at	TRIM2	1.995917461	5.275290269	8.56E-07	4.76E-05
218051_s_at	NT5DC2	1.995583212	5.181657757	0.000106849	0.00199539
202282_at	HSD17B10	1.992482092	9.087585295	0.000178743	0.00293697
207625_s_at	CBFA2T2	1.990993718	5.993313376	0.000178157	0.002930534
203980_at	FABP4	-1.990989917	5.783226463	0.007465977	0.041317664
222263_at	SLC35E1	1.988330405	5.407621749	0.000364156	0.004889636
200675_at	CD81	1.988073317	8.485159931	6.02E-06	0.000228354
201514_s_at	G3BP1	1.987556247	5.521682387	3.51E-05	0.000850897
221538_s_at	PLXNA1	1.987006141	5.065519656	0.0009579	0.009726029

212694_s_at	PCCB	1.986126095	7.372956544	0.002227972	0.017753878
74694_s_at	RABEP2	1.982960237	5.86177506	1.32E-05	0.000422019
204788_s_at	PPOX	-1.980871401	6.430756272	4.76E-09	8.37E-07
218704_at	RNF43	1.97936654	6.011978055	0.000422157	0.005404493
210460_s_at	PSMD4	1.978109405	6.939231107	0.003338328	0.023521713
203856_at	VRK1	-1.977855013	5.650248631	0.001974111	0.016328125
217221_x_at	RBM10	1.977473	6.102987304	0.007497768	0.041425735
202941_at	NDUFV2	1.977367013	7.325258384	0.000342615	0.004683531
213156_at	ZBTB20	1.974394665	7.439183509	4.84E-05	0.001090712
201903_at	UQCRC1	1.974324775	7.343368224	0.007782624	0.042498842
212638_s_at	WWP1	1.973937936	7.770874387	1.85E-05	0.000535432
219312_s_at	ZBTB10	1.973657678	4.97474854	0.0002994	0.004297819
201015_s_at	JUP	1.971295663	4.807077304	0.00057175	0.006794131
204934_s_at	HPN	1.971039802	5.571518285	0.000137335	0.002419644
204249_s_at	LMO2	-1.970544056	6.804023288	1.60E-05	0.00048308
212685_s_at	TBL2	1.969922217	7.35766678	1.81E-06	8.70E-05
208335_s_at	ACKR1	-1.969770189	3.947508166	7.72E-07	4.37E-05
205172_x_at	CLTB	1.968154553	5.61687197	0.000144386	0.00251959
217926_at	C19orf53	1.966975196	7.259540618	0.00251832	0.019367128
201119_s_at	COX8A	1.965998454	9.428529683	0.000685868	0.00764827
212599_at	AUTS2	1.96579698	6.78323592	8.77E-05	0.001696952
202790_at	CLDN7	1.962968879	5.011180022	0.001363733	0.012595932
209529_at	PPAP2C	1.962158742	4.757215728	0.001438772	0.013071166
204238_s_at	DNPH1	1.962158614	5.000454254	0.009879066	0.049959373
200093_s_at	HINT1	1.959853228	8.599109606	0.000271933	0.004007348
207571_x_at	THEMIS2	-1.95616024	5.204573862	1.86E-05	0.000536219
212434_at	GRPEL1	1.955991504	6.987575123	0.000332599	0.004601031
200638_s_at	YWHAZ	1.9555938	7.867819311	0.000607471	0.007058009
213194_at	ROBO1	1.954240841	5.327696034	0.003362342	0.023650196
205627_at	CDA	-1.952929317	3.818199637	1.13E-06	5.95E-05
217140_s_at	VDAC1	1.95260298	6.170501851	0.000164487	0.002787897
208018_s_at	HCK	-1.952508562	5.254249865	8.79E-06	0.000305806
201795_at	LBR	-1.952274119	8.717958242	0.003458352	0.024176739
215034_s_at	TM4SF1	1.950484101	6.090857036	0.006858292	0.03896379
221496_s_at	TOB2	1.94991594	4.731176801	0.000716077	0.007903204
221958_s_at	WLS	1.948263649	5.410417925	0.001622544	0.014245361
205212_s_at	ACAP1	-1.94793619	3.668151659	1.70E-06	8.35E-05
220307_at	CD244	-1.946671849	4.466346063	8.80E-06	0.000305806
218009_s_at	PRC1	-1.943918433	7.22076821	6.92E-05	0.001442102
219275_at	PDCD5	1.943763738	4.761509806	7.63E-05	0.001535586
203523_at	LSP1	-1.942723393	4.189340354	0.00015693	0.002674687
209194_at	CETN2	1.942480573	7.490933637	0.000875246	0.009136126
220145_at	MAP9	1.941298066	4.673455145	0.001213127	0.011611539
212816_s_at	CBS	1.939059991	5.525162348	1.62E-05	0.000487145

200903_s_at	AHCY	1.938250287	7.138469605	0.000121914	0.002205307
208649_s_at	VCP	1.938084072	7.849358449	0.000149737	0.002593179
202806_at	DBN1	1.937594986	5.227313399	0.000552994	0.006622895
201659_s_at	ARL1	1.935303741	6.994460983	2.62E-05	0.000687757
215714_s_at	SMARCA4	1.934416155	6.085879317	0.002446927	0.018989513
41858_at	PGAP2	1.932992632	6.213618444	1.94E-05	0.000547514
213241_at	PLXNC1	-1.930952926	5.72286026	1.88E-05	0.00053998
201713_s_at	RANBP2	1.930741503	7.97527553	0.001238533	0.011784751
219083_at	SHQ1	1.930009868	6.22848563	0.000660916	0.00744263
205640_at	ALDH3B1	-1.928756855	4.029652003	1.23E-05	0.000400327
200699_at	KDELR2	1.927262649	8.675932266	4.63E-06	0.000185432
207543_s_at	P4HA1	1.927034572	7.523304934	0.000171886	0.00288126
202839_s_at	NDUFB7	1.923405112	5.364471959	0.000605228	0.007036497
207992_s_at	AMPD3	-1.922635309	6.711353367	1.84E-06	8.83E-05
213237_at	KNOP1	1.921809312	5.754092161	4.57E-05	0.001042317
203236_s_at	LGALS9	-1.920534275	3.94635232	2.10E-06	9.79E-05
218893_at	ISOC2	1.918539686	5.108580466	2.49E-05	0.00066097
206445_s_at	PRMT1	1.918194329	5.529728219	0.004263713	0.02813852
205141_at	ANG	1.917002903	4.790435453	0.001837113	0.015518301
201186_at	LRPAP1	1.916378563	7.830244279	0.000560354	0.006692135
222138_s_at	WDR13	1.915406974	6.384027088	0.004120344	0.027480983
203402_at	KCNAB2	-1.914300993	4.832275792	2.62E-05	0.000688231
209791_at	PADI2	-1.913562304	4.102567661	1.70E-06	8.35E-05
202559_x_at	CHTOP	1.912846984	7.054272161	6.66E-05	0.001393245
204808_s_at	TMEM5	1.912254666	7.662897622	1.67E-06	8.25E-05
204822_at	TTK	-1.911628783	5.357479294	0.000201747	0.003205509
201242_s_at	ATP1B1	1.911125358	6.442440188	0.008159753	0.043939184
217789_at	SNX6	1.909027812	6.506810966	0.003086305	0.022212654
201904_s_at	CTDSPL	1.907175165	7.253504677	0.002351912	0.018483786
203865_s_at	ADARB1	1.905356576	6.342223561	4.73E-05	0.001073037
201407_s_at	PPP1CB	1.905048489	7.07838958	0.00017299	0.002886644
218200_s_at	NDUFB2	1.904570861	9.833308238	2.63E-05	0.000690101
219630_at	PDZK1IP1	-1.904507692	4.791378581	0.003714178	0.025468665
214004_s_at	VGLL4	1.901482214	6.232391857	1.13E-07	9.54E-06
210830_s_at	PON2	1.899181757	6.25508971	0.000261813	0.003899056
213674_x_at	IGHD	-1.898172524	4.381192418	0.001461517	0.013205647
211501_s_at	EIF3B	1.896157686	7.142356863	0.000236639	0.003609382
204198_s_at	RUNX3	-1.893060171	4.206049739	8.64E-07	4.79E-05
204008_at	DNAL4	1.891694994	5.120860983	0.000252145	0.003801503
218847_at	IGF2BP2	-1.889486315	5.479774485	0.00033765	0.004646313
201341_at	ENC1	1.888741434	6.792368194	0.000120511	0.00218642
201754_at	COX6C	1.88739398	9.48071691	0.000583935	0.006889039
213315_x_at	CXorf40A	1.884500923	6.777217156	0.001322628	0.012330748
213351_s_at	TMCC1	1.8829375	5.711572307	0.000400312	0.005212918

205450_at	PHKA1	1.88230672	5.400766467	0.000721173	0.00794507
202454_s_at	ERBB3	1.882298915	6.902793918	2.93E-05	0.000750123
202322_s_at	GGPS1	1.882257806	7.512742507	6.31E-05	0.001343947
212281_s_at	TMEM97	1.880000545	7.093315874	0.00017936	0.002944473
208980_s_at	UBC	1.87906205	9.679159643	0.001698944	0.014713361
208146_s_at	CPVL	-1.877824105	5.438111125	0.008163345	0.043939184
209934_s_at	ATP2C1	1.875501199	6.908672733	0.000968038	0.009791744
206662_at	GLRX	-1.875295292	7.922803972	0.001198234	0.011497012
219848_s_at	ZNF432	1.87174457	7.175281223	0.001154433	0.011202531
218254_s_at	SAR1B	1.871039857	7.622869591	0.000730357	0.008016223
217809_at	BZW2	1.870548872	7.928590153	7.58E-06	0.00027457
221739_at	C19orf10	1.870378584	7.817463671	0.000133794	0.002368666
219013_at	GALNT11	1.869769373	8.066130336	5.75E-06	0.000221028
221666_s_at	PYCARD	-1.869631677	4.233336079	0.000151127	0.002608925
213400_s_at	TBL1X	1.86864555	6.959473373	5.15E-05	0.001143998
202071_at	SDC4	1.866955085	7.52331474	0.000251827	0.003801421
200866_s_at	PSAP	1.866230901	6.852109266	0.004079552	0.027358578
217491_x_at	COX7C	1.863786605	9.143591002	0.000329023	0.004563779
200024_at	RPS5	1.861533773	10.50319669	6.62E-05	0.001387488
206978_at	CCR2	-1.859568414	5.460813916	7.96E-06	0.000284768
213415_at	CLIC2	-1.858291002	3.811050696	0.000131659	0.0023422
218229_s_at	POGK	1.858219269	6.058423764	6.14E-05	0.001320516
220948_s_at	ATP1A1	1.85820369	8.693250516	8.46E-05	0.001665068
212983_at	HRAS	1.857967632	5.443508459	5.76E-05	0.001258004
218693_at	TSPAN15	1.857465721	5.497054747	6.26E-05	0.001338781
212897_at	CDK19	1.856145268	6.674954033	0.001161465	0.011251809
219489_s_at	NXN	1.855326762	6.921012678	1.94E-05	0.000547514
205119_s_at	FPR1	-1.855012799	5.917025902	0.00036048	0.004853526
204129_at	BCL9	1.854678488	5.115663789	0.001168269	0.011286482
221689_s_at	PIGP	1.854027262	8.026774658	2.28E-05	0.000617676
222071_s_at	SLCO4C1	-1.853855958	3.74226541	5.09E-10	1.79E-07
214806_at	BICD1	1.853274401	5.058406333	0.004994926	0.031292653
201940_at	CPD	1.85299404	6.175194914	0.000122575	0.002212886
217747_s_at	RPS9	1.851896316	9.194452052	0.0005292	0.006430568
218706_s_at	GRAMD3	1.851468311	5.059132635	0.000330149	0.004574071
203896_s_at	PLCB4	1.851230124	5.167553801	0.002116193	0.017078969
205554_s_at	DNASE1L3	-1.85083714	5.30445961	5.30E-05	0.001174332
204231_s_at	FAAH	1.85022404	5.476927918	0.00011076	0.002048107
209064_x_at	PAIP1	1.847021151	7.3148753	2.43E-05	0.000648592
206680_at	CD5L	-1.846796945	4.669841984	6.98E-05	0.00144602
212744_at	BBS4	1.845881998	6.139182339	0.000168104	0.00283518
209681_at	SLC19A2	1.845636611	6.963657249	4.23E-05	0.000982236
218872_at	TESC	-1.845149741	5.394060966	0.000631024	0.007244304
209109_s_at	TSPAN6	1.843557881	5.773646849	0.004668691	0.029863945

218174_s_at	TMEM254	1.843531801	5.206042239	0.000310385	0.004379873
203024_s_at	C5orf15	1.84299566	8.076526493	1.24E-05	0.000402528
206554_x_at	SETMAR	1.842983156	5.662224642	5.94E-05	0.001288722
210470_x_at	NONO	1.842702996	7.242944668	0.009053631	0.046984337
204817_at	ESPL1	-1.840801943	5.894055165	9.09E-07	4.98E-05
203040_s_at	HMBS	-1.839969788	7.206685682	0.000654263	0.007386335
202417_at	KEAP1	1.839390438	6.585846637	0.001776256	0.015151203
221581_s_at	LAT2	-1.838029576	5.332494888	0.000900038	0.009299374
219229_at	SLCO3A1	-1.837788603	4.474661629	0.000264545	0.003926949
218212_s_at	MOCS2	1.837660071	5.161716788	0.000131542	0.0023422
205159_at	CSF2RB	-1.834470315	5.373891736	4.56E-07	2.84E-05
200082_s_at	RPS7	1.834075734	9.782742105	0.000320921	0.004483571
201114_x_at	PSMA7	1.83292879	8.389128242	0.003513735	0.024498353
212228_s_at	COQ9	1.832312012	7.404009667	0.000482566	0.00600352
204982_at	GIT2	-1.832120902	3.784730289	0.000381511	0.005052194
201923_at	PRDX4	1.83159618	8.735438185	0.001020629	0.010182907
201324_at	EMP1	1.831121072	6.791446391	0.003935253	0.026654779
201577_at	NME1	1.831026176	8.582059513	0.000412419	0.00530883
203113_s_at	EEF1D	1.83055238	8.280983206	0.000925223	0.009489896
200001_at	CAPNS1	1.830424929	6.613528505	0.00960195	0.049030653
204688_at	SGCE	1.827867593	5.60926527	0.004584531	0.029542599
202888_s_at	ANPEP	-1.826956594	4.397472889	0.000168693	0.002838134
202343_x_at	COX5B	1.826750228	8.502713229	0.002788372	0.02077879
207648_at	DRP2	-1.825559142	4.547946084	0.0005623	0.006704328
209815_at	PTCH1	1.824523363	6.485756284	0.000764953	0.00826312
203083_at	THBS2	1.824516499	7.604667326	0.000248539	0.003762652
221088_s_at	PPP1R9A	1.82389375	6.386962654	2.44E-05	0.000650032
33322_i_at	SFN	1.823752214	9.171833195	0.008711476	0.04578273
201066_at	CYC1	1.822527795	7.860046715	0.002057457	0.016790171
215017_s_at	FNBP1L	1.822319647	7.142651923	0.000403128	0.005239808
220212_s_at	THADA	1.821457692	5.872414404	0.00043417	0.005510138
217867_x_at	BACE2	1.820209067	7.010420304	0.000328776	0.004563779
201128_s_at	ACLY	1.820060214	9.05310319	0.000157699	0.002682782
205607_s_at	SCYL3	1.819606738	6.097669325	2.98E-05	0.000757948
209036_s_at	MDH2	1.819442769	8.453305679	7.03E-06	0.000258093
221286_s_at	MZB1	-1.817654493	4.286088973	0.007419525	0.041130125
201729_s_at	KIAA0100	1.81603099	6.119247435	0.000616585	0.00711875
204168_at	MGST2	1.815264359	8.413661233	2.13E-05	0.000589173
209177_at	NDUFAF3	1.815254575	6.269712337	0.004339856	0.028477675
219497_s_at	BCL11A	-1.814921708	4.824175846	9.05E-05	0.001740679
218465_at	TMEM33	1.814506011	7.129755798	1.15E-05	0.000378581
217286_s_at	NDRG3	1.814314876	7.848821847	0.000528738	0.006429229
218976_at	DNAJC12	1.814082203	5.771879016	0.004723816	0.030121741
218395_at	ACTR6	1.813946838	5.957450682	0.001661816	0.014478938

219307_at	PDSS2	1.813817089	6.244359087	0.00122022	0.011640783
201079_at	SYNGR2	1.812431077	7.686761324	0.000125606	0.002256467
222294_s_at	RAB27A	-1.812050937	5.951979714	4.76E-06	0.000190016
205038_at	IKZF1	-1.811410633	3.863137735	1.07E-05	0.000355293
218931_at	RAB17	1.810946338	6.014525432	3.76E-05	0.000900781
201519_at	TOMM70A	1.81005386	9.075339938	5.61E-05	0.001232091
214435_x_at	RALA	1.809858155	5.988761891	0.000182162	0.002969556
209268_at	VPS45	1.809457212	6.390467681	5.28E-05	0.001170414
207668_x_at	PDIA6	1.808691602	9.030083076	0.000679086	0.007605093
200895_s_at	FKBP4	1.807499609	8.412074441	4.47E-05	0.001024564
208852_s_at	CANX	1.806420163	7.987762757	8.61E-05	0.001681698
202530_at	MAPK14	-1.805900374	6.575988447	0.002067882	0.016860197
211000_s_at	IL6ST	1.804850976	5.075010194	0.000258693	0.003874678
218039_at	NUSAP1	-1.804150438	8.159945824	0.000337443	0.004646313
212202_s_at	TMEM87A	1.803933535	8.152055	0.000277294	0.004060172
203868_s_at	VCAM1	-1.801947476	6.970808162	0.000954925	0.009709247
213787_s_at	EBP	1.801115789	6.196847369	0.005461967	0.033273971
64486_at	CORO1B	1.800028148	6.549461289	5.75E-06	0.000221028
219587_at	TTC12	1.799786909	4.549096856	0.004156736	0.027653107
213462_at	NPAS2	1.798766582	4.993235283	0.002266659	0.017952647
209340_at	UAP1	1.796951432	7.324361737	0.001322731	0.012330748
215280_s_at	PPFIA3	1.79615785	5.487188593	6.56E-05	0.001383141
201491_at	AHSA1	1.795641276	8.4943088	0.000546002	0.006573585
208808_s_at	HMGB2	-1.795407973	9.365874783	2.54E-05	0.000672792
203725_at	GADD45A	-1.794614226	5.166999077	0.000277285	0.004060172
203257_s_at	C11orf49	1.791205972	5.69695539	5.75E-05	0.00125569
204776_at	THBS4	-1.790973073	5.696012227	8.00E-06	0.000285767
220088_at	C5AR1	-1.790140666	4.609633022	0.000266144	0.003944279
211595_s_at	MRPS11	1.790003831	5.751253339	0.002875334	0.021228949
212032_s_at	PTOV1	1.788824831	4.64982955	0.009139022	0.047333454
201925_s_at	CD55	-1.788431297	4.996721173	4.89E-05	0.001097405
206049_at	SELP	-1.788003969	6.169191066	9.28E-06	0.000318945
207389_at	GP1BA	-1.787885927	6.002720803	3.57E-06	0.000151142
213869_x_at	THY1	1.787295606	5.956345115	2.25E-05	0.000611228
203536_s_at	CIAO1	1.785841227	5.364408484	0.008000151	0.043298837
220261_s_at	ZDHHC4	1.785835478	5.022695798	0.001361549	0.012584426
203263_s_at	ARHGEF9	1.785777806	5.378579462	0.008528541	0.045165114
214167_s_at	RPLP0	1.785275475	10.29670981	0.000481223	0.005990881
213010_at	PRKCDBP	1.784499422	4.824377171	0.000360352	0.004853526
207317_s_at	CASQ2	-1.781724256	3.888239567	1.69E-05	0.000497782
202214_s_at	CUL4B	1.781576025	6.217203797	0.007445325	0.041223162
200723_s_at	CAPRIN1	1.78041834	7.711991672	0.006335488	0.036899175
218922_s_at	CERS4	1.78003613	4.531740111	0.000856562	0.009018316
205789_at	CD1D	-1.776906891	3.95121517	6.85E-08	6.70E-06

209454_s_at	TEAD3	1.776474248	4.538506423	6.39E-06	0.000239015
209684_at	RIN2	1.77625097	6.559991368	0.00176394	0.015074266
205681_at	BCL2A1	-1.775962374	3.82816339	3.96E-06	0.000163797
213110_s_at	COL4A5	1.774654272	6.639178948	0.007237165	0.040511349
203284_s_at	HS2ST1	1.773789524	6.519934383	0.00307101	0.02216279
212364_at	MYO1B	1.772432804	6.192640853	2.57E-06	0.000117078
220688_s_at	MRTO4	1.77241072	6.066532362	0.000949897	0.009667064
201222_s_at	RAD23B	1.772295894	7.121192181	0.000174056	0.002893752
218135_at	ERGIC2	1.771613358	7.624042346	7.37E-05	0.001497623
212765_at	CAMSAP2	1.770923962	6.679370894	3.91E-06	0.000162811
218361_at	GOLPH3L	1.770764668	5.942399457	0.002486193	0.019161543
211396_at	FCGR2C	-1.767921002	3.587450933	8.50E-09	1.35E-06
204558_at	RAD54L	-1.767323372	4.397830686	2.68E-05	0.000699814
217043_s_at	MFN1	1.766347153	6.219555732	0.00056236	0.006704328
220570_at	RETN	-1.764963926	4.475840118	0.001737239	0.014929808
203342_at	TIMM17B	1.764586167	6.119896594	0.000240878	0.003655741
209606_at	CYTIP	-1.764470349	4.679097942	3.94E-06	0.000163595
202475_at	TMEM147	1.764348983	8.018116581	2.34E-05	0.000629377
201093_x_at	SDHA	1.764204505	7.679814141	0.000581164	0.006870281
200936_at	RPL8	1.763489915	10.75901264	0.000630322	0.007240789
213687_s_at	RPL35A	1.762921649	10.39752221	1.44E-05	0.000450058
201767_s_at	ELAC2	1.76253722	5.537411467	0.009676114	0.049264137
212684_at	ZNF3	1.760968631	6.202504837	7.75E-05	0.001555001
208112_x_at	EHD1	-1.760889838	4.957089265	7.24E-05	0.001478742
221610_s_at	STAP2	1.75957689	6.605629139	3.68E-07	2.39E-05
218140_x_at	SRPRB	1.759123834	8.183826457	1.73E-06	8.46E-05
212151_at	PBX1	1.758961232	6.226623781	0.000133494	0.00236793
212508_at	MOAP1	1.758567509	7.423390067	0.000305932	0.004357388
218451_at	CDCP1	1.758009764	5.474699675	0.003308806	0.023372515
204826_at	CCNF	-1.756755448	4.712507935	0.000213978	0.003364766
200644_at	MARCKSL1	1.756474597	7.706632461	7.73E-05	0.001551169
202939_at	ZMPSTE24	1.754582815	8.185733435	3.11E-05	0.000784157
221561_at	SOAT1	1.754117289	5.609644397	0.001401547	0.012828577
204005_s_at	PAWR	1.751831795	5.161885794	4.22E-05	0.000982236
214831_at	ELK4	1.751539685	4.50669778	0.000589244	0.006907751
201653_at	CNIH1	1.749948823	7.611037732	0.000493675	0.00610778
203538_at	CAMLG	1.749337558	8.091483278	3.90E-06	0.000162811
219045_at	RHOF	-1.748612653	4.660787636	0.000197559	0.003152284
214438_at	HLX	-1.747211561	5.990172881	2.07E-06	9.72E-05
202946_s_at	BTBD3	1.746840899	5.964022914	0.000575733	0.006831521
218190_s_at	UQCR10	1.746731345	8.646705896	0.001109669	0.010863828
204046_at	PLCB2	-1.74646915	5.202400049	0.001320816	0.012330312
208680_at	PRDX1	1.746154435	9.566305491	0.000372939	0.004975596
211594_s_at	MRPL9	1.745307443	5.485051697	0.007986147	0.043235836

208517_x_at	BTF3	1.743516722	10.01416926	0.00030822	0.004369014
201646_at	SCARB2	1.743144029	6.828660193	1.78E-07	1.32E-05
200793_s_at	ACO2	1.742373115	6.567066926	0.000405578	0.005245533
202743_at	PIK3R3	1.74212498	6.530604334	0.000357066	0.004826333
218624_s_at	CENPBD1P1	1.741452426	6.193656115	2.88E-06	0.000126741
216806_at	RP3-334F4.1	1.739354294	5.995282962	0.000113127	0.002081354
201541_s_at	ZNHIT1	1.739276999	5.491358134	0.00569032	0.034231766
205907_s_at	OMD	1.737775351	6.290739115	0.004398737	0.028706123
204162_at	NDC80	-1.737643694	5.10455466	0.002353768	0.018490422
209368_at	EPHX2	1.737267098	4.809228776	0.005146673	0.031942934
207711_at	SOGA1	1.736549988	5.542119221	2.56E-05	0.000676803
204384_at	GOLGA2	1.735501986	5.355323599	0.001309531	0.012287685
212538_at	DOCK9	1.734690541	5.568581923	0.000390472	0.005126314
204278_s_at	EBAG9	1.734543599	5.958962665	2.75E-06	0.000123749
202210_x_at	GSK3A	-1.734418639	5.523172558	0.000453547	0.005700666
219372_at	IFT81	1.733042259	4.957788649	4.56E-06	0.00018352
58696_at	EXOSC4	1.731659079	6.102652519	0.000360023	0.004853526
217744_s_at	PERP	1.731521299	5.923244849	7.32E-05	0.001491434
218001_at	MRPS2	1.731264042	5.013331645	0.000274725	0.00403872
200885_at	RHOC	1.731203899	6.745846598	0.001532122	0.01364141
214787_at	DENND4A	-1.729891166	4.111905739	1.02E-06	5.47E-05
218601_at	URGCP	1.729532889	6.34811651	0.000156589	0.002671399
205066_s_at	ENPP1	1.728732719	5.791231486	0.000874911	0.009136126
203964_at	NMI	-1.727283059	6.217901473	0.00155282	0.013769286
212775_at	OBSL1	1.725834892	4.916075977	0.00611755	0.036010974
207252_at	INE1	-1.72496126	4.695551638	8.82E-05	0.001703104
37145_at	GNLY	-1.724341406	3.990857695	1.53E-05	0.000469855
208716_s_at	TMCO1	1.724089138	8.695673312	0.000123139	0.002220875
205483_s_at	ISG15	1.723939788	5.763657823	0.005613186	0.033946409
203502_at	BPGM	-1.722178848	5.99591033	3.37E-07	2.24E-05
201983_s_at	EGFR	1.722144235	5.489009745	0.000386073	0.005092843
209692_at	EYA2	1.72191048	5.578449675	0.000326984	0.004548082
211759_x_at	TBCB	1.720678419	7.706968126	0.008814508	0.046141768
206247_at	MICB	-1.718971146	4.540514656	6.26E-05	0.001338781
205403_at	IL1R2	-1.718745791	4.048901002	0.0015418	0.013707551
208056_s_at	CBFA2T3	-1.717253067	4.965022584	1.87E-05	0.000537998
218592_s_at	CECR5	1.717082547	7.560998165	0.000521314	0.006372849
204247_s_at	CDK5	1.716199295	4.656955344	0.009620675	0.049091214
212952_at	CTC-425F1.4	1.716196804	6.439829234	0.000586021	0.006892921
212886_at	CCDC69	-1.715370219	3.643831605	9.51E-10	2.76E-07
204834_at	FGL2	-1.714730018	5.138252984	7.42E-05	0.001505846
205246_at	PEX13	1.714674433	4.649533285	0.000569096	0.006771389
201667_at	GJA1	1.713636797	7.313168798	0.009595327	0.049030653
217839_at	TFG	1.713391952	8.192010457	6.17E-06	0.000232567

216350_s_at	ZNF10	1.713261702	4.985570876	0.00147528	0.013297148
202986_at	ARNT2	1.711749496	4.491977276	0.003629353	0.025065559
200693_at	YWHAQ	1.71094732	9.611010818	0.000590272	0.006913021
206565_x_at	SMA4	1.710905687	4.847258261	0.000342978	0.004684375
212954_at	DYRK4	1.710292868	6.215002208	0.007939476	0.043034123
202431_s_at	MYC	1.709456208	6.54627657	0.007677315	0.042170534
218160_at	NDUFA8	1.708888438	7.892496181	0.000972928	0.009824906
201063_at	RCN1	1.708469991	7.858985798	0.000455546	0.005721853
207666_x_at	SSX3	1.708036065	4.779281799	0.003618265	0.025007869
212529_at	LSM12	1.707644681	4.746754589	0.000182632	0.002974158
209127_s_at	SART3	1.705671277	6.643549416	0.000309551	0.004378249
201938_at	CDK2AP1	1.702370447	8.833202646	5.15E-05	0.001143998
203508_at	TNFRSF1B	-1.701836574	4.642660097	0.000508812	0.006249259
218446_s_at	TVP23B	1.700888616	5.795098867	0.001315994	0.01231043
206417_at	CNGA1	1.700677013	4.806454584	0.000299779	0.004299893
204949_at	ICAM3	-1.698919867	5.807257879	0.00238446	0.018627515
201231_s_at	ENO1	1.698305901	9.661093497	0.002365333	0.018546562
202664_at	WIPF1	-1.697590024	5.512887485	0.000168314	0.00283518
211048_s_at	PDIA4	1.696078788	7.936828388	3.40E-05	0.000828094
208905_at	CYCS	1.695715177	9.805219378	0.000422891	0.005404493
202715_at	CAD	1.695167441	5.841445946	0.000378313	0.005031706
220404_at	GPR97	-1.695161713	5.023438097	2.89E-07	1.99E-05
201700_at	CCND3	-1.694953378	6.779016834	8.62E-08	7.88E-06
201745_at	TWF1	1.694745794	7.616803664	0.000127523	0.002284179
203922_s_at	CYBB	-1.694136655	5.354607086	1.20E-07	9.88E-06
219799_s_at	DHRS9	-1.69237494	5.382261537	0.000870842	0.009121393
208669_s_at	EID1	1.690612777	7.421197395	0.006206786	0.036323103
217791_s_at	ALDH18A1	1.690216302	6.737626525	0.001395916	0.012802661
200804_at	TMBIM6	1.690025369	9.624718541	0.000587517	0.006900712
222061_at	CD58	-1.689732581	4.062471088	4.90E-05	0.001098995
212921_at	SMYD2	1.689005293	4.993482232	0.000289859	0.004206684
222212_s_at	CERS2	1.688312519	7.326342559	0.000617443	0.007124164
205120_s_at	SGCB	1.687899644	6.409009171	8.80E-05	0.001701404
217942_at	MRPS35	1.687426143	8.224055831	1.64E-06	8.15E-05
204288_s_at	SORBS2	1.685493948	5.823331625	0.003070277	0.02216279
210594_x_at	MPZL1	1.682910335	5.529324327	0.000223419	0.003473018
204444_at	KIF11	-1.682767025	5.105098151	0.000268132	0.003964105
209432_s_at	CREB3	1.682501115	6.720235416	0.002970506	0.021656662
219505_at	CECR1	-1.682240802	4.10356988	0.001348244	0.012490722
217968_at	TSSC1	1.681951061	6.293776109	0.001163923	0.011256383
203465_at	MRPL19	1.681926061	5.134455779	0.002997916	0.021796976
202887_s_at	DDIT4	1.681760946	7.695658576	0.006144789	0.036098906
202957_at	HCLS1	-1.681759298	6.874252983	0.00040242	0.005234319
201397_at	PHGDH	1.68163602	7.076150123	4.52E-05	0.001032966

201637_s_at	FXR1	1.681358345	7.683058051	0.00426579	0.02813852
200747_s_at	NUMA1	1.680246614	6.938070379	0.000152424	0.002627267
210446_at	GATA1	-1.678472264	4.821644983	4.46E-05	0.001024217
217940_s_at	CARKD	1.677879431	8.125686301	4.05E-05	0.00095157
217802_s_at	NUCKS1	1.677814466	8.955443577	0.000134563	0.002377674
201599_at	OAT	-1.677724152	7.22537003	0.005567984	0.033728815
207375_s_at	IL15RA	-1.677354776	4.36502222	0.000282107	0.00412185
218133_s_at	NIF3L1	1.676675097	6.889839737	0.001682465	0.014603187
209435_s_at	ARHGEF2	-1.675813091	6.812657373	8.69E-06	0.000304462
201780_s_at	RNF13	1.675640994	6.317092931	0.002187205	0.01751296
213085_s_at	WWC1	1.67383201	6.982112701	1.07E-07	9.10E-06
219718_at	FGGY	1.673629912	5.146850249	0.00018716	0.003031687
219306_at	KIF15	-1.673479786	5.785940253	0.000381138	0.005050904
53987_at	RANBP10	-1.673095063	6.225594684	3.88E-09	7.32E-07
212740_at	PIK3R4	1.672127193	6.508541613	0.001930528	0.016084335
213246_at	TMEM251	1.67176717	6.457009659	0.003059128	0.022104046
201663_s_at	SMC4	-1.6703269	6.807438366	0.000194668	0.003118091
203035_s_at	PIAS3	1.670207469	5.859184601	6.03E-05	0.001302791
213763_at	HIPK2	1.667340126	6.160188254	0.001746259	0.014993236
209017_s_at	LONP1	1.666456468	6.187090432	0.000226614	0.003503227
205899_at	CCNA1	-1.665643081	4.166450801	1.01E-05	0.000336656
212106_at	FAF2	1.665232878	5.157847141	0.001621757	0.014245361
212428_at	KIAA0368	1.664388815	6.864785895	0.001468038	0.01324493
213532_at	ADAM17	1.664215657	5.653108615	4.57E-06	0.00018352
204924_at	TLR2	-1.663833117	4.629132574	0.004180257	0.0277521
206643_at	HAL	-1.662767364	4.37722629	2.72E-05	0.000707177
217770_at	PIGT	1.662344947	6.120873208	0.000918584	0.009442983
205672_at	XPA	1.661678419	6.562097776	0.001074528	0.010622142
204222_s_at	GLIPR1	-1.659862746	4.713041711	0.005682277	0.034228419
215963_x_at	RPL3	1.659320974	11.92117608	7.47E-05	0.001511213
209933_s_at	CD300A	-1.659258367	5.185792842	0.000308421	0.004369014
203781_at	MRPL33	1.65908727	8.246192114	0.000640511	0.007307344
209897_s_at	SLIT2	1.658338325	6.652333656	0.001245432	0.011825786
202115_s_at	NOC2L	1.657974413	4.505227882	0.001979186	0.016348397
217227_x_at	IGLV1-44	-1.657705219	4.991593744	1.68E-05	0.000494963
202143_s_at	COPS8	1.657345299	5.890443954	0.004226259	0.027983282
209967_s_at	CREM	1.655580069	4.49438369	0.000204819	0.003240254
219127_at	PRR15L	1.65516983	6.605578026	5.72E-05	0.001252118
208594_x_at	LILRA6	-1.654564285	4.763442572	6.27E-05	0.001338949
206666_at	GZMK	-1.654211351	4.572113352	0.000621693	0.007159645
218151_x_at	SLC52A2	1.653512853	5.974994032	0.00185058	0.01561043
200993_at	IPO7	1.653142185	7.453755453	7.85E-05	0.00156716
201888_s_at	IL13RA1	1.652472651	4.697031235	0.000219865	0.003427561
32502_at	GDPD5	-1.652429441	4.539117345	0.000266921	0.003949535

208923_at	CYFIP1	1.650133226	8.741645321	4.09E-05	0.000958472
209311_at	BCL2L2	1.64982529	6.734538746	9.08E-05	0.001742963
212667_at	SPARC	1.649781936	8.338313207	0.009358275	0.048093088
213132_s_at	MCAT	1.648075163	5.937836483	9.06E-05	0.001740679
210613_s_at	SYNGR1	-1.646497294	5.191639295	0.001788368	0.015233196
214627_at	EPX	-1.645350295	5.653159354	8.72E-06	0.000304915
202631_s_at	APPBP2	1.644902537	5.412144295	1.26E-05	0.00040594
218645_at	ZNF277	1.644257551	6.11762712	0.00031679	0.004451017
209902_at	ATR	1.64353268	4.771026691	0.009581506	0.049000724
220432_s_at	CYP39A1	1.642416833	4.784675843	0.00141997	0.012964759
204409_s_at	EIF1AY	-1.642230907	5.100530035	0.005972656	0.035416052
206991_s_at	CCR5	-1.641857197	5.047502956	0.000603512	0.007021008
209879_at	SELPLG	-1.640783203	5.471452534	1.49E-05	0.000459722
209679_s_at	SMAGP	1.640757433	5.279494682	0.000868954	0.009117287
203517_at	MTX2	1.639874784	7.180941617	0.002225028	0.017751233
201201_at	CSTB	1.638356955	8.394915408	0.001175034	0.011327902
221479_s_at	BNIP3L	-1.637970732	7.976456892	2.43E-05	0.000648592
208872_s_at	REEP5	1.636878334	7.884520951	0.004287268	0.028231857
218767_at	REXO4	1.636810992	5.660588826	0.000898756	0.009296264
218919_at	ZFAND1	1.636255027	7.029250542	0.000140787	0.002468572
204436_at	PLEKHO2	-1.635750838	4.722806874	0.00242752	0.018891268
218962_s_at	TMEM168	1.635647038	7.672491335	0.000392273	0.00513521
202976_s_at	RHOBTB3	1.63544189	6.872304861	0.001870981	0.015717274
212519_at	UBE2E1	1.635187036	8.54121857	0.00043268	0.005498853
212242_at	TUBA4A	-1.635066578	6.254971455	2.18E-05	0.000597735
212553_at	RPRD2	1.634573044	5.479133567	0.001410412	0.012903257
218788_s_at	SMYD3	1.634165893	6.109243152	0.000799093	0.00856111
214909_s_at	DDAH2	1.633140275	5.018346873	0.005329639	0.032685592
217728_at	S100A6	-1.633109359	6.085199143	0.000786561	0.008451605
208837_at	TMED3	1.632805629	7.757542367	0.000126296	0.002264421
207180_s_at	HTATIP2	1.631137413	5.526426844	0.000139914	0.002459565
205707_at	IL17RA	-1.631069876	6.001080571	3.09E-07	2.10E-05
222039_at	KIF18B	-1.630737844	6.052630432	0.000597127	0.006961515
221084_at	HTR3B	-1.630134485	4.168795802	1.06E-05	0.000352503
204518_s_at	PPIC	1.629976782	5.456228037	0.00074253	0.008107101
213503_x_at	ANXA2	1.628891895	9.732390567	0.004703689	0.030022323
202910_s_at	CD97	-1.628851279	4.893290445	0.001032999	0.010284538
219999_at	MAN2A2	-1.625831242	5.582911428	2.09E-06	9.78E-05
200036_s_at	RPL10A	1.625526892	10.0157373	0.000104251	0.001955424
214271_x_at	RPL12	1.625513782	10.90732174	0.000350396	0.004760806
204311_at	ATP1B2	-1.62459002	4.598276094	0.000819065	0.008673457
204021_s_at	PURA	1.624304949	6.238349874	6.74E-06	0.000250702
206555_s_at	THUMPD1	1.622555564	8.137963444	0.000259115	0.003877826
204866_at	JADE3	1.621487264	5.459465882	0.000383685	0.005073634

219594_at	NINJ2	-1.621150403	3.983452533	0.001563741	0.013842056
205308_at	ZC2HC1A	1.620433871	6.770170926	9.34E-06	0.000319271
213491_x_at	RPN2	1.620165479	9.948349943	0.00014565	0.0025344
35626_at	SGSH	1.619610462	6.592740407	0.000231541	0.003554244
204689_at	HHEX	-1.619321079	5.080555834	1.56E-06	7.79E-05
204298_s_at	LOX	1.618956909	4.518498817	0.001101206	0.010821527
33304_at	ISG20	-1.618947765	5.578172783	0.000937729	0.009591266
203697_at	FRZB	-1.618862369	4.874736318	2.76E-05	0.000714639
202320_at	GTF3C1	1.618510419	4.509366969	0.009642217	0.04918741
200790_at	ODC1	1.618229698	9.373703703	0.007082062	0.039949645
201518_at	CBX1	1.617607044	8.383629889	9.76E-06	0.000329518
201304_at	NDUFA5	1.617517353	7.543751987	0.000706877	0.007834773
219544_at	BORA	-1.617292129	5.358891882	3.09E-07	2.10E-05
204137_at	GPR137B	1.616226947	5.674457059	0.000614654	0.007105512
201976_s_at	MYO10	1.615899273	7.743738781	3.82E-05	0.000907876
219053_s_at	VPS37C	1.615193971	7.405726222	4.21E-05	0.000980004
221434_s_at	SLIRP	1.614595377	7.806153709	0.004149973	0.027628218
218209_s_at	RPRD1A	1.614343746	6.583981855	0.000263474	0.003917415
210973_s_at	FGFR1	-1.61402898	4.960003848	0.000609413	0.007067183
208911_s_at	PDHB	1.613781663	6.42654245	0.008012132	0.043325213
215084_s_at	LRRC42	1.61365588	5.013728037	2.36E-05	0.000633565
202554_s_at	GSTM3	1.613032327	6.143054651	0.001306804	0.012281005
209279_s_at	NSDHL	1.612917394	5.244738201	0.002440205	0.018957715
220341_s_at	C5orf45	1.612915517	4.876931616	0.004319121	0.028395584
204540_at	EEF1A2	1.611655035	4.383768845	0.003081309	0.022194161
203202_at	KRR1	1.611596899	6.484693618	0.000917969	0.009441966
206380_s_at	CFP	-1.610962417	3.462056846	4.67E-05	0.00106179
221868_at	PAIP2B	1.610284372	6.124278134	0.000135148	0.0023834
201523_x_at	UBE2N	1.610132473	6.283040502	0.00224824	0.017860906
204307_at	TECPR2	-1.609534601	4.395447544	0.00146604	0.013233437
213518_at	PRKCI	1.608657532	6.351956569	5.84E-06	0.000223765
201846_s_at	RYBP	1.608152766	6.458622187	8.29E-05	0.001635819
201195_s_at	SLC7A5	-1.608063075	5.835632076	0.000943121	0.009624883
205715_at	BST1	-1.607520796	3.467515287	6.45E-05	0.001367547
214617_at	PRF1	-1.60605304	5.020790158	3.91E-06	0.000162811
202170_s_at	AASDHPPT	1.605166893	5.836791574	0.000182187	0.002969556
217927_at	SPCS1	1.604621477	8.554306394	0.000808751	0.008594096
215147_at	AF007147	-1.603747185	4.609867245	0.000199905	0.003181781
221840_at	PTPRE	-1.602248025	5.996008885	1.09E-05	0.000360555
202541_at	AIMP1	1.60214186	6.053158235	0.000491026	0.00608393
203764_at	DLGAP5	-1.600971747	5.999710515	0.001735263	0.014919839
202472_at	MPI	1.600025875	4.72997273	0.005093864	0.031718277
218219_s_at	LANCL2	1.599581833	6.389758624	0.000192626	0.003094806
201260_s_at	SYPL1	1.599447519	7.741540413	0.001980142	0.016348913

211798_x_at	IGLJ3	-1.599421113	4.487053314	0.000614661	0.007105512
220528_at	VNN3	-1.599349985	4.202760137	1.23E-05	0.000399429
218358_at	CRELD2	1.599300953	8.263914374	0.00098705	0.009923677
207104_x_at	LILRB1	-1.598489441	3.532208181	6.39E-06	0.000239015
210996_s_at	YWHAE	1.598310548	7.164218243	0.001707117	0.014754137
206632_s_at	APOBEC3B	-1.597870559	5.355564971	0.000215667	0.003382934
210544_s_at	ALDH3A2	1.597862378	5.739774686	0.000404704	0.005241667
221589_s_at	ALDH6A1	1.596855777	6.553530287	0.000499487	0.006167869
212614_at	ARID5B	1.596543092	8.101117564	8.86E-06	0.000307628
218597_s_at	CISD1	1.595427464	6.664169235	0.000212243	0.00334036
200652_at	SSR2	1.595302424	8.9920527	0.000340399	0.004673367
203584_at	EMC2	1.594619511	7.058796898	0.000751445	0.008184891
205420_at	PEX7	1.593550691	4.972874343	0.003716827	0.025477258
215143_at	DPY19L2P2	-1.593012072	4.448249294	5.00E-05	0.001116875
212341_at	YIPF6	1.591547975	8.787476377	0.003571467	0.024787469
211936_at	HSPA5	1.591338456	9.029695027	8.48E-05	0.001666954
218476_at	POMT1	1.590059383	5.500756539	3.23E-05	0.000805831
219833_s_at	EFHC1	1.58985627	5.300975095	8.77E-05	0.001696952
208675_s_at	DDOST	1.589709119	7.444251313	0.004089477	0.027374948
208838_at	CAND1	1.589665922	6.538559061	0.001750473	0.015008271
210112_at	HPS1	-1.588988059	4.607118227	0.000399877	0.00521237
219579_at	RAB3IL1	-1.588064301	4.419835565	4.52E-07	2.83E-05
214375_at	PPFIBP1	1.588006605	5.451800143	0.001717727	0.014817823
208095_s_at	SRP72	1.587277075	8.307071928	2.71E-05	0.000705297
215449_at	TSPO2	-1.587162555	3.63294881	0.000731138	0.008016223
209065_at	UQCRB	1.58665729	6.161770715	2.98E-05	0.000757948
59697_at	RAB15	1.586580798	6.336745577	3.50E-06	0.000148866
208741_at	SAP18	1.585875573	5.234005791	0.001617753	0.014230625
205639_at	AOAH	-1.585233859	3.657671666	4.58E-05	0.001042728
200749_at	RAN	1.584918363	7.339065567	0.00150469	0.013475889
203014_x_at	SGSM3	1.584726571	6.021018722	0.000490937	0.00608393
209760_at	KIAA0922	-1.584614704	6.520685558	0.000324086	0.00451744
221020_s_at	SLC25A32	1.583956415	5.534994278	5.51E-05	0.001213832
211986_at	AHNAK	1.583845738	8.439937512	0.000133657	0.002368522
217777_s_at	PTPLAD1	1.583812804	5.579658982	8.54E-05	0.001673147
218991_at	HEATR6	1.58330457	5.342492221	1.96E-05	0.000550059
203305_at	F13A1	-1.583257127	6.899571581	0.000942593	0.009624868
221437_s_at	MRPS15	1.582438382	5.431541319	0.003449986	0.024136705
202708_s_at	HIST2H2BE	1.582185223	7.932592673	0.002635196	0.020012227
209710_at	GATA2	1.580787157	8.043059795	0.000639655	0.007302129
204891_s_at	LCK	-1.580321459	4.29199356	0.000410742	0.005297352
219278_at	MAP3K6	1.579731103	4.355714481	0.001647212	0.014382931
201975_at	CLIP1	1.579276987	6.184959097	0.003924649	0.026592808
213429_at	BICC1	1.5787702	5.3887947	0.000167532	0.002833583

201939_at	PLK2	1.578011552	4.699821717	0.0005844	0.006889039
202803_s_at	ITGB2	-1.577721285	5.652326584	0.000184826	0.003001868
208796_s_at	CCNG1	1.576969683	8.219772363	0.001038807	0.010336077
203005_at	LTBR	1.576524608	5.105827126	5.86E-06	0.000224194
209524_at	HDGFRP3	1.575548542	5.630180826	0.002859747	0.021161873
212361_s_at	ATP2A2	1.575158099	6.163826029	0.001874349	0.015738336
200924_s_at	SLC3A2	1.574717208	5.7752748	0.006175673	0.036222163
212975_at	DENND3	-1.573702855	6.126868269	0.000303199	0.004328567
219572_at	CADPS2	1.573387058	7.088780075	0.000644924	0.007334811
218928_s_at	SLC37A1	1.573359537	4.563118771	0.007782562	0.042498842
202866_at	DNAJB12	1.572137111	6.094237485	0.000888472	0.009226792
213188_s_at	MINA	1.569569607	6.793259929	2.21E-05	0.000603197
213982_s_at	RABGAP1L	-1.569355019	5.858986927	0.002628201	0.019976947
206700_s_at	KDM5D	-1.56917564	7.049742689	0.006550633	0.03769603
201115_at	POLD2	1.568496167	5.598750391	0.002922507	0.021473207
201892_s_at	IMPDH2	1.567027449	8.636153994	0.008042842	0.043427073
218035_s_at	RBM47	1.567019646	8.12986298	0.000404515	0.005241667
211429_s_at	SERPINA1	-1.565432876	6.189282869	0.002463815	0.019076315
214481_at	HIST1H2AM	1.564039084	4.710327528	0.000112138	0.002069411
215554_at	GPLD1	-1.563327988	3.64253026	9.01E-08	8.08E-06
203458_at	SPR	1.562771059	7.051833974	1.86E-06	8.90E-05
212245_at	MCFD2	1.562302927	6.954426028	3.85E-05	0.000914207
211964_at	COL4A2	1.561554788	7.229384198	0.006147857	0.036105331
36030_at	IFFO1	-1.561069928	5.988439221	9.17E-05	0.001755654
203620_s_at	FCHSD2	-1.560942078	3.925726161	0.000587889	0.006900712
222146_s_at	TCF4	1.559308097	5.366480144	0.008944675	0.046670531
203415_at	PDCD6	1.559282546	6.720656407	0.000662329	0.007453948
222116_s_at	TBC1D16	1.559112924	6.481351721	0.000169757	0.0028508
212233_at	MAP1B	1.558335655	6.956871822	0.001319754	0.012330312
205594_at	ZNF652	1.55831614	6.126272631	4.94E-05	0.001105081
207843_x_at	CYB5A	1.557973923	8.287212018	0.001184788	0.011403899
213839_at	CLMN	1.557136802	6.148242677	0.001310203	0.012287685
206464_at	BMX	-1.556658673	4.059288131	0.000523229	0.006387711
211968_s_at	HSP90AA1	1.555031668	9.937957656	0.000142102	0.002484477
201854_s_at	ATMIN	1.553982993	6.467889012	1.67E-05	0.0004946
218326_s_at	LGR4	1.552477467	5.545311611	0.002455731	0.019029835
214011_s_at	NOP16	1.551717397	6.859940308	0.000916485	0.009437318
218188_s_at	TIMM13	1.548985596	5.893357786	0.001107359	0.01084702
202623_at	EAPP	1.548659193	6.792103494	0.000632035	0.007249094
217848_s_at	PPA1	1.548560243	9.186116353	0.000648804	0.007344311
207192_at	DNASE1L2	-1.54794034	3.699366412	8.76E-05	0.001696952
219402_s_at	DERL1	1.547456252	7.284515314	0.00941393	0.048305825
200925_at	COX6A1	1.547300002	8.708467042	0.004338885	0.028477675
201994_at	MORF4L2	1.547088015	10.34953964	2.58E-05	0.000679946

200013_at	RPL24	1.546939431	10.66137261	0.000799815	0.00856379
217752_s_at	CNDP2	1.546472302	8.871541262	0.000290291	0.004206684
204868_at	ICT1	1.545368635	6.89395686	0.000168265	0.00283518
219295_s_at	PCOLCE2	-1.54385136	4.486110949	8.75E-05	0.001696952
211163_s_at	TNFRSF10C	-1.543711113	3.526814579	5.59E-06	0.000215689
217934_x_at	STUB1	1.54312359	7.784174075	6.48E-05	0.0013722
219060_at	WDYHV1	1.54274691	5.497001842	0.00064183	0.007317827
217313_at	AC004692.5	1.542515687	5.576735677	0.005546349	0.033653495
204777_s_at	MAL	-1.540972147	5.197160226	0.000491912	0.006090789
207395_at	BTN1A1	1.539325607	4.770407648	9.56E-06	0.000324877
206478_at	KIAA0125	-1.538879657	4.820616115	1.42E-05	0.000446592
203431_s_at	ARHGAP32	1.537799716	6.653109727	0.000119315	0.002169026
219528_s_at	BCL11B	-1.537798953	4.650551271	3.79E-06	0.000159533
212449_s_at	LYPLA1	1.537700821	8.070089198	0.000368076	0.004931407
209530_at	CACNB3	1.537621751	5.100242284	0.000217335	0.00340197
205220_at	HCAR3	-1.536394971	4.698709075	0.000299029	0.004297819
212523_s_at	SPIDR	1.535631983	5.138187379	0.000850602	0.008965882
222372_at	MAGI1	1.535318865	5.964913065	0.003129327	0.022451604
203416_at	CD53	-1.534776	7.731027547	2.17E-05	0.000595876
212626_x_at	HNRNPC	1.533869166	9.281238576	0.004101353	0.027434363
206187_at	PTGIR	-1.533763868	4.063086873	3.25E-05	0.000805831
208645_s_at	RPS14	1.533244829	11.43849366	0.000118282	0.002154529
212878_s_at	KLC1	1.532440849	4.796837932	0.0056204	0.033954064
203501_at	CPQ	1.531701876	5.66759805	0.000990577	0.009937291
206770_s_at	SLC35A3	1.530879987	6.221006272	0.002472091	0.01909189
206994_at	CST4	1.530695221	4.511496668	0.004765448	0.030281621
218427_at	SDCCAG3	1.530047179	6.399282213	0.000779444	0.008389914
218008_at	TMEM248	1.529998599	8.007059532	0.00056965	0.006773581
214256_at	ATP10A	-1.529385097	4.435168384	0.00017283	0.002886644
211662_s_at	VDAC2	1.529014933	9.647678286	0.001499311	0.01345407
218557_at	NIT2	1.528349905	7.331061341	0.001137077	0.011078777
209511_at	POLR2F	1.52744083	6.47374883	0.001375599	0.012679914
207470_at	BC113958	1.526879549	4.543543116	0.0012884	0.012151754
221063_x_at	RNF123	-1.526742605	5.698892372	0.000110687	0.002048107
208168_s_at	CHIT1	-1.525849163	3.734444378	3.86E-05	0.000916314
202780_at	OXCT1	1.525490622	6.68874511	0.008443514	0.044848965
205441_at	OCEL1	1.525409702	5.736327377	0.000988213	0.009924459
213524_s_at	G0S2	-1.525369866	6.183394498	0.004713335	0.030065391
203227_s_at	TSPAN31	1.524893102	6.829110287	0.000224765	0.003483479
219036_at	CEP70	1.523397537	6.017380899	1.76E-06	8.55E-05
212171_x_at	VEGFA	1.522898982	6.453866497	0.000772573	0.008330669
209014_at	MAGED1	1.522790236	8.939804639	0.002470648	0.019088806
217900_at	IARS2	1.522541095	7.207404897	0.000104607	0.001960104
209585_s_at	MINPP1	-1.522384996	5.967275639	0.003080058	0.022193896

209043_at	PAPSS1	1.521937281	8.502230855	0.000715258	0.007898941
200877_at	CCT4	1.521398485	9.214052396	0.000744739	0.008126362
218226_s_at	NDUFB4	1.520434943	9.28118137	0.00109884	0.01080989
203505_at	ABCA1	1.520307916	6.43897685	0.000437101	0.005533775
31845_at	ELF4	-1.520295038	4.685989398	0.000632386	0.007249094
213193_x_at	TRBC1	-1.518916566	6.143342695	9.04E-06	0.000312623
200046_at	DAD1	1.518415812	8.881246327	0.002071251	0.016880145
201057_s_at	GOLGB1	1.51825576	8.74582735	4.86E-05	0.001092208
200818_at	ATP5O	1.517968866	8.976719901	0.006845992	0.038942301
220937_s_at	ST6GALNAC4	-1.51704601	3.855970264	5.30E-06	0.000206369
209734_at	NCKAP1L	-1.51643323	4.918687896	0.000268546	0.003967026
212544_at	ZNHIT3	1.516010463	6.587831488	0.000333085	0.004604273
219368_at	NAP1L2	1.515436126	6.047738203	0.00562674	0.033960997
211651_s_at	LAMB1	1.514565098	4.707090053	0.008721798	0.045821383
210023_s_at	PCGF1	1.513632109	5.804294654	0.005971857	0.035416052
205913_at	PLIN1	-1.512809566	4.206552979	0.001728003	0.014871394
212300_at	TXLNA	1.511681858	5.603093001	0.004856689	0.030681929
202540_s_at	HMGCR	1.511321523	6.979247112	0.002821058	0.020929915
206121_at	AMPD1	-1.511036594	4.793623765	6.60E-05	0.001386069
201638_s_at	CPSF1	-1.510938315	3.782129451	2.56E-05	0.000676803
202011_at	TJP1	1.51051525	7.945454539	9.15E-05	0.001754893
211764_s_at	UBE2D1	1.510451033	5.933742194	0.004566901	0.029449751
200733_s_at	PTP4A1	1.509771952	5.886319824	0.001296072	0.012198949
203303_at	DYNLT3	1.509645271	8.460384614	8.30E-05	0.001635819
203532_x_at	CUL5	1.509355814	4.351454719	0.006352895	0.036965239
204730_at	RIMS3	-1.509346258	4.717025482	0.000117675	0.002147736
219812_at	PVRIG	-1.50691317	4.272035924	6.99E-05	0.00144602
209336_at	PWP2	1.506295535	5.303916451	0.001915132	0.015992665
202822_at	LPP	1.50573699	8.352493642	1.50E-06	7.50E-05
203095_at	MTIF2	1.505179881	7.857003201	0.000316643	0.004451017
211615_s_at	LRPPRC	1.505043503	8.233583987	0.00043325	0.005502281
218548_x_at	TEX264	1.501234155	5.884893519	0.001510547	0.013515109
204411_at	KIF21B	-1.499028113	3.47118665	6.63E-05	0.001389654
212450_at	SECISBP2L	1.498554524	6.830611319	0.000344179	0.004689043
201707_at	PEX19	1.497961381	5.330410846	0.004744146	0.030156741
212653_s_at	EHBP1	1.497817523	7.715234578	0.002794938	0.020803221
203939_at	NT5E	1.497619838	4.51850259	0.001959549	0.01625226
219446_at	RIC8B	1.497490258	6.015599556	0.00023605	0.003603409
206465_at	ACSBG1	-1.497408598	4.766853286	0.000177416	0.002925686
220643_s_at	FAIM	1.497252465	5.328676591	0.000286739	0.004175061
219850_s_at	EHF	1.496478901	4.423559571	0.00494747	0.031109873
206138_s_at	PI4KB	1.494925784	7.379725166	0.000789118	0.008474102
213712_at	ELOVL2	1.494606992	4.332307369	0.006130808	0.036051516
200708_at	GOT2	1.494516808	8.428677243	0.001221375	0.011645726

217976_s_at	DYNC1LI1	1.493165575	6.097057386	0.005821934	0.034805993
219741_x_at	ZNF552	1.493011515	4.984498889	0.005044615	0.031540488
205733_at	BLM	-1.492705189	6.451702307	1.25E-08	1.74E-06
202786_at	STK39	1.492428673	7.433708763	0.007231648	0.040506086
218837_s_at	UBE2D4	1.49233887	5.513055045	0.001806157	0.015348973
215030_at	GRSF1	1.491936252	4.615233947	0.000541504	0.006528034
205590_at	RASGRP1	-1.491908579	4.097835382	1.78E-06	8.58E-05
211563_s_at	URI1	1.491535521	5.752984573	0.000656454	0.007403548
203185_at	RASSF2	-1.489725374	5.707751381	2.87E-06	0.000126741
218434_s_at	AACS	1.489455674	6.127492682	0.000399079	0.005205673
204573_at	CROT	1.489017384	7.883996045	0.000296278	0.004276501
200883_at	UQCRC2	1.488906593	7.909102537	0.006637165	0.03809808
206316_s_at	KNTC1	-1.488136356	5.195110736	0.000703929	0.007806822
218660_at	DYSF	-1.487490844	6.330824551	6.58E-05	0.001384457
207220_at	ART4	-1.486682732	4.67293026	1.05E-05	0.000350359
218286_s_at	RNF7	1.485499742	7.470920999	0.000735209	0.008044248
204057_at	IRF8	-1.483400422	5.502383037	0.000478635	0.00596679
201918_at	SLC25A36	1.482420447	7.016333233	0.000865043	0.009081465
221815_at	ABHD2	1.481618383	5.478523584	0.005762847	0.034577082
201582_at	SEC23B	1.48111977	6.888750687	0.00065429	0.007386335
204505_s_at	DMTN	-1.479978164	5.55674546	1.31E-06	6.68E-05
218224_at	PNMA1	1.479868094	6.773042936	0.004012112	0.027055125
221520_s_at	CDCA8	-1.479422711	3.760648188	0.004558438	0.029405546
208717_at	OXA1L	1.479363732	6.970666682	0.00651173	0.037578582
213737_x_at	GOLGA8N	-1.479146513	7.515464837	1.75E-05	0.000512868
203454_s_at	ATOX1	1.479007315	5.713390196	0.008929174	0.046602948
209890_at	TSPAN5	-1.47840946	6.245333647	1.68E-05	0.000495378
202641_at	ARL3	1.477894114	7.4864412	0.000284504	0.00414582
208787_at	MRPL3	1.477618939	8.977844788	0.005118239	0.031794277
214075_at	NENF	1.477393326	5.614126605	3.33E-06	0.000142797
213289_at	APOOL	1.477188092	4.961889266	0.001045753	0.010388234
203448_s_at	TERF1	1.476079523	5.938205976	0.000371184	0.004958515
204745_x_at	MT1G	1.475977254	7.592076648	0.008406466	0.044743145
207621_s_at	PEMT	1.475695773	4.692738856	0.007727332	0.042335963
221552_at	ABHD6	1.475163757	5.219683596	0.001062174	0.010522772
203028_s_at	CYBA	-1.474789499	4.220346215	0.005179523	0.032066051
212692_s_at	LRBA	1.472238706	7.921764908	0.007252141	0.040547141
202672_s_at	ATF3	1.471523872	6.279585938	0.001991825	0.016412287
217552_x_at	CR1	-1.470491141	3.649553652	1.84E-05	0.00053136
209763_at	CHRDL1	-1.470258524	5.234348554	0.003955047	0.026729453
218447_at	CMC2	1.469668969	7.767019981	0.005485935	0.033375506
209035_at	MDK	1.469559311	5.205713181	0.004161664	0.027661801
218732_at	PTRH2	1.469486045	7.575913634	0.001706153	0.014752782
205084_at	BCAP29	1.468701811	5.604950648	0.008987763	0.046761935

214829_at	AASS	1.468669868	4.295362012	0.002550354	0.019515009
219338_s_at	LRRC49	1.468007108	5.557022805	0.000864528	0.009081465
221486_at	ENSA	1.467796436	7.739042798	0.00075255	0.008187176
39966_at	CSPG5	1.467498414	5.550717023	0.002123808	0.017100369
212908_at	DNAJC16	1.467048556	6.419945215	1.65E-05	0.000492509
212216_at	PREPL	1.466214318	6.111324938	0.000422851	0.005404493
201799_s_at	OSBP	1.465876931	5.720846927	0.006761806	0.038619585
212526_at	SPG20	-1.465867294	4.576846788	0.003890811	0.026402657
201764_at	TMEM106C	1.465244663	8.229825805	0.005860215	0.034920695
204675_at	SRD5A1	1.464042842	5.655333854	0.00519216	0.032100816
205174_s_at	QPCT	-1.463854784	3.989438318	0.00055614	0.00665186
202923_s_at	GCLC	-1.463716169	6.540313538	0.000183075	0.002978707
221568_s_at	LIN7C	1.462442219	5.735266673	0.006359085	0.036977724
203667_at	TBCA	1.461117891	9.355492084	0.000263893	0.003920452
39318_at	TCL1A	-1.460831962	4.505844961	0.000353425	0.004794838
202580_x_at	FOXM1	-1.460547145	5.477032578	0.000292457	0.004228023
209192_x_at	KAT5	1.460036666	5.143549095	0.008772123	0.045980103
204565_at	ACOT13	1.459712976	6.297246361	0.002672929	0.020216095
211967_at	TMEM123	1.458510282	8.740320802	0.002120832	0.017093776
218701_at	LACTB2	1.458003208	5.816678188	0.000646386	0.007338504
201606_s_at	PWP1	1.457796963	8.528831232	5.98E-05	0.001294096
200002_at	RPL35	1.457217266	9.376468832	0.000654301	0.007386335
201274_at	PSMA5	1.456439992	8.188839562	0.004594602	0.029575149
218213_s_at	TMEM258	1.455478204	9.430042535	0.002692412	0.020304676
212312_at	BCL2L1	-1.455365027	6.06167995	0.000210829	0.00332097
216295_s_at	CLTA	1.45486095	8.6431345	0.001762968	0.015073003
208912_s_at	CNP	1.45471485	6.693106812	0.001773354	0.0151335
219037_at	RRP15	1.45398533	6.255445305	0.001311595	0.012288752
200777_s_at	BZW1	1.453960827	8.602522471	0.001094619	0.010785079
201250_s_at	SLC2A1	-1.453777142	7.608704983	1.55E-05	0.000471691
203621_at	NDUFB5	1.453490479	8.483802709	0.001451225	0.013132112
213539_at	CD3D	-1.453013167	5.445463591	0.000224517	0.003482587
205417_s_at	DAG1	1.452510444	8.215372561	0.000720944	0.00794507
217773_s_at	NDUFA4	1.451856248	8.878545358	0.000730619	0.008016223
209647_s_at	SOCS5	1.451388838	6.468006593	4.24E-05	0.000983765
207746_at	POLQ	-1.450634021	5.597819461	2.04E-05	0.000568395
823_at	CX3CL1	1.450454423	4.987537957	1.82E-05	0.000529614
203867_s_at	NLE1	1.450319277	5.246252492	0.007920814	0.042983931
202121_s_at	CHMP2A	1.449813524	6.902418452	0.00448448	0.029092649
203245_s_at	LINC00094	1.448991984	4.92128236	0.002063198	0.016829511
209625_at	PIGH	1.447219807	4.980285854	0.005652948	0.034096673
211503_s_at	RAB14	1.446808748	7.329455091	0.003212161	0.022866485
213395_at	MLC1	-1.445654666	3.678529131	0.000155891	0.00266691
219843_at	IPP	1.444879242	5.622088683	8.45E-06	0.000298311

212430_at	RBM38	-1.44458442	5.41152025	6.81E-06	0.000252629
204037_at	LPAR1	1.444315795	5.250173069	0.002225852	0.017751233
209218_at	SQLE	1.442987673	7.095131647	0.000730587	0.008016223
215046_at	KANSL1L	1.442960105	4.257478595	0.001868016	0.015699577
222237_s_at	ZNF112	1.442211065	5.146550179	0.000635946	0.007270865
214864_s_at	GRHPR	1.442140569	7.418238339	0.000122329	0.002210629
218184_at	TULP4	1.441932959	6.517301635	8.11E-05	0.001611146
208159_x_at	DDX11	-1.440444867	6.59553454	0.002858691	0.021161873
221553_at	MAGT1	1.440379012	5.367413378	0.000680934	0.007615272
217898_at	EMC7	1.440261836	8.465185336	0.000646854	0.007338504
222093_s_at	INO80B	-1.439028575	3.715850829	1.80E-05	0.000522864
201953_at	CIB1	1.438336733	8.032639781	0.00084914	0.008955635
218380_at	LOC728392	-1.437881775	5.169111222	0.000444877	0.005607105
49452_at	ACACB	-1.436909071	6.758000969	9.48E-05	0.001801092
212556_at	SCRIB	1.436538728	5.72509893	0.001460537	0.013205647
204266_s_at	CHKA	1.436225308	7.158366	0.001313583	0.012300464
212830_at	MEGF9	-1.43613352	6.698453149	0.000131484	0.0023422
202737_s_at	LSM4	1.435658818	6.851554045	0.004399785	0.028706123
221599_at	AAMDC	1.43545682	5.845633646	0.003704701	0.025432272
209042_s_at	UBE2G2	1.434933553	5.590786394	0.000535133	0.006497936
204236_at	FLI1	-1.434870439	4.166376696	1.93E-05	0.000547514
212554_at	CAP2	1.434387246	4.534089019	0.003237682	0.022974243
207996_s_at	LDLRAD4	1.434215646	5.033883041	0.005990209	0.035487183
201339_s_at	SCP2	1.43391231	7.719176353	0.006095138	0.035911045
209139_s_at	PRKRA	1.433570217	5.505641871	0.007188629	0.040376428
200062_s_at	RPL30	1.433436702	11.73805509	0.00024903	0.003764364
218560_s_at	JMJD4	1.433200612	5.367051444	0.000405863	0.005245533
219504_s_at	RPAP2	1.433199414	4.505987166	0.000493953	0.00610778
218136_s_at	SLC25A37	-1.432637856	6.441219899	0.007407474	0.041100694
221631_at	CACNA1I	-1.432023734	5.050421228	0.00080028	0.00856379
206209_s_at	CA4	-1.431524809	5.035818716	7.19E-05	0.001474855
204859_s_at	APAF1	-1.431442633	5.396203895	2.60E-05	0.000685126
205784_x_at	ARVCF	-1.430726492	4.688291047	0.007430803	0.041167684
206011_at	CASP1	-1.430322679	5.074123822	0.000301132	0.004315917
212885_at	MPHOSPH10	1.430142247	6.55513798	0.002242575	0.017839156
219293_s_at	OLA1	1.429485342	9.248255508	0.000304978	0.004350571
204304_s_at	PROM1	-1.428717787	4.368985149	0.000741123	0.008096571
202427_s_at	MPC2	1.428547212	9.550364439	0.003768976	0.02572864
212310_at	MIA3	1.428545039	7.095153353	0.002694946	0.020315402
204957_at	ORC5	1.427222746	7.667786094	0.004633989	0.029725147
202603_at	ADAM10	1.427172814	8.49381191	0.001764922	0.015075617
200935_at	CALR	1.42573149	4.807231628	0.000934173	0.009570953
206003_at	CEP135	-1.425540482	4.760956981	8.87E-05	0.001710677
221141_x_at	EPN1	-1.425213763	4.004407122	0.000283577	0.004138907

209143_s_at	CLNS1A	1.424772775	8.328720151	0.00175967	0.015055709
207769_s_at	PQBP1	1.42439421	5.298065591	0.00157711	0.013940162
218696_at	EIF2AK3	1.423992801	7.508139738	0.006524519	0.037628638
203925_at	GCLM	-1.423588935	5.515633907	0.001136738	0.011078777
201900_s_at	AKR1A1	1.423080248	8.010505289	0.000236836	0.003609382
203358_s_at	EZH2	-1.42065244	6.551397047	0.001910475	0.015968355
208783_s_at	CD46	1.420296982	8.294799765	0.003684568	0.0253321
201579_at	FAT1	1.420015374	7.810012032	0.000332077	0.004597293
203467_at	PMM1	1.419437582	5.631388153	0.005888598	0.035033666
201622_at	SND1	1.418677426	8.248237748	0.000355908	0.004814236
200610_s_at	NCL	1.418063868	9.521774938	0.002320847	0.018280823
218830_at	RPL26L1	1.41581628	7.426840509	0.001652077	0.014414683
202902_s_at	CTSS	-1.414397485	5.627070507	0.001662861	0.014481145
213543_at	SGCD	1.413325275	4.496725854	0.007566889	0.041706833
210128_s_at	LTB4R	-1.412987549	3.892644699	0.000241989	0.003669568
205596_s_at	SMURF2	1.412649789	6.915688031	6.12E-05	0.001319311
204872_at	TLE4	-1.411256721	5.717436345	0.00725228	0.040547141
221704_s_at	VPS37B	1.411157992	5.359588707	0.005055727	0.031568425
201797_s_at	VARS	1.409613877	4.780073163	0.000642994	0.007326527
217080_s_at	HOMER2	1.408653151	6.649625433	0.000618201	0.007128408
205243_at	SLC13A3	1.408557661	5.01606555	0.002892904	0.021307057
202110_at	COX7B	1.408349612	9.163465574	0.006929266	0.039257252
213308_at	SHANK2	1.407976611	5.629112568	0.000647428	0.007340456
208818_s_at	COMT	1.407562256	7.723507796	0.003554528	0.024724627
212773_s_at	TOMM20	1.407357312	6.735988582	0.000947816	0.009651257
210405_x_at	TNFRSF10B	1.407344738	4.574379407	0.00089306	0.009258666
213015_at	BBX	1.406263519	7.180466389	7.02E-05	0.001450566
218930_s_at	TMEM106B	1.406138906	5.806794745	0.002505604	0.019285559
208611_s_at	SPTAN1	1.406004365	5.914305144	0.000804125	0.008576204
212446_s_at	CERS6	1.404146139	6.106712119	0.000121011	0.002193316
200813_s_at	PAFAH1B1	1.403967273	5.614294894	0.000747146	0.00814291
219200_at	FASTKD3	1.403282022	6.066067419	0.004504422	0.029149634
205758_at	CD8A	-1.403184379	5.801802391	7.10E-05	0.001462955
217591_at	SKIL	1.403001578	4.469956367	0.003211592	0.022866485
221059_s_at	COTL1	-1.402927632	6.544562353	0.001379296	0.012707588
202897_at	SIRPA	-1.40277629	5.157434525	0.000537269	0.00651516
204811_s_at	CACNA2D2	1.402716675	5.475749858	0.003851968	0.026168201
218258_at	POLR1D	1.402599865	8.080575705	0.002765147	0.020682624
220607_x_at	NELFCD	1.402558731	7.919166181	0.000760371	0.008252621
213571_s_at	EIF4E2	1.402538476	7.507280048	0.002466132	0.019079408
221742_at	CELF1	1.401145561	6.691699293	0.000726353	0.00799732
210137_s_at	DCTD	1.400943362	7.452252047	0.001175997	0.011331205
202562_s_at	C14orf1	1.400513855	7.017431671	0.00191493	0.015992665
212822_at	HEG1	1.400499818	6.055405182	0.002374318	0.018582409

205514_at	ZNF415	1.399437212	4.681679969	0.000431332	0.005493978
208782_at	FSTL1	1.39858378	9.626087859	0.009783615	0.049659383
215111_s_at	TSC22D1	1.398005698	9.281919303	0.000688476	0.007672668
214782_at	CTTN	1.397591156	4.808990635	0.000760018	0.008252621
215416_s_at	STOML2	1.397244995	7.645264433	0.003367901	0.023675229
207190_at	ZZEF1	-1.396985882	3.575615922	9.05E-07	4.97E-05
209656_s_at	TMEM47	1.395525927	6.546344145	0.007652895	0.042102809
218580_x_at	AURKAIP1	1.395434708	7.416415729	0.002030026	0.016635549
217957_at	C16orf80	1.395291896	6.720192731	0.002719015	0.020429475
201322_at	ATP5B	1.39464677	9.878610848	0.002001872	0.016468838
207628_s_at	WBSCR22	1.394595878	7.905967956	0.00091459	0.009428418
201019_s_at	EIF1AX	1.393460357	8.094261204	6.90E-06	0.00025551
204402_at	RHBDD3	1.393453631	5.251357448	0.0008041	0.008576204
202971_s_at	DYRK2	1.393165343	4.55016556	0.000180107	0.002954083
203869_at	USP46	1.392127979	4.668836339	0.002386759	0.018637508
217027_x_at	AC004941.5	1.389659821	4.274569999	0.005209129	0.032183972
202109_at	ARFIP2	1.389409122	6.852196294	3.40E-05	0.000828094
222235_s_at	CSGALNACT2	-1.389165741	5.600020061	0.005809315	0.034769505
218220_at	C12orf10	1.388708131	6.711159606	0.002734361	0.020519488
204453_at	ZNF84	1.388365064	6.412902321	0.000644259	0.007334811
212357_at	FAM168A	1.386337696	5.995242552	0.000197939	0.003153235
201036_s_at	HADH	1.385895448	7.091092668	0.008182385	0.04398911
213811_x_at	TCF3	-1.38539428	6.794655715	0.00106176	0.010522772
209102_s_at	HBP1	1.385082506	7.060736978	0.00060785	0.007058009
206059_at	ZNF91	1.384061829	7.852907574	0.002272931	0.017978966
202816_s_at	SS18	1.383589384	5.168963241	0.00404166	0.027194214
203616_at	POLB	1.383180408	7.984343436	0.003180195	0.022691925
203630_s_at	COG5	1.381227455	8.237348496	0.001712134	0.014783527
217956_s_at	ENOPH1	1.38061285	7.291538687	0.000992905	0.009949722
220252_x_at	CXorf21	-1.379966038	5.11935745	3.33E-05	0.000820628
220741_s_at	PPA2	1.379390354	8.044403996	0.005439709	0.033162401
218332_at	BEX1	-1.379106226	5.81101093	0.009083649	0.047100018
208752_x_at	NAP1L1	1.377688227	9.264637896	0.002938071	0.021519971
213752_at	KAZN	-1.3776461	4.095571274	2.42E-05	0.00064707
221582_at	HIST3H2A	1.377138922	6.96088482	0.002677653	0.020227897
203860_at	PCCA	1.37692676	6.289238355	0.000240531	0.003655741
204201_s_at	PTPN13	1.376852424	5.024287645	0.000978723	0.009850795
200826_at	SNRPD2	1.376208471	8.578909876	0.002794063	0.020803221
201029_s_at	CD99	1.376024076	9.6098669	0.001476151	0.013298444
207194_s_at	ICAM4	-1.375411286	4.20374746	2.84E-05	0.000730468
222065_s_at	FLII	1.373881524	5.655941867	0.005854147	0.034907285
40273_at	SPHK2	1.372777677	5.577110943	0.000734429	0.00804266
216836_s_at	ERBB2	1.371700123	6.013624838	3.82E-05	0.000907876
221755_at	EHBP1L1	-1.371285607	3.64359856	0.004984634	0.031254671

221265_s_at	VWA9	1.370954201	6.259104365	0.00034115	0.004673367
220609_at	LOC202181	-1.370267734	4.239463081	0.000318778	0.004467288
218142_s_at	CRBN	1.369981912	6.554616813	0.000108413	0.002012858
217797_at	UFC1	1.369674578	8.484603152	0.006842784	0.038938279
201106_at	GPX4	1.369438555	7.795756177	0.006278421	0.036666262
218500_at	THEM6	1.369367169	4.695727724	0.007250678	0.040547141
209545_s_at	RIPK2	1.369218881	6.708649631	0.00089803	0.009294381
209306_s_at	SWAP70	1.368813839	5.381476379	0.008236053	0.044222236
203136_at	RABAC1	1.367886025	7.331808665	0.007382179	0.040985211
201046_s_at	RAD23A	-1.367838813	6.728028333	0.00121069	0.011598268
201399_s_at	TRAM1	1.367403383	6.964423525	0.001368443	0.012622503
219933_at	GLRX2	1.367334285	7.462090551	0.001643891	0.014363823
219024_at	PLEKHA1	1.36635619	5.852853342	0.000173519	0.002889673
218462_at	RPF1	1.36591393	7.073948962	0.000503548	0.006192933
203910_at	ARHGAP29	1.364068264	7.061841639	0.000684544	0.007638163
203545_at	ALG8	1.3639544	7.704645452	0.001170103	0.011292261
220952_s_at	PLEKHA5	1.363184602	6.348430869	0.000201589	0.003205509
203133_at	SEC61B	1.362889198	10.1777492	0.007291863	0.040582347
211040_x_at	GTSE1	-1.362545688	8.368274611	9.77E-06	0.000329518
34868_at	SMG5	1.362230356	5.628151185	0.001023106	0.010202049
206004_at	TGM3	-1.362206459	4.698940311	0.000257956	0.00386681
208641_s_at	RAC1	1.36205998	8.185613868	0.001888345	0.015816833
202967_at	GSTA4	1.362022717	7.72951501	0.001019361	0.0101758
219570_at	KIF16B	1.361822295	4.643273232	0.000105852	0.001979375
219115_s_at	IL20RA	1.361406372	4.467739302	0.001447775	0.013116941
205929_at	GPA33	1.358249855	5.223436157	0.000583935	0.006889039
213568_at	OSR2	1.357849968	4.69315761	0.005492286	0.033403038
213232_at	KIAA1467	1.357568289	4.291894935	0.000476719	0.00594695
217774_s_at	TRMT112	1.357395393	8.244498528	0.00139941	0.012815427
217800_s_at	NDFIP1	1.357332098	6.634440004	0.001647651	0.014382931
213113_s_at	SLC43A3	-1.355662808	6.767373217	6.49E-06	0.000242163
220336_s_at	GP6	-1.355517227	5.123550849	0.000123435	0.002221822
219376_at	ZNF322	1.354588498	5.298628009	0.001753875	0.015025382
214193_s_at	DIEXF	1.354573212	5.065358389	0.001291001	0.012163745
203450_at	CBY1	1.354252179	7.638966247	0.002091386	0.016960372
205900_at	KRT1	-1.352691588	5.042514946	0.00186421	0.015674793
207890_s_at	MMP25	-1.351642093	4.646865516	0.001717072	0.014817823
200085_s_at	TCEB2	1.351256988	7.98271078	0.002747548	0.020609988
209464_at	AURKB	-1.351202291	4.845926573	0.000710221	0.007862296
209149_s_at	TM9SF1	1.350918896	6.165077438	4.94E-05	0.001105081
208625_s_at	EIF4G1	1.350725015	6.79603606	0.004375522	0.028645051
221726_at	RPL22	1.350391442	9.299269353	0.001788151	0.015233196
219038_at	MORC4	1.349950732	6.05630647	0.00015583	0.00266691
201175_at	TMX2	1.349524225	8.496112636	0.000270546	0.003990112

219356_s_at	CHMP5	1.348936817	6.793798469	0.007493962	0.041422443
200852_x_at	GNB2	1.348624144	6.673334989	0.00307355	0.022164467
218983_at	C1RL	-1.347785366	4.27231497	0.005263698	0.032411621
201682_at	PMPCB	1.347475346	8.604392938	0.001507625	0.013495565
209731_at	NTHL1	1.34735344	4.339513702	0.004168425	0.027690577
211241_at	ANXA2P3	1.347292857	4.663086812	0.002815309	0.020903929
220176_at	NUBPL	1.346929849	4.52668475	0.000473853	0.005915238
210031_at	CD247	-1.346507968	4.385512478	0.00142624	0.012989582
210346_s_at	CLK4	-1.346434284	7.441188222	3.48E-05	0.000843812
217920_at	MAN1A2	1.345485841	5.826083034	0.000730548	0.008016223
202481_at	DHRS3	1.345174139	6.335343154	0.007759586	0.042449093
40420_at	STK10	-1.344176754	5.54028904	1.13E-07	9.54E-06
209828_s_at	IL16	-1.344166484	4.289113526	0.000763746	0.00826312
202960_s_at	MUT	1.344041996	8.001968402	3.12E-05	0.000784157
210218_s_at	SP100	-1.343950866	4.308277766	0.000752359	0.008187176
208683_at	CAPN2	1.342609811	9.091333383	0.000681563	0.007615272
211178_s_at	PSTPIP1	-1.342358624	4.935393616	0.000148412	0.002575111
218773_s_at	MSRB2	1.341654389	7.977804785	0.00956779	0.048971659
204232_at	FCER1G	-1.341555379	6.414062269	2.26E-05	0.00061359
200973_s_at	TSPAN3	1.341371737	4.193966613	0.009346733	0.048068911
210319_x_at	MSX2	1.341332343	4.250755965	0.004480964	0.02908015
206437_at	S1PR4	-1.340655611	4.68952025	0.001430854	0.01301864
203714_s_at	TBCE	1.340601767	6.862066302	0.000336446	0.004636717
211069_s_at	SUMO1	1.339398158	8.95664134	0.002350523	0.018483786
200817_x_at	RPS10	1.339395899	11.74854057	0.002470377	0.019088806
205692_s_at	CD38	-1.338903624	5.687525089	1.51E-05	0.000464341
220059_at	STAP1	-1.338838422	4.383903492	0.007710474	0.042281604
213222_at	PLCB1	1.338131159	6.729677863	0.000814264	0.008639291
203606_at	NDUFS6	1.337944063	8.864283597	0.00227259	0.017978966
45297_at	EHD2	-1.337739285	3.894851906	0.001003623	0.010040618
208113_x_at	PABPC3	1.337311292	10.54805609	0.000517183	0.006330823
212876_at	B4GALT4	1.337253143	5.579218394	0.002083878	0.016930238
217874_at	SUCLG1	1.33492701	8.391971402	0.005773799	0.034602582
212330_at	TFDP1	-1.334027493	6.686673533	0.000153129	0.002632745
201676_x_at	PSMA1	1.333046781	8.98703215	0.009019902	0.04686249
217483_at	FOLH1	1.33240193	5.256838815	0.007067294	0.039890948
211926_s_at	MYH9	-1.33236281	5.365543888	0.004633228	0.029725147
203176_s_at	TFAM	1.33150988	5.449944633	0.002110209	0.017054656
217814_at	CCDC47	1.330304063	7.041380019	0.003315509	0.0233931
203110_at	PTK2B	-1.329833605	5.18128453	0.001418148	0.012954589
213414_s_at	RPS19	1.329690942	11.64228774	0.000711611	0.007868165
203329_at	PTPRM	1.329490994	5.608525701	0.005066663	0.031609581
213766_x_at	GNA11	1.329454117	6.801486269	0.001634012	0.014297994
202232_s_at	EIF3M	1.328268467	6.670792235	0.006969712	0.039428578

221565_s_at	CALHM2	-1.328153485	4.12322884	0.00322944	0.022950225
210724_at	EMR3	-1.327997828	3.857407494	0.000486064	0.006042923
202606_s_at	TLK1	1.327908605	6.630661263	0.003244767	0.023001089
212997_s_at	TLK2	1.327467326	6.152278849	0.002379487	0.01860456
212657_s_at	IL1RN	-1.327173938	5.193561287	0.000580081	0.006867882
219471_at	KIAA0226L	-1.32634442	4.180980849	0.003238605	0.022974243
203718_at	PNPLA6	-1.325832969	5.808961888	0.000216478	0.003392419
205356_at	USP13	1.325579331	7.194885625	1.77E-06	8.56E-05
222365_at	TBCEL	-1.325560764	3.79044168	1.22E-05	0.00039915
200864_s_at	RAB11A	1.324745361	6.354943111	0.000117931	0.002150276
216620_s_at	ARHGEF10	1.324454009	5.912112419	0.004846868	0.030654401
218112_at	MRPS34	1.324078149	5.274361383	0.004552268	0.0293761
201695_s_at	PNP	-1.323878495	7.735842019	0.003040913	0.022020592
217816_s_at	PCNP	1.323466789	8.351483761	0.008985648	0.046761935
206513_at	AIM2	-1.322818432	5.240176838	0.002941913	0.021519971
221488_s_at	CUTA	1.32231175	8.623212849	0.001016593	0.010153715
209770_at	BTN3A1	-1.321306867	5.474537103	0.009226553	0.047678782
208659_at	CLIC1	1.320638273	8.275051314	0.006306848	0.036779222
209550_at	NDN	1.319712142	6.636514535	0.004459065	0.02894831
204324_s_at	GOLIM4	1.319070914	5.142911364	0.008793885	0.046067768
213804_at	INPP5B	-1.31763465	5.16705214	0.000181737	0.002967501
221712_s_at	WDR74	1.316111132	6.038894295	0.001987304	0.016392262
203780_at	MPZL2	1.315975306	5.091329319	0.00296271	0.021638192
209539_at	ARHGEF6	-1.315071057	5.732862946	0.002582574	0.019703845
221808_at	RAB9A	1.314323607	7.199166188	0.004304687	0.028328055
221951_at	TMEM80	1.313111281	5.28142613	0.008394206	0.044743003
205809_s_at	WASL	1.312849111	4.148052838	0.005532418	0.033593947
201420_s_at	WDR77	1.312205576	5.31895806	0.00786545	0.04278325
207812_s_at	GORASP2	1.31179995	7.535949122	0.00205613	0.016790171
219067_s_at	NSMCE4A	1.311790452	6.189231927	0.006127626	0.036044394
219224_x_at	ZNF408	-1.31170779	4.406557445	0.001929718	0.016084335
213958_at	CD6	-1.310823995	4.395114633	0.005961533	0.035416052
213455_at	FAM114A1	1.310786327	7.587615832	0.000257356	0.003860975
218496_at	RNASEH1	1.310084959	6.800262628	0.000432172	0.00549622
202017_at	EPHX1	1.309441282	4.382820021	0.002937236	0.021519971
212567_s_at	MAP4	1.30880209	5.416547454	0.005623311	0.033954064
213713_s_at	GLB1L2	1.30872184	6.603783111	0.000942227	0.009624868
201391_at	TRAP1	1.307964406	7.560916203	0.000420787	0.005397912
221750_at	HMGCS1	1.307743902	6.109404163	0.004532657	0.029271193
204526_s_at	TBC1D8	1.307266275	8.061600981	0.000852259	0.008978175
203556_at	ZHX2	1.30725558	5.814308578	0.000384689	0.005082532
205361_s_at	PFDN4	1.307034407	6.548006971	0.009841138	0.049854498
208841_s_at	G3BP2	1.306948339	7.64030316	0.000523577	0.006387711
208656_s_at	CCNI	1.305825638	9.899837872	0.000232406	0.003562625

209670_at	TRAC	-1.305321653	4.719646173	0.007800884	0.04257313
212339_at	EPB41L1	1.304176945	4.552356401	0.001257659	0.01192331
213256_at	3-Mar	-1.303864238	5.273578919	0.002150253	0.0172776
213049_at	RALGAPA1	1.303818212	6.554779319	0.000244866	0.003710111
213045_at	MAST3	-1.301156247	4.517976289	0.006330029	0.036879123
212129_at	NIPA2	1.300755787	8.372429469	0.002865322	0.021179416
221475_s_at	RPL15	1.298247421	11.37589882	0.000464291	0.005815726
220805_at	HRH2	-1.297466865	3.852287666	0.00015363	0.002636376
207940_x_at	CNR1	-1.29673094	4.020836714	0.000259889	0.003886222
214467_at	GPR65	-1.29601434	4.660737563	0.002277229	0.01800517
221223_x_at	CISH	-1.294814708	5.936710068	0.00042118	0.005397912
213479_at	NPTX2	1.294586242	4.105132634	0.003999655	0.026981071
213067_at	MYH10	1.293664399	4.201109224	0.007750133	0.042422758
207861_at	CCL22	-1.293347595	3.602264431	2.23E-05	0.00060668
213434_at	STX2	-1.293328823	5.573553625	0.0011874	0.011411024
217722_s_at	NGRN	1.292905068	7.882538547	0.00021746	0.00340197
220306_at	FAM46C	-1.292421356	5.350519772	0.000188314	0.003047694
219058_x_at	TINAGL1	-1.292237791	4.409306539	0.000774206	0.008343353
201097_s_at	ARF4	1.291017497	9.712506604	0.001361799	0.012584426
214140_at	SLC25A16	1.290042785	5.889276188	0.008170225	0.043946198
201536_at	DUSP3	1.289401623	6.526231949	0.00034266	0.004683531
220485_s_at	SIRPG	-1.288755975	4.149526616	0.000373006	0.004975596
219621_at	CLSPN	-1.287818665	4.141498954	0.000656636	0.007403548
220659_s_at	C7orf43	-1.287678427	4.499909294	0.007229548	0.040506086
208697_s_at	EIF3E	1.28695992	10.11421919	0.001890515	0.015823224
218967_s_at	PTER	1.286938385	4.882554453	0.001992406	0.016412287
90610_at	LRCH4	-1.286936338	5.961578243	0.000501613	0.006177541
208204_s_at	CAV3	-1.286808947	4.158360504	0.002410066	0.01879543
202418_at	YIF1A	1.286184724	7.120406016	0.000127881	0.002288345
41220_at	9-Sep	1.285385718	9.380582758	0.005426847	0.033137046
209795_at	CD69	-1.284827953	4.10431642	2.71E-05	0.000705297
200029_at	RPL19	1.284632342	11.13939029	0.00061188	0.0070868
217949_s_at	VKORC1	1.284510437	7.46556247	0.007114015	0.040053917
211945_s_at	ITGB1	1.283956118	9.529393199	0.001677531	0.014595001
210967_x_at	CACNB1	-1.283858411	4.462500283	0.002086598	0.016944808
203775_at	SLC25A13	1.283411116	6.286252707	0.004739453	0.030147867
209123_at	QDPR	1.282742411	6.55473247	0.006935986	0.039283155
205612_at	MMRN1	-1.281863083	3.681746031	1.83E-05	0.00053136
213691_at	LOC101928378	-1.281602785	4.054384368	0.000403667	0.005241667
203554_x_at	PTTG1	-1.281301061	8.110370258	0.001488461	0.013382976
205932_s_at	MSX1	1.281128455	5.171330718	0.000404696	0.005241667
212806_at	PRUNE2	1.279759719	4.7110503	0.002369741	0.018560093
204470_at	CXCL1	-1.279644573	4.553648382	0.000537586	0.00651516
217013_at	AZGP1P1	1.279547172	4.228783639	0.005018746	0.031389473

200953_s_at	CCND2	-1.279202969	6.417304892	0.002705643	0.020368155
203332_s_at	INPP5D	-1.279048211	6.26878753	4.30E-05	0.000993502
212871_at	MAPKAPK5	1.278874798	5.550464532	0.007874613	0.042796707
217726_at	COPZ1	1.27736253	8.051460986	0.001993203	0.016412287
207469_s_at	PIR	1.27723403	5.151074724	0.004671605	0.02987214
219720_s_at	GPATCH2L	-1.276980862	4.213271329	0.001469023	0.013247282
206954_at	WT1-AS	-1.27665962	4.214980234	0.001154679	0.011202531
202271_at	FBXO28	1.276655394	7.356274177	0.001383093	0.012736158
209104_s_at	NHP2	1.276626085	8.770688594	0.00853267	0.045165114
207081_s_at	PI4KA	1.276609391	6.066666654	0.007259864	0.040554557
201214_s_at	PPP1R7	1.275897999	7.44307762	0.004576598	0.029501878
201722_s_at	GALNT1	1.274388974	7.269202055	0.004108549	0.027452876
201592_at	EIF3H	1.273799968	9.913230864	0.00556191	0.033714353
219751_at	SETD6	1.273793538	5.796205641	0.000107728	0.002004206
202871_at	TRAF4	1.273513547	4.49968722	0.002365985	0.018546562
216870_x_at	DLEU2	-1.272608781	4.8037366	0.000358888	0.004843802
202936_s_at	SOX9	1.272377167	6.335811157	0.003745804	0.025627857
217500_at	TIAL1	-1.271505649	3.947394519	0.000272175	0.004007677
212515_s_at	DDX3X	1.271113531	5.373658649	0.004135058	0.02754898
206715_at	TFEC	-1.270823375	4.396721446	0.000144709	0.002520424
204970_s_at	MAFG	-1.270526589	6.569915194	3.23E-05	0.000805831
204511_at	FARP2	1.269037352	5.858635539	0.004804501	0.030466269
212080_at	KMT2A	1.268732297	5.744965319	0.007691949	0.042217999
200811_at	CIRBP	1.268320702	7.952952653	0.005051232	0.031568425
209860_s_at	ANXA7	1.268252414	7.740794922	0.004210387	0.027908503
217527_s_at	NFATC2IP	-1.26766984	7.294892222	0.002971159	0.021656662
212307_s_at	OGT	1.267421216	8.232214847	0.004847579	0.030654401
218470_at	YARS2	1.267280344	5.602052031	0.001680391	0.014599046
221484_at	B4GALT5	-1.266909032	7.076820075	0.000306476	0.00435835
201816_s_at	GBAS	1.26636221	7.158589712	0.006661689	0.038190896
201209_at	HDAC1	1.265938081	8.578981063	0.005532861	0.033593947
203181_x_at	SRPK2	1.265592646	7.606502202	0.001927893	0.016077202
210519_s_at	NQO1	1.264302328	7.009706919	0.006873732	0.039003041
215820_x_at	SNX13	1.264301267	4.641612453	0.000584966	0.006889039
218977_s_at	TRNAU1AP	-1.263512345	4.616886074	0.00686629	0.038972906
212088_at	PMPCA	1.263320175	6.484027186	0.002749291	0.020614607
202077_at	NDUFAB1	1.263042634	8.971998277	0.002114071	0.017071902
217641_at	GPR135	-1.262706808	3.605466517	7.30E-06	0.000266082
208087_s_at	ZBP1	-1.262161227	3.623833841	0.000124509	0.002238953
204975_at	EMP2	1.261440082	6.06494741	0.003158115	0.022569599
218208_at	PQLC1	-1.261406721	6.15199577	0.004250937	0.02808567
203653_s_at	COIL	1.260980484	4.700848105	0.000447094	0.005631172
216570_x_at	RP4-595K12.1	1.259852281	7.356311914	0.000440398	0.005566003
221597_s_at	TMEM208	1.25954324	7.320743535	0.001951142	0.016197223

200050_at	ZNF146	1.259321856	8.287818039	0.001217669	0.011623197
203935_at	ACVR1	1.258765584	5.91497615	0.000460481	0.005771955
202535_at	FADD	1.258749747	5.927630429	0.001391815	0.012790707
207815_at	PF4V1	-1.258282318	3.659479755	3.40E-05	0.000828094
207175_at	ADIPOQ	-1.258230557	4.658233429	0.004432026	0.02884341
210418_s_at	IDH3B	1.257462941	8.034525015	0.00748	0.041377567
218984_at	PUS7	1.257000311	7.185019358	5.10E-05	0.001135973
218472_s_at	PELO	1.256772986	6.145583672	0.002163677	0.017370202
206630_at	TYR	-1.256467504	5.091158289	0.003655729	0.025190645
212991_at	FBXO9	-1.256462985	6.270572132	0.000279582	0.004090395
212879_x_at	PIAS4	1.256364529	4.436990056	0.007865678	0.04278325
200006_at	PARK7	1.256254351	10.09719052	0.003924059	0.026592808
218678_at	NES	1.256143416	5.315959387	0.008274624	0.044307582
214224_s_at	PIN4	1.25448927	8.49736998	0.009807321	0.049761547
218027_at	MRPL15	1.253918639	7.561279462	0.002377083	0.018593707
204153_s_at	MFNG	-1.253509227	5.168398315	0.000380371	0.005044396
202234_s_at	SLC16A1	1.253161427	5.645133415	0.005311528	0.032629233
217778_at	SLC39A1	1.252670162	5.166799947	0.000117365	0.002144228
202191_s_at	GAS7	-1.252516848	4.672716867	0.003100463	0.022279475
211937_at	EIF4B	1.252371318	9.295065504	0.0020567	0.016790171
218982_s_at	MRPS17	1.252353527	7.831982317	0.005706362	0.034316983
218605_at	TFB2M	1.251951117	6.956927639	0.004532809	0.029271193
215088_s_at	SDHC	1.251827773	7.468019913	0.000761026	0.008254779
219342_at	CASD1	1.251497626	6.066169852	0.000275193	0.004042354
200074_s_at	RPL14	1.25111496	9.652986677	0.002929669	0.021517181
203680_at	PRKAR2B	-1.251110981	6.15293828	0.004281817	0.028208169
206364_at	KIF14	-1.250503822	5.498102277	4.80E-05	0.001082036
213721_at	SOX2	1.25035362	4.14701567	0.001701475	0.014726257
205831_at	CD2	-1.250184015	5.178874659	1.55E-05	0.000471691
210644_s_at	LAIR1	-1.248778076	6.348914279	1.94E-05	0.000547514
218230_at	ARFIP1	1.247984107	6.709467906	0.000673735	0.007559054
205297_s_at	CD79B	-1.247734976	3.828081456	0.001695026	0.014700695
204336_s_at	RGS19	-1.247544069	6.166650019	1.14E-06	5.97E-05
203806_s_at	FANCA	-1.246771245	3.656472751	0.001523152	0.013588
202013_s_at	EXT2	1.24636018	6.384456927	0.000260913	0.00389516
219377_at	GAREM	1.245869867	5.037364539	0.002284647	0.018048217
218456_at	CAPRIN2	-1.245397219	6.797088892	0.000151165	0.002608925
208636_at	ACTN1	-1.244895547	7.534774044	0.002770157	0.020683635
209076_s_at	WDR45B	1.244263104	8.463072564	0.000955104	0.009709247
205683_x_at	TPSAB1	-1.244147607	4.686625215	0.004033463	0.027159045
40465_at	DDX23	1.243923963	5.795295687	0.005314623	0.032637285
213236_at	SASH1	1.243907097	5.960666579	0.001440579	0.013081083
218192_at	IP6K2	1.243605945	7.943238534	0.004668321	0.029863945
212381_at	USP24	-1.243591386	4.931839469	4.14E-05	0.000964561

212017_at	FAM168B	1.242791542	7.841229952	0.001137926	0.01108001
212169_at	FKBP9	1.242580707	6.523423659	0.000217787	0.003404182
202151_s_at	UBAC1	-1.242081611	6.825317732	0.003779314	0.025780519
208397_x_at	KCNJ5	-1.240654043	3.774966722	0.000411415	0.005302294
204488_at	DOLK	1.240352795	6.753677746	0.000190571	0.003073329
216941_s_at	TAF1B	1.239819926	5.008691152	0.005859448	0.034920695
202308_at	SREBF1	1.239587486	6.260786018	0.002821098	0.020929915
219331_s_at	KLHDC8A	-1.238957311	4.378429512	0.002932533	0.021519971
204871_at	MTERF1	1.237434157	4.492455533	0.009740055	0.049493226
201454_s_at	NPEPPS	1.237023747	7.193965261	0.000769721	0.008308577
213048_s_at	Y16709	1.236168335	8.90143867	0.000616258	0.00711875
218900_at	CNNM4	1.236015623	4.571473759	0.002110383	0.017054656
212099_at	RHOB	1.235674076	5.928906168	0.000591205	0.006913021
204653_at	TFAP2A	1.232847656	6.200354034	0.008335696	0.044508964
212875_s_at	C2CD2	1.232455372	7.020133855	0.00058389	0.006889039
218692_at	SYBU	1.232109347	6.504482214	0.002031233	0.016635549
210151_s_at	DYRK3	-1.232039093	4.739421892	0.001919307	0.016020216
200827_at	PLOD1	1.231891211	5.267942298	0.002256603	0.017888492
214948_s_at	TMF1	1.231730572	8.696615021	0.000797086	0.008544609
201358_s_at	COPB1	1.231602705	9.81683461	0.000459782	0.005767143
210653_s_at	BCKDHB	1.231198344	4.227043123	0.003835616	0.026066794
212892_at	ZNF282	1.230101751	5.360242208	0.001099582	0.010811376
213104_at	TSR3	1.22812244	4.365588603	0.005733337	0.034445227
218360_at	RAB22A	1.227538326	6.450449366	3.35E-05	0.000821919
221196_x_at	BRCC3	1.22717038	5.819567532	0.00121389	0.011611539
213750_at	RSL1D1	1.226718796	6.700808967	0.004741242	0.030148762
217759_at	TRIM44	1.22576789	7.93910093	0.00039563	0.005168063
212982_at	ZDHHC17	-1.225356112	7.628505669	8.74E-05	0.001696952
201552_at	LAMP1	1.225129581	8.252679377	0.002198242	0.017585937
219131_at	UBIAD1	1.225030022	4.612163528	0.005278174	0.032457041
207783_x_at	HUWE1	1.223986471	12.39454483	0.000730796	0.008016223
212089_at	LMNA	1.222458117	5.776556757	0.009651505	0.049221061
218147_s_at	GLT8D1	1.221546917	5.829182209	0.001831036	0.015495443
219186_at	ZBTB7A	-1.220379344	4.83006576	0.00538237	0.032942701
213761_at	MDM1	-1.220298414	5.585403791	0.000174294	0.002895083
215012_at	ZNF451	-1.22003786	6.538891763	0.000923356	0.009481377
207076_s_at	ASS1	1.219023582	6.463677557	0.002209386	0.017651922
205278_at	GAD1	-1.218274779	3.877330902	0.002939381	0.021519971
212023_s_at	MKI67	-1.218025781	5.988313356	0.007968032	0.043163318
212495_at	KDM4B	1.217591223	5.994749252	0.004261519	0.028135255
217909_s_at	MLX	1.217496279	6.211819236	0.001483333	0.013349996
218556_at	ORMDL2	1.216706324	7.698000683	0.009329403	0.048051487
213100_at	UNC5B	1.216297841	5.46196382	0.003085074	0.02221253
214278_s_at	NDRG2	-1.215694167	3.789446625	0.000667496	0.007498263

212565_at	STK38L	1.214945739	5.195561745	0.000400489	0.005212918
218074_at	FAM96B	1.214494332	7.704317749	0.003251073	0.023026962
221340_at	CDX4	-1.214420109	3.590580594	0.000314293	0.004424787
201445_at	CNN3	1.213952951	4.650387091	0.006696077	0.038304815
202486_at	AFG3L2	1.212567913	7.37203473	1.74E-05	0.000508897
214147_at	MROH7	-1.212215044	3.789206424	0.002434055	0.018923453
205014_at	FGFBP1	-1.211964226	4.623208375	0.002939267	0.021519971
218679_s_at	VPS28	1.211818837	7.595947455	0.006046083	0.035725609
209906_at	C3AR1	-1.210442929	5.924418928	0.008306381	0.044443272
220078_at	USP48	-1.210200061	4.678628558	9.70E-06	0.000328398
212928_at	TSPYL4	1.20944361	7.358126242	0.000580585	0.006867882
201138_s_at	SSB	1.209267211	5.9112685	0.00183575	0.015513952
56919_at	WDR48	-1.20905697	5.814064752	5.14E-05	0.001142485
212623_at	TMEM41B	1.208212648	6.554858884	0.001950528	0.016197223
201791_s_at	DHCR7	1.208116106	6.398105159	0.006455875	0.037386014
217525_at	OLFML1	1.207968232	5.353013491	0.005334959	0.032707251
219947_at	CLEC4A	-1.207691162	4.242051231	0.009211715	0.04761556
200096_s_at	ATP6V0E1	1.20742121	8.875183473	0.006134779	0.03606327
213507_s_at	KPNB1	1.206557089	9.462516488	0.000406344	0.00524804
210634_at	KLHL20	1.205375676	5.04411338	0.007983069	0.04323197
202094_at	BIRC5	-1.20533969	5.273724993	0.000727847	0.008008945
202824_s_at	TCEB1	1.204595719	8.363354222	0.002431771	0.018916305
212415_at	6-Sep	-1.204378384	4.367651733	0.005812203	0.034769505
214198_s_at	DGCR2	1.203058844	6.933427964	0.001626747	0.014261719
220971_at	IL25	-1.202996184	3.888330632	0.004055191	0.027245161
219559_at	SLC17A9	-1.202938329	5.556034771	3.95E-05	0.000932179
218087_s_at	SORBS1	-1.202330869	4.100576584	0.004596918	0.029575149
200904_at	HLA-E	-1.202115828	6.743518499	0.001324257	0.012333915
219090_at	SLC24A3	-1.20191064	3.625078219	0.002146545	0.017255394
214032_at	ZAP70	-1.201858326	4.032677147	0.001086192	0.010719529
203801_at	MRPS14	1.200799659	5.860208495	0.003057097	0.022098101
204655_at	CCL5	-1.200722625	5.699856813	5.40E-05	0.00119199
208724_s_at	RAB1A	1.200042537	9.285392316	0.009205012	0.04759692
219363_s_at	MTERF3	1.199970301	6.74422531	0.00368144	0.025329635
205128_x_at	PTGS1	-1.199677107	5.989169	0.000299266	0.004297819
202130_at	RIOK3	-1.199603396	7.60886009	0.000297718	0.004290512
201330_at	RARS	1.198973059	7.110481154	0.003141503	0.022518763
214339_s_at	MAP4K1	-1.198918621	5.259715745	9.17E-06	0.000316309
219281_at	MSRA	-1.198877748	6.59003589	2.21E-05	0.000603197
201776_s_at	EFCAB14	1.198825412	6.260613782	0.001790847	0.015247215
214484_s_at	SIGMAR1	1.198784439	4.222307024	0.005054338	0.031568425
211305_x_at	FCAR	-1.198650649	3.602986349	1.36E-05	0.000431278
213119_at	SLC36A1	-1.198614454	7.003194655	0.004365093	0.028602227
211778_s_at	OVOL2	1.197950484	6.093573217	0.000327819	0.004555591

202225_at	CRK	1.197588385	6.002569294	0.001302787	0.012255849
203184_at	FBN2	-1.197228862	4.678505184	0.005105261	0.031762433
221247_s_at	WBSCR16	1.196277086	6.480142958	0.005618448	0.033954064
208393_s_at	RAD50	1.196059019	6.109299118	0.008730329	0.045839865
203386_at	TBC1D4	1.195234765	6.549652836	0.004069389	0.027330513
218164_at	SPATA20	1.195107123	6.74286647	0.007838825	0.042691016
220526_s_at	MRPL20	1.194688191	7.941661679	0.006299192	0.036758016
219073_s_at	OSBPL10	1.194588648	5.416356938	0.008531119	0.045165114
209379_s_at	CCSER2	1.193339592	5.948804965	0.007337726	0.040775549
216065_at	B3GALT4	-1.193127869	3.897454897	0.000422647	0.005404493
214750_at	PLAC4	-1.193030823	3.59129542	0.00014837	0.002575111
207576_x_at	OXT	-1.192881214	3.607289312	0.000664988	0.00747928
206127_at	ELK3	-1.192759907	4.589200901	0.00143734	0.013064651
217272_s_at	SERPINB13	-1.192212391	4.418748715	0.006772867	0.038645686
203010_at	STAT5A	-1.192058606	5.874117699	8.22E-05	0.001625199
202052_s_at	RAI14	1.191979257	6.594197196	0.00517507	0.032060187
214670_at	ZKSCAN1	1.19164475	7.151277323	0.001602308	0.014121934
215776_at	INSRR	-1.191081232	3.744090083	5.80E-05	0.00126338
202324_s_at	ACBD3	1.19107422	7.455139322	0.000539698	0.006524824
202537_s_at	CHMP2B	1.189273285	6.941203763	0.009706561	0.049391648
202211_at	ARFGAP3	1.189175096	7.064887063	0.004234102	0.028014927
217918_at	DYNLRB1	1.18888259	7.561723161	0.00581393	0.034769505
220449_at	LINC01260	-1.188462421	4.725062716	0.005560866	0.033714353
210783_x_at	CLEC11A	-1.187849826	4.216908414	0.009352958	0.048087401
203166_at	CFDP1	1.186880629	7.066316801	0.008402671	0.044743145
214135_at	CLDN18	-1.186469832	4.908360155	0.001444184	0.013107316
218281_at	MRPL48	1.186253623	7.336697604	0.004910099	0.030953427
203792_x_at	PCGF2	-1.185144904	4.011192079	0.001047923	0.010404135
220471_s_at	MYCT1	-1.183555826	4.42908889	0.001341307	0.012439056
218003_s_at	FKBP3	1.183480558	8.271337105	0.003948592	0.026708964
200881_s_at	DNAJA1	1.18299847	9.10104926	0.003267925	0.023128409
210015_s_at	MAP2	1.182626881	4.82173128	0.004692913	0.029976942
213313_at	RABGAP1	1.182189567	7.764028849	0.00501174	0.031356384
211370_s_at	MAP2K5	1.181108857	5.274270435	0.008273376	0.044307582
203314_at	GTPBP6	1.180945644	6.7888037	0.002196269	0.017577843
221039_s_at	ASAP1	-1.180878353	5.2637393	0.000228192	0.003517912
215157_x_at	PABPC1	1.18047325	11.31753856	0.000490549	0.00608393
203201_at	PMM2	1.179924859	5.68070255	0.003033813	0.021981925
210313_at	LILRA4	-1.179852966	3.456678826	3.80E-05	0.000905762
202020_s_at	LANCL1	1.177582581	7.586889462	0.000935596	0.009574802
212589_at	RRAS2	1.177477607	6.114478144	0.008998435	0.04676507
202464_s_at	PFKFB3	-1.175564776	6.185843355	0.002867587	0.021180306
222022_at	DTX3	-1.173900838	4.350713921	0.002480536	0.01914322
219026_s_at	RASAL2	1.17273892	4.303889254	0.008417502	0.044775824

202041_s_at	FIBP	1.172722425	6.964157606	0.000917699	0.009441966
218252_at	CKAP2	-1.171969393	6.394383419	0.001656503	0.014439528
201507_at	PFDN1	1.171931095	5.841750105	0.005089785	0.031703676
206420_at	IGSF6	-1.170523047	5.137942915	0.003562547	0.024753743
211434_s_at	CCRL2	-1.169624628	3.994106052	0.003423631	0.023979843
205235_s_at	KIF20B	-1.169274738	4.661641275	0.005275372	0.032454372
211724_x_at	MIOS	1.168403356	8.086149026	0.006026561	0.03563758
205587_at	FGFR1OP	-1.168078535	3.995658653	0.005719188	0.034382809
213512_at	C14orf79	1.166954019	5.483742403	0.002500364	0.019261436
201612_at	ALDH9A1	1.166770298	8.785774166	0.000648555	0.007344311
203971_at	SLC31A1	1.166728001	6.61142746	0.002591521	0.019755618
217749_at	COPG1	1.166181111	6.068875737	0.000189938	0.003065834
219290_x_at	DAPP1	-1.165756569	6.639773376	8.14E-05	0.00161489
203418_at	CCNA2	-1.165545555	5.382309116	0.000240694	0.003655741
206631_at	PTGER2	-1.165340316	3.746748282	0.003595616	0.02491725
206324_s_at	DAPK2	-1.165258605	4.862593281	0.006394117	0.037110633
207094_at	CXCR1	-1.165165856	3.80811906	0.000997753	0.009992826
208635_x_at	NACA	1.164464801	11.38836172	0.001517203	0.013548151
220968_s_at	TSPAN9	1.163732283	5.619692451	0.000260811	0.00389516
219882_at	TTLL7	1.162811939	5.432809899	0.005186098	0.032085031
206621_s_at	EIF4H	1.162618717	9.12812639	0.003206181	0.022841699
203039_s_at	NDUFS1	1.161955343	6.322030015	0.00673528	0.038492124
207474_at	SNRK	-1.161808264	4.390967444	0.0012899	0.012159637
209635_at	AP1S1	1.161618126	6.033534657	0.000488343	0.006063014
202491_s_at	IKBKAP	1.161151761	7.809529375	0.004629674	0.029718313
201561_s_at	CLSTN1	1.16031523	7.021018722	9.18E-05	0.001755673
204523_at	ZNF140	1.160191232	5.731266096	0.002785208	0.020764734
201303_at	EIF4A3	1.160090063	8.584703307	0.009518644	0.048774714
206562_s_at	CSNK1A1	1.159939983	7.805242429	0.00116555	0.011266165
215105_at	N4BP2L2-IT2	-1.15936598	4.28818257	0.002114384	0.017071902
221188_s_at	CIDEB	-1.15916426	5.609781905	0.002466301	0.019079408
201190_s_at	PITPNA	1.158448441	7.13091287	0.003065763	0.022143235
211707_s_at	IQCB1	1.157677398	6.806507231	0.002658625	0.020149576
220966_x_at	ARPC5L	1.157602197	8.359630678	0.001328824	0.012362404
218124_at	RETSAT	1.157378863	6.726176742	0.000272934	0.004015614
201671_x_at	USP14	1.156756189	5.986465199	0.006541907	0.037681382
201587_s_at	IRAK1	1.156279449	8.564983639	0.008993324	0.04676507
218302_at	PSENEN	1.15506144	5.67924765	0.007350527	0.040821877
204744_s_at	IARS	1.154849081	9.288678919	0.007833424	0.04268336
221987_s_at	TSR1	1.154530459	5.354683445	0.004651247	0.029804489
221664_s_at	F11R	1.154208956	6.326800071	0.000495271	0.006119937
209049_s_at	ZMYND8	-1.154125406	6.859128791	0.007662261	0.042130917
201258_at	RPS16	1.153942155	10.71410425	0.007508765	0.041461439
208868_s_at	GABARAPL1	-1.152642945	4.228725801	0.000336446	0.004636717

207158_at	APOBEC1	-1.15261001	4.649138122	0.001320579	0.012330312
212334_at	GNS	1.151978271	7.90102636	0.00407253	0.02733153
200017_at	RPS27A	1.151952941	11.20060987	0.004537578	0.029291641
36545_s_at	SFI1	-1.15145567	4.536248019	0.009844753	0.049858998
218614_at	KIAA1551	-1.15114648	6.335568925	0.001122115	0.010962203
220851_at	BC069756	-1.150847967	4.024399513	0.004048128	0.027227717
219666_at	MS4A6A	-1.150812652	7.147227389	0.006819874	0.038866266
201597_at	COX7A2	1.150112482	9.805529675	0.003264115	0.023110392
221027_s_at	PLA2G12A	1.148814495	6.461995612	0.001858493	0.015651633
200034_s_at	RPL6	1.14842363	10.92942701	0.00332234	0.023431917
216654_at	TNXB	-1.147031605	4.303604732	0.001768504	0.015099164
212285_s_at	AGRN	1.147002225	6.149065658	0.008399618	0.044743145
219956_at	GALNT6	-1.146701734	5.152000371	0.000140139	0.002459565
219553_at	NME7	1.146556434	5.967391401	0.00062687	0.007213297
203081_at	CTNNBIP1	-1.146000706	4.66328858	0.003938772	0.02666444
204715_at	PANX1	1.145689018	5.433085417	0.000219552	0.003425912
220712_at	C8orf60	-1.145017522	5.960707732	0.00036067	0.004853526
220144_s_at	ANKEF1	1.144830899	3.984003394	0.001069145	0.010586094
205642_at	CNTRL	-1.144770905	5.508411484	0.000206506	0.003261301
211037_s_at	MBOAT7	-1.144620227	3.812446042	0.001072322	0.010606072
219177_at	BRIX1	1.144276001	5.260928702	0.007230045	0.040506086
217885_at	IPO9	1.14424282	7.725240835	0.001143875	0.01112723
209694_at	PTS	1.143520141	7.747053198	0.000914216	0.009428418
217988_at	CCNB1IP1	1.143509659	7.149558221	0.000804254	0.008576204
201198_s_at	PSMD1	1.143159911	8.048462473	0.006831429	0.038919197
211822_s_at	NLRP1	-1.142984956	4.132814645	0.001725264	0.014863393
203302_at	DCK	-1.142920712	5.380692607	0.003185545	0.022712379
205944_s_at	CLTCL1	-1.14256699	4.552289141	0.002770938	0.020683635
212942_s_at	CEMIP	1.141992927	4.79337418	0.00919677	0.047578652
202532_s_at	DHFR	-1.141845925	6.059417409	0.008323158	0.044492002
200043_at	ERH	1.141348589	9.232770265	0.004130173	0.027536497
218412_s_at	GTF2IRD1	1.140972729	5.956186383	0.000222364	0.003460926
214287_s_at	CDK13	-1.140255713	4.508632661	0.000265807	0.003942486
208756_at	EIF3I	1.139312208	8.651552503	0.000848326	0.008952218
206308_at	TRDMT1	1.137580491	4.275241999	0.000307575	0.004367179
219452_at	DPEP2	-1.136531336	3.404550048	0.001896341	0.015864723
207809_s_at	ATP6AP1	1.134506532	7.954210175	0.000808187	0.008593145
222056_s_at	FAHD2A	1.134446385	6.596325522	0.002858451	0.021161873
210789_x_at	CEACAM3	-1.13338447	5.872849084	1.64E-05	0.000490167
208031_s_at	RFX2	-1.132883255	3.761148588	0.00039615	0.005171156
219162_s_at	MRPL11	1.132173363	6.276883314	0.001551184	0.013764216
213376_at	ZBTB1	1.131537985	6.907987315	0.000362072	0.004868807
205987_at	CD1C	-1.131128207	3.480363322	0.00035557	0.004813221
200781_s_at	RPS15A	1.12964998	10.83702572	0.001939476	0.016137009

213623_at	KIF3A	1.129135563	4.756326293	0.000778363	0.00838322
221250_s_at	MXD3	-1.128694469	3.569904519	0.000876041	0.009139213
201240_s_at	SPCS2	1.128357478	10.35331925	0.001501691	0.013462215
205807_s_at	TUFT1	1.126363937	6.761195659	7.34E-05	0.001493729
203585_at	ZNF185	-1.126204759	5.843124191	0.008189987	0.04401366
221601_s_at	FAIM3	-1.125910165	4.972480254	0.000613197	0.007097561
218216_x_at	ARL6IP4	1.125786024	7.825098333	0.002972573	0.021658336
203149_at	PVRL2	1.125776307	6.267627531	0.000147922	0.002571481
31835_at	HRG	-1.125669498	4.223604356	0.002146093	0.017255394
219598_s_at	RWDD1	1.124835562	8.220412286	0.00301167	0.021873478
210954_s_at	TSC22D2	-1.124220282	3.449521436	4.41E-05	0.001013429
202421_at	IGSF3	1.124000538	6.961408209	0.001801517	0.015316664
201628_s_at	RRAGA	1.123549473	7.595351616	0.004032676	0.027159045
212824_at	FUBP3	1.123194486	5.160623673	0.002178589	0.017462136
210502_s_at	PPIE	1.123106479	6.818317525	0.004734833	0.030147867
206539_s_at	CYP4F12	-1.122826997	5.301205736	0.009717797	0.049393852
212074_at	SUN1	1.122712473	6.937197677	0.000838551	0.008854171
219182_at	TMEM231	1.12219859	5.961840089	0.001284435	0.012128049
216364_s_at	AFF2	-1.122001801	3.699101671	2.99E-05	0.000757948
202915_s_at	FAM20B	1.121644648	5.446095768	0.008317543	0.044489975
202408_s_at	PRPF31	1.121563524	7.046960098	0.001044732	0.010388234
218336_at	PFDN2	1.121345277	7.510202418	0.004902925	0.030928129
204980_at	CLOCK	1.121321809	7.373612651	0.000373644	0.004980474
208196_x_at	NFATC1	-1.121185618	4.946556084	0.008644155	0.045557438
201023_at	TAF7	1.121180333	8.246337631	0.00275105	0.020619346
204118_at	CD48	-1.12094179	6.587630639	0.000230533	0.003542841
203140_at	BCL6	-1.120530742	8.131561085	0.009872175	0.049956372
202634_at	POLR2K	1.119564176	6.590460264	0.002968751	0.021656662
220036_s_at	LMBR1L	1.119450029	6.221132037	0.00023424	0.003581762
221698_s_at	CLEC7A	-1.118972746	5.068847408	0.004902932	0.030928129
211208_s_at	CASK	1.118492084	6.894888665	0.001461166	0.013205647
202217_at	C21orf33	1.117879279	8.263029866	0.002245387	0.017853753
203713_s_at	LLGL2	1.117554112	5.775273804	0.004281512	0.028208169
218898_at	FAM57A	1.117080627	6.585809806	0.003468592	0.0242282
202325_s_at	ATP5J	1.116869388	8.497487929	0.007865557	0.04278325
215269_at	TRAPPC10	-1.116634494	5.92009398	0.001206167	0.011560997
211197_s_at	ICOSLG	1.116505944	4.225361228	0.004215831	0.027934461
211728_s_at	HYAL3	-1.115959047	3.897647392	0.008711937	0.04578273
217620_s_at	PIK3CB	-1.114997602	4.148653617	0.001572972	0.013915293
204328_at	TMC6	-1.11489886	6.087368594	0.001104535	0.010836767
219669_at	CD177	-1.114599352	4.945260108	0.005780545	0.034626469
221229_s_at	TRMT61B	1.114087587	6.39766468	0.006853281	0.038959531
214113_s_at	RBM8A	1.11384925	7.020202191	0.007279394	0.040582347
220474_at	SLC25A21	-1.113755216	3.922656628	0.000591003	0.006913021

218026_at	COA3	1.113663345	8.106801218	0.007123429	0.040084084
204510_at	CDC7	-1.113152362	6.13694597	3.01E-06	0.000131966
205467_at	CASP10	-1.112480797	5.103185426	0.005194693	0.032105623
207023_x_at	KRT10	1.112361315	7.743135138	0.007934713	0.043033442
204912_at	IL10RA	-1.11190613	5.817248805	2.37E-05	0.000634731
213007_at	FANCI	-1.111780522	5.85923314	0.001217371	0.011623197
201916_s_at	SEC63	1.111659624	8.198092807	0.005828255	0.034832397
202848_s_at	GRK6	-1.111293502	5.427166063	0.008952676	0.046687048
209764_at	MGAT3	-1.111261325	3.833070092	0.00126624	0.01198602
204355_at	DHX30	1.111161239	6.09585675	0.00607822	0.035846016
203878_s_at	MMP11	1.11078855	5.723051668	0.002006985	0.016502497
200743_s_at	TPP1	-1.110656316	7.651257782	0.000153174	0.002632745
209971_x_at	AIMP2	1.108656924	8.169933002	0.002538384	0.019475687
208999_at	8-Sep	1.108392758	6.724161724	0.002571092	0.019657242
209817_at	PPP3CB	1.108271337	6.319848971	0.003270466	0.023137441
205663_at	PCBP3	-1.10795221	4.004227876	0.003694718	0.025392333
209560_s_at	DLK1	-1.107807514	4.385052481	0.002486349	0.019161543
212731_at	ANKRD46	1.107629633	6.506917757	0.000908005	0.009376391
219283_at	C1GALT1C1	1.107520219	6.957422875	0.006136786	0.036063479
206197_at	NME5	1.106093496	6.303660895	0.009332835	0.048051487
217450_at	AGFG2	1.105466834	3.965807807	0.004455773	0.028941164
220646_s_at	KLRF1	-1.105361519	3.87740598	0.000700805	0.007781614
211474_s_at	SERPINB6	1.105314688	8.710766614	0.003953551	0.026729219
201593_s_at	ZC3H15	1.105197204	8.060232705	0.009666748	0.049257591
218386_x_at	USP16	1.104828934	7.617449048	0.003129293	0.022451604
204066_s_at	AGAP1	1.104531489	5.819327059	0.000890025	0.009237672
210409_at	MLLT4-AS1	1.1042735	5.365013011	0.005843717	0.034879206
207681_at	CXCR3	-1.103083361	3.778222643	0.001214615	0.011611539
206109_at	FUT1	-1.101805799	5.753563156	0.002706399	0.020368155
218030_at	GIT1	-1.101072688	5.790344121	0.001817906	0.015420164
218503_at	FOCAD	1.100524515	6.382201851	0.000871951	0.009124197
210487_at	DNTT	-1.100446034	3.448224805	0.001383984	0.01273795
202744_at	SLC20A2	1.099695825	4.402284741	0.006887965	0.039047457
217418_x_at	MS4A1	-1.097997385	4.768519769	0.007113235	0.040053917
222078_at	PKLR	-1.097770296	3.530866531	0.000268985	0.003970294
221692_s_at	MRPL34	1.09771948	5.839480833	0.008776089	0.045987711
207473_at	MLN	-1.097515684	5.020321522	0.00087066	0.009121393
215171_s_at	TIMM17A	1.097145738	8.469734191	0.003510381	0.024484305
201032_at	BLCAP	1.096775102	8.636669789	0.000632631	0.007249094
212906_at	GRAMD1B	-1.096677895	5.892617008	0.000186858	0.003029492
213355_at	ST3GAL6	-1.096580448	4.363710202	0.001320476	0.012330312
202879_s_at	CYTH1	-1.096044046	3.958666545	0.000709503	0.00785911
207602_at	TMPRSS11D	-1.095421616	5.324499345	0.001340067	0.01243386
202278_s_at	SPTLC1	1.095287165	4.258536407	0.005469653	0.033298605

213391_at	DPY19L4	1.094538123	6.338381082	0.004280205	0.028208169
220134_x_at	EVA1B	1.094501573	4.337242925	0.00152724	0.013611193
216835_s_at	DOK1	-1.092798945	5.441284145	0.000471511	0.005898078
202625_at	LYN	-1.092303216	5.314209547	0.003918215	0.026568897
214998_at	AAK1	-1.09230118	3.882956144	0.000669327	0.007514209
207734_at	LAX1	-1.09195593	5.610721605	0.000142719	0.002492881
213826_s_at	H3F3A	1.090853213	5.27478153	0.004050372	0.027232798
210216_x_at	RAD1	1.089740177	6.043770904	0.001596003	0.01409353
200945_s_at	SEC31A	1.089704478	8.452808647	0.006446225	0.037365627
206534_at	GRIN2A	-1.089378939	4.177076886	0.007936997	0.043033442
202389_s_at	HTT	1.089170764	5.61062288	0.006179767	0.036222944
218721_s_at	C1orf27	1.089005257	6.498213426	0.003429587	0.02401236
211325_x_at	DSTNP2	1.08863514	5.205550196	0.001704012	0.014741235
208090_s_at	AIRE	-1.088462896	3.548795492	0.000884951	0.009205904
220577_at	GVINP1	-1.088397614	5.000414224	0.005608546	0.033929567
213070_at	PIK3C2A	1.086899233	7.002768166	0.00437946	0.028645051
218203_at	ALG5	1.086733772	8.170048777	0.000911981	0.009412139
220808_at	THEG	-1.085658843	3.70188916	0.000592087	0.00691891
220553_s_at	PRPF39	1.085644101	6.012891193	0.001157345	0.011222441
216551_x_at	PLCG1	-1.085586425	4.971265069	0.00304707	0.022051769
201282_at	OGDH	1.08537754	4.45213898	0.002362357	0.018541968
205968_at	KCNS3	1.083975152	6.289700141	0.00832522	0.044492002
214155_s_at	LARP4	1.083618594	4.948503438	0.00136012	0.012581626
205219_s_at	GALK2	1.083084315	4.488064551	0.001908446	0.01595869
201121_s_at	PGRMC1	1.082435666	8.292757859	0.000107036	0.00199539
203218_at	MAPK9	1.082054699	6.863679208	0.003970745	0.026815726
205488_at	GZMA	-1.081585488	5.123347496	0.000970291	0.009809112
209510_at	RNF139	1.081232132	7.573317221	0.002968947	0.021656662
211802_x_at	CACNA1G	-1.080966327	3.669176226	0.000692724	0.007710611
212973_at	RPIA	-1.080724429	7.570283716	0.000554744	0.006639499
219449_s_at	TMEM70	1.080620592	8.052104407	0.00986311	0.04993814
212672_at	ATM	-1.079769288	4.930471123	0.001465204	0.013232423
203549_s_at	LPL	-1.078751047	7.195282527	0.007829016	0.04268336
203320_at	SH2B3	-1.077990693	6.216826745	0.000107016	0.00199539
207001_x_at	TSC22D3	1.077947887	4.187372477	0.002447413	0.018989513
203119_at	CCDC86	1.077399957	6.555217948	1.49E-05	0.000459319
214352_s_at	KRAS	1.07712255	8.279639032	0.002527315	0.019419971
212034_s_at	EXOC7	1.074499103	7.766942584	0.003248588	0.023018275
202550_s_at	VAPB	1.074195194	7.691240059	0.006383993	0.037063639
202365_at	UNC119B	1.073753983	7.450674793	0.002860994	0.021161873
200745_s_at	GNB1	1.073434781	7.072960214	0.002124455	0.017100369
214907_at	CEACAM21	-1.072339436	5.120263714	0.001241336	0.011793016
212006_at	UBXN4	1.071637085	7.368351527	0.002365097	0.018546562
209593_s_at	TOR1B	1.070582681	7.110192236	0.004497253	0.029113542

201921_at	GNG10	1.070196219	8.874038237	0.004138688	0.027563122
207113_s_at	TNF	-1.069904596	4.331491693	0.009129442	0.047310638
212502_at	ADO	1.069887078	6.390409656	0.005669289	0.034161433
201944_at	HEXB	1.068043368	8.875215674	0.006451844	0.037386014
217207_s_at	BTNL3	-1.067789382	6.130759379	9.29E-05	0.001769868
206725_x_at	BMP1	-1.067484497	3.692384526	0.00020693	0.003265169
219822_at	MTRF1	-1.066998373	4.480403343	0.008701958	0.04578273
206160_at	APOBEC2	-1.064392161	5.288016389	0.000322414	0.004497571
213499_at	CLCN2	1.062834588	4.704796528	0.009883695	0.049959373
204546_at	KIAA0513	-1.062738423	5.609415105	0.004665089	0.029861796
208289_s_at	EI24	1.062307936	6.897595487	0.000588902	0.006907751
204450_x_at	APOA1	-1.061975669	3.892064856	0.004589749	0.029555398
208967_s_at	AK2	1.061372432	7.934205802	0.006322015	0.036844172
208667_s_at	ST13	1.060482673	8.269247072	0.004433444	0.02884341
218897_at	TMEM177	1.060307268	5.361035572	0.008579622	0.045348015
203026_at	ZBTB5	1.059955988	7.251162553	0.000188834	0.003053407
203164_at	SLC33A1	1.059932991	6.719912795	0.003281011	0.023203067
202853_s_at	RYK	1.05862829	7.235008512	0.001923674	0.016049339
200873_s_at	CCT8	1.05812333	9.564109868	0.008430557	0.04480617
200627_at	PTGES3	1.057940114	9.397510728	0.00575009	0.034511863
208919_s_at	NADK	-1.057613343	6.974735521	0.000792649	0.008505373
206761_at	CD96	-1.057317501	3.887079882	0.001601786	0.014121934
203341_at	CEBPZ	1.057041033	6.840661909	0.009369132	0.048116452
214730_s_at	GLG1	1.0570391	8.530522727	0.001486974	0.013376181
201633_s_at	CYB5B	1.05633334	4.380104646	0.002076364	0.016903792
200718_s_at	SKP1	1.055920625	10.6355682	0.001435976	0.013058743
218424_s_at	STEAP3	-1.052943371	6.595797354	0.000310169	0.004379873
201074_at	SMARCC1	1.052816101	7.627791853	0.00335964	0.023640282
218858_at	DEPTOR	1.051785005	6.19445643	0.001880394	0.015775871
203197_s_at	C1orf123	1.049433722	6.61219005	0.006604771	0.037924038
213207_s_at	GOSR2	1.04885614	5.228239994	0.001190327	0.011433144
218241_at	GOLGA5	1.0487582	7.115662033	0.009053107	0.046984337
202860_at	DENND4B	-1.048716709	6.475448558	0.00032064	0.004483571
212761_at	TCF7L2	1.047099967	8.253961994	0.008031892	0.043380758
216034_at	ZNF280A	-1.04700739	5.658317667	0.000682558	0.007620643
214819_at	IQSEC2	1.046673275	3.887269723	0.002758053	0.020653063
210114_at	INVS	1.046439575	5.857843257	0.000635923	0.007270865
213794_s_at	NGDN	1.046276842	7.095042883	0.002770862	0.020683635
212717_at	PLEKHM1	-1.04599826	5.98540249	0.001888887	0.015816833
202279_at	C14orf2	1.044731167	8.124766998	0.006200823	0.036307138
215517_at	PYGO1	1.044507066	3.885366085	0.006403088	0.037150914
219661_at	RANBP17	1.043857066	5.538879354	0.00151958	0.013562752
206398_s_at	CD19	-1.043631955	4.130870818	0.007571038	0.041717124
201405_s_at	COPS6	1.043125543	7.962872229	0.00898573	0.046761935

206006_s_at	CEP162	-1.042914965	5.425440234	0.005736727	0.034454275
201362_at	IVNS1ABP	-1.041897918	6.4963669	0.002023599	0.016602776
201027_s_at	EIF5B	1.04165373	6.629577532	0.008563589	0.045289452
203701_s_at	TRMT1	1.040999225	5.215509378	0.008917253	0.046580613
201290_at	SEC11A	1.040600626	9.842573842	0.009066036	0.047035367
218259_at	MKL2	1.040033139	7.084115255	0.000480255	0.0059829
213913_s_at	TBC1D30	1.038198359	4.896577384	0.008427547	0.04480617
209408_at	KIF2C	-1.037162266	6.883446655	0.001172465	0.011309091
209784_s_at	JAG2	1.036572377	5.883193966	0.006571525	0.037768714
204628_s_at	ITGB3	-1.035555816	5.070368405	0.002931157	0.021519472
221252_s_at	GSG1	-1.035124429	4.153187761	0.001334444	0.012400568
217627_at	ZNF573	1.03474878	6.597005932	0.007780174	0.042498842
205474_at	CRLF3	-1.03470978	6.417961724	7.44E-05	0.001507304
208514_at	KCNE1	-1.034691624	4.435300928	0.00607125	0.035826982
209401_s_at	SLC12A4	-1.034170496	4.518914238	0.003156624	0.02256776
204687_at	PARM1	1.033511927	7.039170178	0.000618803	0.007130862
214566_at	SMR3A	-1.033401082	3.763288452	0.002271265	0.017978966
205805_s_at	ROR1	1.033168518	5.184087242	0.007886246	0.042834471
220061_at	ACSM5	-1.033036443	4.203220221	0.007041617	0.039770568
220138_at	HAND1	-1.0316102	5.102102717	0.003600506	0.0249353
203849_s_at	KIF1A	-1.031475003	4.546349339	0.007417327	0.041130125
208093_s_at	NDEL1	-1.031223556	5.996453929	0.00107541	0.010625119
218901_at	PLSCR4	-1.030383435	5.238704681	0.00774168	0.042401868
212377_s_at	NOTCH2	1.029948304	7.84410461	0.004019578	0.027095479
212119_at	RHOQ	1.029703842	6.593767671	0.004086891	0.027374948
221032_s_at	TMPRSS5	-1.028939643	5.024781704	0.008571569	0.045318547
33736_at	STOML1	1.027935656	5.876602824	0.008743319	0.045881726
210682_at	LPO	-1.027579913	3.822433752	0.002607944	0.019847724
200792_at	XRCC6	1.027336001	9.650217092	0.00485391	0.030681929
209633_at	PPP2R3A	1.027008046	5.690653753	0.001060952	0.010522069
211210_x_at	SH2D1A	-1.026683167	4.854484136	0.009317829	0.048028316
220539_at	TTC40	1.026132244	3.869218514	0.007207684	0.040429163
218141_at	UBE2O	-1.024895832	3.846440436	0.00675095	0.038569628
211934_x_at	GANAB	1.024762427	4.908772896	0.003502471	0.024438452
220164_s_at	FBXO40	-1.024238672	4.138013842	0.008405495	0.044743145
220323_at	CNTD2	-1.024160359	6.790520314	0.000487515	0.006056842
215685_s_at	DLX2	-1.023931829	4.412584741	0.001397555	0.012811271
206855_s_at	HYAL2	1.023384406	5.73715025	0.006083156	0.035863556
206674_at	FLT3	-1.022624077	4.943162474	0.001342759	0.012446213
215170_s_at	CEP152	-1.022120961	4.300901875	0.001007023	0.010069128
214096_s_at	SHMT2	1.021721596	8.207214428	0.002888571	0.021296164
207301_at	EFNA5	-1.018468338	3.637688153	0.00283114	0.020995902
206411_s_at	ABL2	1.018030176	5.280425202	0.000228333	0.003517912
215299_x_at	SULT1A1	-1.017455399	7.239401657	0.000431199	0.005493978

219558_at	ATP13A3	1.017117109	4.635874484	0.004385494	0.028674263
218132_s_at	TSEN34	1.017080651	7.834610382	0.001796722	0.015283005
213870_at	COL11A2	1.016708127	3.879196287	0.009575608	0.048997964
219221_at	ZBTB38	1.016663143	5.459670495	0.004448656	0.028932086
212505_s_at	MAU2	-1.015311324	5.936747103	0.001411628	0.012907925
203049_s_at	TTC37	1.0150957	6.040430617	0.006564166	0.037738278
222130_s_at	FTSJ2	1.013949642	6.095716479	0.006198636	0.036307138
220202_s_at	RC3H2	1.013338408	5.419976639	0.002877136	0.02123368
203052_at	C2	-1.010752492	5.12869541	0.009329786	0.048051487
211566_x_at	BRE	1.010643153	6.280121579	0.002426804	0.018891268
210176_at	TLR1	-1.010426898	4.123721569	0.000290163	0.004206684
221010_s_at	SIRT5	-1.01039076	5.272281543	0.001882247	0.015775871
202510_s_at	TNFAIP2	-1.010249049	6.210108973	0.001843002	0.015560858
221080_s_at	DENND1C	-1.009600969	5.599085622	0.003668394	0.025258883
203820_s_at	IGF2BP3	-1.008011536	4.191712508	0.006837109	0.038928096
203673_at	TG	-1.008006218	4.869629496	0.005428619	0.033137046
207804_s_at	FCN2	-1.007694512	3.982537315	0.001881764	0.015775871
206396_at	SLC1A1	-1.006197634	3.852774776	0.004996421	0.031292653
208474_at	CLDN6	1.005384803	4.317533453	0.003021346	0.021926343
219099_at	C12orf5	1.004888247	5.658050959	0.007396842	0.041054157
210234_at	GRM4	-1.004771855	5.231684471	0.002117863	0.017084906
210214_s_at	BMPR2	1.004664668	5.092339944	0.005281088	0.032464049
205610_at	MYOM1	-1.004602461	4.459444847	0.001102549	0.010825123
201600_at	PHB2	1.004496915	9.296723397	0.00521376	0.032201706
213000_at	MORC3	-1.00440121	5.20705855	0.005836212	0.034857167
203175_at	RHOG	-1.004066793	6.465777556	0.000197707	0.003152284
211693_at	IGH	-1.003999769	4.661686239	0.001699181	0.014713361
208813_at	GOT1	1.003726089	6.580556036	0.008619666	0.045454572
215690_x_at	GPAA1	1.003715558	6.744967031	0.009764561	0.049576426
211433_x_at	FAM214B	-1.0035734	6.266013903	4.11E-05	0.000961371
209054_s_at	WHSC1	-1.003230803	6.032639859	0.003779395	0.025780519
211260_at	BMP7	-1.002759986	5.568717112	0.00501173	0.031356384
215389_s_at	TNNT2	-1.002684744	3.880629822	0.000441988	0.00558223
53991_at	DENND2A	-1.002660255	3.855358149	0.003445798	0.024116632
211726_s_at	FMO2	-1.001936173	6.336657152	0.008363126	0.044616348
209898_x_at	ITSN2	-1.001899298	7.081437445	0.002542788	0.019497905
209785_s_at	PLA2G4C	1.001570189	5.964716995	0.00491668	0.03096902
201207_at	TNFAIP1	1.00142754	7.091243867	0.00196216	0.016259168
213326_at	VAMP1	-1.000611273	5.750334756	0.001955759	0.016228188
49679_s_at	MMP24-AS1	1.000179468	5.696324993	0.008473622	0.044943618
