# Supplementary material for: Prognostic signatures associated with high infiltration of Tregs in bone metastatic prostate cancer
Source: Aging (Albany NY). 2021 Jul 6;13(13):17442–61. doi: 10.18632/aging.203234 (PMC8312432; doi:10.18632/aging.203234)
Supplement: Supplementary Table 3 [file aging-13-203234-s004.pdf]

Supplementary Table 3. Differentially expressed genes in GSE77930.

ID_REF	Gene	logFC	AveExpr	P.Value	adj.P.Val
A_23_P411246	TMC8	4.864449913	16.32945216	7.16E-19	1.73E-15
A_23_P7313	SPP1	4.792633119	13.21698401	4.07E-11	1.57E-09
A_24_P142305	HBA2	4.552652413	11.31914095	1.76E-13	1.92E-11
A_23_P85008	MAOB	-4.432842512	10.34172249	4.44E-10	1.13E-08
A_23_P87346	HBD	4.38520456	9.48106747	2.00E-08	2.86E-07
A_24_P151295	MLLT1	-4.328902108	10.29701051	1.04E-16	5.28E-14
A_23_P344673	LOC401022	4.206326026	16.31503565	1.75E-15	4.31E-13
A_32_P216369	CCDC144A	-4.174109738	11.11413799	1.67E-14	2.86E-12
A_23_P42548	TRDN	4.164418708	14.7562026	2.63E-13	2.64E-11
A_24_P90900	CTRL	4.119632883	13.59326176	1.17E-11	5.77E-10
A_23_P300301	JMJD1C	-3.899267807	10.54947896	3.42E-12	2.10E-10
A_23_P117363	SERPINA6	-3.892017973	10.42615329	1.50E-15	3.78E-13
A_32_P229760	EIF3E	3.828795513	11.02213566	3.78E-18	5.84E-15
A_23_P37856	HBA1	3.806755388	13.16386259	5.89E-08	7.20E-07
A_23_P31816	DEFA3	3.795706298	10.13993512	5.58E-07	4.99E-06
A_24_P293192	FXYD3	3.785525304	13.40110561	3.22E-10	8.68E-09
A_23_P28969	CHMP4B	3.77554378	11.55252317	1.68E-18	3.42E-15
A_32_P180741	TNK2	-3.745142855	8.685313314	2.51E-17	1.98E-14
A_24_P83899	GRM5	3.689749371	14.71038235	2.53E-12	1.62E-10
A_24_P190472	SLPI	3.559818965	11.39247459	1.30E-06	1.04E-05
A_24_P82466	GAS7	3.547568973	12.41842833	2.60E-14	4.02E-12
A_32_P122240	ASCL5	-3.475222255	10.56918628	3.54E-16	1.34E-13
A_24_P304969	C16orf35	3.446137278	16.75681107	1.78E-16	8.21E-14
A_24_P934355	NOS1	3.438836119	15.90372913	5.56E-12	3.08E-10
A_23_P142533	COL3A1	3.430788806	12.62406051	1.76E-08	2.57E-07
A_24_P314159	APP	3.409757355	10.85895327	2.45E-14	3.83E-12
A_23_P128192	PFDN5	3.391852273	13.40702639	5.95E-18	7.34E-15
A_23_P11806	COL11A1	3.362523422	9.851348251	2.21E-08	3.11E-07
A_23_P64539	HBG1	3.345932685	8.958900508	8.48E-07	7.18E-06
A_32_P107746	ENSA	3.342048091	10.73125652	1.01E-16	5.28E-14
A_23_P309381	HIST2H2AA4	3.336744177	14.1373968	4.15E-09	7.52E-08
A_23_P330419	ACPT	3.33556934	14.94947387	7.77E-13	6.42E-11
A_24_P306425	CLIP1	-3.332354782	11.09565084	3.04E-15	7.09E-13
A_23_P8820	FABP4	3.305551706	8.996161163	1.15E-10	3.68E-09
A_23_P6963	UBE2E1	3.30205202	9.648782192	5.56E-19	1.66E-15
A_23_P96827	APCS	-3.261854734	8.633592317	4.74E-19	1.66E-15
A_23_P36795	SYT1	-3.240324236	10.87693947	0.000171524	0.000715208
A_32_P194821	RPL21	3.222840762	14.29612775	5.27E-16	1.79E-13
A_23_P168916	CA1	3.212466471	9.686978619	2.60E-07	2.58E-06
A_24_P59361	KIF16B	-3.199043747	10.2966351	3.98E-14	5.54E-12

A_23_P168551	SLC29A4	-3.195274252	10.37899039	1.25E-14	2.27E-12
A_24_P103363	FAM172A	-3.183707273	11.28551177	6.87E-17	3.90E-14
A_24_P825874	POTEE	-3.182465684	12.73623427	7.86E-07	6.72E-06
A_23_P206920	MYH11	-3.164058461	9.396130072	1.03E-05	6.22E-05
A_24_P324749	ANKRD36B	-3.154850325	10.11152652	5.11E-14	6.85E-12
A_23_P82370	HOXA6	-3.144192748	9.236354576	7.91E-17	4.31E-14
A_23_P46238	CELA2A	3.143054178	12.21515442	2.46E-10	6.95E-09
A_24_P341089	FLJ40330	-3.141478716	12.91326003	5.43E-14	7.20E-12
A_23_P359052	BOD1L	-3.136262254	11.2623661	4.75E-13	4.30E-11
A_23_P75453	MEN1	-3.120478734	11.28836872	3.91E-15	8.69E-13
A_24_P933492	ZDHHC21	-3.112008296	11.77402131	4.03E-11	1.56E-09
A_24_P320604	ARMC2	3.110670549	15.32333403	1.46E-10	4.48E-09
A_23_P203558	HBB	3.098439333	15.35907096	7.32E-06	4.62E-05
A_23_P42649	POLR2J	3.0934694	13.35038826	2.64E-16	1.10E-13
A_32_P215143	LOC100288583	-3.044086941	14.31974936	1.43E-15	3.70E-13
A_24_P929824	ITGB8	-3.039536219	6.6727219	9.23E-23	3.57E-18
A_24_P566891	ARF1	-3.039403398	13.4990992	4.34E-17	2.89E-14
A_32_P110751	EIF4A2	3.030871072	10.94315947	5.97E-17	3.56E-14
A_23_P57118	TGM3	-3.014031664	8.682044812	3.73E-14	5.30E-12
A_24_P944154	MCTP2	-3.005900063	12.64373452	1.53E-08	2.28E-07
A_24_P366390	GOLGA6L9	-2.996529196	14.01730506	1.67E-11	7.62E-10
A_23_P8558	ABHD11	2.985256217	15.48359559	2.35E-10	6.67E-09
A_24_P508103	LOC440905	-2.980556118	13.92843833	8.14E-05	0.000374744
A_24_P148261	TGFB2	-2.979354	13.1915921	1.55E-06	1.21E-05
A_23_P166269	FAM3B	2.976272539	12.44718359	0.000775301	0.002659834
A_24_P304549	LAMP1	2.972036457	9.492684858	1.02E-16	5.28E-14
A_24_P820037	SLC6A17	-2.956553269	7.678773514	1.76E-20	1.13E-16
A_24_P392723	CROCCL2	2.949289797	10.17659306	1.49E-09	3.19E-08
A_24_P93656	CAND1	-2.942320946	10.91159596	1.35E-10	4.20E-09
A_23_P50872	NDUFB7	2.937168204	12.80533353	2.85E-16	1.16E-13
A_23_P9485	ORM2	2.934810984	11.12248639	0.000537259	0.001932659
A_23_P99496	MCF2L	-2.927058939	11.52774365	5.43E-12	3.04E-10
A_23_P212974	OTUD4	-2.919811186	10.62971557	7.98E-18	8.82E-15
A_24_P131785	FAM193A	-2.917777356	10.83438214	8.65E-15	1.70E-12
A_23_P2920	SERPINA3	2.915508275	11.60000536	0.000284141	0.001110252
A_24_P329597	UBQLN1	2.913983444	10.69414986	1.62E-18	3.42E-15
A_24_P63262	RPN1	2.91184941	12.22683262	1.30E-17	1.19E-14
A_23_P52986	VWCE	2.909941066	14.82693372	2.96E-10	8.08E-09
A_24_P932680	LOC100289230	-2.901369719	11.2377987	6.81E-12	3.66E-10
A_23_P36305	ATG16L2	-2.89933377	12.38698636	1.17E-12	8.81E-11
A_23_P164057	MFAP4	-2.898594715	9.686474951	0.000354179	0.001346264
A_24_P201973	TEP1	-2.881621796	9.87936343	2.72E-13	2.71E-11
A_23_P118254	FOXF1	-2.880167333	11.05351354	4.03E-07	3.75E-06
A_23_P25463	CEP290	-2.874322484	11.92384542	4.11E-14	5.71E-12

A_23_P9465	FPGS	2.859189281	16.63237141	7.65E-14	9.58E-12
A_23_P41992	GFRA3	-2.855087867	9.677754986	5.16E-17	3.22E-14
A_23_P35564	SEC31B	-2.854897339	13.20553192	1.15E-11	5.69E-10
A_24_P829261	MALAT1	-2.85483978	16.38333898	3.32E-14	4.85E-12
A_32_P627	TAF9B	-2.820177624	12.92692519	1.09E-09	2.44E-08
A_32_P199767	ZNF630	-2.815181322	9.722873593	2.21E-16	9.44E-14
A_23_P405707	BCOR	-2.806801954	13.53434991	4.02E-13	3.77E-11
A_23_P304373	HTR5A	2.806399921	11.57239909	2.31E-12	1.51E-10
A_32_P149492	RP11-94I2.2	-2.80044279	13.02474832	3.25E-18	5.71E-15
A_24_P325533	PKLR	-2.785510432	8.756458865	1.45E-13	1.64E-11
A_24_P399622	COPE	2.783603299	11.5677811	3.97E-06	2.72E-05
A_24_P944427	SETD5	2.776518717	10.91652755	7.23E-16	2.29E-13
A_24_P119745	FN1	2.776334823	12.52765429	3.21E-09	6.08E-08
A_23_P171314	FUNDC2	2.774386295	10.59300175	1.47E-14	2.57E-12
A_23_P167293	SCOC	2.773185374	9.351288885	6.96E-17	3.90E-14
A_24_P320328	SUB1	2.771087307	13.36682618	9.07E-14	1.11E-11
gi|66792937|ref|NM_001024457.1|	PLGLB1	-2.768491999	9.618773156	0.000140644	0.000602347
A_23_P164284	CLDN7	2.76513359	13.42414712	6.62E-08	7.97E-07
A_24_P253827	AP2B1	2.762938752	11.46303303	5.97E-17	3.56E-14
A_23_P144549	IBSP	2.762037898	8.283869074	7.35E-09	1.22E-07
A_23_P420348	POTED	-2.758654893	11.03838044	3.84E-06	2.65E-05
A_24_P137501	SFRP2	-2.757773569	9.217357659	0.001814535	0.005526852
A_23_P61524	CCDC71	2.75368039	10.81081686	8.25E-16	2.55E-13
A_24_P84719	LOC344593	-2.748805685	10.00454514	1.17E-15	3.32E-13
A_23_P256540	MRPL33	2.748359443	13.34733196	5.67E-16	1.89E-13
A_23_P93169	LGSN	-2.744220273	8.782675427	3.18E-05	0.00016475
A_24_P289208	TFF3	2.742264752	9.492195001	9.91E-11	3.26E-09
A_24_P185854	DMD	-2.74185364	10.80497371	7.55E-06	4.74E-05
A_24_P380734	SDC2	2.740457437	11.24282119	5.24E-08	6.52E-07
A_23_P409626	HNRNPUL2	2.740201462	12.23626345	8.50E-18	9.13E-15
A_23_P40240	CTSZ	2.729796413	14.34327394	2.23E-11	9.66E-10
A_23_P140490	BRUNOL6	-2.72698444	9.975970887	3.08E-09	5.88E-08
A_24_P347411	POSTN	2.719724321	8.493946462	3.39E-08	4.47E-07
A_23_P217015	SET	2.713955809	10.99594195	5.04E-14	6.77E-12
A_23_P130266	LLGL1	-2.712029544	11.67823994	2.24E-14	3.55E-12
A_24_P7085	RPL29P2	2.7065369	14.29472224	1.05E-14	1.99E-12
A_23_P31602	BUD31	2.705212481	12.36048023	8.42E-16	2.57E-13
A_23_P58266	S100P	2.703390048	13.13312987	1.58E-05	8.93E-05
A_23_P305292	LOC728264	-2.702618029	9.108514884	9.58E-09	1.53E-07
A_23_P402194	BHLHE22	2.690392295	14.54016862	1.72E-09	3.57E-08
A_24_P136807	RFC1	-2.688524664	12.4944397	2.24E-12	1.47E-10
A_24_P314554	GOLGA2LY1	-2.68679838	11.16702573	3.07E-11	1.25E-09
A_23_P7483	BDP1	-2.682078028	10.25643983	1.91E-11	8.54E-10

A_32_P150142	DKFZp434L192	2.673276429	13.4431686	8.56E-10	1.99E-08
A_24_P273694	TTC23L	-2.663040289	9.53866864	1.05E-08	1.65E-07
A_24_P380679	C7orf53	-2.660135216	12.30924845	4.00E-13	3.76E-11
A_23_P4014	SMG6	-2.660040283	9.784794821	3.71E-14	5.30E-12
A_24_P340813	SFRS18	-2.653156417	15.41792066	3.69E-16	1.38E-13
A_23_P93180	HIST1H2BC	2.63892033	12.48767136	9.44E-09	1.51E-07
A_23_P3767	NFAT5	-2.63771402	11.03473431	1.43E-13	1.63E-11
A_24_P100228	XBP1	2.63470496	10.81689528	2.23E-11	9.66E-10
A_24_P219785	CALM3	2.626562975	11.06066651	1.35E-13	1.54E-11
A_23_P151198	PDAP1	2.617175812	11.25269133	6.26E-16	2.05E-13
A_24_P101629	FAM127B	2.609149103	10.19598725	8.90E-15	1.73E-12
A_32_P489920	MTX3	-2.597450142	9.209767838	1.21E-10	3.85E-09
A_23_P123645	UGCG	-2.592128625	12.79525107	4.56E-13	4.14E-11
A_24_P341279	TRAK1	-2.589678811	9.577737317	2.22E-14	3.53E-12
A_32_P211080	HERC2P2	-2.583443895	13.52593872	8.35E-14	1.04E-11
A_24_P64241	ANKRD20A2	-2.579079421	10.29091184	9.93E-09	1.58E-07
A_24_P258473	SMOC1	2.578123189	9.033262132	2.16E-05	0.000117686
A_24_P204244	ANXA2P1	2.577555591	11.30821102	1.84E-10	5.39E-09
A_23_P35230	CD46	2.574801055	10.93156018	9.86E-11	3.25E-09
A_23_P127033	ECHDC3	2.571870726	13.09158338	0.000124808	0.000542329
A_23_P150827	LRP6	-2.56976956	11.54053131	1.67E-14	2.86E-12
A_24_P323628	RPL23AP53	2.56939104	14.91306053	2.76E-14	4.19E-12
A_24_P63950	AP1S1	2.567755619	9.258397106	1.24E-19	5.35E-16
A_23_P402081	HIST1H2BN	2.566473728	14.11490379	3.40E-10	9.08E-09
A_24_P19410	CBX7	-2.565803918	14.08590082	1.94E-11	8.69E-10
A_23_P362148	DNER	2.565529278	8.788664918	0.000591493	0.002103274
A_24_P333644	SYCP2	-2.561382636	8.335452267	2.06E-09	4.17E-08
A_23_P58132	RHOH	2.559904996	14.38500286	3.51E-10	9.33E-09
A_24_P904484	LOC283174	-2.559363759	11.05793122	1.47E-06	1.15E-05
A_32_P91250	UBE2L3	2.557879136	12.42924346	8.98E-14	1.10E-11
A_24_P290999	NEAT1	-2.553350708	14.44704296	2.90E-07	2.83E-06
A_23_P40470	HIST1H2BE	2.551827077	13.39606603	3.99E-09	7.30E-08
A_23_P40174	MMP9	2.55113147	11.36159071	3.83E-07	3.59E-06
A_24_P75376	ZC3H13	-2.550685062	11.13684577	1.05E-11	5.31E-10
A_23_P31064	MOXD1	-2.549614967	9.99373233	3.10E-06	2.20E-05
A_23_P155596	FMO3	2.546916601	8.495225118	1.41E-09	3.05E-08
A_24_P185421	PNMAL1	-2.545887657	12.5645455	1.81E-08	2.62E-07
S3989049	IGL@	2.545249764	12.46888226	0.000315379	0.001218044
A_24_P555791	HERC2	-2.543734216	10.85538394	1.03E-12	7.95E-11
A_23_P373100	MGC24103	-2.541744516	10.07210534	1.59E-07	1.69E-06
A_24_P194845	RAC1	2.53709881	13.38412874	3.66E-17	2.62E-14
A_23_P105691	GPRC5D	-2.536050832	8.382160412	2.55E-13	2.60E-11
A_23_P29800	CLDN11	-2.535636111	10.2519262	2.03E-13	2.14E-11
A_23_P26358	SMG1	-2.533122052	14.07760333	8.73E-11	2.96E-09

A_23_P434809	S100A8	2.531673394	11.86250472	0.000418034	0.001555668
A_24_P113572	PHF21B	-2.531302701	10.57715793	0.000580268	0.002068492
A_23_P36445	TMED2	2.530737063	11.69735868	1.25E-12	9.23E-11
A_23_P167997	HIST1H2BG	2.529767841	12.53360907	5.00E-09	8.74E-08
A_23_P407840	FNDC1	2.5288668	8.588737319	1.50E-07	1.61E-06
A_24_P400997	SMCHD1	-2.528587888	9.980119464	1.48E-09	3.17E-08
A_23_P346265	GNPTAB	-2.527423233	13.17746184	1.94E-08	2.78E-07
A_23_P31407	AGR2	2.526418874	13.35397711	0.001322749	0.004207496
A_23_P254212	RPA4	-2.526067829	12.74560632	1.37E-09	2.97E-08
A_24_P363953	RAB11FIP2	-2.525465826	11.06975089	8.58E-12	4.45E-10
A_24_P16036	C2orf79	2.523201259	13.09727382	4.28E-13	3.95E-11
A_24_P187787	SETD2	-2.521227605	11.77337217	2.51E-11	1.06E-09
A_24_P324405	ANKRD11	-2.521070827	13.97538689	5.81E-11	2.09E-09
A_23_P13701	TMBIM4	2.521017479	12.02619928	1.15E-14	2.12E-12
A_24_P330971	EIF3K	2.520084177	13.75862293	4.47E-14	6.16E-12
A_23_P214444	TAP2	-2.519055092	8.890098107	3.91E-17	2.69E-14
A_23_P156025	IRX2	-2.518884075	13.22881192	0.000484165	0.001765598
A_23_P23048	S100A9	2.513723674	9.494178039	1.60E-06	1.24E-05
A_32_P112623	LOC100293193	-2.510819128	11.71490507	1.49E-09	3.19E-08
A_24_P278684	DFFB	-2.510786738	9.92787675	1.65E-11	7.54E-10
A_24_P41629	RPL21P44	2.510525214	12.06135046	1.44E-12	1.04E-10
A_32_P16854	ANAPC13	2.509580345	12.22034416	1.02E-14	1.95E-12
A_24_P188183	MPHOSPH8	-2.507436072	11.59765609	1.43E-09	3.09E-08
A_23_P57950	RHO	-2.505938378	12.52747098	6.15E-19	1.66E-15
A_32_P85273	GOLGA8F	-2.502521347	11.24251776	2.18E-09	4.38E-08
A_24_P137376	ATP2C1	2.496941822	11.97477023	1.30E-12	9.54E-11
A_23_P382302	RIF1	-2.484945274	9.440824205	5.19E-10	1.29E-08
A_24_P75917	CCDC144B	-2.484731057	9.480014228	3.04E-09	5.81E-08
A_23_P254442	ZNF783	-2.484565551	12.40810204	1.89E-09	3.86E-08
A_23_P70127	TMED9	2.482122055	12.45057601	1.41E-15	3.65E-13
A_24_P168416	PRDX2	2.479424729	11.37967594	3.27E-16	1.28E-13
A_24_P136454	TNS1	-2.477873965	11.06410726	1.09E-14	2.04E-12
A_23_P148519	FAM156A	-2.475209964	15.01496207	4.75E-15	1.03E-12
A_24_P143440	DYNLRB1	2.473751095	12.20473309	1.20E-17	1.14E-14
A_24_P684119	LOC100130288	2.470720944	8.875617352	5.33E-13	4.70E-11
A_24_P332341	POLR2J4	2.470008814	11.50940485	3.28E-16	1.28E-13
S4622413	LOC400986	-2.467724506	14.20685326	5.99E-12	3.29E-10
A_23_P69226	TMEM111	2.467544034	11.16080494	1.37E-17	1.23E-14
A_24_P117964	BOD1	2.462813493	9.508494446	6.73E-17	3.89E-14
A_23_P393015	MYL10	2.458267176	12.45246854	2.16E-11	9.48E-10
A_23_P45934	SRP9	2.456397808	11.41643948	6.93E-13	5.82E-11
A_24_P131589	CD86	-2.450847104	12.36700382	2.25E-06	1.66E-05
A_24_P522631	TMEM201	-2.448533107	13.48917064	1.18E-19	5.35E-16
A_24_P307759	SYNE2	-2.447597363	10.28352574	7.34E-10	1.75E-08

A_24_P753331	LOC388889	2.447409644	12.57912363	3.23E-11	1.30E-09
A_24_P190541	BRWD1	-2.44059226	11.24939239	6.86E-11	2.43E-09
A_23_P319005	HMP19	2.436243406	13.67042527	1.54E-09	3.27E-08
A_23_P108994	RPN2	2.430030643	11.6234802	1.08E-15	3.15E-13
A_23_P164228	ATP5G1	2.425771858	13.33369406	4.89E-13	4.39E-11
A_24_P174550	RHOA	2.425395631	11.25584299	3.58E-14	5.17E-12
A_32_P82424	LOC647252	2.423826867	10.53855232	1.47E-12	1.06E-10
A_23_P168610	TSPAN13	2.42330056	12.20391722	2.88E-11	1.19E-09
A_23_P83388	EPPK1	-2.422818279	12.88898059	1.74E-06	1.33E-05
A_23_P301925	LOC100288578	-2.420945558	13.96163806	1.63E-08	2.40E-07
A_23_P329835	UTY	-2.419762096	9.189009096	8.03E-08	9.42E-07
gi|58219001|ref|NM_001010888.1|	ZC3H12B	-2.41901769	9.280699335	8.56E-05	0.000391351
A_23_P421513	LOC220594	-2.419006571	11.48973316	5.08E-08	6.35E-07
A_23_P215459	ELN	-2.41568976	9.364753951	2.75E-05	0.000144764
A_23_P60627	ALOX15B	2.414299669	10.41454652	0.009297142	0.02245929
A_23_P41114	CSTA	2.39845711	8.495695691	4.41E-06	2.99E-05
S31784172	RGPD3	-2.395932186	14.69326427	7.63E-14	9.58E-12
A_24_P309317	PSAP	2.394770956	11.52790121	1.40E-15	3.65E-13
A_23_P68106	TMSB10	2.394506438	15.03551719	4.33E-10	1.11E-08
A_23_P152570	TOM1L2	-2.393484394	13.75401457	3.12E-10	8.45E-09
A_24_P228611	FER1L4	2.39235533	12.21535135	5.39E-11	1.97E-09
A_24_P215653	CLEC14A	2.391847303	12.09011295	5.89E-12	3.24E-10
A_24_P359165	SWAP70	-2.391478495	10.95309062	7.03E-12	3.74E-10
A_23_P427472	FAM193B	-2.387391093	8.820723922	1.81E-06	1.38E-05
A_23_P71598	MPDZ	-2.385036858	13.06222315	4.79E-18	6.86E-15
A_23_P18806	YIPF5	2.383239177	10.60673895	1.77E-18	3.43E-15
A_32_P136776	LOC644192	-2.38186	10.48121128	2.07E-11	9.19E-10
A_23_P216167	PSD3	-2.378868589	12.30068625	3.20E-08	4.25E-07
A_32_P9753	TMEM49	-2.378446167	9.872298444	1.32E-08	2.02E-07
A_24_P693448	ZNF552	-2.377703225	9.60827024	1.90E-06	1.44E-05
A_23_P117190	DNAJC15	2.377577642	11.1890964	2.72E-07	2.68E-06
A_24_P652033	DPY19L1P1	-2.368007547	10.26211625	4.48E-09	8.01E-08
A_24_P102203	UBR1	-2.367320098	10.37807739	8.79E-14	1.08E-11
A_24_P235266	GRB10	-2.365601576	15.12861116	6.76E-12	3.64E-10
A_32_P119569	GOLGA8E	-2.364723622	10.36178797	4.56E-10	1.15E-08
A_24_P42264	LYZ	2.363525942	8.449024139	1.83E-12	1.26E-10
A_32_P195850	DPY19L2	-2.362357981	11.51072961	1.96E-05	0.000107959
A_24_P203630	ANKRD36	-2.36200059	14.77493396	1.06E-11	5.33E-10
A_24_P595877	SFRS12	-2.35711564	10.39508042	3.92E-14	5.49E-12
A_23_P382654	ASCC3	-2.356162546	10.27552666	5.41E-11	1.97E-09
A_23_P387000	XKR6	-2.354786467	8.952142077	1.52E-06	1.18E-05
A_24_P335656	SECTM1	-2.354171916	11.96503676	5.21E-12	2.93E-10
A_23_P63655	ATP5C1	2.353167963	11.54038729	5.37E-11	1.96E-09
A_32_P31618	GSR	2.351772729	10.41045688	2.12E-14	3.44E-12

A_24_P371670	HNRNPA0	2.350904469	10.44194095	2.62E-14	4.04E-12
A_24_P573978	CYCS	2.348863994	12.68194507	7.84E-11	2.70E-09
A_24_P260101	MME	2.348192265	10.03710647	4.21E-06	2.88E-05
A_23_P83414	PPP1CB	2.345104566	11.38959655	4.28E-09	7.69E-08
A_23_P415633	FLJ25006	-2.342650023	11.76243171	1.17E-13	1.35E-11
A_23_P83556	CRK	2.341302678	9.685062603	1.26E-14	2.29E-12
A_24_P161018	PARP14	-2.340571549	12.11027205	1.34E-08	2.04E-07
A_24_P136161	HNRNPCL1	2.337774453	9.78739001	5.26E-15	1.13E-12
A_24_P113131	BZRAP1	-2.334998973	10.41162133	1.60E-08	2.36E-07
A_23_P311608	GOSR2	2.334193143	9.279031487	4.67E-18	6.86E-15
A_23_P65240	COL4A1	2.33398326	13.13008208	7.17E-08	8.53E-07
A_23_P122210	FLJ11292	-2.330784605	14.22086062	3.47E-10	9.22E-09
A_23_P149649	SDHB	2.329402544	12.97904908	5.71E-12	3.16E-10
A_24_P289260	DACT2	-2.326639533	9.603049225	0.008760018	0.021326847
gi|46409495|ref|NM_207436.1|	FLJ42957	-2.324058912	11.38630564	2.34E-07	2.35E-06
A_23_P30163	FLJ13197	-2.317109769	12.10483103	1.32E-08	2.02E-07
A_24_P398319	TMEM9B	2.31444313	10.27492599	5.72E-14	7.51E-12
A_23_P301133	IP6K2	-2.314101131	14.2053989	9.10E-11	3.06E-09
A_23_P77223	MESP1	2.312516544	13.30667021	1.16E-05	6.89E-05
A_23_P8497	GHRHR	2.311547387	13.09342775	7.77E-09	1.28E-07
A_23_P256148	AKIRIN1	2.310980172	11.05456159	3.60E-10	9.50E-09
A_23_P10559	AATK	-2.310944286	12.4915514	5.20E-08	6.47E-07
A_23_P18055	C3orf51	-2.310919403	12.03565664	2.96E-11	1.21E-09
A_23_P84587	WNK1	2.308674913	9.224300898	1.40E-19	5.41E-16
A_24_P827037	LRRC15	2.308567128	8.231736732	4.38E-08	5.57E-07
A_23_P69810	AGPAT9	-2.305912678	10.18120545	1.59E-10	4.77E-09
A_32_P119616	ETFA	2.30512734	11.21883133	3.95E-13	3.73E-11
A_23_P213745	CXCL14	2.304238181	9.274212456	0.001038796	0.003420123
A_24_P944144	DIDO1	-2.302015235	12.54844386	2.42E-12	1.57E-10
gi|34147618|ref|NM_138818.2|	PCA3	-2.30008432	13.17352136	0.000121378	0.000529684
A_23_P305210	ZNF680	-2.299619721	10.87840886	1.98E-09	4.04E-08
A_23_P320185	NDUFA11	2.298400718	14.05732167	2.49E-17	1.98E-14
A_23_P323180	HOXD3	-2.298347007	10.42145079	1.77E-05	9.85E-05
A_23_P112260	GNG10	2.297594613	11.76081006	2.30E-11	9.92E-10
A_24_P566764	LOC162632	-2.293801697	9.440758821	3.66E-07	3.46E-06
A_23_P139471	RPS26	2.292518388	9.023170675	1.02E-16	5.28E-14
A_23_P335143	SNRPN	-2.291920659	9.133189667	2.83E-09	5.46E-08
A_23_P306234	SNRNP48	-2.288819348	12.20384729	1.34E-09	2.91E-08
A_24_P937119	ZNF292	-2.287908299	11.84983555	2.83E-08	3.84E-07
A_32_P166480	C9orf93	-2.287404447	8.470708919	7.64E-10	1.81E-08
A_32_P54616	KIAA1407	-2.28666649	11.2155174	1.23E-07	1.36E-06
A_23_P200199	TXNDC12	2.285953193	10.77650011	2.06E-17	1.69E-14
A_23_P324107	RORC	2.285244801	9.744156302	2.83E-06	2.03E-05
A_23_P402164	LOC142937	-2.285244394	10.05813367	1.76E-12	1.21E-10

A_32_P196483	RPS3A	2.283857735	14.07471588	1.68E-09	3.50E-08
A_24_P271285	NACC1	-2.28176441	14.15684923	3.05E-17	2.36E-14
A_32_P133670	ANP32A	2.281408222	9.648187219	1.93E-16	8.60E-14
A_23_P133153	DMP1	2.280405029	7.828384434	2.20E-07	2.24E-06
A_23_P379159	BRWD3	-2.278796562	9.961337872	4.26E-11	1.62E-09
A_24_P16541	NBPF20	-2.273371171	13.31469438	7.19E-12	3.82E-10
A_23_P146233	LPL	2.268593106	11.76845126	9.52E-05	0.000428925
A_24_P380991	RUFY2	-2.267901395	12.03372526	1.10E-12	8.32E-11
A_24_P153643	DOCK3	-2.267717388	8.95154998	0.000324506	0.001247938
A_24_P230916	MIER1	-2.266893957	10.97957044	2.43E-12	1.57E-10
A_24_P921584	ALDOA	2.266514717	12.1885992	6.26E-19	1.66E-15
A_23_P103698	ROBLD3	2.265267167	13.90348123	2.29E-12	1.50E-10
S34543730	STARD9	-2.264139794	10.37083295	2.59E-09	5.06E-08
A_23_P316850	ODF3L2	2.262900652	11.42913558	9.80E-09	1.56E-07
A_24_P826646	NPM1	2.262187261	12.58536266	4.34E-11	1.64E-09
A_23_P28246	SLC23A3	-2.260684479	10.7032463	1.71E-08	2.50E-07
A_32_P122136	LOC150759	-2.26060675	12.9768944	2.67E-07	2.64E-06
A_23_P23611	AMY1C	-2.258877601	11.94608074	1.56E-05	8.86E-05
A_23_P167493	CARTPT	-2.256205583	9.895269954	0.002583652	0.007493211
A_23_P259555	OR12D3	-2.254966638	7.690517212	3.75E-16	1.38E-13
A_23_P328034	C20orf96	-2.25361989	9.942082305	4.99E-10	1.25E-08
A_24_P73669	GSPT1	2.250625048	11.18169124	1.87E-10	5.46E-09
A_24_P285831	NDUFB5	2.248415972	11.8371133	4.36E-10	1.11E-08
A_24_P113521	DGCR8	-2.245550769	13.98318981	1.14E-08	1.78E-07
A_23_P381979	OGT	-2.242715718	15.70761755	2.55E-07	2.53E-06
A_23_P26124	RORA	-2.23775874	11.36668807	3.56E-08	4.66E-07
A_23_P45140	KRAS	-2.23739169	11.33997156	5.24E-11	1.93E-09
A_24_P347624	SNURF	2.237360607	13.88468129	2.03E-06	1.53E-05
A_24_P316489	NDUFA10	2.236813314	9.600194329	1.00E-11	5.07E-10
A_24_P303770	CTSB	2.235425787	11.31900618	6.69E-12	3.61E-10
A_23_P29851	LRPAP1	2.234128661	10.50471239	1.60E-16	7.64E-14
A_24_P942604	SMC1A	-2.232054302	11.66612441	4.90E-14	6.66E-12
A_24_P226198	RAD50	2.222822227	9.242652442	1.63E-17	1.37E-14
A_23_P125977	C1QC	2.222580048	8.374870526	5.52E-13	4.83E-11
A_24_P146211	HIST1H2BD	2.220704525	14.29131312	3.40E-07	3.24E-06
A_24_P418637	MACF1	-2.220390122	10.05099141	4.60E-12	2.65E-10
A_24_P84428	CACYBP	2.220205241	11.9739978	1.32E-11	6.33E-10
A_24_P237175	CST2	2.21987204	8.810357756	0.000592541	0.002105451
A_24_P235783	SF1	-2.219231047	9.93453541	8.06E-10	1.89E-08
A_23_P363647	DDX26B	-2.219049883	9.919485184	5.18E-09	9.00E-08
A_23_P217797	DDX3Y	-2.218270292	14.43493947	1.21E-06	9.76E-06
A_23_P404871	ERGIC1	2.217170988	14.34043746	3.97E-09	7.26E-08
A_23_P386331	FBXO38	-2.215757293	9.646330204	3.58E-11	1.41E-09
A_23_P392222	C9orf119	2.214524719	11.02812046	5.86E-12	3.23E-10

A_24_P59387	C8orf60	-2.211047016	10.2078739	1.09E-09	2.44E-08
A_24_P345081	NBPF3	-2.210718871	14.85974397	2.83E-13	2.80E-11
A_23_P324461	MIDN	2.210575205	8.875776399	6.34E-17	3.72E-14
A_23_P27994	TYROBP	2.210204848	13.24245187	2.47E-07	2.46E-06
A_24_P607880	IPW	-2.208955312	12.96556964	1.48E-05	8.46E-05
A_23_P403955	TARDBP	-2.206565782	13.09919648	3.79E-10	9.94E-09
A_24_P319354	SUMO1	2.205056008	11.06766153	6.10E-13	5.24E-11
A_24_P841622	RPL32P3	-2.204364171	12.37680142	3.26E-10	8.76E-09
A_23_P334218	WDR67	-2.203896998	12.68450528	4.40E-08	5.59E-07
A_23_P163782	MT1H	2.202820561	14.1763679	6.25E-09	1.06E-07
A_24_P55148	HIST1H2BJ	2.201092084	12.3425062	3.08E-08	4.12E-07
A_23_P101742	MRPL54	2.200930377	12.83829215	4.98E-16	1.72E-13
A_23_P307940	CAPZA2	2.200778199	10.83167739	9.22E-12	4.75E-10
A_23_P145824	PPP1R9A	-2.199602368	13.40140903	3.21E-08	4.27E-07
A_24_P289726	PSMD3	2.198213586	11.7741419	2.27E-21	2.93E-17
A_23_P157865	TNC	2.196794892	11.19276008	1.65E-06	1.27E-05
A_23_P100660	SERPINF1	2.191769024	11.61205338	0.000301273	0.001170458
A_23_P50331	FAM32A	2.191682258	11.80504044	8.09E-15	1.60E-12
A_32_P109214	CCDC144NL	-2.191300103	9.893473346	1.08E-09	2.42E-08
A_24_P225308	ARID4B	-2.190608653	14.29694456	5.04E-18	6.96E-15
A_23_P115286	BCL10	2.188746054	9.04830207	2.48E-14	3.85E-12
A_24_P325992	LIFR	-2.187608285	10.207309	2.01E-06	1.51E-05
A_23_P121586	HERC3	-2.187228671	10.83723565	7.93E-07	6.78E-06
S4066184	RPSA	-2.184169322	9.209721913	4.80E-14	6.53E-12
A_24_P120380	PIGY	2.18300973	12.73255437	2.49E-12	1.60E-10
A_23_P312181	TCEB2	2.182014559	15.26543657	6.07E-18	7.34E-15
A_23_P59294	RREB1	2.181907826	12.17165728	2.14E-11	9.39E-10
A_32_P22654	ALAS2	2.181241636	8.859546656	0.000155167	0.000655338
A_24_P332631	HEATR7A	-2.180641983	8.960353288	2.01E-07	2.06E-06
A_23_P414252	SNX8	-2.180324822	10.09067528	1.10E-11	5.51E-10
A_23_P403284	OTX1	-2.180005489	9.719011955	3.14E-06	2.22E-05
A_32_P22191	BBX	2.178381599	10.51080866	1.18E-17	1.14E-14
A_24_P405552	SERP1	2.178216532	10.17307841	1.88E-09	3.86E-08
A_32_P64418	DIS3L2	-2.178043355	11.88243698	1.81E-14	3.05E-12
A_23_P128337	PTMS	-2.175863614	12.88341359	9.19E-14	1.12E-11
A_23_P200073	C1orf128	2.172906402	9.782247701	1.08E-12	8.25E-11
A_32_P146579	LOC100132351	-2.172437692	10.50252273	7.71E-10	1.82E-08
A_23_P200940	PPIH	2.17108658	10.89898961	1.84E-10	5.39E-09
A_23_P165624	TNFAIP6	2.170715025	9.878848677	1.49E-09	3.19E-08
A_24_P227211	KLC2	-2.170169674	9.540446059	1.13E-14	2.09E-12
A_23_P106425	C15orf38	2.169863898	8.548193489	8.00E-19	1.82E-15
A_24_P247749	RAB21	2.168073979	9.133597551	6.25E-13	5.34E-11
A_24_P229756	LOC644950	2.167161514	15.1473921	1.22E-12	9.09E-11
A_24_P409483	DPY19L2P4	-2.166525275	10.85367196	9.50E-05	0.000428348

A_23_P204119	RBM19	-2.166097569	11.59863256	4.25E-10	1.09E-08
A_23_P67661	COX7A1	2.165298701	11.85722328	1.54E-05	8.72E-05
A_23_P146908	STX8	2.165195815	10.99859692	1.50E-20	1.13E-16
A_23_P66017	PRRT2	-2.162191072	13.75193828	0.000274077	0.001076365
A_24_P628384	ALKBH2	2.162069026	11.53472599	2.96E-07	2.88E-06
A_24_P664891	COX6A1	2.162042173	13.04697254	9.44E-10	2.16E-08
A_24_P205008	TSEN54	-2.160065761	12.43969044	4.90E-11	1.82E-09
A_24_P298224	NCKAP5	-2.158794026	9.049756699	2.12E-05	0.000115657
A_24_P808522	RPS14	2.15796679	14.99099382	4.20E-11	1.61E-09
A_24_P85026	ZCCHC7	-2.156668237	11.57777494	3.64E-09	6.77E-08
A_24_P42589	TRIM73	-2.155962209	9.882686848	3.02E-08	4.06E-07
A_24_P64100	SLC25A37	-2.155424331	14.50885953	1.01E-09	2.29E-08
A_23_P358195	GOLGA6L10	-2.155111889	12.0695594	9.68E-07	8.05E-06
A_23_P251785	NARG1L	-2.15486716	13.4537266	9.83E-15	1.88E-12
A_24_P382266	PIAS2	-2.153715222	12.25924699	8.78E-10	2.03E-08
A_24_P98277	GOLGA7	2.153656884	9.752395707	5.58E-13	4.87E-11
A_24_P213724	KLHL20	-2.153541437	8.148980069	4.22E-10	1.09E-08
A_23_P7144	CXCL1	-2.152844946	9.657229595	1.69E-05	9.50E-05
A_23_P431330	CRIPAK	-2.150067639	13.64151132	5.50E-11	2.00E-09
A_23_P253921	ZNF167	-2.149923168	9.685918246	3.88E-06	2.67E-05
A_32_P213210	LOC100129566	2.149417335	10.40791965	5.50E-17	3.38E-14
A_23_P169494	ORM1	2.148414967	11.15526435	0.011601083	0.02713558
A_24_P156911	HIST2H2BE	2.147202218	13.24160725	1.61E-08	2.38E-07
A_23_P388244	C22orf39	2.145972954	10.14256276	5.94E-13	5.13E-11
A_24_P932981	KLF6	-2.145935997	9.739409668	5.77E-10	1.41E-08
S816197	TRIM66	-2.145930741	11.87615072	7.50E-13	6.23E-11
A_24_P277934	COL1A2	2.145049197	13.95294437	0.00046994	0.00172019
A_23_P112859	CST1	2.144025057	8.915029565	0.001034529	0.003407773
A_32_P183970	C15orf62	2.143815014	11.88167216	3.91E-10	1.02E-08
A_24_P10890	PRR5	2.141453146	12.41345171	2.73E-11	1.14E-09
A_32_P192922	LOC157562	-2.138969078	12.64351224	1.06E-06	8.71E-06
A_24_P44462	TPM1	2.138706047	11.911314	2.67E-10	7.43E-09
A_23_P149050	NCRNA00115	-2.138356939	12.13710679	2.01E-11	8.92E-10
A_24_P82200	MEIS2	-2.137973602	9.392646908	8.31E-05	0.000381444
A_24_P374918	CYTH2	-2.137564429	10.0868929	2.94E-10	8.04E-09
A_24_P153576	SHPRH	-2.135551366	12.70969206	1.14E-10	3.67E-09
A_24_P209113	NDFIP1	2.134407424	10.21018878	8.04E-12	4.19E-10
A_23_P63751	PRDX3	2.134148885	12.0231362	4.27E-12	2.51E-10
A_23_P200524	AK3L1	2.133878907	10.28863421	1.23E-05	7.23E-05
A_32_P221958	AGAP7	-2.133567314	13.02873995	3.24E-10	8.71E-09
A_24_P59220	POTEF	2.132212593	12.31577975	1.99E-12	1.34E-10
A_23_P64873	DCN	2.131799439	14.43098103	0.004415044	0.011847442
A_23_P7030	MRPL3	2.130880189	9.862341852	1.57E-16	7.60E-14
A_24_P23636	PCLO	-2.129556177	10.1531646	7.32E-07	6.30E-06

A_24_P401990	ERCC2	-2.128901891	10.32113555	1.82E-13	1.97E-11
A_23_P158096	COL27A1	-2.128064587	12.96405946	4.01E-05	0.000202281
A_23_P62070	SLC6A19	-2.127086315	10.5709682	2.33E-16	9.79E-14
A_32_P127978	IKZF2	-2.126585317	9.924028102	1.05E-10	3.43E-09
A_32_P40493	GUSBL2	-2.125278248	10.23461109	5.74E-06	3.75E-05
A_23_P146325	DDEF1IT1	-2.125275454	10.93752573	5.30E-08	6.59E-07
A_23_P151614	PSME1	2.125233802	10.90796893	1.49E-10	4.56E-09
A_24_P119702	GPR98	-2.12506378	8.949985081	1.50E-07	1.61E-06
A_24_P149766	DCAF10	2.121073728	9.765954582	3.05E-13	2.99E-11
A_24_P83379	WDFY3	-2.120948326	12.96295412	1.37E-14	2.46E-12
A_23_P122532	C6orf47	-2.120227924	12.72838568	1.54E-13	1.72E-11
A_23_P121064	PTX3	2.119626103	10.63816262	0.001083581	0.003544835
A_24_P231173	GIGYF1	2.118742589	9.926672306	1.37E-08	2.07E-07
A_24_P339272	NCRNA00188	2.117269839	12.60233044	2.30E-08	3.21E-07
A_23_P348737	NR2F1	-2.117162258	11.76897529	0.000609244	0.00215681
A_23_P18406	CAMK2N2	-2.115494211	12.35283563	8.84E-09	1.43E-07
A_23_P42811	AGR3	2.113181881	9.352368375	0.007879211	0.019462884
A_23_P107507	CBX1	-2.112441551	11.63496712	3.34E-08	4.41E-07
A_24_P347854	SYNGR2	2.111546675	11.4969057	3.47E-09	6.50E-08
A_23_P331598	IPO7	2.111521094	12.07574661	8.12E-13	6.68E-11
A_23_P406025	PRUNE2	-2.110916395	13.0234167	0.001252095	0.004012087
A_24_P941912	DTX3L	2.110181145	10.64465548	5.03E-12	2.86E-10
A_23_P165535	EEF1B2	2.108199191	14.25989446	2.60E-11	1.09E-09
A_24_P47547	RAN	2.107364518	11.78045833	2.20E-11	9.57E-10
A_23_P115792	PLEKHA1	-2.106856897	12.199319	9.79E-14	1.18E-11
A_23_P45365	COL4A5	-2.106653129	13.26324482	4.42E-06	2.99E-05
A_23_P81721	SLC25A27	-2.105820547	11.67002881	5.41E-07	4.86E-06
A_23_P1615	FIBP	2.105497341	10.35591798	1.35E-15	3.59E-13
A_24_P84008	LOC441722	2.104893072	11.7969871	3.62E-18	5.83E-15
A_23_P48596	RNASE1	2.104333954	13.60623753	2.67E-06	1.93E-05
A_23_P21409	CEP63	2.103812121	10.97652649	1.79E-13	1.94E-11
A_32_P165713	SARNP	2.101444022	12.24531694	2.63E-13	2.64E-11
A_24_P920525	SORBS1	-2.101352965	9.365554329	1.94E-10	5.65E-09
A_23_P372467	AHSA2	-2.101040536	14.67529742	1.67E-09	3.50E-08
A_23_P257672	LYNX1	2.100773598	12.0065484	4.43E-13	4.07E-11
A_23_P351556	METTL3	-2.09973016	12.44460241	2.91E-07	2.84E-06
A_23_P157299	AEBP1	2.099714249	11.51227276	0.00014855	0.000631665
A_23_P308603	SRC	-2.098606604	12.78216417	1.27E-13	1.46E-11
A_23_P256663	GALR3	2.097884006	11.8886999	2.35E-09	4.67E-08
A_23_P7976	HIST1H1E	2.097843285	13.27789302	1.90E-05	0.000105161
A_23_P432583	OAZ3	2.097739825	11.42893494	3.03E-07	2.94E-06
A_24_P38387	NDRG1	2.096649522	9.777130302	6.68E-09	1.12E-07
S16817617	LOC646080	-2.095732838	15.50746824	1.55E-09	3.29E-08
A_24_P191781	PARM1	2.095619416	9.348104542	1.62E-06	1.25E-05

A_24_P379379	CAPZA1	2.095292872	9.061650607	9.00E-18	9.41E-15
A_24_P935318	MRPS11	2.094630953	10.46144529	7.50E-17	4.14E-14
A_32_P24832	OLFML3	2.094431036	9.989118055	2.92E-06	2.09E-05
A_32_P198923	YWHAZ	2.092749356	11.11497084	1.63E-11	7.49E-10
A_32_P146286	LOC100130331	2.091856659	12.08621188	3.14E-07	3.03E-06
A_23_P151895	CILP	-2.091249529	9.19489388	5.55E-05	0.000268742
A_23_P359174	LOC100130401	-2.091234204	9.487083697	1.51E-10	4.58E-09
A_24_P931744	STBD1	-2.08863263	8.723375099	1.38E-08	2.09E-07
A_24_P142141	ABHD2	2.086736521	9.667984592	9.18E-11	3.08E-09
A_23_P76006	SERPINH1	2.086375121	10.12414405	7.64E-14	9.58E-12
A_24_P934477	CBR3	-2.084655534	9.982977059	5.08E-09	8.86E-08
A_24_P510357	LOC100293440	2.084388189	8.887196847	2.70E-05	0.000142797
A_24_P136438	ANKRD26	-2.083904405	10.77185918	7.41E-12	3.91E-10
A_32_P193218	LPP	2.082548193	9.084149046	1.51E-15	3.78E-13
A_23_P156842	EEF1E1	2.081836772	12.14710768	2.65E-11	1.11E-09
A_24_P233850	SDHC	2.080822267	11.36333561	1.49E-14	2.60E-12
A_32_P34	LOC100131581	2.07930795	13.00642591	5.42E-10	1.34E-08
A_23_P4649	APOC1	2.078909147	12.62636679	3.72E-07	3.50E-06
A_23_P92967	MOCS2	2.078704509	10.19791728	3.14E-11	1.27E-09
A_32_P107876	FRAS1	-2.078396257	12.34713537	0.000420133	0.001562877
A_32_P89371	RGPD5	-2.078145754	12.85735106	3.57E-10	9.45E-09
A_23_P206899	TMEM159	2.076328805	10.71732205	2.90E-11	1.19E-09
A_32_P167471	CLMN	-2.075509274	13.18261215	2.13E-08	3.02E-07
A_23_P39465	BST2	2.074651989	12.81996722	0.000117367	0.000515165
A_23_P102391	SLC40A1	2.07333096	11.23153518	1.80E-05	1.00E-04
A_23_P323783	LSM14B	2.073201898	10.28547689	2.42E-11	1.03E-09
A_23_P139396	C11orf73	2.07275539	13.04938961	7.47E-12	3.93E-10
A_23_P145529	PKIB	2.071943422	10.36442873	8.46E-05	0.000387565
A_23_P171366	USP11	2.070843648	16.83806304	1.74E-12	1.20E-10
A_23_P143551	ATP6V1E1	2.066150514	10.91974877	1.37E-13	1.56E-11
A_23_P200984	PTPRF	2.066114358	10.45618249	3.09E-14	4.57E-12
A_24_P841677	CXorf23	-2.065664235	9.249744248	1.35E-10	4.19E-09
A_23_P137366	C1QB	2.065454958	12.37286828	4.76E-06	3.18E-05
A_23_P217899	CCNL2	-2.065431646	11.34033584	2.61E-10	7.29E-09
A_23_P171237	ACRC	-2.064744922	11.50937907	4.39E-10	1.12E-08
A_24_P188085	ARHGEF10	-2.064352492	9.677255345	1.25E-08	1.92E-07
A_24_P414419	HADH	2.064038202	10.17148704	7.31E-13	6.08E-11
A_32_P113436	HNRNPA1L2	2.06376165	12.08371082	1.38E-11	6.55E-10
A_24_P375002	YBX1	2.063277386	12.12220247	2.05E-10	5.94E-09
A_23_P152583	ENGASE	-2.062313497	12.98597426	4.32E-12	2.53E-10
A_32_P83465	NBPF10	-2.062154314	15.15485353	6.88E-11	2.43E-09
A_24_P174613	FBXW7	-2.061723847	13.34435452	1.13E-07	1.27E-06
A_24_P255524	CALD1	-2.061542653	11.82735369	1.58E-08	2.34E-07
A_23_P152678	B9D1	2.056155175	10.69684645	1.30E-09	2.85E-08

A_23_P161197	SEC61A2	-2.055593772	11.91065945	1.05E-10	3.42E-09
A_24_P18802	VPS18	-2.054833998	13.74947621	2.25E-07	2.28E-06
A_23_P127584	NNMT	2.054811082	13.23118352	1.22E-05	7.16E-05
A_23_P134125	MAP3K5	-2.054518465	14.64964789	1.45E-06	1.14E-05
A_24_P118260	LOC100128203	2.054141157	7.916413509	1.73E-16	8.16E-14
A_32_P97496	NCRNA00174	-2.052593175	13.33431709	9.51E-09	1.52E-07
A_23_P311468	ZNF428	2.051946554	10.25384777	4.59E-15	1.00E-12
A_23_P7961	PGC	2.051455226	12.66971503	0.014171337	0.032197751
A_23_P86283	LAPTM5	2.051052597	11.32988958	2.35E-06	1.73E-05
A_23_P133068	ANK2	-2.050357115	8.623225568	6.34E-07	5.58E-06
A_24_P174641	PDLIM5	2.048718247	14.08934774	8.02E-05	0.000370017
A_23_P215913	CLU	2.048285042	11.65507225	4.89E-07	4.45E-06
A_24_P144423	PGM5	2.047723595	10.21093088	6.54E-08	7.89E-07
A_24_P915806	HNMT	2.047664581	11.03545486	2.96E-05	0.000154719
A_23_P110362	MAPKSP1	2.047427171	11.78603773	2.98E-10	8.11E-09
A_23_P417144	RAI1	-2.04652284	14.78098074	1.22E-14	2.24E-12
A_24_P290502	GCC2	-2.045318181	10.11058696	4.19E-09	7.57E-08
A_32_P71788	FKBP4	2.045167605	11.05669419	5.50E-10	1.36E-08
A_23_P30848	HLA-E	2.044883091	11.7222641	8.31E-13	6.77E-11
A_24_P34155	RUNX1	-2.044803904	11.19153318	0.00019831	0.000812195
A_23_P49674	ARHGEF15	-2.044497272	11.94191521	3.83E-16	1.40E-13
A_23_P216312	CNOT7	2.04384628	11.84480259	2.46E-13	2.54E-11
A_24_P57977	SNIP	-2.042712995	12.21228846	4.84E-06	3.23E-05
A_24_P45728	CGN	-2.04202854	12.10178448	1.57E-07	1.67E-06
A_24_P121535	ACTC1	2.041985817	11.57255013	1.33E-12	9.72E-11
A_32_P920268	LOC100287114	-2.041960411	7.692271091	4.72E-12	2.71E-10
A_23_P304450	GATA6	-2.041876146	11.31392692	2.35E-08	3.27E-07
A_23_P157361	WDR60	-2.040603757	12.32380435	3.15E-09	5.98E-08
A_24_P927716	UNQ9368	-2.040281255	9.322466199	0.000112363	0.000496392
A_24_P374834	OTUD1	2.040158148	8.7442405	2.70E-13	2.69E-11
A_32_P185481	LOC100131165	-2.039677291	9.793457899	1.10E-09	2.45E-08
A_23_P105144	SCUBE2	2.039564063	10.61079066	0.01560539	0.034973391
A_23_P434965	LIMD1	2.039420269	8.851487306	8.59E-16	2.58E-13
A_24_P311036	MYOG	-2.038867637	9.673635295	1.23E-07	1.36E-06
S16888181	LOC389765	-2.037836537	13.10510963	2.13E-11	9.37E-10
A_23_P126766	MTMR11	-2.037776004	9.458607627	3.95E-14	5.51E-12
A_24_P8575	OTUD3	-2.037343169	8.944117512	9.85E-10	2.24E-08
A_23_P331348	DOCK7	-2.036652908	8.742765856	3.91E-12	2.35E-10
A_23_P55270	CCL18	2.034564176	11.25327839	0.009406121	0.022691388
A_24_P243528	HLA-DPA1	2.033060366	9.17445116	4.85E-09	8.53E-08
A_23_P385529	POLR3E	-2.031305188	9.975766687	6.46E-07	5.66E-06
A_23_P34345	VCAM1	2.03037388	8.19980506	1.04E-08	1.64E-07
A_23_P27353	SLC14A2	-2.02991847	8.538501084	3.72E-16	1.38E-13
A_32_P49848	RHOQ	2.029093066	10.3294349	5.49E-13	4.82E-11

A_23_P156109	FAF2	-2.028852949	12.23123509	3.62E-09	6.74E-08
A_24_P7974	SLC26A6	-2.027475121	12.22359257	8.53E-16	2.58E-13
A_32_P109604	LOC100132733	-2.027221624	8.716507938	3.42E-10	9.11E-09
A_23_P20107	GSTK1	2.026512689	10.93042789	1.53E-11	7.14E-10
A_24_P372913	HNF1A	2.024886151	13.28346727	3.90E-07	3.65E-06
A_23_P12329	APH1A	2.02372928	11.89469997	3.75E-17	2.64E-14
A_24_P203502	LOC284288	2.023343089	9.665430066	3.77E-12	2.27E-10
A_23_P122007	C5orf30	-2.023106787	13.94317254	3.99E-07	3.72E-06
A_23_P13222	RCN1	2.022956734	11.78476167	7.91E-09	1.30E-07
A_24_P253003	WNT11	-2.022904578	10.6176563	0.003460048	0.009606905
A_23_P15055	HBM	2.022450856	9.818359521	3.46E-06	2.42E-05
A_23_P354827	ZNF550	-2.021830725	13.76042423	7.06E-10	1.69E-08
A_23_P19164	TTC1	2.021020159	11.42810526	4.61E-07	4.23E-06
A_23_P163546	TAF1C	-2.018764258	14.40150224	4.02E-10	1.04E-08
A_23_P118105	NDUFB10	2.018431311	14.49940993	1.10E-14	2.04E-12
A_24_P256539	C12orf47	2.018295811	10.57006304	3.95E-09	7.23E-08
A_23_P62099	MAGEC2	-2.018232852	8.266203789	0.002802417	0.008034342
A_24_P281913	MLL	-2.017551017	14.60192769	1.14E-09	2.54E-08
A_23_P16032	TRAPPC2P1	2.017318447	11.41575263	1.76E-13	1.92E-11
A_23_P326160	ZC3H12A	-2.014250512	13.45523998	5.80E-05	0.000279406
A_23_P7752	SEMA6A	-2.013980095	10.55583509	0.000236562	0.000947401
A_32_P486443	ZNF500	-2.013477778	11.44455727	1.27E-08	1.95E-07
A_23_P201619	NEK7	2.012385325	9.47944417	4.85E-15	1.05E-12
A_23_P201047	THBS3	-2.011990048	13.22897798	2.09E-11	9.21E-10
A_23_P94397	OMD	2.011135493	8.243077825	7.42E-06	4.68E-05
A_32_P834166	LOC100128843	2.010481405	13.0822801	2.63E-09	5.12E-08
A_23_P145984	TSPAN12	-2.010207951	10.85578463	0.000185385	0.00076574
A_32_P204676	FABP5	2.009302295	13.9124225	0.000423319	0.001572914
A_23_P122216	LOX	2.009141203	10.66334013	0.001072314	0.003515478
A_23_P15619	PRAC	2.009108058	14.85087422	0.000334818	0.001281736
A_32_P170454	LOC283454	-2.008978641	8.277514736	2.74E-06	1.97E-05
A_23_P86424	NCOA4	2.007069186	11.02785304	3.73E-11	1.46E-09
A_24_P928522	DST	-2.007029196	11.52191467	3.36E-10	8.99E-09
A_24_P235643	CUEDC2	2.00581746	10.53862842	1.68E-14	2.86E-12
A_24_P273972	CFH	2.005630584	7.882901635	1.33E-11	6.38E-10
A_24_P290527	ZFX	-2.005167596	9.327614331	5.24E-08	6.51E-07
A_32_P131562	COMMD1	2.004546991	10.33723405	6.40E-15	1.30E-12
A_24_P307896	GIGYF2	-2.004300882	11.7991733	1.67E-11	7.65E-10
A_23_P133739	HUS1B	-2.002787398	8.430511951	7.00E-13	5.86E-11
A_24_P346101	PRELID2	-2.002244301	9.360838858	4.16E-06	2.84E-05
A_23_P376627	D2HGDH	-2.001608374	14.00674088	1.30E-09	2.85E-08
A_23_P40039	AMMECR1L	-2.00105434	13.33299681	3.40E-13	3.26E-11
A_23_P39309	PSG9	-1.999818862	7.202689653	1.47E-17	1.27E-14
A_32_P91633	LOC100289173	1.998442551	14.17979189	1.40E-10	4.33E-09

A_23_P161644	RBM14	-1.998062324	9.408962346	2.25E-11	9.71E-10
A_23_P123242	RBAK	-1.997717709	8.920569261	5.45E-10	1.35E-08
A_23_P153616	MADCAM1	1.996706199	12.93601364	5.30E-09	9.19E-08
A_24_P164505	LOC100129396	-1.996064228	8.713858239	8.83E-08	1.02E-06
A_24_P323114	ANXA2P3	1.99604104	9.413921479	1.57E-05	8.89E-05
A_23_P202206	GSTO2	1.995598897	10.14883354	9.91E-05	0.00044495
A_24_P291814	COL12A1	1.994638681	9.598271669	0.00019019	0.000783334
A_23_P426944	PAX9	1.994014123	13.32982768	1.11E-08	1.74E-07
A_23_P435183	LRRFIP1	-1.991610527	12.38212498	2.62E-11	1.10E-09
A_23_P6196	SCAND1	1.991593139	10.72130605	1.21E-17	1.14E-14
A_24_P456723	LOC728537	-1.990256007	11.95955555	2.87E-07	2.81E-06
A_24_P323545	MYH14	-1.990231419	11.44709122	3.89E-09	7.15E-08
A_24_P87763	EEF2	1.990004033	13.5665064	8.68E-15	1.70E-12
A_24_P133933	ARHGDIA	1.989948149	10.75396752	1.08E-11	5.42E-10
A_23_P144916	GFPT2	-1.989291011	11.22778341	2.07E-07	2.11E-06
A_23_P4283	XAF1	-1.988240139	10.16945121	2.03E-06	1.52E-05
A_24_P416014	RSL24D1	1.987159352	11.33484508	1.79E-10	5.27E-09
A_23_P250196	MTDH	1.986937843	11.49517824	3.14E-11	1.27E-09
A_23_P94800	S100A4	1.985975559	11.61579438	1.25E-05	7.32E-05
A_23_P92569	WWC2	-1.985709954	12.09558519	3.89E-06	2.68E-05
A_23_P135079	SARDH	-1.985631095	12.40836413	8.76E-12	4.53E-10
A_23_P19829	PILRB	-1.985254889	15.41551188	9.11E-10	2.09E-08
A_23_P426021	SEL1L3	-1.985220013	13.84148528	1.34E-05	7.74E-05
A_23_P76071	B3GNT4	-1.984836246	8.221450486	7.27E-14	9.29E-12
A_23_P23380	ELOVL1	1.984674301	11.60835272	7.39E-14	9.40E-12
A_23_P217228	TRO	-1.984141446	10.49825299	8.63E-07	7.30E-06
A_23_P257649	RBP1	1.983383443	9.045398656	5.81E-08	7.13E-07
A_23_P17269	CCDC88A	-1.982638919	10.66419272	3.54E-06	2.47E-05
A_23_P101281	ZNF587	-1.982148187	14.5358832	2.53E-13	2.58E-11
A_23_P91468	PSMA7	1.980484835	13.69229839	1.48E-13	1.67E-11
A_24_P566701	SKP1	1.980484368	12.84415334	2.30E-08	3.21E-07
A_32_P162306	LOC440577	1.979010255	11.91915758	3.08E-11	1.26E-09
A_23_P321949	PLA2G2A	1.977165546	10.89486106	0.011690725	0.027315538
A_23_P397347	MCM9	-1.976663432	10.2805773	5.17E-07	4.67E-06
A_23_P19663	CTGF	1.976589154	9.639001986	6.96E-07	6.03E-06
A_23_P203392	ASRGL1	1.976547799	11.89552379	6.35E-05	0.000302437
A_23_P206707	MT1G	1.97616681	13.04499684	1.27E-05	7.43E-05
A_24_P854492	MIAT	-1.974401682	9.436431696	6.90E-06	4.39E-05
A_24_P132099	HMGN3	1.974196285	12.14863235	2.06E-09	4.17E-08
A_23_P92520	ANP32C	1.971389487	8.697210936	5.35E-15	1.14E-12
A_24_P65941	C21orf96	-1.97084682	8.231097387	3.08E-14	4.57E-12
A_32_P148118	LOC642838	1.970247603	9.502886824	0.003256454	0.009123117
A_32_P68050	NEK1	-1.970056418	9.270925977	7.72E-08	9.11E-07
A_24_P677783	LOC152024	1.97003374	12.03342232	6.95E-12	3.71E-10

A_24_P67096	ABCA5	-1.968406704	11.50355501	5.72E-07	5.10E-06
A_24_P800629	TMED10P	1.968374995	12.53519391	0.000225093	0.000907761
A_24_P230675	SOCS2	1.968324636	10.01564629	1.45E-05	8.30E-05
A_23_P109304	SLC5A3	-1.967747259	11.20153492	2.03E-05	0.000111629
A_24_P930088	LOC100286909	-1.966671565	11.79589253	1.40E-05	8.06E-05
A_23_P12746	MRC1L1	1.966552687	9.728286144	5.60E-06	3.67E-05
A_23_P209519	DNAJB2	1.965409017	10.78310872	9.22E-13	7.36E-11
A_23_P99579	C14orf142	1.965127483	10.19411851	4.32E-10	1.11E-08
A_23_P78092	EVI2A	1.964435625	9.500295672	1.33E-08	2.02E-07
A_24_P332314	FAM111B	-1.964406302	8.687412432	7.24E-09	1.20E-07
A_23_P94133	POP1	-1.963747324	9.415432477	2.22E-08	3.12E-07
A_24_P301954	MGC16384	-1.963356287	12.66172656	1.02E-12	7.91E-11
A_24_P74753	ATP6AP1	1.962120079	11.30508121	3.28E-15	7.51E-13
A_23_P85742	CREG1	1.961970972	12.32648603	9.18E-09	1.47E-07
A_23_P411806	SLC44A1	1.961242496	9.722331933	2.23E-10	6.38E-09
A_23_P130780	GLTSCR1	-1.961033135	14.68318784	3.89E-15	8.69E-13
A_23_P46903	CAMK2G	1.960234671	9.40865691	3.98E-16	1.44E-13
A_32_P60223	ING5	-1.959969263	14.3602928	5.55E-09	9.58E-08
A_24_P62615	CAP1	1.959946203	10.94777164	1.67E-13	1.84E-11
A_23_P160460	UAP1	1.959818532	11.78475144	2.62E-10	7.31E-09
A_32_P143048	ZFYVE9	1.95904996	8.430698394	2.11E-10	6.08E-09
A_24_P927886	GNA11	1.959028978	12.12452117	3.27E-11	1.31E-09
A_23_P251499	PCOLCE	1.956205078	9.916784157	6.66E-10	1.61E-08
A_23_P392529	C21orf81	-1.955804232	10.99170317	6.35E-06	4.09E-05
A_23_P55601	ZNF236	-1.955580233	10.86789405	3.03E-12	1.89E-10
A_24_P264211	PTMA	1.953944053	15.51564989	5.34E-09	9.26E-08
A_24_P95154	TUSC3	1.953521587	13.35890611	2.95E-07	2.88E-06
A_23_P131208	NR4A2	-1.952603063	11.45316464	4.31E-06	2.93E-05
A_24_P491087	CCT6P1	-1.952417166	11.26430063	1.34E-07	1.46E-06
A_23_P96590	GPRASP1	-1.951467251	11.04689204	5.27E-10	1.31E-08
A_32_P46981	HSBP1L1	1.951455814	11.83673783	1.78E-06	1.36E-05
A_32_P221076	ZC3H11A	1.950349399	10.44378526	4.25E-08	5.44E-07
A_23_P154526	GRB14	-1.949620064	12.16391306	5.76E-06	3.76E-05
A_24_P356453	CCDC56	1.949166659	13.41466155	7.12E-11	2.49E-09
A_23_P416314	HRASLS5	-1.948734945	14.54374994	5.44E-15	1.15E-12
A_24_P88800	COX7A2L	1.948541795	12.85482525	5.27E-11	1.94E-09
A_23_P203807	SLC26A10	-1.948355567	9.480135336	1.51E-05	8.58E-05
A_23_P206212	THBS1	1.945624032	9.091538273	4.89E-07	4.45E-06
A_23_P65254	POMP	1.945164083	13.49414224	9.71E-13	7.70E-11
A_23_P50597	FLJ21369	-1.944299687	8.545403181	4.34E-08	5.54E-07
A_23_P107214	RAB5C	1.943514983	11.83093081	2.49E-12	1.60E-10
A_23_P70849	NOS3	-1.943414178	10.29954579	1.36E-07	1.47E-06
A_24_P7202	KIDINS220	-1.94308942	12.51821641	1.05E-10	3.43E-09
A_23_P141738	SS18	1.942796729	11.08526282	4.41E-17	2.89E-14

A_23_P419051	PAPOLA	1.940640409	11.53636285	3.67E-11	1.44E-09
S16889064	DKFZP761N09121	-1.939807557	9.939665403	6.06E-06	3.93E-05
A_23_P154566	TOX2	-1.939599459	12.11549125	0.00014226	0.000608194
A_24_P147910	Septin 9	1.939272607	8.395062635	2.75E-16	1.13E-13
A_23_P138931	MMP13	1.939163242	8.144187991	1.56E-05	8.84E-05
A_23_P161837	MRVI1	-1.938567255	10.76733037	0.000185157	0.000765047
A_24_P125096	MT1X	1.938153705	14.64510717	6.08E-07	5.38E-06
A_24_P66001	UCRC	1.938100446	10.71491961	1.42E-08	2.14E-07
A_23_P407684	ZNF598	1.937568078	10.08879732	9.77E-12	4.99E-10
A_24_P293089	ZNF579	1.937310992	11.92301709	2.83E-12	1.78E-10
A_23_P42163	RPL7L1	1.936467802	11.61816241	3.20E-14	4.69E-12
A_23_P357966	PCMTD1	-1.934899296	10.62505966	1.04E-06	8.54E-06
A_23_P22200	ASB8	1.934752071	9.173995316	1.25E-20	1.13E-16
A_24_P835763	PCF11	-1.934088376	14.18121401	1.50E-10	4.57E-09
A_23_P211965	ATP6V1A	1.93302224	11.53182823	1.50E-13	1.69E-11
A_24_P376483	HLA-A	1.932634867	11.4942624	2.41E-12	1.56E-10
A_32_P117354	LIMCH1	-1.93154809	15.04708766	6.33E-08	7.67E-07
A_23_P106998	MRPS23	1.931143999	11.7705041	1.37E-08	2.07E-07
A_24_P268993	LEAP2	-1.930277197	11.39970877	1.55E-12	1.09E-10
A_23_P18739	TMEM184C	1.928932269	9.741139441	1.25E-12	9.23E-11
A_23_P37283	CGRRF1	1.928841183	9.624542891	2.88E-11	1.19E-09
A_23_P352484	DOK6	-1.92874565	11.59958079	0.000898166	0.003021347
A_23_P15727	FKBP10	1.927993481	12.68637268	0.000341955	0.001306084
A_24_P391526	MAGED1	1.927770559	13.24684787	2.04E-10	5.91E-09
A_23_P142776	EIF3F	1.927332629	12.35161058	2.34E-11	1.00E-09
A_24_P35109	CLDN3	1.927134203	11.29908405	3.02E-09	5.79E-08
A_23_P48513	IFI27	1.926737909	13.99756612	0.000428099	0.001588995
A_23_P70355	SERPINB6	1.926113998	10.44228822	2.88E-10	7.92E-09
A_23_P91350	RP5-1022P6.2	-1.926062232	13.14629813	2.80E-09	5.43E-08
A_23_P29655	C3orf14	1.925213752	10.50661509	0.007674405	0.019028635
S16818883	LOC728871	-1.924267081	15.38451263	4.83E-13	4.35E-11
A_24_P724040	SNRPB2	1.923821642	12.52130943	6.53E-12	3.55E-10
A_24_P20383	ARPC4	1.922826789	10.22008453	6.51E-14	8.39E-12
A_24_P315739	C20orf12	-1.921573354	8.814836116	5.64E-07	5.04E-06
A_24_P216294	CST3	1.921427197	12.04671015	5.84E-11	2.10E-09
A_32_P142013	RABEP1	-1.920761102	11.08561287	4.14E-10	1.07E-08
A_23_P382	ARHGEF10L	-1.919831852	13.82116539	6.67E-10	1.61E-08
A_24_P489695	FLJ46111	1.919609651	10.92152379	3.26E-13	3.16E-11
A_23_P142506	GADD45B	1.919396786	11.672643	1.23E-10	3.91E-09
A_23_P383977	SNAPC5	1.919140441	9.944050518	1.09E-12	8.28E-11
A_24_P3783	HIST1H2BM	1.918797937	14.44359983	9.85E-08	1.13E-06
A_24_P302374	CLCN6	-1.918620014	13.59893482	4.08E-11	1.57E-09
A_23_P3681	NETO2	-1.91851589	12.86082842	0.000119031	0.00052103
A_24_P456452	LOC100289949	-1.918024314	11.43398772	2.13E-08	3.02E-07

A_23_P102694	DEFB129	-1.917260041	10.26744841	1.24E-12	9.20E-11
A_24_P936122	STX16	-1.917181734	16.1020381	6.80E-15	1.37E-12
A_23_P54781	RBBP6	-1.917101745	14.7209936	1.42E-12	1.03E-10
A_32_P234459	HLA-H	1.916829313	12.44783114	1.17E-09	2.59E-08
A_23_P119907	ANKZF1	-1.916715772	11.86815211	4.65E-07	4.26E-06
A_23_P112482	AQP3	1.916088608	11.34145072	2.57E-06	1.86E-05
A_23_P309207	ZNF577	-1.915426064	10.63930158	1.09E-05	6.52E-05
A_32_P25253	ISCA1	1.915130891	11.04133759	1.52E-12	1.08E-10
A_24_P129588	GPR120	-1.913765566	11.29939143	4.10E-09	7.45E-08
A_23_P19590	EZR	1.913583808	12.57518524	1.13E-09	2.52E-08
A_24_P941322	QKI	-1.912914639	10.59283302	5.97E-10	1.46E-08
A_24_P66578	LOC100287723	1.912379673	10.6240038	0.002150032	0.006391294
A_32_P77977	UTP11L	1.912281521	9.709950352	1.27E-15	3.51E-13
A_32_P50452	SMS	1.911253168	11.73185898	6.67E-07	5.81E-06
A_23_P418477	AKAP11	-1.910786114	10.24066664	1.16E-08	1.81E-07
A_24_P272088	SPTB	-1.910660483	9.769624722	2.17E-05	0.000118191
A_23_P101573	SPACA4	-1.910048723	7.536756047	1.22E-15	3.39E-13
A_23_P85800	CD52	1.909980122	9.797385001	2.21E-05	0.000119952
A_24_P916266	ZNF175	-1.90701954	8.66409111	6.87E-08	8.21E-07
A_32_P119248	FOXD4	-1.906542925	10.84062787	3.23E-06	2.28E-05
A_24_P278637	FADD	1.906350618	9.20848664	1.50E-15	3.78E-13
A_32_P14544	RPS2	1.906243273	13.88081312	9.06E-10	2.08E-08
A_23_P119266	DNASE2	1.905685532	9.453478459	2.22E-13	2.31E-11
A_23_P151930	TCF12	-1.905498629	10.03521791	1.74E-09	3.62E-08
A_24_P944640	EPB41L5	-1.905160169	10.8710363	6.73E-08	8.07E-07
A_32_P147078	SLC8A1	-1.904828051	10.50806722	1.61E-05	9.08E-05
A_23_P25121	FKBP11	1.903839013	14.03534035	4.87E-06	3.24E-05
A_24_P714134	LOC399881	1.903352355	9.777729116	1.59E-11	7.38E-10
A_24_P279220	ATP6V0C	1.903131367	11.71452392	7.19E-16	2.29E-13
A_23_P6615	CPB1	1.90278606	11.30697883	4.04E-06	2.77E-05
A_23_P145336	LOC100294275	1.902645882	9.715385684	9.38E-09	1.50E-07
A_24_P70117	DTWD1	1.902042096	9.738937534	1.11E-10	3.58E-09
A_32_P170811	CCDC88C	-1.90197065	10.31869086	9.16E-09	1.47E-07
A_24_P166807	TPD52	1.901468342	11.56651174	4.87E-08	6.12E-07
A_24_P37589	ACPP	1.901234093	11.3914326	0.000611384	0.002163072
A_32_P108505	BTF3	1.900896644	13.70585963	2.87E-08	3.88E-07
A_24_P126393	TTC19	1.900400991	9.530528487	2.23E-09	4.48E-08
A_24_P3804	C6orf26	-1.900212444	11.80854309	6.88E-06	4.37E-05
A_24_P331655	KIAA0895L	-1.899030419	12.8720488	9.89E-14	1.19E-11
A_24_P239076	IGLL1	1.89895813	11.66045257	0.000663109	0.002318097
A_23_P137786	ADAMTSL4	-1.897242716	12.92512465	9.50E-10	2.17E-08
A_24_P303420	LOC221442	-1.897051958	9.628575191	7.71E-07	6.60E-06
A_23_P384816	SLC45A4	-1.897008223	11.37488838	3.96E-05	0.000200132
A_32_P196142	LOC100130938	-1.896972564	9.502409123	6.06E-08	7.36E-07

A_23_P251686	ADRBK2	-1.89644174	10.29553432	4.51E-10	1.15E-08
A_24_P224776	LSM4	1.895679844	11.63879583	1.18E-11	5.79E-10
A_23_P94103	SCARA5	1.895517347	10.32188703	0.000990755	0.003286239
A_23_P33894	MAGED2	1.895002897	11.34761214	8.34E-08	9.72E-07
A_23_P61466	CD163L1	1.894055932	9.079972435	1.02E-05	6.17E-05
A_32_P179526	ZBTB20	-1.893989735	13.90467761	3.39E-05	0.000173997
A_23_P214144	COL10A1	1.893426224	11.75412298	0.000332355	0.001273439
A_23_P28953	DNMT3B	-1.893199253	12.5373609	1.31E-07	1.44E-06
A_32_P72447	UBE2S	1.893116725	12.46867742	1.18E-10	3.79E-09
A_24_P79855	DKFZp566H0824	-1.893044351	10.86994272	1.36E-07	1.47E-06
A_24_P393596	C14orf25	-1.892509339	9.756327621	5.42E-05	0.000263336
A_23_P133543	KLHL3	-1.892499862	10.24702839	7.36E-05	0.000343351
A_24_P371399	C3orf58	1.891833665	10.06192252	1.54E-10	4.66E-09
A_23_P126298	FCGR3B	1.890038299	7.856695579	5.58E-09	9.62E-08
A_23_P56630	STAT1	1.889809199	10.16404439	1.75E-10	5.17E-09
A_23_P150746	DKFZp547G183	-1.889770421	10.23423972	3.56E-09	6.64E-08
A_24_P255218	MYO5A	-1.889665652	9.824247167	1.10E-07	1.24E-06
A_23_P1782	CD82	1.888542356	11.30662142	4.78E-09	8.44E-08
A_24_P408772	NAMPT	1.888506251	12.13829928	1.07E-06	8.80E-06
A_24_P920693	LUC7L3	-1.888108023	14.03630647	9.38E-10	2.15E-08
A_23_P364619	DCAF4L2	-1.887993755	9.306095751	4.60E-05	0.000227768
A_24_P290609	PDLIM2	-1.887417758	9.253859305	2.89E-10	7.94E-09
A_32_P44316	EEF1A1	1.887002594	12.8613014	1.27E-09	2.78E-08
A_24_P57367	AHCY	1.88678587	10.38568604	1.29E-10	4.04E-09
A_24_P29594	HBS1L	-1.884860908	8.880504368	3.57E-08	4.66E-07
A_24_P307785	RPAIN	-1.884524398	12.85645656	9.88E-09	1.57E-07
A_24_P177353	THOC2	-1.883896381	14.37543585	2.50E-12	1.60E-10
A_32_P169491	LOC100288367	-1.88292862	10.14438893	2.54E-09	4.98E-08
A_23_P17769	DDT	1.882764163	15.45154349	1.49E-08	2.23E-07
A_32_P206698	CKS1B	1.882657873	11.53387746	5.60E-11	2.02E-09
A_23_P70095	CD74	1.881902654	8.212622733	1.09E-12	8.31E-11
A_24_P274842	TP53TG1	1.881490062	13.03476405	1.19E-06	9.60E-06
A_32_P65943	EEF1D	1.881116413	13.23227771	1.70E-05	9.51E-05
A_24_P414733	C6orf108	1.881055063	11.03118759	3.76E-14	5.34E-12
A_23_P427768	LCE2C	-1.880330931	11.48050202	8.78E-09	1.42E-07
A_32_P135902	EIF4A1	1.880162571	12.79795981	1.25E-10	3.94E-09
A_24_P319942	SSR3	1.879393443	10.24430204	1.26E-08	1.93E-07
A_24_P182764	ATG4B	-1.879115319	14.22383506	1.87E-10	5.47E-09
A_23_P72068	GMDS	1.87878377	10.32718745	2.97E-10	8.11E-09
A_32_P355396	TECPR2	-1.878379621	12.3243279	8.72E-09	1.41E-07
A_23_P114349	XAGE3	-1.878327937	8.917654461	3.36E-09	6.31E-08
A_23_P254165	RAI2	-1.877489154	11.92408932	0.000153268	0.000648697
A_24_P362646	TXNDC9	1.877125948	10.63487437	2.58E-13	2.62E-11
A_24_P127828	VCP	1.877047851	10.07349961	2.07E-14	3.40E-12

A_24_P374154	SF3A1	1.876977042	10.30237016	1.99E-16	8.74E-14
A_32_P16315	RP11-631M21.2	-1.87566878	10.02980293	9.68E-08	1.11E-06
A_23_P54840	MT1A	1.875213466	14.54996309	1.60E-11	7.39E-10
A_24_P189516	ACADSB	-1.874880476	13.29468919	1.30E-07	1.42E-06
A_23_P46470	ERRFI1	-1.874764202	14.69472082	5.80E-06	3.78E-05
A_23_P29422	GYG1	1.87419798	9.296425304	3.67E-11	1.44E-09
A_32_P93493	PTPRG	-1.873405812	10.86626551	5.31E-06	3.50E-05
A_24_P282578	SSR2	1.873167256	12.95851386	1.14E-11	5.63E-10
A_23_P216935	C9orf45	-1.872450863	9.672245407	2.65E-07	2.63E-06
A_32_P43588	POTEG	-1.872365982	9.286998158	7.50E-05	0.0003493
A_23_P87532	CAPRIN2	-1.871999665	12.63528922	1.55E-11	7.22E-10
A_24_P406132	MAPK13	1.87192804	10.61228669	1.15E-05	6.81E-05
A_24_P289139	SH3KBP1	-1.871465964	10.10870991	1.97E-07	2.03E-06
A_24_P157156	CCDC150	-1.870326826	8.174182594	9.86E-05	0.000442786
A_32_P150086	LOC284232	-1.869900746	9.300749308	3.25E-06	2.30E-05
A_32_P461784	LOC646470	-1.868754441	9.540515241	8.07E-07	6.89E-06
A_24_P280628	VPS13B	-1.868545828	9.876917882	6.06E-09	1.03E-07
A_23_P430051	NGLY1	-1.868499453	10.85225478	2.05E-12	1.37E-10
A_24_P302802	PCCB	1.868484355	10.60158449	4.50E-12	2.61E-10
A_24_P260443	THBS4	-1.868388026	9.951135777	0.000546175	0.001959596
S4617114	LOC284408	-1.867753988	11.44387937	3.48E-08	4.57E-07
A_23_P143374	NINL	-1.867663763	12.53124568	0.000417405	0.001554073
A_23_P71741	KIF27	-1.867290642	11.86984889	1.32E-10	4.14E-09
A_24_P712562	C17orf67	-1.86686414	11.00583709	5.03E-08	6.30E-07
A_23_P45756	SYF2	1.865710091	12.98181344	8.16E-10	1.90E-08
A_24_P356592	PRKAR1A	1.864610435	10.58670157	1.55E-08	2.31E-07
A_24_P932736	HMBOX1	-1.864304883	11.16316099	6.97E-05	0.000327506
A_32_P190303	LONRF2	-1.863574501	13.35647218	5.24E-06	3.45E-05
A_23_P117387	MIA2	-1.861817078	8.48898011	0.000208975	0.000850828
A_32_P528311	RTN4RL2	-1.86044402	10.44008472	7.28E-12	3.85E-10
A_24_P941787	PRPF4B	-1.860204812	13.23418132	8.06E-10	1.89E-08
A_24_P754185	KIAA1109	-1.860132346	13.30632945	1.99E-12	1.34E-10
A_24_P384573	EWSR1	1.859727178	9.416046902	3.38E-17	2.52E-14
A_24_P388536	WDR82	1.859653299	9.015850943	1.23E-14	2.25E-12
A_23_P205531	RNASE4	1.859531694	11.67229671	0.00019169	0.000788756
A_24_P79054	TGFB1	1.85845752	8.166827479	1.84E-13	1.97E-11
A_23_P44974	MRPL13	1.857982245	13.65285347	4.40E-10	1.12E-08
A_23_P143857	LRRC3B	-1.857468628	9.630531717	3.17E-05	0.000164486
A_23_P352684	DCAF5	-1.856666799	11.91867832	5.95E-15	1.24E-12
A_23_P102037	COQ10B	1.856228993	9.552880605	2.69E-12	1.71E-10
A_23_P7896	DUSP22	1.856013389	8.489155327	3.31E-09	6.23E-08
A_24_P227993	UBE2I	-1.855985959	12.87364824	5.82E-10	1.42E-08
A_23_P118571	SOST	1.855975023	8.250429038	4.11E-06	2.81E-05
A_23_P79661	CCDC93	-1.854808248	12.86575515	2.67E-09	5.19E-08

A_32_P199301	TFDP1	1.854795008	9.465092553	6.58E-14	8.44E-12
A_24_P81900	SLC2A3	1.854696009	9.800224465	1.76E-06	1.34E-05
A_24_P219140	YY1	1.854049325	12.79645671	9.10E-13	7.29E-11
A_24_P350890	RANBP2	-1.854035491	11.27005793	1.40E-08	2.11E-07
A_23_P153480	KLK5	1.853380321	10.91007837	0.000278657	0.001091139
A_24_P17945	TSPYL1	1.852143914	8.739614022	3.63E-14	5.20E-12
A_23_P310350	SHPK	1.85199845	13.76772361	6.69E-09	1.12E-07
A_23_P426636	AHNAK	1.85156717	9.463240587	1.31E-11	6.29E-10
A_23_P111041	HIST1H2BI	1.851153889	14.93924571	1.73E-07	1.81E-06
A_24_P65803	C20orf20	1.850951694	9.380384422	1.20E-13	1.40E-11
A_23_P256618	CCDC90A	1.850434388	11.19644972	1.08E-11	5.42E-10
A_23_P148513	GNG5	1.850341057	13.36582533	8.36E-13	6.80E-11
A_24_P456944	LOC441124	-1.850310767	13.90618867	1.33E-09	2.91E-08
A_24_P307626	ATP1A4	1.849824582	9.658906367	1.42E-11	6.71E-10
A_23_P108708	RNF181	1.849717479	14.3997645	6.97E-12	3.72E-10
A_23_P115118	BMP8B	1.84925486	14.99177298	7.05E-05	0.000330762
A_24_P162482	MYST4	-1.848914563	9.456630193	9.70E-07	8.06E-06
A_23_P153418	ISOC2	1.848535069	13.01698639	3.91E-11	1.52E-09
A_32_P69149	STEAP1	1.847723878	13.71817916	0.001157226	0.003750951
A_23_P49521	SFRS1	-1.847677304	12.95105819	1.25E-07	1.38E-06
A_23_P59543	TARP	1.847609216	14.60056246	0.005276918	0.013793849
A_23_P92765	CCDC112	-1.847503195	12.38711334	1.62E-08	2.39E-07
A_32_P148058	RUNX2	1.84743631	8.370650193	5.13E-09	8.93E-08
A_32_P72940	RPL35	1.84739142	16.46521924	2.36E-11	1.01E-09
A_24_P914590	C20orf43	1.846766354	9.294744604	1.08E-14	2.02E-12
A_23_P97283	PAQR6	-1.84617715	12.05901249	4.09E-05	0.000205587
A_24_P296520	UBE2G1	1.845605676	10.48788076	2.68E-10	7.45E-09
A_23_P145863	S100A11	1.845201326	13.43773342	2.51E-07	2.50E-06
A_32_P88598	MAN1A2	1.844356207	11.24440164	4.23E-11	1.62E-09
A_24_P382401	ZNF791	1.844021616	8.804542178	1.53E-15	3.82E-13
A_23_P348979	FAM27E3	-1.842445274	11.07703564	3.21E-07	3.08E-06
A_23_P390504	FOXC1	1.842379215	11.39789109	0.000327637	0.001257729
A_23_P94365	ADCK5	-1.842368857	11.42900294	1.40E-09	3.04E-08
A_23_P250571	DMXL1	-1.84210261	14.44257241	1.78E-06	1.36E-05
A_23_P53557	LTBR	1.841957758	12.11228357	2.09E-12	1.39E-10
A_23_P158449	SLC37A3	-1.841464193	10.27160812	2.22E-07	2.25E-06
A_23_P111132	HSPA1A	-1.84129122	17.1220274	1.19E-11	5.86E-10
A_23_P21134	DDIT3	1.841271665	11.51574872	6.28E-07	5.54E-06
A_32_P140656	IL6ST	1.840025345	10.89878197	1.62E-10	4.86E-09
A_24_P394865	GOLM1	1.840024885	10.58527107	1.66E-07	1.75E-06
A_23_P387691	DIP2B	-1.839516753	10.44857413	2.75E-12	1.73E-10
A_23_P59163	ATF6B	1.838763734	10.76679365	1.25E-09	2.76E-08
A_24_P306034	ANKDD1A	-1.838368424	9.258346987	4.84E-10	1.21E-08
A_32_P31182	RPL7	1.838284271	14.62504678	4.69E-07	4.29E-06

A_24_P687305	POM121L8P	-1.838086175	10.68991878	1.15E-05	6.81E-05
A_24_P5750	KLK2	1.837946466	14.1869256	0.000556536	0.001993444
A_23_P38115	CCDC49	-1.837532651	12.31046407	5.72E-14	7.51E-12
A_23_P305335	RBM8A	-1.83745018	9.431839596	2.00E-06	1.50E-05
A_24_P144620	C6orf35	1.837221932	9.403334657	2.87E-13	2.82E-11
A_32_P112104	SMARCC1	1.836920014	11.70672198	4.59E-10	1.16E-08
A_24_P808534	DHX9	1.835850466	10.25455428	4.12E-12	2.45E-10
A_32_P139738	HERC2P4	-1.834782675	9.6325842	5.90E-07	5.24E-06
A_23_P252201	EAF2	1.834679132	10.89496623	2.48E-05	0.000132186
A_24_P537188	LOC100288215	-1.834458076	10.39362699	4.27E-09	7.68E-08
A_32_P80455	RORB	-1.833292424	11.22186709	0.01970083	0.042661795
A_32_P81173	USP34	-1.832893904	13.66390276	2.61E-10	7.29E-09
A_23_P57268	CXADR	1.832797386	11.38026067	9.72E-08	1.11E-06
A_24_P81473	GOT1	1.832748925	11.40519593	2.32E-13	2.40E-11
A_24_P203678	ACAT1	1.832367812	10.56354892	1.02E-08	1.61E-07
A_24_P488588	HNRNPA3	1.8320568	10.59805847	9.35E-11	3.12E-09
gi|118600962|ref|NM_198465.2|	NRK	-1.831963326	7.939495743	0.000415603	0.001548822
A_24_P918518	P4HTM	-1.831900775	10.06762901	4.03E-09	7.35E-08
A_32_P24372	TBC1D3B	-1.830827195	10.53075573	5.81E-08	7.13E-07
A_23_P52978	PRPF19	1.830621877	13.19303905	2.71E-11	1.13E-09
A_23_P123544	CHRAC1	1.830541873	11.06107599	1.21E-09	2.67E-08
A_23_P130689	ELOF1	1.830291348	11.00691269	1.89E-16	8.49E-14
A_23_P97309	CASP9	1.830066061	15.89764466	1.18E-08	1.83E-07
A_24_P127159	LOC346329	1.830012859	8.825787321	2.57E-15	6.10E-13
A_23_P90163	BCAT2	1.829981877	8.901532215	1.49E-12	1.06E-10
A_23_P89621	CBX4	1.829760784	9.969945295	2.27E-10	6.49E-09
A_24_P186862	CHMP5	1.829402943	10.24377696	6.96E-11	2.45E-09
A_23_P162982	DHRS4	1.829104206	11.19059845	4.14E-13	3.84E-11
A_23_P81017	ANKRD50	-1.828776817	9.723372811	1.56E-09	3.30E-08
A_24_P404628	C22orf30	-1.828748857	8.842542902	1.80E-10	5.30E-09
A_23_P166536	BRD1	-1.826129784	14.64111279	8.80E-12	4.54E-10
A_24_P50972	GOLGA6L6	-1.826088629	11.13982611	5.23E-06	3.45E-05
A_23_P39971	ERLEC1	1.825326239	12.19490136	1.90E-06	1.44E-05
A_23_P98864	GOLGA3	-1.825311407	9.438209231	1.91E-08	2.75E-07
A_24_P191664	GOLIM4	-1.824609024	11.36185101	1.25E-08	1.93E-07
A_32_P123771	ATP5E	1.823430133	15.19842916	4.24E-11	1.62E-09
A_24_P41180	RPS5	1.821883686	14.06387784	4.88E-10	1.22E-08
A_23_P139123	SERPING1	1.821815771	9.191087721	1.05E-05	6.32E-05
A_24_P159135	NEDD8	1.82149725	13.41523782	3.09E-10	8.39E-09
A_24_P205316	DNAJC5	-1.821223103	13.62626676	1.49E-12	1.06E-10
A_32_P72067	ARHGAP24	-1.820291181	9.949681205	6.52E-05	0.000309369
A_24_P237559	LNPEP	-1.820149503	9.535595287	5.01E-07	4.54E-06
A_23_P66241	MT1M	1.819497629	12.75098541	0.004157913	0.011243218
A_23_P25336	GLTP	1.819335795	11.16098366	1.06E-09	2.39E-08

A_24_P333445	MORF4L2	1.818312309	9.99497199	5.41E-10	1.34E-08
A_24_P388252	PPP3R1	1.818214644	12.41866654	2.55E-12	1.62E-10
A_23_P144959	VCAN	1.818039899	10.42528046	1.73E-06	1.33E-05
A_24_P238250	LGALS7	-1.817937377	9.663722102	1.29E-14	2.32E-12
A_24_P220786	RGS12	-1.817903294	11.61930186	1.71E-07	1.79E-06
A_23_P417891	ARHGEF7	-1.817280929	9.077098882	2.38E-07	2.38E-06
A_23_P87580	ANP32D	1.817234654	8.891622079	5.09E-16	1.74E-13
A_23_P502196	IDH3B	1.81664714	11.5051477	8.67E-10	2.00E-08
A_24_P406664	RAD23B	1.815965677	10.27021267	1.13E-13	1.32E-11
A_23_P116809	COPZ1	1.815108243	11.06435221	7.69E-12	4.03E-10
A_32_P39963	EXOSC6	-1.815055212	12.07753897	3.71E-09	6.87E-08
A_24_P51360	LARS	-1.814027227	10.78709027	7.89E-12	4.13E-10
A_32_P776626	RGPD1	-1.81395207	8.300841507	0.000552266	0.001979797
A_23_P36496	RBMS1	1.813130853	10.31470197	1.60E-11	7.39E-10
A_24_P109821	hCG_19809	1.81257961	14.39323987	2.84E-09	5.50E-08
A_24_P256219	MAF	1.811245705	8.796305717	1.09E-09	2.44E-08
A_23_P149470	NDUFS2	1.811230191	11.05723258	1.53E-12	1.09E-10
A_23_P20648	SURF1	1.810774229	11.81471866	4.32E-16	1.52E-13
A_23_P67466	PSMD8	1.810339223	13.91171753	4.54E-14	6.23E-12
A_23_P124905	NPTX1	-1.809530638	10.39713241	0.016045828	0.035802867
A_24_P65616	PVR	1.809302471	9.726238401	6.86E-12	3.68E-10
A_24_P319736	MEIS1	-1.808125964	9.692439295	7.19E-07	6.21E-06
A_24_P93353	TTC37	-1.807557078	9.663822885	4.80E-10	1.21E-08
A_24_P320545	PTK7	1.807256257	9.703032569	2.57E-10	7.21E-09
A_24_P169462	GOSR1	1.807043595	11.57920481	1.50E-10	4.57E-09
A_23_P29630	SPCS1	1.807035231	14.40990943	8.60E-10	1.99E-08
A_23_P153853	ECH1	1.806762628	12.67315236	9.10E-10	2.09E-08
A_32_P141724	COMMD7	1.805551702	10.22839077	1.20E-15	3.38E-13
A_23_P165090	NDUFS7	1.803892447	12.96647577	4.43E-13	4.07E-11
A_23_P256190	CDC42BPA	-1.80208889	13.24858711	3.58E-08	4.68E-07
A_23_P79931	ATRN	1.801637817	10.4886442	1.84E-13	1.97E-11
A_24_P397903	FAM164A	-1.801385242	10.64214526	5.71E-07	5.09E-06
A_23_P46936	EGR2	-1.800785806	10.31492085	5.75E-05	0.000277421
A_23_P217151	ZNF673	-1.800563862	12.17669034	1.51E-07	1.61E-06
A_24_P753161	BMPR2	1.800537309	9.393794927	9.36E-10	2.14E-08
A_24_P357100	RASD2	1.800308495	12.57533337	1.12E-06	9.10E-06
A_24_P860797	PAIP2B	1.800132418	12.04609385	5.75E-07	5.13E-06
A_23_P37598	NPTN	1.799969346	11.02276995	5.76E-11	2.07E-09
A_24_P933180	GGCX	1.799953711	10.32584039	5.27E-12	2.96E-10
A_23_P104323	MGMT	1.799940377	12.03302944	3.72E-11	1.45E-09
A_23_P148984	DARS2	1.799797875	11.15602526	7.42E-07	6.38E-06
A_24_P102512	CABIN1	-1.799692766	12.54015398	7.46E-09	1.23E-07
A_23_P16671	DDA1	1.799604936	10.69683844	5.42E-12	3.03E-10
A_23_P58606	CCNG1	1.798970149	11.49711278	3.47E-07	3.31E-06

A_23_P143218	ACOT8	1.798709723	11.07057094	5.00E-14	6.73E-12
A_23_P350234	UBE2NL	1.79835486	9.314095983	4.28E-11	1.62E-09
A_23_P215897	RNF19A	-1.7980957	9.5281183	7.34E-09	1.22E-07
A_23_P147995	PICALM	1.7968399	11.16328068	3.88E-10	1.01E-08
A_23_P500206	IL17RE	1.796183214	11.68004738	7.89E-06	4.93E-05
A_23_P106162	C14orf106	-1.7958939	10.75201907	9.18E-09	1.47E-07
A_24_P152094	RP11-431O22.2	1.795496613	12.60276129	2.03E-13	2.14E-11
A_24_P388632	PCGF3	-1.795403286	14.16861585	5.97E-11	2.14E-09
A_23_P251216	VPS13D	-1.795153831	12.16367106	4.61E-08	5.82E-07
A_23_P251259	GTF2H4	1.795085807	8.870312487	3.52E-15	7.92E-13
A_23_P105044	MRPL23	1.795006166	13.9202055	8.54E-08	9.92E-07
A_24_P148521	TMBIM1	1.794848108	8.828548447	2.40E-11	1.02E-09
A_32_P78491	ETV1	-1.7948123	8.102989221	0.000172529	0.000718854
A_32_P36942	C2orf60	-1.794577654	10.62626698	3.82E-10	9.99E-09
A_24_P154573	ZNF509	-1.793345604	10.20058696	2.00E-07	2.06E-06
A_24_P153800	SUPT16H	-1.793222429	9.070171145	1.01E-15	2.98E-13
A_24_P399065	NUDT16L1	1.792474443	10.43358616	1.60E-12	1.13E-10
A_32_P197870	KIAA1919	-1.792339789	12.47803311	3.52E-08	4.62E-07
A_23_P91081	EPCAM	-1.791936228	16.6611034	4.79E-12	2.74E-10
A_23_P62831	FAM176B	1.791930034	9.652492621	8.79E-13	7.08E-11
A_23_P8961	IL7	-1.791070786	9.788769242	0.000125382	0.00054464
A_32_P231302	LOC100129361	1.790861631	12.08181466	1.32E-10	4.14E-09
A_24_P28367	COPA	1.790358417	11.93094106	6.36E-15	1.30E-12
A_24_P233944	CEPT1	1.789256846	9.576210503	2.31E-14	3.65E-12
A_23_P432591	CCDC125	-1.787272139	11.98288748	9.83E-07	8.16E-06
A_32_P42574	C1orf198	1.786782421	17.01789388	1.23E-10	3.90E-09
A_23_P502174	DYNC2LI1	1.785448724	10.17155117	2.10E-09	4.24E-08
A_23_P325080	PTOV1	1.784951956	9.552379159	2.09E-14	3.40E-12
A_23_P49254	HBQ1	-1.783882564	10.17117591	2.11E-08	2.99E-07
A_32_P83776	C1orf96	-1.783737097	9.613313453	4.75E-08	5.99E-07
A_23_P252874	GPR116	1.783476631	10.09094529	6.58E-05	0.000311733
A_24_P247044	ZNF492	-1.783322952	12.09336651	1.24E-08	1.91E-07
A_24_P208737	GHRLOS	-1.783285929	11.16085618	2.03E-07	2.08E-06
TMPRSS2_Ex3	TMPRSS2	1.782918171	9.917267372	1.91E-07	1.98E-06
A_24_P367289	DDR1	1.782803319	8.901965911	9.16E-13	7.32E-11
A_23_P396981	CCDC66	-1.782643058	11.38528943	0.000666674	0.002329086
A_24_P333421	ZNF862	-1.781156289	13.94290837	1.40E-07	1.51E-06
A_23_P44244	SMARCA1	-1.78098081	14.13132182	7.04E-05	0.00033043
A_24_P924301	GALNTL4	-1.780952905	8.640885583	7.60E-06	4.77E-05
A_32_P27698	Septin 14	-1.780832656	9.628682674	6.10E-09	1.04E-07
A_23_P257164	AMT	-1.780770931	10.66643845	1.73E-06	1.33E-05
A_23_P54597	RSL1D1	1.780265784	12.54113846	4.31E-11	1.63E-09
A_23_P55779	CYP2A13	1.780190222	11.97616768	4.69E-09	8.30E-08
A_24_P13991	MLX	1.780063945	10.68648721	2.64E-13	2.65E-11

A_23_P217820	DHCR24	1.780048473	8.7484146	5.69E-10	1.40E-08
A_23_P348911	LOC100129726	-1.77874745	9.792711588	2.41E-08	3.34E-07
A_24_P161959	TMEM179B	1.778271684	12.40690172	7.00E-11	2.46E-09
A_23_P387585	CXorf50	-1.777719891	10.8327172	8.36E-06	5.17E-05
A_24_P124957	RAB11A	1.777656479	12.86676814	2.62E-10	7.31E-09
A_24_P84984	TTC3	-1.777531267	13.09993766	3.20E-10	8.64E-09
A_32_P6015	MNX1	-1.777151306	12.97603658	0.000609749	0.00215768
A_23_P30283	FAM174A	-1.77712873	13.52634296	5.10E-09	8.88E-08
A_23_P57534	DDX17	1.776886023	8.643286437	1.63E-10	4.88E-09
A_23_P332992	HIST3H2BB	1.776654326	14.67928316	1.92E-06	1.45E-05
A_23_P9443	C9orf78	1.776316472	11.57769014	7.15E-13	5.98E-11
A_23_P80362	NHP2L1	1.775535107	14.26897328	2.36E-11	1.01E-09
A_23_P204052	PCBP2	1.77542969	11.24144285	1.26E-11	6.09E-10
A_23_P2990	CEBPE	1.774700589	9.434496725	9.43E-10	2.16E-08
A_24_P13572	DLST	1.774697349	8.230329369	2.70E-14	4.13E-12
A_32_P8952	LMBRD2	-1.774049036	10.10243741	4.35E-08	5.55E-07
A_23_P52240	ADRB1	1.773929341	8.350994393	4.96E-07	4.50E-06
A_23_P167263	PHF17	1.773896831	10.18814427	6.52E-08	7.87E-07
A_23_P166633	ITGB5	1.773865243	12.63997556	3.60E-07	3.41E-06
A_32_P136402	THOC7	1.773639623	13.91058628	1.11E-11	5.51E-10
A_24_P243278	DAP	1.7736216	9.293522464	1.80E-14	3.04E-12
A_24_P229199	ATPAF1	1.773388579	10.97122715	3.56E-10	9.43E-09
A_23_P395595	FNBP4	-1.772782397	15.21076149	4.38E-12	2.55E-10
A_24_P567408	CLN3	-1.772358722	13.43868709	2.92E-08	3.94E-07
A_23_P155463	LRRC2	-1.771827523	10.65924525	0.000244474	0.000974973
A_24_P123011	YWHAE	1.771151329	10.54122161	2.14E-10	6.14E-09
A_23_P200772	ZNF644	-1.77111743	11.39913862	1.78E-08	2.60E-07
A_23_P94118	GTF2E2	1.770738687	10.61994183	1.40E-10	4.32E-09
A_23_P75903	TOLLIP	1.770376446	11.45024508	0.000156662	0.000660639
A_32_P2148	LOC643387	-1.77015716	8.425083919	1.02E-08	1.62E-07
A_23_P75790	C11orf9	-1.770156749	10.60126079	2.78E-09	5.39E-08
A_23_P137948	NENF	1.769392707	14.38841394	3.12E-09	5.94E-08
A_24_P876772	LOC100134822	-1.769069432	10.22418059	3.52E-08	4.62E-07
A_23_P150950	ZFC3H1	-1.768744782	13.5628259	1.14E-10	3.67E-09
A_23_P142574	MOGAT1	-1.767861255	8.908522422	0.001555972	0.004841846
A_23_P327013	KIAA0146	-1.767736601	14.49319403	2.13E-09	4.30E-08
S4620509	LOC286063	-1.767432074	10.2807475	9.63E-05	0.000433947
A_23_P251927	CHCHD3	1.766609463	11.55042661	1.09E-12	8.28E-11
A_24_P940831	ACSS1	-1.766312002	11.3038289	1.01E-05	6.08E-05
A_23_P22970	PIK3R3	-1.766070298	9.347922727	5.57E-07	4.98E-06
A_23_P20196	ARPC1B	1.765356287	12.12428739	1.15E-09	2.56E-08
A_24_P538403	ROCK1	-1.765243207	13.69416146	4.02E-12	2.40E-10
A_23_P62115	TIMP1	1.764740365	13.06039613	3.29E-05	0.00016952
A_24_P357551	PHF2	-1.764668096	11.00654863	6.07E-10	1.48E-08

A_24_P15563	C14orf118	-1.764367874	10.42631723	8.46E-13	6.86E-11
A_23_P53838	IRS2	1.763746948	8.47459376	8.76E-09	1.42E-07
A_23_P203488	SMPD1	1.762681465	10.01264604	1.57E-10	4.74E-09
A_24_P287785	AMDHD2	-1.761942165	13.34725418	3.92E-08	5.08E-07
A_23_P24633	THYN1	1.761612159	12.87827187	2.07E-12	1.38E-10
A_23_P121253	TNFSF10	1.761583488	13.10348108	0.000357425	0.001356869
A_24_P105794	RPL31	-1.76110129	14.82120597	3.02E-08	4.06E-07
A_24_P896381	CCDC57	1.761055971	11.87579241	1.07E-07	1.21E-06
A_32_P172803	MAP9	-1.76024924	10.9928286	1.74E-07	1.82E-06
A_23_P123582	IPPK	-1.760103606	12.10782362	1.63E-13	1.80E-11
A_32_P456537	TCP1	1.759414967	10.15471797	2.32E-09	4.62E-08
A_24_P179044	SNX9	1.758373157	8.669261049	1.68E-12	1.17E-10
A_24_P517901	HNRNPA1	1.758321279	12.06501524	3.11E-09	5.93E-08
A_24_P884376	LOC730375	-1.758310267	12.47858351	3.21E-08	4.27E-07
A_24_P721898	BTBD7	-1.756676652	13.55538097	9.46E-14	1.15E-11
A_24_P816384	UBE2QP1	-1.7566331	10.89943994	8.10E-08	9.50E-07
A_24_P348989	LILRA1	1.756549009	12.30096165	0.00236358	0.006944475
A_23_P135486	AHSP	1.75645698	8.088632847	1.06E-05	6.34E-05
A_24_P391468	ENOX2	-1.756137485	8.693297867	1.20E-08	1.86E-07
A_32_P215700	C1orf88	1.755545139	10.64902482	0.001130029	0.003671716
A_24_P162173	ANK3	1.754822706	8.981843152	6.45E-07	5.65E-06
A_24_P308051	DNAH8	-1.75436664	10.44316992	0.004343298	0.01167516
A_32_P216520	WIF1	1.754134031	11.65782974	0.011897081	0.027697344
A_24_P327050	TRAPPC2L	1.753987112	12.84562304	2.32E-12	1.51E-10
A_24_P116871	TXNL4A	1.753038239	12.7904642	9.81E-12	5.01E-10
A_32_P17765	LGR4	-1.752757973	8.290218355	2.79E-10	7.73E-09
A_23_P42042	LYRM2	1.752544626	10.97986959	1.47E-10	4.48E-09
A_23_P39131	GLTSCR2	1.752506058	11.40777247	4.18E-12	2.47E-10
A_24_P125561	SIGLEC9	1.75216361	11.67737579	9.19E-09	1.47E-07
A_24_P170538	UTP14A	-1.751136145	9.83875816	4.77E-12	2.73E-10
A_23_P425826	MRFAP1L1	1.750899892	11.42504256	4.78E-13	4.32E-11
A_24_P233995	MOSC1	1.750856476	10.75113755	1.71E-07	1.80E-06
A_23_P110184	SC4MOL	1.75082766	11.17679032	2.64E-05	0.000139874
A_24_P11965	MRFAP1	1.750553546	11.67084397	9.45E-12	4.86E-10
A_23_P164650	APOE	1.750455896	13.36581475	2.77E-06	1.99E-05
A_32_P123514	PABPC4L	-1.749824763	10.81490576	8.11E-06	5.04E-05
A_32_P122373	MSTP2	-1.748997029	11.4726511	6.89E-06	4.38E-05
A_24_P461497	LOC646048	1.748558056	10.14330187	1.38E-10	4.27E-09
gi|56606128|ref|NM_001008408.1|	RBM33	-1.74820766	13.478276	7.18E-09	1.19E-07
A_32_P137632	FBXL17	1.748121269	11.80803898	7.46E-08	8.83E-07
A_24_P321525	RERG	-1.747592358	11.20249037	6.64E-09	1.12E-07
A_23_P64280	UNQ565	-1.74747134	8.485439959	4.42E-07	4.07E-06
A_23_P94579	SNHG7	-1.747409456	11.49217021	6.43E-08	7.77E-07
A_24_P375592	PHF20	-1.747254639	10.52517863	4.08E-09	7.43E-08

A_24_P936537	LOC730658	-1.746134583	11.30639045	2.04E-07	2.09E-06
A_23_P256008	ZDHHC11	-1.744738789	14.12451012	1.68E-05	9.42E-05
A_24_P941309	ZNF224	-1.744269181	9.714764145	2.06E-07	2.11E-06
A_23_P331813	ZNF687	1.744235973	9.544248264	2.51E-12	1.60E-10
A_23_P118122	RGS11	-1.743982207	13.26115634	8.99E-06	5.51E-05
A_24_P301186	WDR89	-1.742829362	10.88641157	9.34E-13	7.42E-11
A_23_P86493	LBX1	1.742365603	11.38529867	2.71E-12	1.71E-10
A_23_P258136	MXRA5	1.742050746	9.054911133	2.36E-07	2.37E-06
A_32_P112279	CHTF8	1.742014162	10.3487234	5.44E-15	1.15E-12
A_23_P391637	TBC1D24	-1.741870838	12.9773849	1.82E-06	1.38E-05
A_24_P792130	LOC100289574	-1.741848038	11.68048755	2.69E-08	3.68E-07
A_23_P128728	ARG2	1.740618296	13.36721558	0.003744387	0.010283113
A_23_P208293	PVRL2	1.740607475	13.82597413	4.03E-08	5.19E-07
A_23_P76488	EMP1	1.740556967	13.46308321	2.84E-05	0.000149294
A_24_P167806	IDH3A	1.740277767	9.648888933	8.37E-14	1.04E-11
A_32_P123	SLC25A5P1	1.740097896	13.35794355	1.63E-06	1.26E-05
A_24_P277747	CNPY3	1.738941208	9.755600182	2.36E-10	6.69E-09
A_24_P263910	C14orf182	-1.738888808	9.829478795	1.12E-07	1.25E-06
A_23_P213424	ENC1	-1.738834856	13.62458259	1.88E-06	1.42E-05
A_24_P924462	PRKCZ	-1.738451567	11.86089983	6.69E-10	1.61E-08
A_23_P362228	C1orf213	-1.738199749	11.50418496	1.19E-07	1.33E-06
A_23_P120125	COLEC11	-1.738087808	11.33453607	0.008557567	0.020897075
A_24_P200652	C6orf62	1.736923755	10.80219709	8.70E-13	7.03E-11
A_23_P83781	CYTH1	-1.736913291	15.13700723	6.62E-13	5.62E-11
A_23_P60793	NCRNA00105	-1.73661078	9.754672512	8.74E-07	7.37E-06
A_23_P386320	MFI2	-1.736488148	9.149983243	1.26E-06	1.01E-05
A_23_P142872	TCF7L1	-1.736409137	8.333235965	2.71E-05	0.000143318
A_24_P188116	ANKRD2	1.736324446	14.28846873	9.24E-10	2.12E-08
A_24_P237870	SMPD4	-1.736305262	15.91897265	5.15E-11	1.90E-09
A_23_P33356	ADAMTS9	-1.736026112	9.163075314	4.86E-06	3.24E-05
A_23_P112201	KDM4C	-1.735944807	13.61209881	4.23E-12	2.49E-10
A_23_P26905	POLG2	-1.735734142	13.5163944	2.41E-08	3.34E-07
A_24_P544543	CAPN1	-1.735246677	13.08306664	2.54E-09	4.99E-08
A_23_P257716	CYP51A1	1.735209381	12.82478419	1.69E-06	1.30E-05
A_23_P2801	ELF1	1.734739439	10.75643329	3.16E-11	1.28E-09
A_23_P205855	ARIH1	1.734404953	11.00441875	2.04E-12	1.37E-10
A_24_P295745	HSPA8	1.734165103	11.77958363	2.68E-10	7.45E-09
A_23_P134085	CNKSR3	-1.733694848	14.18553333	0.000196114	0.000804393
A_24_P22050	RAB20	1.733550816	9.511565442	2.89E-13	2.84E-11
A_23_P425880	TRIO	-1.733293342	11.83995636	2.46E-11	1.04E-09
A_23_P124742	CHKA	-1.733118724	15.93060442	2.23E-11	9.65E-10
A_24_P222655	C1QA	1.733022957	11.00938352	3.69E-05	0.00018808
A_23_P30050	SLC30A9	1.73289516	10.77748352	7.21E-08	8.57E-07
A_24_P22163	DICER1	-1.732765293	12.79500955	1.77E-08	2.57E-07

A_32_P60065	F2RL2	-1.732204214	8.006603706	7.71E-05	0.000357521
A_24_P714618	LOC100288109	-1.731860646	9.152434989	0.000237108	0.00094929
A_23_P34537	EPHX1	1.731497047	10.85605345	3.01E-08	4.05E-07
A_24_P183292	CSNK1A1	1.731088652	10.89438604	2.17E-11	9.50E-10
A_24_P143492	BCAS4	1.730980039	9.121473988	2.71E-10	7.53E-09
A_23_P122439	BTN2A2	-1.730586806	10.57894027	4.28E-07	3.95E-06
A_23_P365610	JUND	-1.729393356	14.63142892	1.11E-15	3.20E-13
A_23_P212126	COLQ	-1.729053152	9.075069397	3.55E-06	2.48E-05
A_24_P417784	POM121	-1.728880323	14.41356789	1.20E-11	5.86E-10
A_23_P303810	CRYBG3	-1.728721718	13.47382533	2.38E-09	4.72E-08
A_24_P762886	KIAA0485	-1.727897932	11.71956305	2.21E-07	2.24E-06
A_24_P177236	CABP7	-1.727760843	10.43492395	0.003415716	0.009503246
gi|61966772|ref|NM_001013659.1|	ZNF793	-1.727611506	12.53902226	1.76E-08	2.57E-07
A_23_P109442	HPS4	-1.726887154	14.41561096	2.29E-08	3.20E-07
A_23_P121037	PODXL2	1.726664884	9.011465846	4.12E-08	5.29E-07
A_23_P43049	DCTN6	1.726549391	12.21075168	6.56E-12	3.55E-10
A_23_P74547	CD53	1.726294937	8.949256995	8.87E-06	5.44E-05
A_24_P141005	SYPL1	1.726226339	11.43433098	1.19E-08	1.85E-07
A_23_P129492	SEPX1	1.726012535	11.09599541	2.90E-10	7.96E-09
A_24_P332081	JAKMIP3	-1.725878175	10.52923602	0.001397837	0.004411853
A_23_P100127	CASC5	-1.7252636	8.966473248	1.69E-06	1.30E-05
A_32_P53486	BOLA2B	1.725233902	14.57479785	3.56E-09	6.65E-08
A_23_P78517	SFRS16	1.725203578	9.657142404	7.57E-12	3.97E-10
A_23_P254522	COL4A4	-1.724968394	8.55546464	3.37E-05	0.000173257
A_23_P393645	ADAMTS13	-1.724185755	11.19452554	3.40E-07	3.24E-06
A_23_P27472	ATP1A3	1.723942802	11.46911724	6.54E-10	1.58E-08
A_32_P44762	CCDC72	1.723940637	16.02058014	1.11E-13	1.30E-11
A_23_P112078	MFHAS1	1.723546623	9.114272604	1.90E-12	1.30E-10
A_23_P307400	CCDC45	-1.723541773	15.0402695	3.67E-11	1.44E-09
A_24_P254789	COL14A1	-1.723428524	8.166052935	0.000235779	0.000944946
A_23_P126939	RAB7L1	1.723022747	9.777154448	7.70E-10	1.82E-08
A_24_P137897	IFRD1	-1.722950992	13.99166118	2.20E-08	3.10E-07
A_24_P221968	LOC643802	-1.722858543	10.19078507	8.31E-09	1.35E-07
A_24_P228978	C1QTNF3	-1.72275994	8.921466244	1.12E-07	1.25E-06
A_23_P50638	LRG1	1.722586988	10.75103847	0.001096239	0.003579658
A_23_P86037	UBE2Q1	1.722532978	10.40377564	6.93E-13	5.82E-11
A_23_P47709	FOLR2	1.720565099	10.41140033	9.12E-06	5.57E-05
A_24_P943484	AHI1	-1.720494814	11.52436331	6.75E-08	8.10E-07
A_23_P204511	SLC48A1	1.720112233	14.85443123	4.26E-07	3.94E-06
A_32_P216872	LOC729602	-1.719976007	12.64743926	1.56E-07	1.66E-06
A_24_P228667	MRPL40	1.719944168	12.29736911	1.25E-10	3.94E-09
A_23_P96087	H1FX	1.719598428	11.81687509	6.52E-09	1.10E-07
A_23_P250042	SELT	1.719497371	12.94446543	1.62E-13	1.80E-11
A_32_P94866	LOC440983	-1.719473745	12.97694081	1.27E-10	4.01E-09

A_24_P28578	EPS15	1.719397719	9.815205288	1.02E-10	3.34E-09
A_23_P211550	RBX1	1.71931706	13.9398389	3.06E-13	2.99E-11
A_23_P6099	PLCB1	-1.718415436	10.63462633	1.39E-06	1.10E-05
A_23_P135271	B4GALT1	1.717965917	9.707947475	2.35E-09	4.67E-08
A_32_P134056	DOCK4	-1.717784939	10.85220381	2.52E-06	1.83E-05
A_23_P318262	DLX5	1.717341256	7.909965585	1.47E-08	2.20E-07
A_23_P319050	ZNF493	-1.717214555	8.911158156	5.43E-08	6.73E-07
A_32_P41035	LOC100133161	-1.716655529	12.13329732	5.34E-08	6.62E-07
A_23_P167168	IGJ	1.716637729	9.689747338	0.000628936	0.002216457
A_24_P342944	CSPG5	1.716270172	8.620440709	4.42E-09	7.90E-08
A_23_P89780	LAMA3	-1.71536271	12.23421389	0.005618746	0.014570258
A_24_P316414	LOC100289058	-1.713797304	9.505262572	2.04E-06	1.53E-05
A_23_P14537	TMED10	1.713478514	14.23587668	5.95E-10	1.45E-08
A_24_P99244	FGF7	1.713433811	7.99567552	8.01E-05	0.000369779
A_23_P32861	NMD3	1.712443448	11.28103326	9.36E-09	1.50E-07
A_32_P60185	SDHD	1.71203463	12.2382256	3.28E-09	6.18E-08
A_24_P107208	CAPRIN1	1.711552304	8.914920362	6.16E-13	5.28E-11
A_23_P17914	PNPLA3	-1.710980659	10.06714317	0.000103946	0.000463547
A_24_P387514	LRP5L	-1.710496987	10.84711196	1.58E-09	3.34E-08
A_24_P229377	RYK	1.709273596	10.11196219	1.70E-08	2.50E-07
A_32_P176550	JMY	-1.708695742	11.31838308	1.11E-07	1.24E-06
A_24_P194886	EHBP1	-1.708473674	11.0904179	2.67E-08	3.66E-07
A_23_P107206	STAT3	1.707903969	11.95149911	8.17E-12	4.25E-10
A_24_P247608	PCMT1	1.707622772	10.68179273	2.76E-10	7.63E-09
A_24_P393811	TMCO1	1.706960013	13.35727149	1.74E-09	3.62E-08
A_24_P256863	THRAP3	-1.706577957	10.54471222	8.43E-07	7.14E-06
A_23_P50241	CLPTM1	1.70620166	9.017929514	1.29E-15	3.51E-13
A_23_P212050	BCHE	-1.705697057	10.89089006	0.003963705	0.010802617
A_23_P203933	ITPR2	1.705469508	9.406243752	2.32E-06	1.71E-05
A_23_P164210	TBC1D3F	-1.705108696	13.60807925	5.48E-08	6.78E-07
A_24_P925635	Septin 13	-1.705101507	10.3716594	7.61E-06	4.77E-05
A_24_P71181	GPBP1L1	1.704931082	10.46633775	4.17E-13	3.86E-11
A_24_P922261	SRGAP1	-1.704615517	11.18719168	6.54E-06	4.19E-05
A_23_P154282	CHCHD5	1.703873679	11.30064142	1.21E-15	3.39E-13
A_23_P151544	DDX24	1.703757999	9.854624614	1.75E-10	5.19E-09
A_24_P52111	RAB24	-1.703636861	13.92623507	5.42E-07	4.87E-06
A_24_P194661	FLJ38379	-1.703336065	8.637355354	0.001129492	0.00367028
A_23_P60591	DNAJC7	1.702804587	12.91434709	1.47E-12	1.05E-10
A_23_P65068	EID3	-1.702531824	9.329176008	3.39E-09	6.35E-08
A_24_P393461	C1orf43	1.701763898	12.09565331	7.98E-11	2.74E-09
A_23_P34733	FH	1.701285976	12.65721299	2.81E-11	1.16E-09
A_23_P354297	CHTF18	-1.701162266	12.84501415	1.24E-06	9.97E-06
A_32_P83203	PARD3B	-1.700990317	11.1130692	1.04E-09	2.34E-08
A_24_P864142	LOC100272228	-1.70054244	10.3996904	6.23E-06	4.02E-05

A_24_P201021	PSCA	1.700292174	8.516404466	0.001949202	0.005876461
A_32_P123743	KCNQ1OT1	-1.699247223	13.8388517	0.000181364	0.00075129
A_24_P919920	ARV1	-1.69907412	9.13862975	7.93E-08	9.33E-07
A_24_P245838	MGAT3	-1.698564032	9.518376095	0.002462128	0.007191457
A_24_P388622	STX18	1.69791104	9.535241566	1.47E-10	4.48E-09
A_24_P114604	TBL1XR1	-1.697860659	13.94721548	3.70E-08	4.82E-07
A_24_P32672	MRPL11	1.697418619	11.36970648	4.14E-11	1.59E-09
A_23_P323252	TNKS	-1.697037504	9.879517977	3.94E-09	7.23E-08
A_24_P350200	LOC100288418	-1.696770896	16.18144937	1.24E-10	3.93E-09
A_23_P119627	NDUFA13	1.696732377	15.01912649	9.96E-11	3.27E-09
A_23_P34741	ZNF593	1.69668809	12.88648477	8.97E-10	2.07E-08
A_24_P384779	PRKAG2	-1.696283105	10.65838875	0.000810567	0.002765132
A_23_P204579	TDG	-1.695675484	15.59707251	1.00E-11	5.07E-10
A_23_P47077	BAG3	1.695432304	10.3186173	2.54E-10	7.13E-09
A_32_P223189	SUMO1P3	1.695367627	10.39318628	1.64E-10	4.90E-09
A_23_P84821	MRPL1	1.695344269	9.809508021	1.21E-10	3.84E-09
A_32_P55860	SKA2	1.695134735	10.17038052	8.92E-08	1.03E-06
A_24_P259922	LANCL1	1.694712776	11.82799814	4.97E-06	3.30E-05
A_23_P74145	CD48	1.694686274	8.997874283	2.06E-06	1.54E-05
A_23_P55127	C17orf48	1.694409245	10.2167096	2.05E-07	2.10E-06
A_23_P371266	DNM3	-1.694107094	9.266951224	5.96E-07	5.28E-06
A_23_P8482	Septin 7	1.69380947	12.22309739	6.64E-10	1.60E-08
A_23_P144684	ANKRD32	-1.692682062	12.27155464	1.70E-07	1.79E-06
A_32_P170406	TLN2	-1.692547281	11.0999403	3.09E-06	2.19E-05
A_24_P50937	LOC100128842	-1.691883098	9.873201366	1.65E-07	1.75E-06
A_23_P105900	BTF3L1	1.691682371	14.26554516	5.65E-08	6.96E-07
A_23_P408996	MBOAT1	-1.69127735	11.99198339	0.000117864	0.000516973
A_32_P89310	PLEKHM3	-1.690617894	12.9411647	2.38E-06	1.75E-05
A_23_P75741	UBE2L6	1.69030774	10.83153493	1.07E-07	1.21E-06
A_24_P187874	FLJ40712	-1.689988333	7.953196151	1.89E-05	0.000104371
A_24_P393353	XRN1	-1.689955768	7.788211256	4.43E-10	1.13E-08
A_23_P39647	SLC4A3	-1.689804942	10.46663483	1.60E-05	9.02E-05
A_23_P51718	PABPC4	1.689086858	9.667930558	7.14E-08	8.49E-07
A_23_P359015	PHF21A	-1.689040665	13.67592788	3.91E-12	2.35E-10
A_24_P196827	HLA-DQA1	1.688596723	10.08688879	0.003255595	0.009121372
A_24_P256380	GPR177	1.688285484	10.69447784	1.15E-06	9.32E-06
A_23_P99582	PNN	-1.687493379	14.64016682	8.01E-09	1.31E-07
A_23_P138168	CNN3	1.687106056	12.14713906	0.000203299	0.000830079
A_23_P109928	PSMD6	1.687103088	12.43929888	2.14E-10	6.14E-09
A_23_P58796	RGMB	-1.687022118	12.62662056	0.000537269	0.001932659
A_23_P41470	DDX60	1.686818872	9.637940452	2.41E-09	4.78E-08
A_23_P133474	GPX3	1.686496562	9.517637143	2.76E-08	3.76E-07
A_24_P160413	MTMR9L	-1.686403452	10.31094439	6.04E-05	0.000289409
A_24_P393838	TOMM20	1.685430769	12.52555177	2.60E-05	0.000138069

A_32_P180315	KIAA1529	-1.684663142	10.02752222	1.86E-05	0.000102893
A_24_P118489	WHAMM	-1.684088313	11.41085556	3.55E-10	9.41E-09
A_23_P147238	WSB2	1.683835408	10.55797816	1.96E-10	5.71E-09
A_32_P193770	C4orf47	-1.683760631	10.80680208	5.30E-06	3.50E-05
A_23_P152487	KARS	1.683481769	9.995819394	6.01E-11	2.15E-09
A_23_P84219	LIPH	-1.682652906	11.40031077	0.003170227	0.008913168
A_23_P117971	LOC100287593	-1.682450495	16.25305551	3.66E-09	6.80E-08
A_24_P151084	CNDP2	1.682429982	10.17778534	8.02E-10	1.88E-08
A_24_P348118	LGALS7B	-1.681684798	8.859875653	3.80E-13	3.60E-11
A_24_P16892	TAF2	-1.681392935	11.73354075	3.57E-08	4.67E-07
A_23_P215787	HBP1	1.681128156	11.48426227	5.52E-05	0.000267599
A_32_P40463	NUDT9P1	-1.68110191	8.756385927	1.16E-05	6.88E-05
A_23_P212792	CEP135	-1.681060443	12.11269977	2.84E-10	7.82E-09
A_23_P15654	TRAF4	1.680899996	11.44154836	1.19E-12	8.87E-11
A_24_P200162	HIGD1A	1.68044851	14.07299933	8.94E-11	3.02E-09
A_23_P45059	DOCK1	-1.679463863	13.00869198	1.53E-10	4.63E-09
A_32_P451874	ANKRD24	-1.678882909	8.735369315	1.30E-06	1.04E-05
A_23_P112883	NRBP2	-1.678347379	14.64665763	4.03E-06	2.76E-05
A_24_P144773	RNF145	1.677738467	8.189631083	3.62E-09	6.74E-08
A_24_P16997	MAP4K5	-1.67771764	10.68143908	2.73E-08	3.72E-07
A_23_P217804	CDC2L1	-1.677595999	14.2505009	1.41E-08	2.12E-07
A_23_P90155	PIH1D1	1.676503916	11.57796599	2.72E-11	1.13E-09
A_23_P46369	RAB13	1.676297677	12.25010652	3.89E-11	1.51E-09
A_32_P107777	MBD2	-1.675934966	10.07354064	8.26E-05	0.000379193
A_32_P194848	TAGLN2	1.675433351	8.960539915	2.40E-07	2.41E-06
A_23_P45424	ITGB1BP2	-1.675425525	9.663374859	1.11E-06	9.05E-06
A_23_P331638	KIF1B	-1.675408414	11.81812007	4.28E-08	5.47E-07
A_32_P135091	RBM41	-1.674943518	11.67035119	1.93E-06	1.45E-05
A_23_P206697	CYB5B	1.674728654	10.18321397	5.60E-09	9.66E-08
A_23_P122375	ZFAND3	1.674600941	10.99506698	2.46E-13	2.54E-11
A_23_P48278	ATF7IP	-1.674313431	8.620705539	3.21E-09	6.08E-08
A_24_P335048	C10orf118	-1.674029864	11.10015377	6.24E-08	7.57E-07
A_24_P129536	MUSK	1.673601726	8.63197453	1.52E-09	3.24E-08
A_24_P61864	CCDC47	1.673166811	11.26750204	4.85E-09	8.53E-08
A_24_P202497	TWSG1	-1.672930015	12.91077818	1.41E-06	1.11E-05
A_23_P217098	VPS13A	-1.672441763	12.8662132	1.46E-06	1.15E-05
A_32_P52206	TYW1	-1.671970163	11.38149421	1.76E-09	3.64E-08
A_23_P28980	PIGU	1.671469175	10.07207389	5.76E-12	3.18E-10
A_23_P334751	B3GALNT2	-1.671336356	10.87284151	1.55E-07	1.66E-06
A_23_P427014	CLDN8	1.67114795	10.21855371	0.006945915	0.017481118
A_24_P450596	ZNF850P	-1.669954188	9.63243318	8.61E-07	7.28E-06
A_24_P289376	C2orf68	1.669821477	8.657803257	4.48E-14	6.16E-12
A_23_P122906	AUTS2	-1.669615587	14.81226069	1.09E-05	6.54E-05
A_23_P99515	C13orf33	1.669582052	9.645111089	1.34E-06	1.07E-05

A_23_P108514	STK16	1.669182541	11.52377886	1.39E-10	4.30E-09
A_23_P11353	ATP6AP2	1.669066289	13.20970527	1.45E-09	3.11E-08
A_23_P39034	SMARCA4	-1.669003362	15.7364815	4.94E-13	4.43E-11
A_23_P15394	CD68	1.668509442	10.87880841	9.81E-07	8.14E-06
A_23_P103503	MDM4	-1.668213081	11.61695409	7.69E-05	0.000356892
A_23_P380092	ERP44	1.667736271	12.78448123	1.02E-07	1.16E-06
A_32_P65473	KIAA1244	-1.667512319	15.46102154	2.83E-07	2.77E-06
A_24_P532232	CREB5	-1.667412632	9.92070195	8.78E-05	0.000400055
A_23_P26413	RBL2	1.667323269	10.27261118	1.02E-07	1.16E-06
A_24_P94034	USP22	1.667267897	9.905925417	4.12E-11	1.59E-09
A_23_P56933	RTN4	1.666824118	12.65680747	2.39E-08	3.31E-07
A_23_P255812	TMEM66	1.666748551	11.29041287	2.94E-10	8.03E-09
A_23_P8269	TAAR8	-1.666705649	8.413218412	6.14E-16	2.03E-13
A_24_P53976	GLUL	1.666312162	12.8779796	1.75E-06	1.34E-05
A_23_P108932	RPL23AP32	-1.665802532	11.72507085	2.90E-09	5.59E-08
A_23_P356581	ROBO3	-1.665753702	10.21730284	1.47E-06	1.15E-05
A_24_P264192	SRRM1	-1.665468256	14.34975949	3.01E-08	4.05E-07
A_23_P13663	FAM60A	-1.66519369	12.76108114	1.12E-08	1.75E-07
A_32_P229299	PHF6	-1.665142881	13.77808586	4.91E-10	1.23E-08
A_32_P213637	LOC100128460	-1.664873836	12.47517653	1.24E-08	1.91E-07
A_24_P64126	CPNE3	1.664831885	12.30330224	1.27E-06	1.02E-05
A_24_P648176	LYRM5	1.664739165	9.314864855	2.07E-09	4.18E-08
A_23_P3994	C17orf61	1.664721188	14.07935347	1.57E-07	1.67E-06
A_24_P353964	HADHA	1.664328983	11.48017382	7.96E-09	1.31E-07
A_23_P141505	CLEC10A	-1.664301186	9.059244275	8.10E-10	1.89E-08
A_23_P217901	TSTD1	1.66372384	14.27005336	4.55E-09	8.10E-08
A_23_P15218	ZNF768	1.663590235	9.254175913	1.75E-11	7.96E-10
A_23_P259641	EZH2	-1.663240704	13.60021682	1.61E-05	9.11E-05
A_32_P104432	NCRNA00087	-1.662939024	11.62728253	6.61E-05	0.0003131
A_32_P107821	LOC100289169	-1.662310708	9.487975674	1.37E-08	2.08E-07
A_23_P37579	MORF4L1	1.662171945	11.57943843	2.22E-07	2.25E-06
A_23_P36266	PDHX	1.661984271	11.93573418	8.85E-10	2.04E-08
A_32_P198399	ADH5	1.661923617	10.44553672	2.06E-09	4.17E-08
A_24_P295806	CHID1	1.661845455	10.68959924	5.28E-13	4.68E-11
A_32_P155776	POTEK	1.661669808	13.35821034	1.20E-10	3.81E-09
A_23_P418785	STXBP5L	-1.661514915	8.962271956	0.002570099	0.007460054
A_32_P87809	tcag7.1141	-1.661407957	9.823668898	1.74E-07	1.82E-06
A_23_P96291	MAGEA1	-1.661190459	8.499345236	0.011282363	0.026503826
A_24_P226355	RANBP9	1.660961776	9.102480228	8.72E-14	1.08E-11
A_23_P218412	SPAG7	1.660892672	10.56359339	2.10E-12	1.40E-10
A_23_P330788	IQSEC2	-1.660887975	11.4281512	4.63E-06	3.11E-05
A_23_P46725	EPC1	1.659775878	11.3942272	1.91E-08	2.75E-07
A_24_P391811	DOCK10	-1.659273077	8.070876385	9.46E-06	5.76E-05
A_32_P542928	ZNF789	-1.658674267	10.86700331	8.01E-09	1.31E-07

A_23_P57941	RBM6	-1.65827226	13.03879548	1.78E-07	1.86E-06
A_23_P144224	SEC62	1.65817053	10.36378254	3.88E-09	7.13E-08
A_23_P88489	CATSPER2	-1.658161604	11.68681266	3.62E-09	6.74E-08
A_24_P236091	ENO2	-1.658003368	12.02441047	0.001498627	0.004687525
A_23_P214627	AIF1	1.657991105	10.74562604	1.76E-05	9.82E-05
A_23_P138308	CD58	1.657443497	11.10437705	3.00E-11	1.23E-09
A_23_P11629	TMEM61	1.657165728	11.03061449	0.00060393	0.002139829
A_32_P191503	LOC349114	-1.65700741	12.533986	8.02E-09	1.31E-07
A_23_P377839	NCRNA00161	-1.656975051	8.524190466	0.011926472	0.027757417
A_24_P299308	UNQ1887	-1.65657861	11.75719099	5.45E-08	6.74E-07
A_32_P98975	C15orf57	1.656477656	9.767098857	2.30E-11	9.92E-10
A_32_P189204	GAS2L3	-1.656007181	10.07879332	1.77E-07	1.85E-06
A_32_P99902	C15orf40	1.655832803	10.87609193	5.18E-12	2.92E-10
A_24_P347418	LRCH1	-1.654640604	13.02740791	3.84E-09	7.06E-08
A_24_P304154	AMPD3	-1.654037032	12.10499177	0.000272211	0.001070338
A_24_P165423	RBP7	1.653382092	11.40561199	4.07E-05	0.000204846
A_23_P27822	GPATCH1	1.65234081	8.868387714	7.41E-14	9.40E-12
A_23_P335463	GMCL1	1.65182821	8.674639441	8.34E-11	2.84E-09
A_23_P107933	VRK3	1.651352341	9.278223636	1.72E-12	1.20E-10
A_23_P105583	C12orf32	1.651110336	9.870363272	8.33E-11	2.84E-09
A_24_P328504	SP140	-1.650393646	9.788655814	4.94E-05	0.000243091
A_23_P33723	CD163	1.649956475	12.9327063	0.001057862	0.00347546
A_32_P169505	KIAA2022	-1.649795683	10.22234643	0.000403017	0.001507758
A_23_P162874	HSP90AA1	-1.649440989	15.69835344	1.41E-09	3.05E-08
A_23_P166135	XRN2	1.649260709	10.35624649	5.29E-11	1.94E-09
A_23_P70290	TMEM30A	1.649201797	10.64335119	5.44E-12	3.04E-10
A_23_P99386	TNFSF11	1.649057212	8.081923774	3.24E-05	0.000167375
A_23_P88767	PLA2G10	-1.648692139	8.177335872	3.80E-07	3.57E-06
A_24_P113295	AP2S1	1.648677384	13.44917782	7.77E-12	4.07E-10
A_24_P374516	TMSB4X	1.648465137	14.63968921	1.65E-12	1.16E-10
A_24_P315457	LOC729992	1.648294608	12.38253407	3.01E-09	5.77E-08
A_23_P301304	FGFR1	-1.648243129	11.97187523	2.12E-05	0.000115665
A_23_P398275	LOC388152	-1.648192541	14.76347874	1.50E-05	8.57E-05
A_23_P111571	HOXA3	1.648053592	14.01218632	3.53E-14	5.12E-12
A_23_P333640	PAPLN	-1.648026567	10.32690938	0.000185152	0.000765047
A_23_P422766	KLHDC1	-1.647869179	10.90207601	7.37E-05	0.000343753
A_23_P3169	GMPR2	1.646891106	8.887941463	2.89E-14	4.35E-12
A_23_P166459	LGALS1	1.646668729	13.65799402	7.47E-05	0.000347782
A_23_P137031	EIF2S3	1.646127203	12.99207756	3.80E-07	3.57E-06
A_24_P264031	LOC100131434	-1.645523955	9.827739788	2.47E-06	1.80E-05
A_23_P118038	NUTF2	1.64533887	12.60974602	9.08E-11	3.06E-09
A_23_P384056	CCDC14	-1.645161657	14.05510105	6.70E-07	5.84E-06
A_24_P350656	NFASC	-1.645031981	10.01128115	0.000593834	0.002109464
A_23_P436281	HIST2H4B	1.644802433	12.983611	1.09E-07	1.23E-06

A_24_P944049	CEP68	1.644287549	9.328018323	4.72E-09	8.34E-08
A_24_P20200	PLEKHB2	1.644215788	10.51757516	4.86E-08	6.11E-07
A_24_P94101	PRO2949	-1.644113003	7.266330381	1.51E-16	7.38E-14
A_23_P150789	PRSS23	1.644070788	13.35348623	3.81E-06	2.63E-05
A_24_P328524	KALRN	-1.643536128	10.14479383	8.60E-06	5.29E-05
A_23_P168556	STX1A	-1.643470472	11.64182653	2.48E-05	0.000132319
A_23_P258912	MYOM2	-1.642936364	9.912137902	0.000110054	0.000487246
A_23_P324706	FAM153B	-1.642865457	9.493815661	0.000138736	0.000595232
A_24_P248167	DCAF4	-1.642739432	9.127045219	1.16E-06	9.45E-06
A_23_P501007	EFEMP1	-1.642369412	11.37577043	0.019383227	0.042084724
A_23_P146584	C9orf30	1.642110778	10.53046853	2.34E-11	1.00E-09
A_23_P13929	NRIP2	-1.640629996	9.820088598	8.93E-11	3.02E-09
A_23_P44674	CRIP1	1.640445469	11.53896202	0.000336379	0.001286693
A_24_P299507	REST	-1.640416751	7.44306404	1.04E-07	1.18E-06
A_24_P291978	ADCK2	1.640225575	8.360713376	9.27E-13	7.38E-11
A_23_P4909	SNRNP70	1.640139989	10.63256947	2.60E-13	2.63E-11
A_24_P216964	NUCKS1	1.639097594	10.7534757	1.84E-10	5.40E-09
A_23_P9932	PDCD4	1.63894447	12.64702725	5.66E-06	3.70E-05
A_24_P220485	OLFML2A	1.638679617	11.75687459	0.001983423	0.005965687
A_23_P427703	MT1L	1.638294728	14.40504159	2.47E-05	0.000131842
A_23_P25030	HSD17B6	1.638062626	10.45485813	0.013565013	0.031001808
A_23_P257417	C2orf63	-1.63781312	10.97864918	2.86E-07	2.79E-06
A_24_P48162	MPG	1.637488136	12.04961033	2.19E-12	1.45E-10
A_23_P131299	ZFAND2B	1.637449827	9.755154448	1.01E-12	7.86E-11
A_24_P182183	SLC25A5	1.63721473	13.72760689	3.33E-06	2.34E-05
A_23_P45361	GLUD2	1.637117593	11.97680184	2.00E-07	2.06E-06
A_23_P112820	S1PR3	1.636998783	11.42359212	1.87E-07	1.95E-06
A_32_P87697	HLA-DRA	1.636333096	12.72141195	0.000304988	0.001183346
A_32_P101689	FAM3C	1.636226687	10.54279536	1.51E-09	3.22E-08
A_23_P372860	HIST1H2AC	1.635210843	9.227055166	9.05E-09	1.46E-07
A_23_P165959	IFT52	1.635208217	8.592211181	3.08E-15	7.14E-13
A_23_P147098	MTPN	-1.635050305	12.10225829	9.12E-10	2.09E-08
A_24_P942385	RICH2	-1.634524122	10.21915595	7.83E-05	0.000362433
A_23_P144165	DZIP3	-1.633960809	13.83864871	9.44E-08	1.09E-06
A_23_P217698	SHOX	1.633861392	10.44263885	1.22E-08	1.88E-07
A_24_P84880	LOC148709	1.633839997	11.23685016	7.97E-10	1.87E-08
A_24_P398810	EIF5	-1.633452858	10.6803844	9.16E-06	5.59E-05
A_24_P220454	CUX1	-1.633232292	14.79459644	1.80E-11	8.12E-10
A_23_P389118	ANO6	-1.633054015	13.34186951	1.74E-05	9.74E-05
A_24_P329777	ATAD1	1.632637557	9.535122481	8.42E-08	9.81E-07
A_24_P149390	EIF4H	1.632228994	8.912947299	2.29E-10	6.52E-09
A_23_P132910	RBM47	1.631177433	13.93755908	1.73E-06	1.33E-05
A_24_P52697	H19	-1.631067336	11.37270069	0.007907048	0.019519184
A_23_P93349	SLC44A4	1.630408325	12.91188546	0.000215152	0.000872765

A_24_P627984	EPM2AIP1	-1.63039463	10.898853	2.15E-06	1.60E-05
A_24_P110914	LOC100190939	-1.630370072	11.91257664	2.98E-08	4.01E-07
A_24_P341019	C20orf30	1.62959063	11.61714474	3.23E-08	4.29E-07
A_23_P419192	ZNF800	-1.629233133	10.17893477	2.32E-10	6.61E-09
A_23_P35820	CFL1	1.629100265	11.7243357	7.00E-11	2.46E-09
A_32_P13719	XPOT	1.629028229	9.770157231	1.98E-11	8.83E-10
A_23_P319583	RIMS3	-1.628752302	10.30333244	0.000235381	0.000943742
A_23_P137238	KDM5D	-1.628568331	11.33662606	7.53E-06	4.73E-05
A_32_P56525	FAM115A	-1.628383207	13.60601801	2.31E-07	2.33E-06
A_23_P400847	IDS	-1.628080544	12.85227301	5.68E-09	9.74E-08
A_23_P210253	DGKD	-1.627788715	15.65906539	6.69E-10	1.61E-08
A_23_P67785	SPAG16	1.627503325	11.07285792	0.00027004	0.001062991
A_23_P404595	ZNF417	-1.627416606	9.585076629	7.92E-13	6.54E-11
A_24_P153734	SEC16B	1.627412389	13.52125928	8.37E-07	7.10E-06
A_23_P342348	COX4I1	-1.627079729	11.74495077	1.52E-11	7.11E-10
A_23_P113701	PDGFA	-1.627016694	13.77237131	8.57E-06	5.28E-05
A_23_P210131	LOC400987	-1.626687374	10.65771739	9.84E-08	1.12E-06
A_23_P170058	PSMB2	1.62597948	13.37234092	2.21E-11	9.59E-10
A_23_P162211	MANSC1	1.625626373	9.902220423	1.73E-11	7.89E-10
A_24_P247931	FBXO25	1.625370037	12.10221797	0.000159721	0.000672366
A_24_P32473	ELOVL7	1.625144893	10.17618979	5.14E-09	8.93E-08
A_24_P685241	RFX3	-1.625091531	10.04496833	1.38E-05	7.97E-05
A_23_P2873	KLC1	1.62444668	11.34584119	9.72E-11	3.22E-09
A_23_P215505	RAPGEF5	1.623862828	10.73020948	0.000194598	0.000799189
A_23_P97005	JAK1	1.623444706	9.369370969	1.47E-14	2.57E-12
A_24_P929322	FLJ42393	-1.623281718	8.49842328	1.86E-05	0.000103151
A_32_P50603	C2orf70	-1.623268692	11.81870667	8.04E-11	2.76E-09
A_23_P202978	CASP1	1.623083139	9.672753886	4.88E-05	0.000240479
A_23_P170453	CST5	1.622962878	10.74291313	4.38E-09	7.85E-08
A_32_P53524	NTN1	-1.622562608	8.268349698	4.74E-06	3.17E-05
A_24_P920904	C8orf59	-1.622319233	11.53517137	8.11E-09	1.32E-07
A_24_P215475	ZNF10	-1.621899688	11.12885374	7.22E-06	4.57E-05
A_24_P58037	LOC392288	1.621592377	10.50876518	1.03E-11	5.20E-10
A_23_P42282	C4B	1.621104506	12.34123501	0.003016623	0.008545883
A_23_P60339	C9orf64	1.620340656	9.740803689	8.99E-09	1.45E-07
A_24_P401830	LOC100129196	-1.619862942	10.77212447	9.43E-06	5.74E-05
A_24_P51061	DCBLD2	-1.619480015	9.112048484	1.25E-06	1.00E-05
A_23_P420692	PPFIA4	-1.619254257	10.19797498	0.009024926	0.021891895
A_23_P87150	LPXN	-1.619094381	11.33700729	0.001228316	0.003948859
A_23_P313560	NCOA3	-1.618818903	13.83679437	2.33E-08	3.24E-07
A_23_P395555	ZNF226	-1.618473409	13.33877712	1.84E-08	2.66E-07
A_24_P338145	STOML2	1.618256234	11.4886367	7.00E-11	2.46E-09
A_24_P943193	PREPL	-1.6180728	14.35022388	3.46E-12	2.11E-10
A_23_P308097	PPP2CB	1.617986841	12.05595358	2.28E-10	6.50E-09

A_23_P217269	VSIG4	1.617410713	10.97638558	0.00077046	0.002644869
A_23_P42368	VTA1	1.6173847	10.59971841	1.61E-11	7.42E-10
A_24_P141629	FAM111A	1.616898989	10.35309948	2.50E-07	2.49E-06
A_24_P202139	METTL9	1.616883434	9.297758321	2.96E-07	2.88E-06
A_23_P14649	C15orf28	-1.616742534	16.07031505	1.58E-09	3.34E-08
A_24_P903378	DHRS4L1	1.61673582	8.863204578	1.29E-15	3.51E-13
A_24_P80532	CCNG2	1.616350676	10.30691103	8.80E-08	1.02E-06
A_23_P20443	LZTS1	-1.616040533	10.71619171	0.000959864	0.003200787
A_23_P134527	YKT6	1.615827256	9.820780028	4.26E-11	1.62E-09
A_23_P34554	CACNA1E	-1.6156161	8.49036681	5.25E-13	4.66E-11
A_24_P138556	EIF2S1	1.615427954	10.41624652	1.96E-09	3.99E-08
A_32_P224302	ZNF135	1.615109437	8.99864677	0.000127099	0.000551047
A_23_P159775	GABRE	-1.615055314	10.32912333	0.000279646	0.001094679
A_23_P115091	RAB25	1.614528561	11.95399675	5.93E-08	7.23E-07
A_23_P126752	CAPZB	1.614517512	11.70749566	3.44E-12	2.10E-10
A_24_P347198	DENR	1.61438	11.95355965	2.85E-10	7.86E-09
A_24_P134526	TSPO	1.613797584	12.68820174	2.31E-10	6.57E-09
A_24_P921477	C17orf95	1.613406179	10.79942537	5.11E-10	1.27E-08
A_23_P131653	PIGF	1.61292848	12.59700132	1.46E-09	3.15E-08
A_23_P257795	NDUFA2	1.612913664	15.27183687	7.74E-10	1.83E-08
A_23_P38085	CARHSP1	1.612700872	11.96937543	2.49E-09	4.91E-08
A_32_P28402	LRRC37B2	-1.612329642	12.95077853	3.39E-10	9.05E-09
A_23_P38618	PIGL	-1.611865384	11.21710161	0.000164179	0.000688662
A_23_P22557	MAP7D3	1.611029393	12.7118364	2.25E-07	2.28E-06
A_24_P357688	NIPBL	-1.610667478	9.426473236	1.25E-07	1.37E-06
A_23_P9823	MLXIP	-1.610262643	14.31106216	4.61E-06	3.10E-05
A_23_P206724	MT1E	-1.609968661	13.74407386	0.000339418	0.001297547
A_24_P119131	TMEM120B	-1.609683304	12.00848423	2.95E-09	5.67E-08
A_23_P113471	FAAH2	-1.609401943	13.43561808	0.000170114	0.000710249
A_24_P923142	ZC3HAV1L	-1.6092075	13.3226429	0.000387578	0.001457528
A_24_P337062	TSC22D2	1.609182242	8.193459597	7.74E-11	2.69E-09
A_32_P71113	SCAI	-1.608914243	11.69220116	9.13E-06	5.58E-05
A_23_P93823	RFC2	1.608897695	10.46518352	3.05E-09	5.82E-08
A_24_P450870	ZNF813	1.608711748	12.50505636	2.47E-08	3.41E-07
A_24_P186608	DPY19L2P2	-1.608167695	8.305065041	9.38E-06	5.71E-05
A_23_P359245	MET	-1.608105137	10.29092577	0.003975577	0.01082811
A_32_P172141	CDON	-1.607974404	10.27449128	7.64E-06	4.79E-05
A_23_P19576	SF3B5	1.607334604	14.32059724	7.50E-11	2.61E-09
A_23_P218892	EIF4G1	1.606797192	12.26581298	2.36E-12	1.54E-10
A_24_P360601	NOL8	-1.606577532	13.52339258	4.81E-09	8.47E-08
A_23_P163506	CYBA	1.606533322	11.12031202	1.27E-08	1.95E-07
A_23_P216325	ASAH1	1.606338513	11.27785627	2.45E-06	1.79E-05
A_23_P39088	PRMT1	1.606160531	12.13552063	2.24E-12	1.47E-10
A_23_P111343	BCLAF1	-1.605987638	13.50794842	2.41E-09	4.78E-08

A_24_P96961	SPSB1	-1.605937456	11.60685053	0.000359349	0.001363237
A_23_P12140	RPL5	1.605793517	13.98603041	2.35E-05	0.000125961
A_24_P127021	ST13	1.60571919	11.96927166	2.39E-08	3.31E-07
A_24_P157342	C3orf10	1.605565753	12.45307987	8.97E-11	3.03E-09
A_24_P103025	PCBP1	1.605474579	14.07263706	8.14E-13	6.69E-11
A_23_P253368	HOXA10	1.604249587	9.698892032	0.000380754	0.001434735
A_23_P420782	TULP3	1.603636179	9.194916553	6.36E-09	1.08E-07
A_24_P87490	FAM18B2	1.602674105	10.52777157	3.97E-13	3.73E-11
A_24_P269687	TOR1A	1.601733338	10.60701864	2.54E-10	7.13E-09
A_24_P214506	SEC13	1.601170992	11.2383595	1.28E-11	6.18E-10
A_23_P256223	VBP1	1.600720392	11.86182428	1.72E-09	3.59E-08
A_23_P307536	C1orf113	-1.599677366	11.35459993	0.000544908	0.001956682
A_23_P360215	POLD4	1.599382544	12.68915952	7.42E-11	2.59E-09
A_23_P74619	SELENBP1	1.599260865	9.779876105	4.80E-07	4.38E-06
A_23_P169766	HIPK2	-1.598662891	15.07216129	3.14E-07	3.03E-06
A_23_P35343	ZNF248	-1.59865363	10.73948128	4.08E-09	7.43E-08
A_23_P138782	ACP2	1.597880609	7.826009572	1.44E-16	7.15E-14
A_23_P158053	C9orf16	1.597856505	10.37186825	5.67E-10	1.39E-08
A_23_P77328	GCHFR	1.597574646	11.11552714	1.96E-07	2.02E-06
A_24_P119456	STAB1	1.59746606	9.869515782	3.83E-06	2.64E-05
A_23_P105803	FGF9	-1.596477371	9.469261957	0.013838869	0.031542382
A_23_P212002	NKTR	-1.596417532	14.85275967	7.58E-11	2.64E-09
A_23_P417942	FNBP1L	-1.596166195	14.92699751	9.44E-11	3.14E-09
A_23_P19657	LRP11	1.595920482	10.99208053	1.13E-07	1.27E-06
A_23_P43779	COPS4	1.595909093	13.09071964	2.60E-13	2.63E-11
A_24_P409330	MRPL52	1.595779541	13.18030721	1.87E-09	3.84E-08
A_24_P6949	FAM128B	1.595601636	13.41374683	2.31E-08	3.22E-07
A_32_P140139	F13A1	1.595375594	12.63065989	0.001973382	0.005939294
A_32_P197511	FLYWCH2	1.595128308	10.32167692	3.30E-13	3.19E-11
A_23_P209712	SP100	-1.595041174	11.67294119	5.93E-09	1.01E-07
A_24_P342880	ZDHHC3	1.594865446	10.13392932	1.52E-14	2.64E-12
A_23_P204702	TMBIM6	1.594745316	13.36553612	6.46E-10	1.56E-08
A_23_P23960	BLOC1S2	1.59444466	12.74524393	3.89E-09	7.14E-08
A_23_P10391	COL5A2	1.594041711	10.70260474	1.15E-05	6.84E-05
A_23_P9144	UHRF2	-1.593831834	14.01318707	2.49E-09	4.91E-08
A_24_P44916	CDC42EP5	1.5936856	10.77004648	1.49E-09	3.19E-08
A_24_P934594	AKAP8L	-1.59348492	12.99301482	8.34E-09	1.36E-07
A_24_P917123	MYLIP	-1.593034965	12.7275748	7.84E-07	6.70E-06
A_23_P318604	CYHR1	-1.592588136	12.11220387	2.29E-05	0.000123428
A_23_P389676	EXOC3	-1.591720375	11.69625765	1.17E-08	1.82E-07
A_24_P257201	MRPL30	1.591299427	11.55562992	2.18E-07	2.22E-06
A_23_P423389	CREB3	1.591129817	10.61423068	4.13E-11	1.59E-09
A_23_P65609	GTF2A1	-1.591064401	9.827641517	2.24E-08	3.15E-07
A_24_P367421	LOC100132816	-1.590554919	10.60243904	5.12E-07	4.63E-06

A_24_P115490	PTCH1	1.590296916	12.41396092	1.16E-06	9.43E-06
A_32_P514790	UNK	-1.590156159	13.15358008	3.70E-10	9.73E-09
A_23_P110473	NAIP	-1.590119394	12.72459379	9.83E-05	0.000441879
A_23_P50773	CRTC1	1.590015227	12.5586105	1.52E-08	2.26E-07
A_23_P154771	DUSP15	1.589622726	10.34134973	3.63E-08	4.74E-07
A_24_P93967	FMR1	-1.589496758	9.469572749	1.21E-10	3.85E-09
A_23_P26094	SLTM	-1.588704401	14.24853144	2.86E-13	2.82E-11
A_23_P214658	PBX2	1.588122326	12.59425118	3.97E-10	1.03E-08
A_23_P125107	HLA-B	1.588113244	12.68858597	3.36E-06	2.36E-05
A_23_P144294	PDS5A	-1.587982313	14.80720909	8.80E-13	7.08E-11
A_23_P137514	IVNS1ABP	-1.587618086	15.04848834	7.02E-08	8.37E-07
A_32_P133916	BNC2	-1.587112079	8.540244428	2.38E-05	0.00012781
A_23_P88522	NMB	1.58705135	11.50166258	9.15E-08	1.05E-06
A_32_P36143	LOC729088	-1.586556427	10.52211549	2.18E-08	3.07E-07
A_23_P96641	PRPS2	1.586543321	10.34282464	4.36E-05	0.000218136
A_24_P161403	LOC131055	1.586418507	13.50914354	3.19E-12	1.97E-10
A_24_P183094	ATP13A3	-1.586402169	15.9792196	1.82E-10	5.35E-09
A_24_P418086	RBM34	1.58603797	10.02590383	1.13E-07	1.27E-06
A_23_P52101	CYB5R1	1.585720648	12.25225169	1.27E-11	6.12E-10
A_23_P410625	ZNF367	1.585702741	10.46899517	4.76E-09	8.40E-08
A_23_P51754	RAP1A	1.585504474	11.31660505	1.18E-08	1.83E-07
A_23_P4764	INSR	1.585366383	11.31169602	5.40E-05	0.000262573
A_23_P305033	MAP3K7IP3	-1.585226583	9.495692571	0.000132961	0.000573682
A_23_P434710	PPP1CA	1.584480092	11.75188807	3.14E-09	5.97E-08
A_23_P110882	TSPYL4	-1.584395173	14.55024364	8.24E-09	1.34E-07
A_23_P250930	CRBN	1.584313809	11.15929422	3.82E-11	1.49E-09
A_23_P304386	HIGD2A	1.584145741	13.44601055	8.24E-08	9.62E-07
A_24_P288685	IL13RA1	1.583845117	9.202914926	2.74E-09	5.32E-08
A_23_P14673	IGDCC4	-1.583701706	8.997656979	0.000153817	0.000650487
A_32_P205550	RPL26L1	1.583378578	13.77648728	2.30E-08	3.21E-07
A_23_P96658	CYorf15B	-1.583196652	10.69520066	0.004122373	0.011160788
A_32_P110016	LOC727869	-1.582900859	10.83947289	0.00132034	0.004200834
A_24_P270166	OS9	1.582867005	11.07254941	5.16E-10	1.28E-08
A_23_P333605	ENPEP	1.582801888	8.123351621	2.28E-08	3.18E-07
A_24_P265856	SENP7	-1.582693861	12.81126959	4.24E-11	1.62E-09
A_23_P201587	SORT1	1.582414755	9.50607642	1.39E-06	1.10E-05
A_23_P329375	POU6F1	-1.582114688	9.520622219	5.18E-05	0.000253451
A_24_P18190	HSPA5	1.581845026	12.4213779	8.60E-07	7.28E-06
A_23_P72643	ADAM9	1.581543556	11.0576676	7.35E-07	6.33E-06
A_32_P99432	TRAPPC5	1.581167908	13.60202067	1.09E-11	5.44E-10
A_24_P398130	USP6NL	-1.581055803	12.04142932	2.34E-07	2.35E-06
A_24_P115183	CLDN4	1.580968585	12.10670268	5.02E-10	1.25E-08
A_23_P171107	CCNB3	-1.580913403	8.381840627	2.61E-07	2.59E-06
A_23_P88589	NR2F2	-1.580683825	15.46571684	3.39E-11	1.35E-09

A_24_P273561	C12orf35	-1.580649106	8.26027626	2.11E-08	3.00E-07
A_23_P136724	LOC344887	-1.580409769	8.371928597	9.15E-06	5.59E-05
A_24_P152968	AKR1C1	1.580235338	8.681968056	4.18E-08	5.35E-07
A_23_P36928	POLR1D	1.57985625	13.64400563	1.45E-10	4.46E-09
A_23_P137381	ID3	1.579836326	12.56504605	7.62E-06	4.78E-05
A_24_P325015	TCFL5	-1.579803505	12.3117513	7.21E-07	6.23E-06
A_24_P314640	MDGA1	1.57961936	12.5583129	1.39E-08	2.11E-07
A_32_P62106	RUNDC2B	-1.579541026	9.286541432	6.70E-07	5.84E-06
A_24_P272993	MEG3	-1.579439052	8.619577424	9.07E-05	0.000411816
A_23_P72737	IFITM1	1.579313942	15.02088103	0.001392771	0.004398734
A_23_P55990	NAPA	1.579172641	9.743565506	8.86E-11	3.00E-09
A_23_P133694	SLC29A1	-1.579101029	12.99042152	7.70E-08	9.09E-07
A_24_P103922	CTBP2	1.57901906	11.82471463	4.29E-07	3.97E-06
A_23_P51317	CCDC76	-1.57895768	11.61295617	2.44E-08	3.38E-07
A_23_P389588	TCF7L2	-1.578561149	9.878352594	1.80E-05	9.99E-05
A_23_P137935	MNDA	1.578364865	8.623370753	6.36E-05	0.000302712
A_24_P240732	IMPAD1	1.578267926	9.669685121	1.78E-10	5.25E-09
A_23_P347059	MOBKL1A	-1.577747382	13.87479596	7.99E-09	1.31E-07
A_23_P158596	AGTRAP	1.577634587	10.05027344	3.22E-08	4.27E-07
A_23_P301877	MED27	1.577270003	9.79169472	1.42E-08	2.14E-07
A_23_P56256	POLR2I	1.577264942	14.33325884	8.21E-10	1.91E-08
A_23_P106	USP33	1.57718986	9.728959237	4.60E-09	8.17E-08
A_23_P35650	RBM17	-1.57712529	13.38541698	3.34E-09	6.27E-08
S16884519	LOC399715	-1.576865218	8.814083555	0.000192126	0.000790295
A_23_P154507	ITGB1BP1	1.576703913	12.2432835	5.64E-09	9.69E-08
A_32_P179837	RNF151	-1.576346357	9.905570075	1.57E-09	3.32E-08
A_24_P92952	ARID1A	1.576009143	9.673314695	3.13E-11	1.27E-09
A_23_P387523	ZBTB40	-1.576006633	13.53157619	5.74E-09	9.83E-08
A_24_P289471	RNASET2	1.575804514	12.08296067	3.59E-07	3.40E-06
A_23_P108883	KCNK12	-1.575652967	8.745344244	8.69E-05	0.000396505
A_23_P411162	PER2	-1.575621012	7.924593185	3.07E-07	2.97E-06
A_23_P213893	MEGF10	1.575530663	7.795361888	3.59E-07	3.40E-06
A_23_P436259	ERN1	-1.575511567	13.00503777	1.26E-06	1.01E-05
A_23_P124024	MED10	1.575140601	12.08413878	1.59E-08	2.36E-07
A_24_P168510	C19orf50	1.574777192	9.80922657	3.50E-16	1.34E-13
A_23_P255057	SLC35A5	1.574653749	9.192868889	1.89E-11	8.46E-10
A_24_P214858	TREML2	-1.574560693	8.605585014	5.80E-12	3.20E-10
A_23_P213375	PCDHB2	-1.574386625	11.50032729	0.001450078	0.004555186
A_23_P68539	MAVS	-1.573984408	15.69602524	1.89E-06	1.43E-05
A_23_P159012	ANKRA2	-1.573943341	14.68938347	1.67E-08	2.45E-07
A_23_P384532	CCDC11	-1.573761566	9.864297204	0.000109855	0.000486534
A_32_P8551	NRN1L	-1.573725948	9.069483662	5.63E-07	5.03E-06
A_23_P36513	PRKAG1	1.572789251	10.80634461	1.05E-09	2.36E-08
A_24_P926025	LOC100190986	-1.572629025	12.10810868	5.26E-08	6.54E-07

A_23_P3592	HSF4	-1.572602631	10.79396262	1.39E-08	2.10E-07
A_24_P902052	KIAA0114	1.572450089	13.15495809	5.79E-07	5.15E-06
A_23_P377888	MTMR15	-1.572004052	14.07185011	4.95E-09	8.68E-08
A_32_P198731	NEURL1B	-1.571972674	14.73698761	0.000273076	0.001073086
A_23_P339633	CCDC75	-1.571959977	9.393940217	1.93E-06	1.46E-05
A_23_P100220	ESRP2	-1.57141534	14.12331577	8.15E-08	9.54E-07
A_23_P252362	MRPS30	1.571329555	10.6380786	6.15E-09	1.04E-07
A_24_P211151	EXOSC5	1.571065328	10.15455759	2.15E-08	3.03E-07
A_23_P131435	CD302	1.571025901	11.36107744	1.18E-07	1.31E-06
A_23_P110791	CSF1R	1.570738083	11.65145908	0.001582024	0.004913034
A_23_P32029	SLC35D2	1.570715055	9.843019692	3.61E-09	6.73E-08
A_23_P206466	CASKIN1	1.570648436	11.08611954	1.26E-09	2.77E-08
A_24_P355006	ADAM22	-1.570431461	9.952760337	0.00035566	0.001351095
A_23_P129101	HEXA	1.570011522	11.4813507	4.15E-06	2.84E-05
A_24_P6921	LOC541471	1.569859291	12.21360065	1.46E-06	1.14E-05
A_23_P136683	HLA-DQB1	1.569643298	10.20725522	5.34E-05	0.000259767
A_23_P135381	SP5	-1.569282099	12.2394087	0.018323461	0.040144171
A_23_P204689	CLEC2D	-1.569019733	10.93976575	5.49E-08	6.78E-07
A_23_P9926	TSPAN10	1.569011001	13.19439997	1.15E-08	1.79E-07
A_23_P302568	SLC30A3	-1.56843096	10.03588381	1.42E-05	8.16E-05
A_23_P319153	NADSYN1	-1.568237498	10.88401544	5.97E-09	1.02E-07
A_24_P570049	PPARA	1.568029854	10.38648689	4.31E-09	7.74E-08
A_32_P89755	SSR1	1.56783637	12.92722291	3.52E-07	3.35E-06
A_24_P913146	HOPX	1.567499167	9.56632748	5.36E-08	6.65E-07
A_23_P502641	SEC22C	1.567497104	11.32587977	5.29E-09	9.17E-08
A_23_P25994	LGMN	1.567466443	11.05718034	6.97E-10	1.67E-08
A_24_P74088	C18orf55	-1.567370734	9.020030773	6.37E-10	1.55E-08
A_23_P52697	CD248	1.566406243	9.750191668	9.18E-06	5.60E-05
A_24_P139773	PPP1R8	1.566360718	9.764278737	6.92E-12	3.71E-10
A_23_P208866	GMFG	1.56621924	12.11107964	3.45E-05	0.000177113
A_23_P396842	C6orf89	1.565942666	11.12908915	1.68E-12	1.17E-10
A_24_P405430	TIA1	-1.565724998	12.09283645	9.76E-08	1.12E-06
A_24_P193498	TM2D3	1.565514782	10.71499939	8.44E-09	1.37E-07
A_23_P213661	HISPPD1	-1.565306452	12.84843342	6.10E-09	1.04E-07
A_23_P208870	AKT2	1.565193626	10.79968727	2.34E-09	4.65E-08
A_32_P468341	C1orf127	1.5648585	9.598669325	5.04E-05	0.000247116
A_32_P132748	LOC100288671	-1.564726971	10.14115742	0.000699998	0.002430283
A_24_P157388	CDV3	1.564104526	11.44397174	4.75E-09	8.40E-08
A_24_P235012	MTCH1	1.563846247	11.21210265	4.40E-10	1.12E-08
A_23_P315964	UMODL1	-1.563722563	7.728959127	1.35E-06	1.07E-05
A_24_P165595	ARL5A	1.563543625	10.47352131	8.36E-09	1.36E-07
A_32_P37974	LRRC58	1.563495708	11.70149625	2.47E-14	3.85E-12
A_24_P246943	PPIA	1.563231348	14.07689218	4.12E-13	3.83E-11
A_32_P22078	RPS9	1.562937495	14.87906816	4.24E-09	7.64E-08

A_23_P94689	URM1	1.562778112	8.841725339	1.78E-16	8.21E-14
A_23_P29499	CTNNB1	1.562756274	12.11136391	8.02E-08	9.41E-07
A_23_P212914	RUFY3	-1.562573097	10.39684597	6.64E-08	7.98E-07
A_23_P216522	TMEM8B	-1.562561669	12.59252113	6.51E-07	5.69E-06
A_23_P206441	FANCA	-1.562417551	10.04823756	0.001230794	0.003954377
A_23_P12601	DHTKD1	1.562241687	8.487966853	3.37E-12	2.07E-10
A_23_P9591	LMAN2	1.56202913	12.89654409	3.46E-11	1.37E-09
A_23_P105794	EPSTI1	1.561948096	8.268567877	3.24E-05	0.000167257
A_23_P66787	ACLY	-1.561804567	12.89966164	4.40E-06	2.98E-05
A_23_P68401	CTNNBL1	1.561632286	9.915223749	1.41E-12	1.02E-10
A_24_P19662	LRRC47	1.561593468	9.090615532	1.31E-13	1.50E-11
A_23_P374351	KCTD20	1.561572929	10.29678569	9.81E-15	1.88E-12
A_24_P232856	RPL9	1.561522803	14.77326975	2.21E-07	2.24E-06
A_23_P9779	IRX5	-1.561335008	13.09434135	0.000478436	0.0017478
A_23_P59410	KIF25	-1.56101328	9.657549505	0.001662029	0.005125703
A_32_P88958	INADL	-1.560621637	10.56141142	3.81E-07	3.58E-06
A_23_P350187	TET2	-1.560518683	12.22852558	3.25E-09	6.14E-08
A_24_P205230	RNASEK	1.560445674	14.25852045	9.41E-09	1.51E-07
A_23_P37623	GOLGA8A	-1.560413995	10.44647928	0.00063868	0.002244456
A_23_P389426	MGC26597	-1.560371678	10.08187209	5.09E-07	4.60E-06
A_32_P95082	CNTLN	-1.560303232	10.72101708	5.89E-06	3.83E-05
S24302515	MSH5	-1.560036115	12.67147328	6.03E-07	5.34E-06
A_23_P154675	SNRPB	1.559927866	12.11731498	4.57E-11	1.72E-09
A_23_P26759	CANT1	1.559549804	11.41796259	4.95E-08	6.21E-07
A_23_P74359	CSRP1	1.559223384	12.09842216	4.20E-07	3.89E-06
A_23_P126790	SARS	1.559024044	12.58474376	1.78E-14	3.01E-12
A_23_P200252	C1orf57	1.558800169	11.84373833	8.18E-09	1.34E-07
A_24_P636974	LOC100289383	-1.558691161	8.491226172	2.35E-08	3.26E-07
A_23_P206110	CYP1A2	-1.558544927	14.22683995	3.31E-08	4.38E-07
A_24_P22562	C17orf88	-1.55834477	10.38795383	5.35E-05	0.00026033
A_24_P130296	C17orf79	1.558251152	13.57343484	5.39E-14	7.16E-12
A_23_P155931	MEPE	1.557887776	7.50243278	6.58E-05	0.000311708
A_24_P73036	C11orf46	1.557695526	11.44007867	5.98E-10	1.46E-08
A_32_P74357	LOC100132161	1.556895681	13.85542464	2.75E-07	2.71E-06
A_23_P38468	TAX1BP3	1.556870904	9.917118478	2.65E-11	1.11E-09
A_23_P3371	RASGRF1	-1.556459258	7.002801731	3.66E-17	2.62E-14
A_23_P148194	ADI1	1.556239023	14.91864338	1.41E-08	2.13E-07
A_24_P103886	IDI1	1.556223075	12.56143221	4.75E-05	0.000234616
A_23_P348992	MYSM1	-1.556146254	10.55920628	0.00017822	0.000739346
A_23_P155288	GFM1	1.55612237	11.6839699	2.28E-08	3.19E-07
A_24_P121271	CPSF6	1.555707303	12.06812173	3.43E-10	9.13E-09
A_23_P163622	AKTIP	1.555589892	9.209479483	1.20E-12	9.00E-11
A_23_P163235	CKMT1A	1.555579232	9.936602815	9.65E-05	0.000434786
A_24_P315564	LOC100132261	-1.555495942	9.369381459	2.12E-07	2.16E-06

A_23_P257503	HERC1	-1.555231663	13.34296345	1.30E-15	3.51E-13
A_24_P42803	WDR26	1.554852878	8.986209817	1.18E-08	1.84E-07
A_24_P298846	C6orf120	1.554541894	8.806928157	2.05E-14	3.38E-12
A_32_P70220	CHMP7	1.55438429	9.299596483	1.88E-11	8.43E-10
A_23_P259362	NPCDR1	-1.554311064	8.948598806	2.77E-08	3.76E-07
A_23_P405110	CDC14A	-1.554288893	9.539379663	0.000146211	0.000622883
A_23_P118552	HEXIM1	-1.553782777	15.21233446	3.54E-07	3.37E-06
A_32_P11230	LOC399744	-1.553213067	14.10795208	1.30E-07	1.42E-06
A_24_P216253	DLGAP4	1.553033829	13.38890982	2.88E-08	3.89E-07
A_32_P163469	NFE2L1	-1.552775354	15.94907004	4.02E-10	1.04E-08
A_24_P889103	SUZ12P	-1.552769967	10.89085135	3.35E-07	3.21E-06
A_23_P113283	ZMAT3	1.552535072	8.362085278	1.51E-10	4.60E-09
A_23_P73297	MAGI1	-1.552332703	11.71374134	3.95E-08	5.10E-07
A_23_P103511	C1orf226	-1.552208967	12.86374322	1.60E-07	1.69E-06
A_23_P28652	C2orf28	1.551780393	13.62226874	3.58E-10	9.46E-09
A_23_P7301	WHSC1	-1.551681932	10.19862271	4.87E-06	3.24E-05
A_23_P15090	DNAJA2	1.551648977	11.82904947	8.51E-08	9.89E-07
A_23_P81507	FAT2	-1.551430745	9.399402566	0.000124343	0.000540551
A_32_P18250	LOC388965	1.551359186	11.7510661	6.87E-08	8.21E-07
A_23_P62276	NKAP	1.551309809	10.87408534	5.04E-09	8.78E-08
A_23_P22224	EIF4EBP1	1.550853638	12.75240387	0.000236459	0.000947181
A_23_P48936	SMAD3	-1.550561164	12.7578539	8.44E-08	9.83E-07
A_23_P326142	C7orf54	-1.550002101	13.20176331	9.58E-09	1.53E-07
A_24_P941167	APOL6	-1.549889144	10.69694937	4.03E-05	0.000203195
A_23_P70867	SBDS	1.549769891	11.8372921	4.05E-11	1.56E-09
A_24_P148811	RUVBL1	1.549726518	9.680992582	2.35E-09	4.67E-08
A_24_P194017	NOSIP	1.549671761	10.10132805	8.38E-16	2.57E-13
A_23_P218369	CCL14	1.549621374	9.696185526	0.01142016	0.026790125
A_24_P221634	NSF	1.549553205	8.098369385	3.05E-09	5.83E-08
A_23_P95823	NSMCE1	1.549506748	13.04834067	5.01E-07	4.54E-06
A_32_P68408	CSNK1A1P	-1.548967793	11.2610832	1.31E-07	1.43E-06
A_23_P133712	CYP39A1	1.548549244	9.438276669	0.000124315	0.000540492
A_23_P85716	FCGR2A	1.548171096	10.03759621	1.46E-06	1.15E-05
A_24_P174461	EAF1	1.547939515	7.976826805	4.61E-09	8.18E-08
A_24_P942017	hCG_2039148	-1.547141884	9.567744088	4.31E-07	3.98E-06
A_23_P33072	MAF1	1.547085387	10.04513309	2.72E-11	1.13E-09
A_32_P47265	SAMD12	-1.546778298	8.295844555	2.15E-05	0.000117205
A_24_P160001	FKBP1A	1.546667317	11.22031393	3.59E-08	4.69E-07
A_32_P117723	NUDT4	1.546528509	10.99746508	5.74E-06	3.74E-05
A_23_P18213	DNAH1	-1.546394003	8.401086805	4.43E-10	1.13E-08
A_23_P46141	CTSS	1.546166085	11.34754394	9.78E-07	8.12E-06
A_24_P382001	POLR3K	1.546009974	12.19055181	1.68E-10	5.00E-09
A_24_P481824	DPY19L4	-1.545708316	11.57171632	3.46E-07	3.30E-06
A_24_P128068	UBE2O	-1.545602164	10.15769728	4.50E-11	1.70E-09

A_23_P304395	OSTCL	1.545537873	12.76273209	4.14E-06	2.83E-05
A_32_P134698	LOC440917	-1.545319665	12.24723966	2.82E-07	2.76E-06
A_24_P351304	IMMT	1.545004331	9.824923708	2.18E-11	9.52E-10
A_23_P154199	PPP1R7	1.544853674	12.78713372	3.31E-08	4.38E-07
A_32_P117145	LRDD	-1.544488965	11.41238136	3.61E-06	2.51E-05
A_23_P70307	SMOC2	1.543954717	14.04598344	0.00270698	0.007798867
A_23_P119040	GREB1L	-1.543912461	8.666110288	2.14E-05	0.000116499
A_23_P129103	LOC80154	-1.54356995	15.21539487	9.37E-06	5.71E-05
A_32_P223859	SMPDL3A	1.543469863	8.808199186	3.41E-08	4.49E-07
A_23_P315241	ACTN4	1.543458732	9.798138491	2.50E-13	2.57E-11
S4187576.2	TIAM1	-1.543274133	10.95030352	0.001021974	0.003370031
A_23_P417753	OPHN1	-1.543190173	10.71463242	0.000906374	0.003046045
A_32_P126311	SLC38A9	-1.542519541	12.80751322	5.90E-06	3.84E-05
A_24_P145316	DTNBP1	-1.542396933	11.03518667	1.48E-07	1.58E-06
A_23_P54996	TEX14	-1.542269722	8.05646759	1.26E-07	1.39E-06
A_24_P379750	MXD1	-1.542248064	13.02232719	1.43E-09	3.08E-08
A_24_P94916	LST1	1.541808752	9.562271726	1.65E-07	1.75E-06
A_23_P253571	PHF5A	1.541585738	10.20868251	1.21E-11	5.91E-10
A_23_P345212	BOD1P	1.541575037	10.06072401	5.96E-11	2.14E-09
A_24_P12932	MRPS16	1.541547328	13.73907963	3.34E-13	3.21E-11
A_23_P37503	MYO1E	1.541376942	8.199276281	4.22E-12	2.49E-10
A_23_P156355	TMEM161B	-1.541124473	12.18835225	4.23E-09	7.64E-08
A_23_P82296	GNB2	1.540817306	11.74189006	1.47E-11	6.91E-10
A_24_P263543	LOC119358	1.540406898	15.29404144	7.58E-10	1.80E-08
A_24_P932308	COQ9	-1.539922735	10.09301454	9.92E-08	1.13E-06
A_23_P355447	ZDHHC22	-1.539629776	10.6662962	0.001519982	0.00474703
A_23_P93677	C7orf42	1.539251193	11.82123734	2.75E-08	3.75E-07
A_24_P390928	TRAPPC6A	1.539096989	11.42878202	1.38E-07	1.50E-06
A_23_P37983	MT1B	1.538901277	14.16913038	7.51E-05	0.00034951
A_24_P359145	SMYD4	-1.538818412	11.75199197	2.46E-07	2.46E-06
A_23_P47527	C11orf10	1.538730913	15.38551172	2.37E-09	4.69E-08
A_23_P72961	PRPS1	1.538668904	10.95567978	1.41E-07	1.52E-06
A_23_P14184	THSD1	-1.538647628	13.02215725	1.44E-05	8.24E-05
A_23_P7957	GNMT	1.538434696	10.92569905	0.004606934	0.012289074
A_24_P300952	APLP2	1.538248988	11.26313674	5.78E-07	5.15E-06
A_23_P51019	SCN2A	-1.538205527	8.320180713	0.001455607	0.004568844
A_23_P210164	HOXD8	1.537841629	9.121613445	1.16E-05	6.89E-05
A_32_P482979	RBM12B	-1.537646071	13.59655743	3.26E-10	8.76E-09
A_23_P74001	S100A12	1.537634384	9.244255358	0.001470819	0.004610092
A_24_P384990	LOC642852	-1.537041958	13.60557087	1.03E-06	8.48E-06
A_23_P253345	C8orf4	-1.536613959	12.09460268	0.016431562	0.036549741
A_32_P154473	KIF5C	-1.536512721	15.6949014	8.17E-08	9.56E-07
A_24_P94351	C1orf56	1.536472517	10.82643168	2.93E-06	2.09E-05
A_23_P105862	FRY	-1.535837262	12.08737246	8.77E-05	0.000399712

A_23_P86230	MYCBP	1.535827065	10.23610872	2.67E-08	3.65E-07
A_24_P187954	ARMC8	-1.535643795	13.42766888	3.33E-07	3.19E-06
A_24_P170660	SPEN	1.535616466	10.82398185	8.38E-07	7.11E-06
A_23_P31372	MDH2	1.534896938	14.09841152	2.67E-11	1.12E-09
A_24_P240137	GOLGB1	1.534658765	12.95956546	1.49E-09	3.19E-08
A_23_P49924	NT5C3L	1.534647644	9.9501837	1.83E-10	5.38E-09
A_23_P52556	CTSD	1.534306211	10.06589989	1.50E-13	1.69E-11
A_23_P33433	MAZ	1.534251652	9.276482682	1.38E-09	3.00E-08
A_23_P45180	GYPA	1.53373738	7.205574636	4.53E-05	0.000224854
A_23_P216225	EGR3	-1.53305705	8.98011761	0.002456556	0.007179488
A_32_P136295	GNG7	-1.532647764	11.73375272	1.23E-05	7.22E-05
A_23_P145089	HSP90AB1	1.532480505	13.90506007	9.66E-10	2.20E-08
A_23_P59426	PAXIP1	-1.532370749	13.44843702	6.96E-09	1.16E-07
A_23_P141688	RAB31	1.532090001	12.33799466	1.18E-05	6.94E-05
gi|61966856|ref|NM_001013701.1|	LOC440157	-1.53208452	12.45749398	0.000397405	0.001488528
A_24_P381136	PACSIN3	1.531699116	8.754225711	3.30E-09	6.23E-08
A_24_P107277	DRAP1	1.531484628	11.46981581	2.37E-11	1.01E-09
A_23_P314526	FLOT1	1.531392208	10.03694819	9.38E-11	3.12E-09
A_24_P252043	PTP4A1	1.531337741	9.631565483	3.55E-07	3.37E-06
A_23_P108054	TMEM86B	-1.531127849	10.25991164	2.05E-08	2.92E-07
A_23_P203351	MS4A7	1.531031045	9.476106571	1.76E-06	1.34E-05
A_32_P56392	RBMX	1.530932478	8.77870173	4.08E-12	2.43E-10
A_24_P376309	PPP1R9B	-1.53056561	12.41465828	1.30E-05	7.57E-05
A_23_P406448	RAB9B	-1.530281908	8.781925532	0.000114106	0.00050317
A_23_P111583	CD36	1.530087796	10.28939135	0.00094646	0.003161639
A_24_P345498	XRCC5	1.530078996	12.62075484	2.02E-13	2.14E-11
A_23_P167856	TMEM63B	1.530062992	9.780589738	9.89E-12	5.04E-10
A_24_P8098	NSD1	-1.529990531	10.20986911	4.72E-08	5.96E-07
A_23_P168974	SDCBP	-1.529853827	11.17672928	6.47E-06	4.15E-05
A_24_P219769	ATP5SL	1.529710103	8.98912338	2.11E-10	6.08E-09
A_24_P131066	CD101	1.529654179	9.820879684	5.34E-11	1.96E-09
A_32_P790284	KATNAL2	-1.529272436	10.29899228	1.15E-06	9.34E-06
A_23_P77630	MAP1LC3B	1.528939211	10.54037964	2.28E-11	9.85E-10
A_23_P129925	SLFN11	-1.528677175	11.06583067	0.000270703	0.001065167
A_24_P234214	HNRPLL	1.527371993	10.82290478	4.25E-08	5.44E-07
A_24_P652510	FAM59A	-1.527368219	7.691477226	4.23E-07	3.92E-06
A_23_P40782	COMMD2	1.526985169	9.480523904	7.27E-10	1.74E-08
A_32_P11477	FAU	1.526783095	14.72561954	1.01E-12	7.88E-11
A_24_P244800	NDRG2	1.526711271	7.83419962	6.78E-13	5.73E-11
A_24_P387158	THOC4	1.526663427	10.82336022	4.73E-12	2.71E-10
A_23_P167818	FAM184A	-1.526661126	10.33571808	2.29E-05	0.000123324
A_23_P92441	MAD2L1	1.526617828	9.985039622	8.20E-06	5.09E-05
A_23_P111745	ZMIZ2	-1.526133546	13.79679525	3.61E-07	3.41E-06

A_23_P416774	CLIC5	-1.525841705	8.07262482	5.65E-06	3.69E-05
A_24_P109511	CHCHD2	1.525808082	14.59653948	5.51E-12	3.07E-10
A_24_P933801	SGK269	-1.525547599	9.394747177	5.22E-05	0.000254827
A_23_P135315	ZBTB43	-1.525238151	13.15140523	6.62E-11	2.34E-09
A_23_P31006	HLA-DRB5	1.52480636	12.107731	0.000244339	0.000974688
A_24_P113926	DPT	1.524683154	7.197977543	1.83E-06	1.39E-05
A_23_P26865	MYH3	-1.524674874	9.834267756	0.000182155	0.000754092
A_23_P78771	NUCB1	1.524597169	10.74025471	4.10E-11	1.58E-09
A_23_P9603	PRKDC	1.524586548	11.83942617	2.14E-06	1.60E-05
A_23_P257609	RPL29	1.524109037	15.15132772	6.80E-09	1.14E-07
A_23_P166023	PFDN4	1.52392693	13.05972316	1.64E-09	3.45E-08
A_24_P70993	CD99	1.523717742	12.30324985	2.32E-07	2.34E-06
S18601758	ZNF275	-1.523682372	14.25090909	4.16E-10	1.07E-08
A_23_P86818	PEX16	1.523236254	10.29324137	7.64E-06	4.79E-05
A_23_P146855	MPPED1	-1.523214724	8.81854582	0.00116875	0.003784185
A_23_P6413	SELM	1.522767655	12.8891936	0.000440148	0.001626648
A_23_P137403	LRRC41	1.522624269	9.543643304	2.72E-11	1.13E-09
A_32_P158302	LOC401397	1.522444659	11.66992191	1.45E-09	3.12E-08
A_24_P89457	CDKN1A	1.522351409	8.450278785	1.56E-10	4.72E-09
A_23_P122254	NUP153	-1.522327161	13.56323591	6.46E-09	1.09E-07
A_23_P29046	CBR1	1.522055172	13.93267608	6.97E-08	8.32E-07
A_24_P238744	P704P	1.522004816	13.85585803	2.03E-07	2.08E-06
A_23_P71319	FDFT1	1.521893745	13.78905051	5.20E-06	3.43E-05
A_24_P318457	E2F4	-1.52154549	12.89427964	7.12E-11	2.49E-09
A_23_P149562	ARHGAP29	1.521458875	9.886132944	1.52E-10	4.60E-09
A_23_P355525	HECTD2	-1.52105589	10.49933971	3.67E-05	0.000187221
A_23_P38795	FPR1	1.520035563	9.345359082	1.74E-05	9.72E-05
A_23_P353726	C10orf46	1.519623273	9.518075676	2.13E-07	2.17E-06
A_24_P240065	VPS24	1.51943446	12.89797127	1.87E-11	8.42E-10
A_32_P152237	LOC151878	-1.51902808	13.08342298	5.59E-07	5.00E-06
A_24_P350160	RSRC2	-1.518763806	11.90788121	5.94E-08	7.25E-07
A_23_P17430	RBM38	1.518725497	9.191127357	1.55E-08	2.30E-07
A_23_P250629	PSMB8	1.518471851	10.2388298	2.97E-09	5.70E-08
A_24_P67375	TPMT	1.518418819	9.140832975	1.87E-08	2.70E-07
A_24_P315873	SDHALP1	1.518373528	11.01391138	5.49E-11	1.99E-09
A_23_P97141	RGS1	-1.518357404	13.08878484	0.002896643	0.00825312
A_23_P210869	COX4I2	1.518088854	11.56743625	2.78E-08	3.77E-07
A_23_P405175	CNST	-1.518059259	13.40743674	6.83E-08	8.17E-07
A_23_P89871	ZNF415	-1.518045369	11.44683209	7.47E-06	4.70E-05
A_32_P204205	SIX4	-1.518031166	12.1358865	5.20E-06	3.44E-05
A_24_P690924	SEC23B	1.517754857	12.25247094	9.59E-08	1.10E-06
A_24_P148796	MST1	-1.517450065	13.08141472	1.38E-05	7.98E-05
A_24_P373297	ZDHHC7	-1.51742207	14.83285733	1.01E-09	2.29E-08
A_23_P35309	TAF5L	1.5168779	10.10194433	1.46E-08	2.19E-07

A_24_P923934	RAPGEF6	-1.516547245	10.65936792	2.53E-09	4.97E-08
A_23_P211285	NDUFV3	1.515647596	14.51749873	9.98E-09	1.58E-07
A_23_P41009	TEX264	1.51563763	10.42559075	1.31E-10	4.11E-09
A_23_P148602	INE1	-1.515028966	16.2307983	2.62E-10	7.31E-09
A_23_P358555	LSMD1	1.514588023	15.02577101	3.43E-08	4.52E-07
A_23_P39110	RUVBL2	1.514585169	11.20301766	1.23E-12	9.16E-11
A_23_P207520	COL1A1	1.514140786	13.87031437	0.004996461	0.013179145
A_23_P59153	LSM2	1.514019572	12.30861112	7.73E-08	9.11E-07
A_24_P170544	RYR1	-1.51371509	7.822573908	0.000157548	0.000663725
A_23_P54373	RAB27A	1.513695692	10.02436191	4.26E-05	0.000213797
A_23_P119583	PDE4C	-1.513610643	15.8932069	1.29E-09	2.82E-08
A_32_P336445	HINT1	1.513513546	15.17873526	6.99E-09	1.17E-07
A_24_P74828	MT1JP	1.513449633	9.510326962	2.50E-05	0.000133036
A_23_P115955	MRPL21	1.5133863	14.23231655	1.72E-10	5.11E-09
A_23_P19936	KDELR2	1.513185898	13.40198049	7.85E-07	6.72E-06
A_24_P148503	FZD5	1.512825837	9.587977945	0.000125459	0.00054479
A_24_P710730	LOC100170939	-1.51280235	14.83758744	0.00011643	0.000511845
A_24_P278156	TMEM38B	1.512390437	10.15562632	4.33E-05	0.00021663
A_24_P159948	ARFRP1	1.512032473	9.474561628	9.35E-12	4.81E-10
A_23_P92093	CELSR3	-1.511893617	11.41414217	0.004052561	0.011003709
A_24_P289383	CHD7	-1.511757249	12.55992096	5.20E-05	0.000254126
A_32_P167493	SAP18	1.511632632	14.60325326	3.27E-09	6.17E-08
A_23_P17773	C22orf13	1.51162221	8.914436052	3.44E-08	4.52E-07
A_24_P283635	ADAMTSL1	-1.511551985	9.335120912	2.05E-07	2.10E-06
A_24_P740705	RPS19BP1	1.511080689	13.36527407	1.34E-07	1.46E-06
A_23_P161194	VIM	1.51104575	13.19171169	7.64E-06	4.79E-05
A_32_P108722	LOC100288196	-1.510527215	12.85642983	3.40E-06	2.38E-05
A_24_P51118	MTAP	-1.51031333	11.26836122	4.22E-07	3.91E-06
A_24_P380196	CTNND2	-1.51003005	10.83063575	0.000341281	0.001303768
A_23_P47073	WDR37	-1.509843981	13.48105895	9.29E-10	2.13E-08
A_24_P927222	RBM27	-1.509713238	12.55300503	2.41E-07	2.41E-06
A_23_P107051	TCAP	-1.509647042	10.53570007	8.00E-08	9.39E-07
A_23_P31323	ACTB	1.509058916	13.5506489	1.33E-10	4.16E-09
A_24_P74160	SNRPD2	1.508763945	15.62497006	6.09E-11	2.17E-09
A_23_P103885	AURKAIP1	1.508529007	14.30161377	1.05E-11	5.29E-10
A_23_P117727	MTFMT	1.508400612	10.51492482	1.38E-07	1.49E-06
A_24_P941773	METTL7A	-1.508049404	11.28669506	2.83E-06	2.03E-05
A_23_P211864	RPP14	1.508047189	9.696484125	4.49E-09	8.02E-08
A_32_P134846	C9orf123	1.507941694	14.3070656	9.26E-07	7.74E-06
A_24_P84419	VAV2	-1.507701575	13.60738649	1.61E-09	3.38E-08
A_23_P257993	DNASE1L3	1.507359297	9.206134622	0.000797164	0.002725174
A_23_P120103	KCNS3	-1.507215472	13.7227528	0.006001707	0.015437122
A_32_P109242	CSRNP3	-1.506992815	9.27283504	6.45E-05	0.000306219
A_23_P1523	RHOD	1.506679269	9.942188068	3.67E-05	0.000187173

A_24_P286054	ZFYVE16	-1.506538569	12.9267352	5.78E-08	7.10E-07
A_23_P366328	VPS37A	1.50641719	11.601615	4.42E-10	1.13E-08
A_23_P354734	PCDHGA8	-1.506039621	13.27405575	1.38E-05	7.95E-05
A_23_P138819	MRPL49	1.505780416	9.334745127	1.27E-12	9.33E-11
A_23_P206484	GFER	1.505677429	12.03134907	4.84E-08	6.09E-07
A_24_P404540	ESF1	-1.505657611	9.140854127	2.44E-07	2.43E-06
A_24_P272594	MAPKBP1	-1.505296274	12.1568542	1.48E-07	1.59E-06
A_23_P117662	HDC	-1.50500973	8.560643501	0.000198296	0.000812195
A_23_P309865	ZNF449	-1.504653917	10.96667022	2.02E-06	1.51E-05
A_32_P34552	POLB	1.504188751	9.777497731	3.91E-07	3.66E-06
A_24_P151834	ANKFY1	-1.503497633	12.28442109	4.10E-09	7.45E-08
A_32_P27271	E2F6	1.503444945	14.38147941	1.15E-06	9.35E-06
A_23_P390677	MECOM	-1.503335878	8.952727908	0.000263581	0.001040741
A_24_P248345	OLFML2B	1.503280723	12.01313032	0.000325418	0.001251073
A_23_P321959	SFT2D1	1.502833354	12.14340417	4.50E-06	3.04E-05
A_23_P133656	LAMA4	1.502819581	12.83997472	0.000145029	0.000618733
A_32_P130788	SAMD13	-1.502370324	11.27845494	0.002500833	0.007291269
A_23_P170352	MRPL12	1.502323489	13.24948381	1.44E-07	1.55E-06
A_24_P105771	ACTR3	1.501987114	11.57306574	2.15E-08	3.03E-07
A_23_P78543	AP1M2	1.501922977	11.16895591	2.18E-09	4.38E-08
A_32_P205637	PARD6B	-1.501847135	11.61923398	9.40E-07	7.84E-06
A_24_P58529	TUBA1C	1.501757018	12.72669682	8.68E-09	1.41E-07
A_24_P371425	ATRIP	1.501081643	8.911543773	1.83E-10	5.38E-09
A_23_P208090	MBD1	-1.501066235	12.48193863	9.88E-10	2.24E-08
A_23_P312358	BEND7	-1.500483103	9.531326142	0.000328344	0.001259944
A_32_P75299	TOMM5	1.499623804	14.56982345	3.40E-08	4.49E-07
A_24_P919330	FTH1	1.499573505	14.26008331	7.54E-09	1.24E-07
A_32_P62863	SCHIP1	-1.499278076	11.2810529	6.04E-05	0.000289296
A_24_P349560	EIF4E	1.498412218	9.911923634	5.04E-09	8.78E-08
A_23_P145153	PDCD2	1.498041573	10.72725197	3.50E-09	6.55E-08
A_23_P346311	BAX	1.497969246	10.09576626	6.92E-16	2.23E-13
A_23_P324813	BCL6B	-1.497702105	10.55462244	0.003011958	0.008535168
A_23_P424582	EGFL8	-1.497362114	12.23906487	8.02E-05	0.000369847
A_23_P403335	EXPH5	-1.497340764	11.88584102	0.000163529	0.000686309
A_23_P423695	MXD4	-1.497185018	13.05686994	1.60E-06	1.24E-05
A_24_P743802	ZNF618	-1.496480739	13.33814439	6.72E-06	4.29E-05
A_23_P153286	ZNF234	-1.496458003	13.95897155	1.23E-08	1.90E-07
A_23_P89380	SLC4A1	1.496323239	7.981760263	0.000207638	0.000845922
A_23_P88347	FERMT2	1.496017702	10.25399621	3.02E-08	4.06E-07
A_23_P320371	LPIN1	-1.495729405	11.16965525	0.006059956	0.015568317
A_32_P1445	PTPN2	1.495546534	10.23214767	1.20E-10	3.81E-09
A_23_P76901	PLEKHG3	-1.495326102	13.38911124	5.40E-06	3.55E-05
A_24_P529644	H6PD	-1.495236075	10.1251983	2.38E-05	0.000127862
A_24_P392774	HSD17B12	1.495145271	8.987981564	5.39E-06	3.55E-05

A_24_P304723	PPIB	1.494714906	14.72950815	1.70E-09	3.55E-08
A_24_P115774	BIRC2	1.494432044	11.0206167	6.89E-08	8.23E-07
A_24_P59632	LOC728613	-1.494336891	11.30267012	4.62E-05	0.000228555
A_23_P213000	WDR1	1.494026014	11.68603046	9.99E-13	7.81E-11
A_24_P296772	PPP1R14A	1.493854392	11.0076874	0.006476454	0.016463435
A_23_P335428	HIBADH	1.493573926	11.90889592	1.82E-08	2.64E-07
A_24_P396980	PFN2	1.492991251	8.251145479	2.01E-08	2.87E-07
A_24_P348006	RASD1	1.492635757	8.331196005	2.56E-08	3.52E-07
A_23_P417383	ASPRV1	-1.492566481	10.42126897	1.45E-06	1.14E-05
A_23_P71316	RBPMS	1.492417571	9.585983799	4.20E-09	7.60E-08
A_24_P6083	CHCHD10	1.492365836	13.37874624	2.06E-05	0.000112748
A_24_P126425	FAM188B	1.491379992	8.549549249	6.68E-05	0.00031597
A_24_P378506	UBE2Z	-1.491262309	13.15540736	3.22E-10	8.68E-09
A_23_P87879	CD69	-1.49114548	8.033387922	0.002073246	0.006195879
A_24_P397515	HMGN4	1.490790825	10.92734247	5.29E-11	1.94E-09
A_23_P92710	RHOBTB3	1.490033733	9.739400492	2.25E-06	1.67E-05
A_24_P269624	CDC37L1	-1.48896125	10.71904053	0.000152427	0.000645666
A_23_P16806	hCG_1993592	-1.488596626	11.48152707	5.73E-08	7.05E-07
A_23_P319640	hCG_1811732	-1.488457452	10.39708319	1.37E-09	2.98E-08
A_24_P551028	SPOPL	-1.488332461	12.88143227	1.33E-05	7.72E-05
A_24_P327815	STIP1	1.487923177	9.673600342	4.44E-12	2.58E-10
A_23_P369994	DCLK1	-1.487816532	11.2907891	0.010817534	0.02554525
A_23_P254978	TATDN1	1.487271489	12.05109135	1.46E-05	8.35E-05
A_23_P56759	KRCC1	1.487120415	12.36215631	1.83E-06	1.39E-05
A_24_P104091	ORAOV1	-1.487102952	12.32324185	1.61E-09	3.39E-08
A_24_P222176	LOC283701	-1.48617824	10.16338657	1.09E-05	6.54E-05
A_23_P7882	SLC22A23	-1.485819466	14.60039059	3.39E-06	2.38E-05
A_24_P367864	USP36	-1.485586962	8.527159335	2.62E-07	2.60E-06
A_23_P27367	KDSR	1.485321025	11.17173543	2.68E-10	7.45E-09
A_23_P326474	L3MBTL4	-1.48518081	7.86807972	8.32E-06	5.15E-05
A_32_P11723	EIF2S2	1.484652986	14.39882339	1.16E-17	1.14E-14
A_24_P49533	ZYG11B	1.483897109	11.80767259	6.60E-11	2.34E-09
A_23_P5301	TFCP2L1	-1.483867101	10.27805554	0.004712089	0.012525541
A_23_P400794	FBXO45	1.48376765	9.159225202	2.39E-11	1.02E-09
A_23_P140978	ZNF263	1.48371128	8.896228824	7.23E-12	3.83E-10
A_23_P10127	SFRP1	-1.483287321	11.21106674	0.012076395	0.028054037
A_32_P5673	FABP3	-1.483030505	9.256084532	2.17E-06	1.61E-05
A_23_P502731	PRRX1	1.48302704	8.745273626	0.010007431	0.023905027
A_23_P17880	DNAL4	1.482865723	8.725949655	1.97E-15	4.79E-13
A_23_P57304	TMEM50B	1.482534217	11.24745514	6.47E-09	1.09E-07
gi|54606887|ref|NM_015206.1|	KIAA1024	-1.482293809	9.695731117	0.002333693	0.006867612
A_23_P86470	CH25H	-1.482264747	9.768095548	0.003686343	0.010148919
A_23_P108303	NDUFA7	1.482252259	15.38601582	5.39E-09	9.33E-08
A_24_P418878	ZNF174	-1.481845429	8.827338802	4.66E-06	3.13E-05

A_24_P282377	DKFZp667E0512	-1.481375481	9.003701318	0.000103685	0.000462544
A_23_P75769	MS4A4A	1.481289839	11.73906662	0.002495758	0.007280861
A_24_P269853	ACTR1A	1.481210111	9.222570418	2.07E-10	5.97E-09
A_32_P524014	UTRN	-1.480824662	16.08637885	5.12E-08	6.39E-07
A_23_P157784	HINT2	1.480651706	12.34899444	1.08E-08	1.69E-07
A_23_P161074	TOR1AIP1	1.480573741	9.606466345	7.78E-11	2.69E-09
A_32_P12183	tcag7.907	-1.480331526	14.48781828	1.05E-07	1.20E-06
A_23_P95330	ZDHHC9	1.480287781	9.918730166	1.76E-09	3.64E-08
A_23_P387471	MICB	-1.479317652	10.57785227	2.70E-08	3.69E-07
A_23_P64828	OAS1	1.478869044	9.13558153	0.000150498	0.000638612
A_23_P127926	WEE1	1.47870682	10.91019758	1.20E-05	7.09E-05
A_23_P428219	EZH1	-1.478427216	12.05953325	9.27E-11	3.10E-09
A_23_P407115	PIP4K2B	1.478335619	8.663980833	1.83E-15	4.48E-13
A_23_P305507	TOP1	1.478058581	12.00407547	4.29E-08	5.47E-07
A_23_P125078	SLC26A11	-1.477895625	14.34525103	6.67E-13	5.65E-11
A_24_P365327	LSM14A	-1.477885393	16.006865	1.36E-12	9.90E-11
A_23_P149153	PDE4DIP	1.477598571	8.511062229	2.63E-06	1.90E-05
A_24_P416728	AES	1.477434942	11.09768343	8.19E-08	9.58E-07
A_23_P250951	SLC26A2	1.477269271	11.45046357	0.001173846	0.003797507
A_24_P342127	KLHDC10	-1.477215852	10.96516888	1.69E-08	2.48E-07
A_24_P247536	FAM133B	-1.477121742	12.36717642	2.02E-06	1.52E-05
A_32_P127350	LOC645323	-1.476992556	7.625457709	3.01E-05	0.000156923
A_23_P53856	N4BP2L2	-1.476752194	15.23461912	3.73E-10	9.81E-09
A_24_P46171	PIGX	1.476045324	11.43460095	6.70E-08	8.04E-07
A_23_P164797	ZNF580	-1.475864927	12.13003763	4.54E-06	3.06E-05
A_23_P112478	NUDT2	1.475474947	11.33679877	2.47E-08	3.41E-07
A_23_P388681	ELAVL1	1.475099272	10.36216753	1.15E-07	1.28E-06
A_23_P107257	SPOP	1.474834406	12.23142874	5.55E-14	7.33E-12
A_24_P174316	KIAA1310	-1.474807196	12.60030328	1.17E-10	3.75E-09
A_23_P132248	ZMAT5	1.47465934	9.770023287	1.85E-11	8.32E-10
A_24_P51786	CCDC50	1.474525553	12.79155357	2.60E-06	1.89E-05
A_23_P22614	Septin 6	-1.4744848	11.26625305	0.000516794	0.001868563
A_24_P3415	PPP2R2C	1.474442487	11.32544438	0.003928768	0.010724017
A_24_P163537	TMED4	1.474358926	13.32677677	9.35E-09	1.50E-07
A_24_P68079	TRANK1	-1.474254659	11.56940001	4.22E-05	0.000211817
A_23_P120947	XRCC6	1.473924373	12.10519014	1.01E-12	7.86E-11
A_32_P43465	ZNF182	-1.47389229	12.163556	6.28E-09	1.06E-07
A_23_P42020	BRD7P3	1.473511124	9.447480403	2.03E-09	4.13E-08
A_23_P12062	TRIT1	-1.473334084	13.66178839	1.37E-08	2.07E-07
A_24_P15754	TOMM40	1.472395827	8.95124992	4.14E-12	2.45E-10
A_24_P411186	BCL11A	-1.47237003	8.749828698	0.007786018	0.019265888
A_23_P339818	ARRDC4	-1.471902654	13.63900468	8.72E-05	0.000397893
A_23_P350704	PRKAB2	-1.471885417	10.24396106	2.12E-08	3.00E-07
A_23_P4919	KDELR1	1.471875926	12.4657203	1.79E-13	1.94E-11

A_32_P39394	GLIS3	-1.471831665	9.638122835	0.001149086	0.003727066
A_32_P8361	KIAA0892	-1.471765914	13.75901698	4.07E-09	7.42E-08
A_23_P119448	SAPS1	-1.471227321	11.27054016	0.002090671	0.006241688
A_23_P9614	NDUFA4L2	1.471122066	8.971429505	1.05E-05	6.31E-05
A_23_P145054	FAM162B	-1.471104367	9.080860534	0.000936907	0.003134769
A_24_P16214	LOC100127980	-1.470995535	13.2605454	3.94E-06	2.71E-05
A_23_P32054	CAMSAP1	-1.470837254	9.301021699	4.12E-07	3.83E-06
A_24_P911571	GNB5	-1.470811722	8.693086015	5.35E-07	4.81E-06
A_24_P365365	TCF3	-1.47026408	14.48521517	1.91E-07	1.98E-06
A_23_P125748	ZMAT1	-1.470040832	11.43867953	0.000340077	0.001299682
A_23_P316472	DNHD1	-1.469959342	9.609262644	7.65E-06	4.79E-05
A_24_P366535	LOC100128760	1.469895719	11.63339065	2.42E-12	1.57E-10
A_24_P271830	FLJ34690	-1.46968521	8.783010512	8.52E-05	0.000389896
A_24_P254551	ARHGEF9	1.469495071	9.32645762	1.14E-05	6.80E-05
A_23_P405129	LTBP2	1.469005951	8.424493306	2.04E-07	2.09E-06
A_23_P138665	GLUD1	1.468688755	14.24465618	1.28E-05	7.48E-05
A_24_P411561	HAVCR2	1.468259066	7.798785509	1.12E-08	1.76E-07
A_32_P62769	LOC100288933	-1.468148465	11.61119604	1.23E-07	1.36E-06
A_32_P34116	NUS1	1.467944145	10.81976842	6.66E-09	1.12E-07
A_23_P371885	ARL17P1	-1.467934328	11.48157112	1.35E-06	1.07E-05
A_23_P36166	PIK3C2A	1.467274655	8.911405432	9.53E-12	4.89E-10
A_24_P230130	LOC401859	1.46721508	14.33200777	6.90E-11	2.43E-09
A_23_P117238	CARS2	1.467211705	9.450849301	2.66E-09	5.18E-08
A_24_P151622	IMMP2L	1.467054977	10.71473397	3.58E-07	3.40E-06
A_23_P120572	RIN2	1.467050075	8.696013352	6.71E-10	1.61E-08
A_24_P234572	RAB7A	1.466948483	10.42184394	1.33E-15	3.56E-13
A_23_P337424	SERBP1	1.466780385	10.40339263	2.43E-07	2.43E-06
A_32_P215318	ACACA	1.466762495	9.032179132	3.03E-06	2.15E-05
A_23_P45409	UPF3B	-1.466543938	12.0901	2.01E-07	2.07E-06
A_23_P58953	NQO2	1.466365575	10.67830003	2.34E-10	6.65E-09
A_32_P106117	GGA2	-1.46611649	13.16967018	5.99E-13	5.16E-11
A_23_P308032	NUP98	-1.465941764	12.84057519	4.42E-08	5.61E-07
A_23_P95470	CD151	1.465858174	11.18263011	1.27E-10	4.01E-09
A_23_P93258	HIST1H3B	1.465728992	10.70057383	1.53E-05	8.67E-05
A_23_P109934	HEMK1	-1.465627247	11.42661252	0.000124046	0.000539685
A_23_P4353	WSB1	-1.465194707	15.40736018	2.34E-07	2.35E-06
A_23_P327643	SMC4	-1.464868399	8.238248878	6.03E-05	0.000288806
A_23_P106362	AQP9	1.46485649	8.540568413	0.000409329	0.001528563
A_24_P12626	CAV1	1.464820636	10.49199581	8.09E-07	6.90E-06
A_24_P130865	PHF8	-1.464681067	10.06376907	1.41E-06	1.11E-05
A_23_P111054	HIST1H2BB	1.464573128	12.88056073	3.22E-06	2.28E-05
A_23_P342668	SFRS17A	-1.464413246	16.25054244	3.49E-09	6.53E-08
A_24_P414556	TTC33	-1.464139123	11.4551569	5.07E-12	2.87E-10
A_32_P524614	C17orf74	-1.464048501	10.1663344	3.58E-10	9.46E-09

A_24_P912095	RBBP4	1.464007337	8.791812058	9.15E-09	1.47E-07
A_23_P62588	CALML6	-1.463567018	8.316653888	1.00E-06	8.32E-06
A_24_P371194	MRPL53	1.463538668	13.66627418	1.34E-11	6.39E-10
A_24_P764598	PTP4A2	1.463375923	9.923507824	1.40E-11	6.63E-10
A_23_P100011	AP3S2	1.463112392	8.692393651	4.88E-12	2.78E-10
A_24_P928068	TAF15	-1.462861481	8.859279773	1.61E-05	9.09E-05
A_23_P165402	SF3B14	1.462795798	14.66354983	1.38E-10	4.29E-09
A_23_P88134	CINP	1.462651855	9.801401363	3.73E-11	1.46E-09
A_23_P211878	FLNB	1.462046921	13.51572622	4.46E-07	4.11E-06
A_23_P53298	BLOC1S1	1.462046772	13.15832654	3.18E-09	6.03E-08
A_23_P1102	ACTA1	-1.461917631	8.953022449	3.60E-05	0.000184023
A_24_P340066	ELF4	-1.461844541	11.4171974	0.000286054	0.001117277
A_23_P86002	TMED5	1.461697797	10.7619351	1.20E-08	1.86E-07
A_23_P209227	CACNB4	-1.461695423	8.023408519	0.000314717	0.001215974
A_23_P167030	PTH1R	1.461679822	8.49408403	5.84E-06	3.80E-05
A_23_P26173	TMED3	1.461633545	13.35008358	1.13E-08	1.76E-07
A_24_P107528	C12orf49	1.461113483	8.477300769	4.79E-09	8.45E-08
A_23_P51518	RGS5	1.460997008	11.36730456	0.001565475	0.004869066
A_23_P71570	OSR2	-1.460917224	11.33758623	0.000393549	0.001476332
A_23_P146526	SMC5	-1.460788358	11.61814288	6.69E-08	8.03E-07
A_23_P406424	RHOC	1.460745862	12.32244269	2.77E-12	1.74E-10
A_24_P35891	ZNF219	-1.460381329	11.43328862	5.28E-06	3.48E-05
A_24_P849467	ZNF585A	-1.460255942	9.528135491	1.57E-09	3.31E-08
A_23_P344578	FAM154A	-1.460142966	8.867386764	8.65E-10	2.00E-08
A_24_P370946	CYR61	1.460094765	10.54907968	1.07E-05	6.41E-05
A_32_P177040	SPDYE5	-1.460029342	9.07006577	3.62E-07	3.42E-06
A_23_P98261	SSSCA1	1.459910768	11.98324298	1.44E-10	4.43E-09
A_23_P251945	DCTN4	1.459854178	12.96344342	5.07E-07	4.59E-06
A_23_P110403	PDLIM3	-1.45954403	12.56879753	0.011138506	0.02620088
A_23_P408271	HSD17B11	1.459501322	12.37081919	0.00511584	0.013431771
A_24_P940441	HIST1H2AK	-1.459224255	9.292692484	0.000120265	0.00052548
A_23_P215963	STK3	1.459023422	9.727452581	6.01E-08	7.32E-07
A_32_P94798	ANXA2	1.458993899	13.81588714	4.97E-06	3.30E-05
A_23_P214897	AKAP12	-1.458709409	9.907185447	0.000501417	0.001821302
A_23_P3921	FLJ11710	-1.458626868	11.23805846	6.15E-07	5.44E-06
A_24_P373877	SYT5	-1.458580574	9.441446738	4.38E-08	5.57E-07
A_23_P39402	ALKBH6	1.458561159	10.07160891	5.54E-11	2.01E-09
A_23_P79978	SLC24A3	-1.458441282	9.623742301	0.009517917	0.022918221
A_23_P55998	SLC1A5	1.458438326	9.694946017	2.92E-10	7.98E-09
A_32_P180435	SPDYE1	-1.45808043	9.242139736	2.44E-06	1.78E-05
A_32_P148085	TOB1	-1.457716957	12.69796585	3.22E-06	2.28E-05
A_23_P210658	SLMO2	1.45760711	12.4793587	4.02E-07	3.74E-06
A_23_P170290	TMEM57	1.457454242	10.1973844	8.98E-09	1.45E-07
A_24_P54485	CCDC115	1.457315296	8.510827428	3.70E-13	3.52E-11

A_24_P407323	PPM1A	1.457091515	10.18219951	1.24E-08	1.91E-07
A_24_P230176	CCDC137	-1.457048619	11.74317224	7.84E-09	1.29E-07
A_24_P366315	KIF21A	-1.456954341	13.87134249	3.36E-07	3.21E-06
A_23_P111206	FKBP5	-1.456580703	14.66499918	0.00240794	0.007056591
A_23_P161041	KLHL12	1.456444168	11.11144877	1.78E-06	1.36E-05
A_24_P589028	LOC100294469	-1.456383661	7.997063126	5.94E-05	0.000285223
A_23_P121345	TBCCD1	1.455973304	9.29365366	3.30E-11	1.32E-09
A_23_P136552	PIGG	1.45554003	10.54557602	1.57E-10	4.74E-09
A_23_P91850	IL20RB	-1.455450003	10.04342082	1.99E-05	0.000109607
A_24_P29733	PFTK1	1.455425368	10.68264534	5.75E-05	0.000277557
A_24_P202948	DGCR6	1.455233582	11.3980302	1.06E-10	3.45E-09
A_24_P140171	CRTAP	1.454929157	10.4544152	1.14E-10	3.67E-09
A_23_P132718	SEMA3B	-1.454625173	11.83627766	1.53E-05	8.67E-05
A_24_P342086	WWP2	-1.453817379	11.55048842	5.48E-06	3.60E-05
A_23_P44177	C3orf38	1.453772599	9.061900722	1.64E-15	4.08E-13
A_24_P231104	LEPR	1.45376863	8.939516469	0.000306192	0.001187304
A_23_P420442	SEMA6D	-1.453465041	9.685304955	0.000675605	0.002356782
A_32_P34970	LOC100287822	1.45338084	8.983034541	4.17E-10	1.08E-08
A_24_P273063	BAALC	1.453306648	12.38834498	2.07E-08	2.95E-07
A_24_P926400	GGA1	-1.453234509	11.54506636	1.28E-06	1.03E-05
A_24_P56130	MYL6	1.453152821	15.02546371	1.77E-10	5.24E-09
A_23_P6535	KLHDC7B	-1.45305695	7.978655294	2.25E-08	3.16E-07
A_24_P328668	ARL13B	-1.453046316	12.39238612	2.73E-08	3.72E-07
A_23_P218111	SERPINA1	1.452650665	12.90634856	2.16E-06	1.61E-05
A_23_P33444	SNTB2	-1.452116608	13.33132895	1.11E-06	9.08E-06
A_32_P231617	TM4SF1	1.452077255	12.53107814	4.39E-07	4.05E-06
A_23_P85543	RNF2	1.452006696	10.33492929	1.01E-08	1.60E-07
A_24_P937546	CMTM7	-1.45170654	10.27018989	1.37E-08	2.08E-07
A_23_P144264	TOMM70A	1.45139041	10.24321635	4.46E-08	5.66E-07
A_24_P338603	SKI	1.45138324	10.58758399	1.09E-06	8.93E-06
A_24_P193295	RAB15	-1.450709131	13.14186434	3.58E-07	3.40E-06
A_23_P259270	CDKL2	-1.450531565	8.423601233	0.003905811	0.010667375
A_24_P187706	GOLGA4	-1.450529717	15.52972897	1.01E-09	2.29E-08
A_23_P132738	CRYGS	-1.450467027	9.911744738	5.07E-06	3.36E-05
A_32_P131367	RNF13	1.450367783	10.37055576	1.50E-06	1.17E-05
A_23_P159893	CHRDL1	-1.450106044	9.893751596	2.83E-06	2.03E-05
A_32_P95823	UBXN4	1.450076893	10.3051234	3.74E-06	2.59E-05
A_32_P10003	TUBA3D	1.450067128	13.45294973	2.15E-10	6.16E-09
A_23_P127394	CRY2	-1.449796863	12.99690377	6.60E-07	5.76E-06
A_24_P252310	FAM131B	-1.449623078	8.247212686	0.00111476	0.00363003
A_23_P34142	WBP5	1.44909397	11.88568091	0.001785152	0.005446812
A_23_P371076	KLF12	-1.449012362	8.458242798	2.37E-06	1.74E-05
A_23_P205389	MOAP1	1.448799825	10.4565352	2.06E-12	1.38E-10
A_24_P416055	FAM103A1	1.448681979	11.31034248	3.41E-11	1.36E-09

A_24_P398585	UNG	1.448619814	9.510069904	1.88E-08	2.71E-07
A_23_P406782	HPN	1.448262018	12.19967232	0.000349661	0.0013321
A_23_P165380	CRYGA	1.448247078	12.41204074	5.52E-08	6.81E-07
A_24_P128041	ATRX	-1.448030058	8.637555618	5.53E-06	3.63E-05
A_24_P259328	MIB2	-1.447719005	10.71680696	4.11E-08	5.28E-07
A_23_P145264	HLA-F	1.447530883	11.87981545	2.91E-07	2.84E-06
A_24_P364066	HIPK3	-1.447251092	14.89540667	7.05E-08	8.40E-07
A_32_P220519	CEP152	-1.446836682	8.896122407	0.000124822	0.00054233
A_23_P386905	UBXN6	1.446366773	10.54400092	2.28E-15	5.48E-13
A_24_P20873	HIST1H4I	1.445413953	11.95267428	2.39E-06	1.75E-05
A_24_P344416	DSC3	-1.445221302	7.489433265	0.000143222	0.000611767
A_24_P205252	FAM13C	-1.445017746	8.964324067	0.010032838	0.023956844
A_23_P99044	KRT71	-1.444978038	9.432244943	1.94E-07	2.01E-06
A_32_P27020	NBPF15	-1.444964414	16.74644032	7.56E-14	9.56E-12
A_24_P303915	SSNA1	1.444285776	11.58877103	1.03E-13	1.24E-11
A_32_P174713	SRP72	1.444106457	11.22927858	4.51E-09	8.04E-08
A_23_P256033	EEF1A2	1.444002009	11.00017564	0.000305277	0.001184111
A_23_P158024	DCTN3	1.443917941	12.95169121	1.44E-06	1.14E-05
A_24_P157926	TNFAIP3	-1.443504235	11.15265537	7.92E-06	4.94E-05
A_32_P36217	SON	1.443473903	10.24198088	5.49E-10	1.35E-08
A_24_P385376	DPH5	1.443228967	10.45089355	9.54E-10	2.18E-08
A_23_P152838	CCL5	1.443087901	9.205164922	3.86E-06	2.66E-05
A_23_P203103	ARCN1	1.442903118	8.751981525	5.48E-12	3.05E-10
A_24_P63109	PPP1R2	1.442849429	10.43081436	6.37E-09	1.08E-07
A_32_P86623	ZBED1	-1.442534023	10.800315	1.31E-08	2.01E-07
A_23_P63816	NRBF2	1.442404172	9.757017597	5.54E-11	2.01E-09
A_23_P351734	NPHP4	-1.442043401	12.12341973	1.13E-08	1.77E-07
A_24_P416301	FOXK2	1.441826787	10.09515714	3.55E-08	4.65E-07
A_24_P100130	BCL2L1	1.441738965	8.388670911	6.22E-11	2.21E-09
A_23_P122228	NDUFS6	1.441720797	14.98422655	1.76E-09	3.65E-08
A_24_P59099	LOC643668	1.441311272	9.724323438	3.67E-09	6.82E-08
A_23_P118203	ZG16B	1.441299911	14.76090604	0.001181653	0.00381893
A_24_P934487	STXBP2	-1.441110045	10.03579226	2.24E-09	4.49E-08
A_24_P252497	TRIB1	-1.440219945	16.18435898	0.000642047	0.002254857
A_32_P117338	AFG3L2	1.439913749	9.212516639	1.98E-09	4.03E-08
A_24_P184445	MMP19	-1.43988953	9.547023453	2.00E-09	4.07E-08
A_23_P85969	ZNF326	-1.439517955	13.64978491	2.94E-09	5.65E-08
A_24_P303589	CUL1	1.439448535	11.86715737	9.17E-11	3.08E-09
A_32_P45894	STAG3L1	-1.439385579	15.02128033	1.45E-07	1.56E-06
A_32_P92171	LOC388564	1.439107193	12.5048341	4.58E-08	5.80E-07
A_23_P250002	HACE1	-1.438766629	11.48761638	1.06E-06	8.74E-06
A_23_P215790	EGFR	-1.438504018	11.66636342	4.90E-05	0.000241427
A_24_P943095	CYTSB	-1.438363664	10.04557513	3.64E-05	0.000185942
S2654756	ZNF37B	-1.438282348	8.745974203	3.20E-06	2.26E-05

gi|51972191|ref|NM_001004305.1|	LOC284757	-1.438162371	10.45027765	0.014066016	0.031997913
A_23_P44105	LAT	-1.437754546	11.51138552	2.86E-07	2.79E-06
A_24_P73389	STK24	1.437462158	8.871880903	2.08E-11	9.19E-10
A_24_P157370	IL17RB	-1.437430618	10.50002181	0.000815566	0.002780959
A_23_P161727	HSPB2	1.437150212	9.765050795	0.000365522	0.001384023
A_23_P301340	C17orf56	-1.43713174	13.96359911	3.03E-08	4.06E-07
A_24_P302998	ATP5I	1.437010454	15.23024527	3.79E-11	1.48E-09
A_23_P351232	ZNF775	-1.436973867	11.25923679	2.45E-07	2.44E-06
A_23_P134755	TRPS1	-1.436435322	12.31999423	0.000173443	0.000721808
A_24_P291278	LAX1	-1.436407369	11.1614437	3.04E-07	2.95E-06
A_23_P251377	C3orf37	1.436391761	11.20482063	1.30E-08	1.99E-07
A_24_P274814	TBXAS1	1.435337293	9.504952701	1.49E-05	8.52E-05
A_24_P39724	CCDC43	1.435313575	9.822839438	3.46E-10	9.20E-09
A_23_P53152	C11orf17	1.435084756	11.37741581	5.64E-11	2.03E-09
A_23_P143068	IQCA1	-1.43492623	9.0930334	0.001048243	0.003448251
A_23_P74581	SNHG12	-1.43483561	14.65443715	1.47E-05	8.39E-05
A_23_P390116	SPATA13	-1.43478656	11.24972754	6.05E-06	3.92E-05
A_24_P80633	CTNNA1	1.434501893	11.70309302	6.03E-11	2.15E-09
A_23_P375566	STXBP4	-1.434373254	10.03368538	2.26E-05	0.00012191
A_24_P78540	NR2C1	-1.43427034	12.10095435	9.08E-09	1.46E-07
A_23_P148546	ARD1A	1.434093493	12.04557531	4.99E-08	6.25E-07
A_23_P89163	EXOC7	-1.434057708	13.70145286	8.15E-11	2.79E-09
A_23_P161022	SNAPIN	1.433979364	12.41408557	5.20E-12	2.93E-10
A_24_P115199	FOXK1	1.433398707	10.63504534	6.04E-08	7.35E-07
A_24_P159181	PDCD7	-1.433368585	15.44255252	4.24E-07	3.93E-06
A_32_P65061	ERCC4	-1.433363162	10.49954889	2.60E-06	1.88E-05
A_32_P68586	ARL1	1.433339332	12.63503516	1.73E-06	1.33E-05
A_23_P340728	PSEN1	1.432071197	10.08238878	2.58E-10	7.22E-09
A_23_P200493	LBR	-1.432015937	15.79874895	1.66E-09	3.48E-08
A_23_P251232	TTTY14	-1.431851833	12.82397082	8.36E-07	7.10E-06
A_24_P731648	SLC16A6	-1.431780559	11.22967742	4.23E-06	2.88E-05
A_24_P940599	SYNCRIP	1.43158474	10.30904559	2.45E-09	4.84E-08
A_23_P501634	BTN2A1	-1.431199511	11.36650602	1.51E-07	1.62E-06
A_23_P429959	PRX	-1.430962594	10.87081266	4.45E-07	4.11E-06
A_24_P255338	SEC24C	1.430828132	10.00067398	5.68E-10	1.40E-08
A_23_P203505	TRIM5	1.430739395	9.344523192	1.11E-06	9.05E-06
A_23_P103968	AKR7A3	1.43067813	11.1315915	4.53E-12	2.62E-10
A_23_P103905	UFC1	1.430493954	13.58952646	1.74E-07	1.82E-06
A_32_P222450	TMEM158	1.430449567	12.04640663	2.55E-06	1.85E-05
A_32_P328023	WDR33	-1.429604758	8.947709903	0.000987652	0.003277351
A_23_P29141	SNAP29	1.429573639	10.39614959	1.14E-10	3.66E-09
A_24_P16631	LOC100133039	-1.429492543	11.27100909	1.04E-08	1.63E-07
A_23_P27392	NCRNA00085	1.429434624	10.28177138	1.20E-05	7.09E-05

A_24_P161725	CXADRP3	1.429262424	10.04102315	2.86E-06	2.05E-05
A_24_P944588	ZNF682	-1.428860549	11.09711825	0.000227973	0.000917847
A_32_P223173	LOC392335	-1.428770582	10.45095981	3.87E-07	3.62E-06
A_24_P237486	MECP2	-1.428717261	11.73664504	4.29E-08	5.47E-07
A_23_P139509	ERGIC2	1.428452195	12.68618857	3.95E-10	1.03E-08
A_24_P170454	USP6	-1.42834157	10.8598356	1.97E-06	1.48E-05
A_23_P340188	FAM122C	-1.428339063	9.071390149	7.57E-05	0.000351743
A_23_P411814	OSTC	1.42787142	13.88947875	1.03E-05	6.18E-05
A_23_P303671	ECM2	1.427791285	9.061179413	2.31E-05	0.00012431
A_23_P49810	HOXB7	1.427648814	9.369440818	4.26E-06	2.90E-05
A_24_P37319	ACSL3	1.427641588	13.31787021	0.006991381	0.017576111
A_32_P35232	KIAA1958	-1.427396346	11.35797448	3.92E-05	0.000198196
A_23_P31584	RABL5	1.427272835	13.64954601	5.08E-08	6.35E-07
A_23_P215525	OSBPL3	-1.427100507	10.7419905	2.56E-05	0.000135946
A_32_P82895	C2CD4C	-1.426869033	10.21207284	0.002111176	0.006294649
A_24_P882871	FLJ21408	-1.426594184	8.045145809	3.96E-08	5.11E-07
A_23_P150162	DRD4	1.42658922	13.7035249	1.00E-07	1.14E-06
A_24_P418816	GPX7	1.42650996	8.697119409	0.000132355	0.000571338
A_23_P87432	RHPN1	-1.426453611	12.88414335	0.001044368	0.003436964
A_23_P258221	ABCC5	-1.426411748	15.49585714	9.11E-06	5.57E-05
A_23_P52176	ZNF124	-1.426278809	11.35325396	2.67E-05	0.000141526
S3603036	KIAA1843	-1.426254539	8.4936674	0.004697111	0.012493449
A_23_P153889	PGLS	1.425849385	13.3426169	1.50E-09	3.20E-08
A_24_P181510	ZNF646	-1.425724658	10.17778127	6.02E-10	1.47E-08
A_24_P317762	LY6E	1.425639692	9.98450023	9.92E-06	6.00E-05
A_23_P138352	WNT2B	-1.425518044	8.917074146	0.000986228	0.003273186
A_24_P803801	GTF2I	1.425505401	11.14387254	4.60E-09	8.17E-08
A_23_P69497	CLEC3B	1.42542528	12.0656152	0.000899093	0.003024202
A_23_P131240	UBXN2A	1.425296387	9.025033257	8.39E-12	4.36E-10
A_24_P303199	ARL15	1.425060879	8.419059937	7.23E-07	6.24E-06
A_24_P121631	ZNF764	-1.42496375	11.99625474	5.88E-08	7.20E-07
A_24_P32520	RNF214	-1.424599859	10.87557307	1.25E-07	1.38E-06
A_23_P819	ISG15	1.424541468	14.18184074	0.001688214	0.005196107
A_23_P17633	IFNAR1	1.424442171	11.58854119	2.44E-10	6.90E-09
A_24_P16592	ADCK4	-1.424021282	8.416279974	2.34E-07	2.35E-06
A_23_P148879	ATP1A2	-1.423848291	8.727002214	0.00046483	0.00170491
A_32_P97169	GPC6	-1.423351333	10.43208985	0.003988001	0.010855847
A_32_P14187	TFAP2A	1.42314539	8.635888927	2.24E-05	0.00012122
A_23_P34983	JTB	1.422942674	15.0884379	4.01E-10	1.04E-08
A_24_P940517	PCYOX1	1.422598487	11.12954667	3.92E-07	3.66E-06
A_24_P363477	FTSJ2	1.422428175	9.673331884	1.35E-12	9.79E-11
A_23_P358995	IBTK	-1.422164365	14.20907091	7.21E-09	1.20E-07
A_23_P137865	MTX1	1.421929872	11.4454514	1.05E-12	8.07E-11
A_23_P30995	CYB5R4	1.42185344	9.978245682	6.02E-08	7.34E-07

A_23_P28730	ZNF512B	-1.421662832	12.23394385	6.98E-08	8.33E-07
A_23_P370514	EPB41L4B	-1.421520529	13.71730579	6.76E-05	0.000318979
A_23_P217168	CXorf36	-1.421463291	10.94751869	0.000236779	0.000948169
A_23_P46351	TDRKH	1.421407418	9.631486517	1.20E-11	5.87E-10
A_23_P158794	RSRC1	-1.421397627	11.36271739	6.59E-08	7.94E-07
A_23_P326080	DEFA4	1.421395725	7.600796115	0.001299142	0.004141906
A_23_P130900	NCLN	1.42095488	9.818727548	6.70E-09	1.12E-07
A_23_P418006	LCE2A	-1.420583202	10.26553704	2.66E-07	2.63E-06
A_23_P122464	ZNF193	-1.420444316	11.45098534	1.34E-07	1.46E-06
A_24_P295601	KIAA0652	1.42041382	10.59903762	1.58E-10	4.75E-09
A_24_P592012	ZBTB46	-1.420370525	10.4646283	4.41E-05	0.000219924
A_24_P377328	SLU7	1.420135603	11.81767304	4.66E-17	3.01E-14
A_24_P476086	KPNA5	-1.420060653	10.59155969	5.65E-06	3.69E-05
A_23_P314642	ZNF280C	-1.419710344	11.12556494	6.75E-06	4.31E-05
A_23_P125668	SLC25A43	1.419424019	11.12858778	6.77E-05	0.000319406
A_24_P160696	C3orf15	-1.419211139	9.267744195	6.61E-06	4.23E-05
A_23_P67355	PRRG2	1.418902541	8.326206692	7.13E-10	1.70E-08
A_23_P339191	CBL	-1.418836269	12.66001787	2.55E-07	2.53E-06
A_23_P342612	HCN2	1.418446638	13.56160514	6.39E-08	7.73E-07
A_32_P22263	HMGN1	1.418391722	12.5993039	1.07E-07	1.21E-06
A_24_P497437	LOC283788	-1.418363696	11.36585532	0.00257379	0.007467966
A_24_P940218	PPP2R2D	-1.418362313	11.32526123	8.31E-06	5.15E-05
A_23_P110802	CENPH	1.417936429	10.15857448	2.10E-05	0.000114931
A_23_P71014	WBSCR22	1.417849922	12.29367526	1.97E-12	1.33E-10
A_23_P22548	CHM	1.417823008	9.441347015	1.19E-10	3.81E-09
A_23_P78677	C19orf53	1.417568356	13.88610498	2.19E-09	4.39E-08
A_23_P90419	PBX4	-1.417526447	11.27660654	0.000200227	0.000819177
A_24_P279797	MRI1	-1.417404621	12.72374841	4.60E-08	5.82E-07
A_24_P943283	DENND1B	-1.417283566	10.84112804	1.25E-05	7.32E-05
A_24_P93371	COMMD4	1.417134549	11.50432819	8.48E-11	2.88E-09
A_23_P404606	C5orf41	-1.417021677	14.16032849	7.39E-07	6.35E-06
A_23_P130089	IFT20	1.416828702	12.23147669	5.54E-10	1.36E-08
A_24_P191656	ACAD9	1.416803257	10.8541084	4.48E-13	4.10E-11
A_32_P114896	PTGES3	1.416707451	10.86920058	5.52E-09	9.54E-08
A_32_P119096	MBOAT2	1.416650323	13.75860911	0.002105572	0.006280843
A_24_P24685	LOC729595	1.416480169	9.560094025	1.19E-06	9.66E-06
A_23_P140146	IFI27L2	1.4161358	12.07206209	0.000118963	0.00052096
A_23_P64879	KCNJ8	1.415674285	11.19017954	0.000699819	0.002429962
A_23_P126186	DEGS1	1.415521397	12.98299684	1.22E-05	7.17E-05
A_23_P132515	SIDT1	-1.415457753	11.31101424	0.004691381	0.012480782
A_23_P342654	PALM2-AKAP2	-1.41536645	15.65245241	2.66E-15	6.28E-13
A_23_P258018	MYL5	1.415349755	10.5845693	0.000127564	0.000552939
A_32_P217643	LOC100287039	-1.415300687	10.957722	5.57E-06	3.65E-05
A_24_P156049	SLC39A6	1.415287932	12.43910812	3.73E-06	2.59E-05

A_23_P84670	GRIN1	1.415285495	10.5274243	1.02E-05	6.14E-05
A_32_P66020	RUNDC2A	-1.414958099	9.230488227	4.74E-08	5.97E-07
A_23_P212497	ACAD11	-1.414878491	11.42053076	2.10E-06	1.57E-05
A_23_P121489	TBC1D23	-1.414377239	10.00013088	3.30E-08	4.36E-07
A_23_P115046	EIF2B3	1.414311266	10.54047196	1.44E-07	1.55E-06
A_23_P133582	ETF1	1.414146987	12.57570763	1.58E-08	2.35E-07
A_23_P117599	AHSA1	1.414123729	12.20434332	6.99E-07	6.06E-06
A_32_P37247	LOC642826	-1.414102585	12.60420274	3.21E-07	3.08E-06
A_23_P397341	PAQR4	1.41399183	8.985205072	1.85E-07	1.92E-06
A_23_P216215	MTFR1	1.413938391	9.265069857	6.56E-12	3.55E-10
A_23_P320250	MYO19	-1.413782989	14.23623522	1.25E-06	1.01E-05
A_23_P400147	CCDC61	1.4136105	13.44573363	3.12E-06	2.21E-05
A_23_P390172	RNASEL	-1.412411619	11.65718141	3.94E-06	2.71E-05
A_23_P4679	ERF	1.412314159	9.991382297	3.87E-06	2.67E-05
A_32_P223935	LOC554203	1.412191656	11.68563114	1.37E-06	1.09E-05
A_24_P216313	ERGIC3	1.412171641	11.51457635	4.11E-13	3.83E-11
A_23_P97725	STX6	1.412076249	9.578983735	3.56E-06	2.49E-05
A_24_P932203	MYST3	1.412053713	8.835158536	8.65E-08	1.00E-06
A_23_P212196	OGG1	-1.411750662	12.1131213	6.79E-08	8.13E-07
A_23_P210074	ZNF514	-1.411678999	11.67525871	3.28E-08	4.35E-07
A_24_P50245	HLA-DMA	1.41160638	11.60702657	8.04E-05	0.000370918
A_23_P163380	MTHFS	1.411480991	13.78134768	7.20E-09	1.20E-07
A_24_P181055	ST3GAL4	1.410864057	9.413334911	5.59E-10	1.38E-08
A_23_P64173	CARD16	1.41075706	9.403676791	0.000150205	0.00063772
A_23_P259692	PSAT1	-1.410566519	13.51537159	0.000524458	0.001893088
A_23_P32414	RP6-213H19.1	-1.410507689	13.39965651	3.63E-05	0.000185426
A_23_P58102	EXOSC7	1.410472638	11.60892753	2.89E-09	5.57E-08
A_23_P136012	FBXO8	1.410009379	10.55157703	1.13E-08	1.77E-07
A_23_P70161	ITGA2	-1.409970979	7.749490458	1.11E-07	1.24E-06
A_23_P100654	ZBTB4	-1.409651594	14.27179274	2.32E-09	4.63E-08
A_23_P201432	ADSS	1.409609044	11.81292483	3.55E-08	4.65E-07
A_23_P49033	SNX1	1.409576703	11.57775246	3.10E-11	1.26E-09
A_24_P29277	COL4A3BP	-1.409352341	14.06431221	1.88E-08	2.71E-07
A_23_P83175	PTPLAD2	1.409146664	7.826518254	1.22E-09	2.69E-08
A_23_P155624	AP2M1	1.408681135	11.61760681	4.34E-08	5.54E-07
A_23_P156209	ZNF622	1.408563227	11.7245884	2.22E-08	3.11E-07
A_23_P55421	CBX8	-1.408518524	8.475913311	3.19E-06	2.26E-05
A_23_P137543	ZNF362	-1.408505267	14.17861833	8.50E-07	7.20E-06
A_23_P51711	CELA2B	1.408262975	11.27274073	1.25E-08	1.93E-07
A_24_P170927	LOC644613	-1.408196989	12.10201867	0.000225863	0.000910393
A_23_P368934	FBRSL1	-1.408064674	13.16293678	2.52E-06	1.83E-05
A_24_P20806	PRR7	1.407760509	9.255970816	1.10E-07	1.24E-06
A_23_P217379	COL4A6	-1.407732921	8.021061799	3.03E-05	0.00015791
A_23_P360240	MYEOV	-1.407663574	9.121114968	1.05E-12	8.07E-11

A_24_P854199	PHB	1.407570873	12.31233289	4.55E-10	1.15E-08
A_32_P16975	ANKS3	-1.407412061	12.49057455	1.91E-06	1.44E-05
A_24_P16815	ZMYM3	-1.406998386	10.61509269	2.09E-05	0.000114138
A_23_P126825	SLC16A1	1.406381335	12.19073309	1.70E-05	9.55E-05
A_24_P319635	MCL1	1.405911786	10.7649689	2.31E-06	1.70E-05
A_23_P125451	PTPRC	1.405760703	8.341222282	2.28E-06	1.68E-05
A_23_P22214	PRKAR2A	1.405464757	8.905303111	1.67E-09	3.50E-08
A_23_P205031	COL4A2	1.405423833	14.74800228	0.001380899	0.00436801
A_23_P352358	KIAA2013	1.404680564	12.55072002	8.61E-15	1.70E-12
A_23_P6935	CD47	1.404627664	12.53638725	4.11E-05	0.000206904
A_23_P168490	HERPUD2	1.404522126	9.861946353	5.96E-15	1.24E-12
A_24_P120115	CFLAR	1.404287835	9.558250611	2.33E-11	1.00E-09
A_32_P55161	AGAP1	-1.404174341	13.73144595	2.59E-06	1.87E-05
A_24_P532330	LOC254128	-1.403699113	11.34021984	9.82E-08	1.12E-06
A_24_P230691	KIF13A	-1.403681626	10.02027298	0.000253004	0.001004306
A_24_P100277	CYB5R3	1.403398992	9.074489513	2.14E-09	4.30E-08
A_24_P100368	DYNLT3	1.403201433	10.63525268	1.36E-05	7.87E-05
A_24_P6808	SDCCAG1	-1.402773398	15.07632721	2.67E-11	1.12E-09
A_23_P266	PIN1L	1.402282086	10.13056843	5.59E-11	2.02E-09
A_23_P138655	CYP26A1	1.402279764	8.542024553	0.004002665	0.010888094
A_23_P56288	LENG8	-1.402058376	10.04443311	8.86E-13	7.12E-11
A_24_P759674	OBFC1	-1.401874794	10.94396241	2.47E-05	0.000131898
A_24_P331727	TMED7	1.401692052	11.24618453	9.79E-08	1.12E-06
A_23_P6561	FLJ10213	-1.401669527	13.46804953	1.17E-07	1.30E-06
A_23_P164415	CCDC40	-1.401666899	9.34289601	2.88E-05	0.000151324
A_24_P453855	PNPLA7	-1.401605526	10.51794248	0.001036565	0.003414187
A_24_P277955	FIS1	1.401213611	12.97859737	1.20E-09	2.66E-08
A_23_P170246	OCRL	1.400963853	8.069421971	4.02E-08	5.18E-07
A_23_P119526	AP3D1	1.400750854	10.4584284	1.11E-10	3.57E-09
A_24_P128442	TBX15	1.400602203	9.803698023	0.000101503	0.000454483
A_24_P208452	BBS5	-1.400527091	11.67190343	3.40E-07	3.25E-06
A_23_P108657	WDSUB1	1.400466881	10.44888442	5.02E-09	8.77E-08
A_23_P79231	CREB1	-1.400218166	11.90161597	1.33E-07	1.46E-06
A_32_P127052	NUDT16	1.400016793	12.53915602	2.56E-08	3.52E-07
A_32_P56001	CD93	1.399876886	10.34413725	0.006593134	0.016723784
A_24_P785293	SNHG1	-1.399686195	15.34564764	1.16E-05	6.85E-05
A_32_P466514	C14orf4	1.399643689	9.096647359	3.50E-12	2.13E-10
A_24_P337504	C5orf24	-1.399214967	13.6941002	1.15E-05	6.83E-05
A_24_P283928	OBFC2B	1.399205278	8.99019151	2.13E-10	6.14E-09
A_24_P367473	CCR3	-1.398853972	9.550776946	4.84E-09	8.52E-08
A_23_P32444	MXRA8	1.398774057	12.96048467	0.001369094	0.004335988
A_23_P314191	ZDHHC17	-1.398506916	13.0342435	1.24E-05	7.23E-05
A_23_P98763	LRTOMT	1.39841219	9.689570299	6.78E-09	1.13E-07
A_23_P46396	PTBP2	-1.397884845	13.53335069	1.29E-05	7.51E-05

A_23_P168014	HIST1H2AJ	1.39774981	14.7998971	0.000144533	0.000616752
A_24_P346604	UBE2W	1.397716585	10.44817799	1.26E-08	1.93E-07
S4614511	FLJ39632	-1.397024904	12.46365743	0.000966854	0.003220248
A_23_P155666	NAAA	1.396993535	12.47986634	8.49E-06	5.23E-05
A_32_P222961	SPIN4	-1.396906491	12.51699262	0.000150497	0.000638612
A_23_P6433	MB	1.396540198	10.05883226	8.39E-07	7.12E-06
A_24_P75072	SMUG1	1.396439227	11.41608715	4.03E-11	1.56E-09
A_23_P258582	GK5	-1.396231732	10.99778449	0.000332541	0.001273775
A_23_P144697	RAD1	1.396109816	10.88622571	4.38E-09	7.85E-08
A_24_P362931	SLC4A7	-1.396046298	12.81150502	4.20E-07	3.89E-06
A_24_P401432	LOC100128343	-1.395973265	9.593221919	1.75E-07	1.83E-06
A_23_P374689	GAD1	-1.395902009	8.711276316	0.001586156	0.004924683
A_24_P677734	LOC729082	1.395725889	10.18300244	1.22E-07	1.35E-06
A_24_P331128	GNA15	1.395572605	8.753770167	5.01E-06	3.32E-05
A_23_P216836	DNLZ	1.395546389	11.54105813	1.75E-08	2.56E-07
A_23_P155103	ADSL	1.395526157	10.60186184	1.03E-07	1.17E-06
A_24_P292710	C14orf129	1.395081045	9.251563984	5.22E-06	3.45E-05
A_24_P100382	GK	-1.39492704	7.652621287	3.27E-06	2.31E-05
A_23_P159986	MGC16121	-1.394860271	10.34160768	0.000154821	0.000654374
A_23_P208835	MAP2K2	1.394819602	11.50380169	8.71E-09	1.41E-07
A_23_P61280	COPG2	1.394655381	11.18115914	2.14E-06	1.60E-05
A_24_P921144	NUFIP2	-1.394402115	15.14073305	6.28E-09	1.06E-07
A_23_P93302	GABBR1	-1.394193771	10.79769839	1.99E-06	1.49E-05
A_24_P278552	MPZL2	1.393994603	8.902259554	0.000579193	0.002065609
A_23_P346206	RAE1	1.393952828	11.41609858	2.89E-10	7.94E-09
A_24_P372959	CCDC91	-1.393892421	12.6120778	4.94E-08	6.21E-07
A_24_P323815	MYCBP2	-1.393844299	11.5885591	1.27E-05	7.38E-05
A_24_P102327	BPA-1	-1.393654949	9.222710985	9.80E-06	5.93E-05
A_24_P406693	P4HA1	1.393423403	10.9285191	0.000153013	0.000647936
A_23_P128868	OR11H12	-1.393226206	7.487784613	3.68E-13	3.51E-11
A_23_P5481	DCAF17	-1.393076032	11.47780047	1.35E-06	1.07E-05
A_23_P130764	KCNJ14	-1.392963336	10.94702079	5.97E-05	0.00028668
A_23_P137276	AKAP13	-1.39259665	13.11707758	3.29E-07	3.15E-06
A_24_P213924	KIAA1609	-1.392420613	13.19849191	8.17E-09	1.33E-07
A_23_P37317	LOC100293413	-1.392389978	9.844671292	1.75E-06	1.34E-05
A_24_P930169	C5orf25	-1.392355063	10.0874443	1.58E-06	1.22E-05
S16883879	SHC2	-1.392172974	8.288201558	1.53E-05	8.70E-05
A_23_P163580	CENPT	-1.391961661	8.533236673	1.82E-08	2.64E-07
A_23_P123905	EXOSC3	1.391604932	12.87862153	3.69E-06	2.56E-05
A_24_P327181	WNK4	-1.391417549	10.46277458	0.008458871	0.020692629
A_24_P133085	CCDC90B	1.39125207	11.28219077	1.49E-07	1.60E-06
A_24_P363548	HIP1	1.391186834	9.401957982	4.97E-09	8.71E-08
A_24_P186986	LOC390424	1.391023327	13.44762236	1.31E-09	2.86E-08
A_23_P376014	DNAH6	-1.390771533	7.827777584	0.000436504	0.001615081

A_24_P149036	DPYSL3	-1.39076862	14.09053614	0.003275571	0.009168044
A_24_P335221	RTN3	1.390717827	10.75274581	3.21E-08	4.27E-07
A_23_P77274	UBE2Q2	1.390551915	9.955551415	3.12E-10	8.44E-09
A_23_P162037	ARNTL	-1.390412677	11.50091215	6.23E-07	5.49E-06
A_23_P86653	SRGN	1.390395562	12.25210901	6.56E-06	4.20E-05
A_24_P226962	KIAA0368	-1.390373961	10.52105987	7.37E-05	0.000343753
A_23_P201711	S100A6	1.390249087	13.00719115	0.000614109	0.002171322
A_23_P303423	SLAIN2	1.390232627	8.9295385	2.80E-06	2.01E-05
A_23_P315364	CXCL2	-1.390143131	10.05856947	0.001068997	0.003506387
A_23_P167812	RBM24	-1.390062183	8.501543071	1.16E-05	6.86E-05
A_24_P153713	MARVELD3	-1.389995072	12.38051125	0.000227438	0.000915978
A_24_P354451	ACOX3	-1.389814574	12.37806702	9.97E-06	6.02E-05
A_23_P206103	ULK3	-1.389697186	14.46679525	3.11E-06	2.21E-05
A_23_P153783	USF2	1.389666416	10.34648563	0.002587936	0.007503949
A_23_P400217	MUM1	-1.38961697	14.07891996	1.35E-06	1.07E-05
A_23_P134477	C7orf50	1.389555788	11.46469923	3.76E-12	2.27E-10
A_23_P47863	TM7SF3	1.389131488	10.68916026	1.33E-07	1.46E-06
A_23_P75470	SCYL1	1.389106916	9.455743297	7.77E-11	2.69E-09
A_23_P95553	WDR6	-1.389061651	16.11254614	6.97E-06	4.43E-05
A_23_P53162	STIM1	1.388885723	11.16485204	1.56E-10	4.72E-09
A_23_P333138	MON2	-1.388628765	10.0010764	0.000171744	0.000715972
A_23_P32233	KLF4	1.38850708	9.317099478	2.42E-09	4.79E-08
A_32_P749354	EIF1AD	-1.38847865	13.30648598	3.12E-14	4.59E-12
A_24_P391368	ATXN10	1.38809377	12.36104019	1.21E-08	1.87E-07
A_23_P423197	RXRA	-1.387883789	13.91903818	6.48E-08	7.83E-07
A_23_P94338	ENPP2	1.387597064	9.22616181	0.000630039	0.002218906
A_32_P82383	LOC100291647	-1.387134208	8.074160331	3.23E-05	0.000166842
A_24_P53051	LACTB	1.387066386	9.365247148	1.52E-09	3.24E-08
A_24_P538478	MED28	1.386986227	10.14710011	2.04E-06	1.53E-05
A_23_P213166	C4orf21	-1.386855756	11.30632091	1.11E-05	6.61E-05
A_23_P88626	ANPEP	1.386634141	8.461107249	3.23E-07	3.10E-06
A_23_P254594	GNRH1	-1.386633179	10.02854465	5.64E-08	6.96E-07
A_24_P215407	DDX6	-1.386523165	9.821124441	4.01E-08	5.17E-07
A_24_P348594	UPK3A	1.385819047	8.366466769	4.86E-05	0.000239267
A_23_P345118	PIM1	-1.385774965	14.33986687	0.000554391	0.001987046
A_24_P160440	MRPL55	1.385460984	14.11452651	2.32E-08	3.23E-07
A_23_P392501	TNRC6C	-1.38539488	14.11941504	4.47E-09	7.98E-08
A_23_P104607	PSMC3	1.385282271	11.49263504	1.61E-12	1.13E-10
A_24_P126682	SMN2	1.385183597	9.252318677	8.07E-09	1.32E-07
A_24_P414719	NFYA	-1.385021536	12.22946888	6.44E-05	0.000306009
A_24_P347378	ALOX5AP	1.384956721	9.035500634	0.000617322	0.002181288
A_23_P130107	ZZEF1	-1.384782055	10.42555312	4.97E-07	4.51E-06
A_24_P407235	CRY1	-1.384683666	11.31596908	0.000510989	0.001850857
A_24_P23258	GRAMD4	-1.384641386	13.79375759	0.007562363	0.018789369

A_23_P71644	FANCG	-1.384631135	12.97736094	1.10E-06	9.01E-06
A_23_P136964	RPGR	-1.384624591	11.97919564	8.19E-06	5.08E-05
A_23_P159671	PHKA2	-1.384510158	13.54367467	0.000178125	0.000739124
A_24_P124624	OLR1	-1.384363471	7.608109385	9.50E-05	0.000428538
A_23_P27346	SMAD4	1.384347052	10.00370541	1.07E-09	2.41E-08
A_23_P319133	DNAJC10	-1.38413374	14.65991524	2.26E-06	1.67E-05
A_23_P118158	HS3ST2	1.384074175	8.959638661	0.001022027	0.003370031
A_23_P37167	MYH6	-1.383909646	8.668147076	0.000167947	0.000702109
A_23_P217120	EHMT1	-1.383721753	14.00069842	1.36E-10	4.22E-09
A_23_P130352	KCTD1	1.383626104	9.228790576	4.69E-05	0.000231909
A_24_P937855	SIKE1	1.383560987	9.572037474	1.09E-09	2.43E-08
A_24_P282274	TCTN1	-1.383423844	10.79968604	1.22E-05	7.17E-05
A_23_P203729	RAB6A	1.38339472	8.920127067	8.80E-09	1.42E-07
A_23_P204696	CDKN1B	1.382930418	8.938944271	5.31E-08	6.60E-07
A_23_P59787	LUC7L2	-1.382809375	14.6873134	1.95E-11	8.70E-10
A_23_P158007	ZDHHC12	1.382407147	9.776608	4.36E-12	2.55E-10
A_24_P241373	MTA1	1.382306709	9.423291872	1.46E-06	1.14E-05
A_23_P138461	C10orf2	1.382098556	8.531865167	4.07E-08	5.24E-07
A_32_P32116	FAM123A	-1.382042446	8.63449321	1.07E-10	3.46E-09
A_24_P386622	ARRB1	1.38199566	8.469557156	3.87E-07	3.62E-06
A_24_P3761	ALDH5A1	1.381901229	8.29696885	3.08E-12	1.91E-10
A_23_P217428	ARHGAP6	-1.381051865	13.76411653	0.001763186	0.005394252
A_24_P231546	FAM178B	-1.380972572	10.48583619	5.33E-08	6.61E-07
A_23_P48080	RPL13P5	-1.380811447	11.18941652	1.50E-05	8.55E-05
A_23_P1029	MFAP2	1.380803992	8.571241105	1.97E-07	2.03E-06
A_23_P16562	SFRS14	-1.380718186	11.76215426	5.44E-09	9.41E-08
A_24_P166661	TMEM60	1.380148374	12.18469959	3.86E-09	7.11E-08
A_23_P420610	FCHO2	-1.380137334	8.435755344	1.40E-06	1.11E-05
A_23_P29939	SNCA	1.380107435	9.66559522	0.001323138	0.004208352
A_23_P82550	STK17A	1.380047506	8.689998897	1.80E-08	2.62E-07
A_24_P13041	RTKN2	-1.379415161	9.775635002	0.000138655	0.000594962
A_24_P313597	BECN1	1.378769966	11.81536296	4.97E-08	6.24E-07
A_32_P203430	ZNF30	-1.378406675	11.96232065	0.000646159	0.002266862
A_32_P111609	TOMM7	1.378365841	16.23161007	4.99E-09	8.73E-08
A_23_P158938	AP3M1	1.378343794	10.15926501	1.34E-08	2.04E-07
A_24_P278172	ZFAND5	1.378308384	9.454947549	1.43E-08	2.15E-07
A_32_P157531	PDCL3	1.378106457	10.14971445	8.30E-09	1.35E-07
A_24_P393787	TRNAU1AP	1.378105226	9.776713421	3.41E-13	3.26E-11
A_32_P100439	C7orf41	-1.378042688	13.59506178	0.003027106	0.008569308
A_23_P40347	HM13	1.377231478	12.85624938	3.74E-09	6.91E-08
A_24_P303354	HIST1H2AG	1.377153603	15.33337477	3.71E-05	0.000188854
A_24_P8151	LOC100131170	-1.376716048	8.779920063	1.60E-05	9.06E-05
A_23_P207003	Septin 4	1.376620325	7.828782077	6.02E-11	2.15E-09
A_23_P141415	MYCBPAP	-1.376489958	9.443246714	0.010059241	0.024009521

A_23_P50674	C19orf36	1.376486625	7.733221285	2.92E-10	8.00E-09
A_32_P177097	ZNF557	-1.376421875	12.83242671	2.34E-06	1.72E-05
A_23_P37727	CX3CL1	1.37614483	11.86235779	0.008439005	0.020649254
A_23_P102988	TXNRD2	1.375861655	8.795734076	2.52E-08	3.47E-07
A_23_P6674	LXN	1.375692084	12.37499717	0.005896486	0.015205899
A_23_P118150	ARL6IP1	1.375685123	11.94561763	0.000146971	0.000625914
A_23_P105973	SERPINA11	1.375341896	7.449623123	9.65E-06	5.86E-05
A_24_P336853	PNO1	1.375145523	10.51620358	5.22E-11	1.92E-09
A_32_P144599	MACC1	-1.375050548	7.790380162	0.001117272	0.003636983
A_23_P431418	U2AF2	1.374871874	9.680981913	1.58E-10	4.74E-09
S34548103	LOC391706	1.374744249	10.48380443	1.26E-05	7.35E-05
A_23_P395438	HTRA3	1.374714489	11.44265974	0.018151763	0.039813087
A_23_P258698	MANBA	1.374325648	10.97787392	1.55E-07	1.65E-06
A_24_P398670	MRP63	1.374055954	15.40893597	6.94E-07	6.02E-06
A_23_P128663	SACS	-1.374042952	12.52535609	9.38E-05	0.000423986
A_23_P164702	MRPL4	1.373689136	11.26928203	5.45E-10	1.35E-08
A_23_P423331	NTNG2	-1.373495164	10.94400466	0.015656329	0.035067241
A_24_P100605	RALGAPA2	-1.373285438	12.34620158	0.000617272	0.002181288
A_24_P300777	ADAM8	-1.373075109	12.48625111	6.64E-05	0.000314068
A_23_P112798	CRIP2	1.372780329	10.70745404	3.79E-05	0.000192703
A_23_P170820	PUM1	-1.3727415	16.04087855	4.41E-16	1.54E-13
A_23_P205867	NR2E3	-1.372610462	9.475414218	0.002417797	0.007081725
A_23_P125001	RAC3	1.372554303	10.6352056	0.000728472	0.002516132
A_24_P206776	CRYAB	1.372146058	12.25850873	0.01975829	0.042776648
A_24_P215628	DCUN1D2	-1.372144304	11.77248241	1.78E-09	3.67E-08
A_24_P259154	MGC16703	1.372030532	8.76617763	2.55E-05	0.00013552
A_24_P82074	CPSF2	1.371946038	8.104614044	1.50E-09	3.21E-08
A_23_P16733	RALB	1.371780129	9.16080698	3.80E-08	4.93E-07
A_32_P182662	AIDA	1.371557694	10.27158084	0.000158717	0.000668361
A_32_P95914	C6orf167	-1.371334453	10.45731983	0.000117639	0.0005161
A_23_P15305	PRPSAP1	-1.371221581	13.73114952	1.74E-11	7.89E-10
A_23_P414978	NUDT14	1.371165816	9.712443996	3.05E-09	5.82E-08
A_24_P278126	NBN	1.371116348	10.10303835	8.24E-07	7.01E-06
A_24_P224469	TRPV3	1.370894045	10.26345651	1.22E-08	1.88E-07
A_23_P93027	FGF18	-1.370719711	10.2184451	0.000235707	0.000944757
A_23_P104798	IL18	1.37051641	8.617300197	1.79E-06	1.36E-05
A_24_P410952	PEA15	1.370245288	10.14224612	3.04E-12	1.89E-10
A_24_P195831	CCDC34	1.36995081	11.19719369	1.04E-06	8.58E-06
A_23_P24586	ACCS	-1.369448449	10.17218671	2.74E-05	0.000144395
A_23_P212179	HRH1	-1.36942068	9.322299632	1.85E-06	1.40E-05
A_23_P104188	ELF3	1.36901124	11.8991502	0.001631542	0.005048985
A_24_P109432	NBEAL1	-1.368769777	11.03205121	9.87E-08	1.13E-06
A_23_P56894	CYP20A1	1.368664049	10.17435957	6.59E-08	7.94E-07
A_23_P211106	SETD4	-1.368412223	11.31244041	3.26E-08	4.32E-07

A_23_P123539	PPP2R2A	1.368058079	11.99346058	3.18E-07	3.06E-06
A_23_P202565	SHOC2	1.367692728	11.73168396	1.86E-06	1.41E-05
A_23_P144877	ATOX1	1.367664607	13.64672404	5.88E-08	7.20E-07
A_23_P257198	NDUFS4	1.367643673	14.14972573	8.09E-08	9.48E-07
A_23_P167005	GPR160	1.367365089	12.6269863	0.000510812	0.001850572
A_24_P360763	CREM	1.367337338	9.48370062	8.15E-10	1.90E-08
A_23_P219045	HIST1H3D	1.367288303	11.21681666	0.000670411	0.00234024
A_32_P139505	RP1-21O18.1	-1.367037994	9.760427972	4.53E-05	0.000224841
A_23_P334538	KLK3	1.367011533	13.85569375	0.018398539	0.040279007
A_23_P111306	RBM16	1.366582289	10.13599126	2.51E-10	7.05E-09
A_24_P263036	ALDH4A1	1.366507897	11.97723333	5.31E-05	0.000258734
A_24_P86389	HIST1H2AM	1.366447847	15.62755656	9.21E-05	0.000417156
A_23_P56673	INO80B	1.366086851	8.852192072	7.14E-12	3.79E-10
A_23_P350879	PRO2852	-1.366077953	10.58683354	1.68E-06	1.29E-05
A_23_P121480	CD200	1.365732966	8.732269769	1.17E-06	9.49E-06
A_23_P35412	IFIT3	1.365655975	8.319735788	7.73E-08	9.11E-07
A_24_P212457	GON4L	-1.365494835	12.38493662	3.90E-06	2.68E-05
A_24_P133991	ANKRD12	-1.365172356	13.66716649	5.70E-06	3.72E-05
A_23_P153905	IER2	-1.364270723	15.59454798	1.34E-10	4.18E-09
A_23_P251186	PROSC	1.364083184	8.885543323	1.54E-07	1.64E-06
A_32_P226009	C5orf42	-1.362579024	9.546909928	7.26E-05	0.000339479
A_23_P163113	PRPF39	-1.36172492	13.18938883	1.94E-05	0.000106863
S16887498	LOC149448	-1.361403142	12.43918087	2.15E-08	3.03E-07
A_24_P200848	SNX13	-1.361234484	13.32149537	8.98E-06	5.50E-05
A_24_P401174	KIAA0556	-1.360980968	9.502579835	5.86E-08	7.17E-07
A_24_P25470	ZNF586	-1.360960912	9.94762164	3.31E-05	0.000170539
A_23_P325017	ZNRF2	1.360653355	11.10276009	2.47E-08	3.41E-07
A_23_P164081	MYH10	1.360451849	10.48306411	4.37E-09	7.84E-08
A_23_P315378	ATG16L1	-1.36014096	8.384481733	9.53E-07	7.94E-06
A_23_P28811	MOCS3	1.36004278	8.942176398	2.04E-13	2.15E-11
S34549607	LOC729470	1.359809026	11.60245631	7.99E-08	9.39E-07
A_23_P23616	PLEKHN1	-1.359750107	11.50966703	0.002685327	0.007747746
A_23_P1926	SDHAF2	1.359625747	12.35262781	9.93E-13	7.79E-11
A_32_P61954	UQCRFS1	1.359189818	14.3113746	4.84E-10	1.22E-08
A_32_P116840	CTAGE5	-1.358769429	12.49623679	5.42E-05	0.000263217
A_23_P7194	LGI2	-1.358423017	8.454551607	0.000306446	0.001188167
A_24_P222114	N4BP2	-1.35782093	10.89022893	1.19E-05	7.02E-05
A_24_P392109	CENPN	1.35781414	11.22100541	9.09E-06	5.56E-05
A_24_P11587	PEX13	-1.357723829	12.12364285	1.29E-08	1.97E-07
A_24_P324871	CRABP1	-1.357642424	8.006799579	2.70E-12	1.71E-10
A_24_P161973	ATP11A	-1.357580968	12.93440221	0.000767942	0.002637392
A_24_P367666	ASH1L	-1.357450958	10.40458489	7.44E-06	4.69E-05
A_23_P203751	TMEM135	1.357447653	10.27985833	7.92E-06	4.94E-05
A_23_P323488	C9orf102	-1.356984036	9.264703853	1.32E-07	1.45E-06

A_24_P478495	TMEM191B	1.356785346	10.75394404	3.43E-09	6.44E-08
A_24_P100419	EDA	-1.356690053	8.853538853	0.001277271	0.00408193
A_23_P104804	ZBTB16	-1.356508121	13.63470772	0.000969424	0.00322766
A_24_P382661	ETS2	1.356395344	9.574575878	1.03E-06	8.48E-06
A_23_P124988	KCNH4	-1.356195343	8.245216668	6.19E-13	5.30E-11
A_23_P50919	SERPINE2	1.355872676	10.27792887	8.71E-07	7.35E-06
A_24_P89038	LOC93622	-1.35581771	10.19937071	1.48E-08	2.21E-07
A_23_P45087	ZNF107	-1.355680221	12.10901201	6.46E-05	0.000306637
A_23_P58747	N4BP3	-1.35566956	9.167820984	0.000939167	0.003141092
A_24_P212860	C9orf156	1.35565553	8.663713653	2.45E-12	1.58E-10
A_23_P43425	C9orf40	-1.35559862	11.37516846	6.83E-08	8.17E-07
A_23_P256641	KCNE1L	-1.355323925	10.21484228	0.001077513	0.003529533
A_23_P200936	MTR	-1.354970815	12.43590732	5.21E-09	9.05E-08
A_24_P924389	BZW1	1.354522345	9.45626159	3.86E-06	2.66E-05
A_24_P940790	C17orf101	-1.354163488	12.15784583	1.34E-08	2.04E-07
A_23_P153867	LASS4	1.354119957	11.8020417	1.44E-05	8.24E-05
A_32_P64016	RUNDC2C	-1.354101199	8.387046127	1.06E-06	8.73E-06
A_23_P348894	SEC23A	1.35409024	9.372568062	4.69E-11	1.75E-09
A_24_P356338	GABARAPL2	1.353925036	13.26561695	4.09E-06	2.80E-05
A_23_P347048	SGPP1	1.353917251	10.09036742	2.17E-05	0.000117967
A_23_P86171	FOXD2	-1.353827481	10.44113891	0.00296257	0.008417405
A_23_P37076	BAG5	1.353827284	9.937514159	1.24E-09	2.74E-08
A_23_P155417	ABHD14B	1.353768518	11.33009083	1.32E-06	1.05E-05
A_23_P110445	APBB3	-1.353670296	11.81557572	2.42E-06	1.77E-05
A_24_P332837	MSL1	1.353600971	12.08570486	5.43E-07	4.88E-06
A_23_P202448	CXCL12	1.353209729	11.21214791	0.022307173	0.047426561
A_24_P322741	IL10RB	1.353185977	10.71285037	1.24E-12	9.20E-11
A_32_P456318	SFRS13B	-1.353060173	10.74462209	0.000270161	0.00106336
A_24_P160969	TP53I11	1.35299646	8.171696688	9.68E-10	2.20E-08
A_24_P375813	RPL7A	1.352970085	14.71041586	3.55E-07	3.37E-06
A_23_P88904	NTHL1	1.352822166	10.93907609	1.05E-06	8.61E-06
A_32_P28685	SNRPA1	1.352659681	11.96884433	2.17E-07	2.21E-06
A_23_P161488	TIAL1	1.352630952	12.07103139	3.86E-07	3.62E-06
A_23_P207680	ARL17	1.352614512	10.04472684	6.78E-06	4.32E-05
A_24_P359067	LOC100130357	-1.352598575	9.627968584	8.38E-06	5.18E-05
A_23_P411188	CHRNA10	-1.35251442	9.443291338	1.97E-05	0.000108323
A_23_P106727	RAB11FIP3	-1.35231046	14.0685791	6.11E-09	1.04E-07
A_23_P251795	GPC2	-1.352302049	11.37106756	0.00013368	0.000576351
A_23_P410312	C12orf76	-1.352301541	13.92742476	2.00E-08	2.86E-07
A_24_P382119	MTMR4	-1.352150709	15.44696236	1.78E-11	8.06E-10
A_23_P157495	PPP3CC	-1.351981414	12.56063088	3.82E-10	9.99E-09
A_23_P107903	RGL3	-1.351390859	11.26054442	0.015378334	0.034544563
A_24_P307816	KIAA1661	-1.351289997	7.839178853	0.000102879	0.000459829
A_23_P3900	WBP2	1.351217078	9.1546742	1.42E-08	2.14E-07

A_23_P214291	EFHC1	-1.350649575	12.59876296	7.61E-06	4.77E-05
A_23_P384663	MPP7	-1.350167119	9.576380842	5.82E-05	0.000280318
A_23_P10927	TM2D2	1.350144979	8.318402313	2.81E-08	3.81E-07
A_24_P370471	ATG10	1.349739611	9.206811736	1.89E-09	3.86E-08
A_32_P57057	USP15	-1.349566108	10.73901437	1.66E-07	1.76E-06
A_24_P916656	CRCP	1.349294611	10.11356441	1.25E-10	3.95E-09
A_23_P251965	ZNF273	-1.348906039	9.235073347	5.16E-05	0.000252474
A_23_P309739	ESR1	-1.348833792	8.230670708	0.000772274	0.002650388
A_23_P106481	TMEM85	1.348419651	13.2767563	2.05E-08	2.91E-07
A_23_P83624	COTL1	1.348291435	8.59721911	2.59E-09	5.06E-08
A_23_P111000	PSMB9	1.348178931	10.39116177	1.43E-08	2.15E-07
A_23_P252700	UNC50	1.348015357	11.71175474	1.64E-09	3.44E-08
A_24_P84668	FILIP1	-1.347785963	9.105618265	0.000557093	0.001994883
A_23_P83579	ARNT2	-1.347135877	13.77761284	1.57E-05	8.88E-05
A_24_P321068	SLC31A1	1.347009718	8.92417086	1.96E-09	3.99E-08
A_23_P165280	POLR2E	1.346912742	11.00380685	2.55E-09	5.00E-08
A_24_P101722	LOC126170	1.346430196	14.42224195	4.15E-07	3.85E-06
A_23_P51856	DUSP10	1.346377157	9.371875807	0.000114726	0.000505444
A_23_P23829	CD34	1.346346077	8.029797089	7.35E-09	1.22E-07
A_23_P159544	ASMTL	1.346174244	9.200579614	8.61E-11	2.92E-09
A_23_P97906	NT5C2	1.345863125	10.7515172	4.88E-08	6.14E-07
A_24_P49411	DEF8	-1.345520853	12.21788981	1.32E-08	2.02E-07
A_23_P134113	C6orf192	-1.345360427	11.75433551	5.74E-05	0.000276944
A_24_P267452	CD3EAP	-1.345308109	10.31469914	4.64E-05	0.000229403
A_23_P91910	PLSCR4	-1.345247761	10.57658953	0.001434747	0.00451142
A_23_P158485	TSPYL2	-1.345176346	14.20066973	4.97E-06	3.30E-05
A_24_P190424	RAB8A	1.34511299	8.954637408	5.45E-11	1.99E-09
A_23_P26367	RPS2P45	-1.344679472	9.155633075	9.17E-12	4.73E-10
A_23_P154266	PTCD3	-1.344488951	14.73394309	1.73E-09	3.59E-08
A_23_P138899	COPB1	1.344346389	13.22541912	2.00E-07	2.06E-06
A_24_P103060	MCFD2	1.344241464	9.645690748	1.12E-07	1.25E-06
A_23_P94754	TNFSF15	-1.344121419	9.44811875	0.00187547	0.005683844
A_23_P22027	INSIG1	-1.343742874	13.85217655	0.00083726	0.002845405
A_23_P128698	SPRY2	-1.343728194	10.73712916	9.90E-05	0.000444639
A_23_P254816	TCF15	-1.343599741	10.82681054	5.25E-05	0.000256122
A_32_P194779	ZBTB34	-1.343478861	11.70513785	3.95E-08	5.10E-07
A_23_P205228	ATP7B	-1.343467099	12.63190427	0.000164223	0.000688772
A_24_P137582	GFM2	1.343424794	10.40427066	2.88E-08	3.89E-07
A_24_P176079	WASF3	-1.343326054	12.44706308	4.30E-06	2.92E-05
A_23_P166333	UFD1L	1.343076206	12.99150961	1.63E-08	2.40E-07
A_24_P169343	C9orf21	1.343024384	10.41684944	2.22E-06	1.65E-05
A_24_P933565	PGAP1	-1.342797688	8.44842596	0.000173006	0.000720379
A_23_P373708	FLJ40504	1.342752662	12.99488693	5.75E-07	5.13E-06
A_23_P40354	MAPRE1	1.34230616	12.05238523	1.34E-07	1.46E-06

A_23_P135499	CLIC4	1.341771659	11.48065463	2.78E-06	2.00E-05
A_23_P54770	APOB48R	1.341496961	7.531807054	1.63E-08	2.40E-07
A_24_P75158	PTAR1	-1.340503867	14.27600249	5.24E-09	9.09E-08
A_23_P156101	TNPO1	1.340098522	11.11922916	1.55E-07	1.65E-06
A_23_P46924	BUB3	1.340085017	10.39008683	1.41E-09	3.05E-08
A_23_P153441	HOOK2	1.340076308	11.07915214	8.80E-08	1.02E-06
A_23_P80763	PVRL3	1.339951262	9.62783554	0.004627623	0.012337458
A_24_P56884	TMEM199	1.339932283	10.73604527	1.52E-11	7.13E-10
A_24_P179611	TPR	-1.339651494	14.09195708	1.18E-07	1.31E-06
A_24_P363679	ATP6V1H	1.339452994	10.26843687	0.000155104	0.000655283
A_23_P42178	HIST1H2BF	1.339425488	15.89681824	1.44E-05	8.25E-05
A_23_P122387	MRPS18A	1.338778508	12.74170077	1.28E-09	2.81E-08
A_32_P19135	RAB4B	1.338741283	8.922465196	1.11E-06	9.05E-06
A_23_P44581	NOMO1	1.338530342	11.46327001	8.19E-10	1.91E-08
A_24_P4110	ELP3	1.33850139	8.427130523	3.00E-12	1.87E-10
A_23_P114057	SEMA4C	-1.338486575	14.90984281	1.54E-05	8.73E-05
A_23_P401238	DDHD2	-1.338413421	12.874365	0.000407498	0.001522168
A_23_P93360	AGER	-1.338151553	10.38008393	2.48E-08	3.42E-07
A_23_P142075	ACP5	1.338076688	13.9598685	0.011851829	0.027618581
A_24_P469641	RNF216	-1.337860885	11.54630444	1.33E-07	1.46E-06
A_23_P159237	GPR20	-1.33784927	8.963686316	0.000108627	0.000481591
A_23_P53217	EED	-1.33782242	14.65493504	3.37E-10	9.02E-09
A_24_P516215	NOB1	1.337651719	9.516245569	7.29E-06	4.61E-05
A_32_P161661	TSNARE1	-1.337500992	12.2651094	1.33E-06	1.06E-05
A_23_P1638	RBM4B	-1.337490417	13.80301622	2.09E-06	1.56E-05
A_32_P15464	TMEM68	1.337374586	9.961885299	3.36E-05	0.00017271
A_23_P152782	IFI35	1.337085639	10.2519931	6.47E-10	1.56E-08
A_24_P81740	TALDO1	1.336818242	14.39800406	1.30E-12	9.52E-11
A_32_P101699	LOC729887	1.336728693	11.1513878	0.000249336	0.000991375
A_24_P128140	C18orf10	1.33667235	11.11395958	4.56E-08	5.78E-07
A_23_P64090	RAB1B	1.336291684	8.864081629	1.72E-07	1.80E-06
A_24_P194081	FXYD5	1.336197061	10.30364757	2.04E-09	4.13E-08
A_23_P138435	ZMIZ1	-1.336178794	15.62168122	2.82E-07	2.76E-06
A_23_P145786	MLXIPL	-1.336011688	8.80132687	0.000669309	0.002336813
A_32_P209735	LOC100292101	-1.33598416	12.56868932	0.000878755	0.002963519
A_24_P108351	HNRNPUL1	1.335871848	8.52933332	6.20E-12	3.38E-10
A_23_P304511	ZNF397	-1.335855878	11.44071939	0.000222725	0.000899336
A_23_P34433	ZCCHC11	-1.335798848	12.71330178	1.48E-10	4.50E-09
A_23_P157861	REXO4	1.335732653	10.51677952	2.32E-08	3.23E-07
A_23_P16992	PKP4	1.335499976	11.11789856	4.64E-11	1.74E-09
A_24_P527404	BMPR1A	1.335156133	9.258426907	1.63E-07	1.72E-06
A_24_P938516	C17orf85	-1.334890913	10.21818084	5.21E-05	0.000254479
A_23_P317620	ARL4C	1.334653773	10.30719044	0.001583652	0.004917695
A_23_P18447	PPARGC1A	-1.334647125	7.713964456	0.011721565	0.027372719

A_24_P34066	SIK3	-1.334511	7.696529689	1.18E-05	6.96E-05
A_32_P351968	HLA-DMB	1.334462121	12.45283841	0.006771253	0.017109419
A_23_P130158	WNT3	-1.334462083	10.74773236	0.000327591	0.001257676
A_24_P111271	PLA2G2D	-1.334423958	9.535911901	5.54E-09	9.56E-08
A_23_P317001	BNIP2	-1.334366537	13.50041846	6.67E-08	8.02E-07
A_23_P253586	DOPEY2	1.333987813	9.631058848	5.13E-09	8.92E-08
A_23_P2474	COPS7A	1.333887265	11.29782853	1.23E-11	5.99E-10
A_24_P393372	PACS2	-1.333875786	11.63246546	1.38E-05	7.96E-05
A_32_P225092	KIAA1217	1.333782287	9.108828453	1.83E-07	1.91E-06
A_24_P233878	GDAP2	1.333776694	9.260283264	3.90E-12	2.35E-10
A_23_P162702	C12orf51	-1.333634348	14.83002641	8.54E-07	7.23E-06
A_23_P143242	CEBPB	-1.333297717	9.603688949	3.19E-09	6.05E-08
A_24_P82106	MMP14	1.333166974	9.528469734	0.000151166	0.000641095
A_23_P136573	ST3GAL5	1.333163905	9.234547728	1.83E-05	0.000101748
A_23_P150457	LYVE1	1.333014301	8.733684564	0.001334875	0.004240108
A_23_P134204	FAM71F1	-1.332877235	9.550178209	0.000373341	0.001410358
A_23_P333420	RANGAP1	1.332763519	9.088482218	3.21E-10	8.65E-09
A_24_P107695	ACTN1	1.332754299	12.03027057	5.99E-07	5.31E-06
A_24_P342150	LOC100129387	-1.332652527	12.30125414	1.19E-08	1.84E-07
A_32_P32413	SETBP1	-1.332470083	12.57911641	2.23E-05	0.000120472
A_23_P49021	WDR61	1.332196142	12.3993547	3.64E-10	9.59E-09
A_23_P141730	DSG2	1.33081938	9.209951275	4.24E-09	7.65E-08
A_23_P134014	ADAT2	-1.330508236	10.37954257	7.30E-05	0.000341344
A_23_P360167	DCTN2	1.330493651	13.25747788	6.16E-15	1.27E-12
A_23_P98995	CALCOCO1	-1.330410367	11.01409437	2.30E-07	2.31E-06
A_32_P157208	LOC572558	-1.330345528	8.261086038	1.92E-07	1.99E-06
A_23_P72127	BEGAIN	-1.330079091	9.411176182	4.42E-06	2.99E-05
A_24_P163009	LOC151009	-1.330057589	14.67878963	0.000635366	0.002235045
A_32_P81334	LARP4	-1.330028333	9.184180976	1.50E-05	8.53E-05
A_23_P123133	OGDH	1.329914012	8.556031523	5.49E-13	4.82E-11
A_24_P740942	ALDOAP2	1.32991297	12.66179541	1.29E-05	7.52E-05
A_24_P11307	AHCYL1	1.329836574	11.30082033	5.62E-09	9.68E-08
A_24_P48139	ADAT1	1.329793666	8.589567009	1.00E-10	3.28E-09
A_23_P79999	ENTPD6	-1.329405881	14.83808583	2.67E-06	1.93E-05
A_24_P206344	ZNF746	-1.329035546	14.19385728	8.16E-10	1.90E-08
S4616917	LOC285147	-1.328816026	10.01422023	0.001411066	0.0044478
A_23_P59869	GJC3	1.328815411	9.000582314	0.014669801	0.033153133
A_23_P325991	NF2	1.328762113	8.359762185	7.12E-11	2.49E-09
A_23_P124164	ISY1	1.328716976	10.29394942	5.75E-13	5.00E-11
A_23_P99920	PTPLAD1	1.328701672	10.44906915	6.35E-07	5.59E-06
A_23_P422212	SLC35F3	-1.327986631	10.40682179	0.004485769	0.01200642
A_32_P131377	REEP5	1.32796316	14.61440293	2.25E-06	1.67E-05
A_24_P414999	LAPTM4B	1.327883973	10.36424495	0.000684938	0.002384926
A_23_P98002	CYP2E1	-1.327721501	10.06670645	0.009492946	0.022869477

A_24_P140475	SORBS2	1.327671946	9.181348498	2.70E-06	1.95E-05
A_23_P203255	API5	1.327610202	10.19709883	5.32E-07	4.79E-06
A_24_P273143	NCRNA00152	1.327598095	12.5066069	1.78E-05	9.90E-05
A_24_P300841	CKAP5	1.32750974	9.996122578	3.94E-08	5.10E-07
A_32_P87013	IL8	-1.327479933	8.773141009	0.00074095	0.002555351
A_24_P372223	MSR1	1.32739097	10.01685792	9.95E-05	0.000446553
A_23_P127915	STK33	-1.327108181	8.916193418	0.016165361	0.036027814
A_23_P251937	CPEB4	-1.326925016	13.87884007	2.49E-06	1.82E-05
A_24_P67980	LOC100129447	-1.326874214	7.447755278	4.73E-06	3.17E-05
A_23_P402254	GPLD1	-1.326847788	9.569944218	0.000699099	0.00242768
A_23_P203949	DDX11	-1.326787591	11.82338592	7.09E-06	4.49E-05
A_23_P307310	ACAN	1.326126778	9.218696942	0.001331313	0.004231919
A_24_P120934	GADD45G	1.325945463	12.8509171	0.004300248	0.011581966
A_23_P122563	PFDN6	1.325721483	13.85511754	2.69E-08	3.68E-07
A_23_P216038	PHF20L1	-1.32570762	12.21812497	1.23E-05	7.22E-05
A_23_P202501	RNLS	1.325615857	8.304335325	6.10E-05	0.000291893
A_23_P356717	ANKS1B	-1.325519927	7.717782901	0.001038809	0.003420123
A_24_P136497	DENND4A	-1.325172001	8.198143529	2.87E-06	2.06E-05
A_23_P380181	LMO4	1.325123329	12.2495795	5.02E-05	0.00024652
A_24_P354800	HLA-DOA	1.325036537	10.66403132	1.75E-07	1.84E-06
A_23_P71530	TNFRSF11B	1.324775761	8.261506228	0.000143223	0.000611767
S4553790	LOC728643	-1.324695848	8.300727372	1.36E-05	7.86E-05
A_24_P166211	DNAJC21	1.324681821	8.407246598	3.15E-08	4.20E-07
A_23_P17481	SIGLEC1	1.324281709	7.688424823	1.09E-06	8.89E-06
A_24_P102080	RAB2A	1.324125253	11.85214991	3.96E-05	0.000200132
A_32_P163169	VDAC1	1.324063519	10.45289522	4.34E-06	2.95E-05
A_23_P81408	MAT2B	1.323988351	10.71015449	2.22E-08	3.12E-07
A_23_P206759	KIAA0174	1.323828039	9.74833291	1.28E-06	1.02E-05
A_23_P83736	ING2	1.323716908	12.14706107	6.40E-07	5.62E-06
A_23_P335920	RPS6KA2	-1.323495477	13.03659615	2.88E-05	0.000151089
A_23_P391443	PPM1H	1.323427714	9.463362957	3.03E-06	2.15E-05
A_24_P396753	TRIB2	-1.32336637	8.906198709	3.08E-06	2.19E-05
A_24_P53150	TRAF7	-1.323326309	12.5034193	7.45E-08	8.83E-07
A_23_P106898	ORAI3	1.323308829	9.749855377	2.49E-06	1.82E-05
A_23_P64938	LPCAT3	1.323149007	10.04564596	9.13E-07	7.64E-06
A_23_P342118	CCDC124	1.323109776	11.22740235	4.61E-11	1.73E-09
A_23_P63908	TRUB1	-1.32275538	12.75257971	1.32E-06	1.05E-05
A_23_P66454	GSDMB	-1.322670237	11.31790871	2.60E-05	0.000137876
A_23_P28238	SNX17	1.322320613	9.41315925	1.51E-08	2.25E-07
A_24_P65292	SOX8	1.322002748	11.63532807	9.05E-08	1.04E-06
A_23_P399797	SMAD5OS	1.321873615	8.174290144	1.79E-08	2.60E-07
A_23_P37560	PEX11A	1.321748413	10.87532628	0.000547612	0.001964568
S18601049	CTA-221G9.4	-1.321333755	9.099770248	8.80E-07	7.41E-06
A_24_P917026	NF1	-1.321325345	12.5152086	2.73E-08	3.73E-07

A_32_P452655	LGALS9C	1.320966132	11.31095835	0.000838807	0.002850162
A_24_P341731	LOC100133224	1.320886964	11.37645894	2.44E-05	0.000130729
A_24_P4816	GABARAPL1	1.320752217	10.46725667	1.40E-06	1.11E-05
A_24_P504050	TMEM170A	-1.320735082	13.10481695	2.32E-08	3.22E-07
A_24_P273603	NUP188	-1.320719154	7.567571922	1.20E-06	9.68E-06
A_23_P158997	LOC729806	-1.320485366	8.269653456	1.13E-05	6.74E-05
A_23_P384635	DZIP1L	-1.320410083	8.824391464	0.000198808	0.000814061
A_24_P569294	MRPS12	1.32029152	12.59309867	5.76E-09	9.86E-08
A_23_P335848	GIT2	-1.3200179	13.24688228	1.57E-06	1.22E-05
A_24_P418250	NCRNA00086	-1.320002783	11.12572541	0.001113808	0.003627379
A_24_P312417	ZBTB26	-1.319477524	10.76604916	1.06E-06	8.70E-06
A_24_P57207	CREB3L3	1.319454182	10.8989161	1.16E-05	6.85E-05
A_24_P781757	LOC148413	1.318677974	8.731317156	1.44E-10	4.43E-09
A_23_P407206	CLN8	-1.318486024	10.58052785	6.45E-06	4.15E-05
A_23_P97532	VAMP3	1.318228853	10.67099037	1.65E-06	1.27E-05
A_23_P38894	C19orf66	-1.318208143	13.23212109	0.000155448	0.000656236
A_24_P171182	ACBD3	1.318011863	10.75639677	1.63E-06	1.26E-05
A_23_P150841	ZNF140	1.317800526	9.347077791	2.49E-08	3.44E-07
A_23_P89710	CEP192	-1.317595786	12.47794664	2.14E-05	0.000116768
A_23_P54929	LYRM1	1.317532416	11.91526637	4.88E-06	3.25E-05
A_23_P325155	CD200R1	-1.317522137	8.110989483	4.53E-05	0.000224893
A_32_P378035	KIAA1656	-1.316941665	7.570100094	3.31E-10	8.89E-09
A_23_P395075	KDM3A	-1.316736915	11.5804989	0.001538559	0.004796145
A_23_P20777	RBM18	1.316721628	12.21519023	6.40E-08	7.74E-07
A_24_P406480	LONRF1	1.315795927	9.388153432	5.56E-05	0.000269535
A_23_P208925	SH3GL1	1.31566677	9.274847214	6.51E-12	3.54E-10
A_24_P43884	MAPKAP1	1.315548867	11.26908384	2.42E-09	4.78E-08
A_24_P419309	SNRNP40	1.315533187	11.55337795	7.01E-09	1.17E-07
A_23_P55551	CHMP1B	1.315453077	10.87859575	3.90E-08	5.05E-07
A_24_P283189	CD14	1.315094957	9.551460463	3.63E-07	3.43E-06
A_23_P382811	SNX31	-1.314828087	8.629481341	0.00074766	0.002576112
A_24_P345002	NUDT11	1.314614424	9.662450777	0.013539235	0.030952423
A_23_P57236	GGT7	1.314562779	8.491663395	1.77E-08	2.57E-07
A_24_P379512	PIGK	1.314149905	10.9402723	6.89E-07	5.98E-06
A_24_P419211	MTMR6	1.31398808	10.42892878	1.04E-05	6.28E-05
A_23_P91221	PKIG	1.313909642	9.908804701	9.68E-11	3.21E-09
A_23_P9166	TLN1	1.3137404	8.755846916	6.56E-12	3.55E-10
A_23_P12733	H2AFY2	-1.313678582	11.74404505	0.000203943	0.000832445
A_24_P136725	SPIN3	-1.313643136	11.0383872	4.53E-05	0.000224854
A_23_P317691	UIMC1	-1.313578114	14.74362495	1.66E-09	3.48E-08
A_24_P743869	NCRNA00081	1.31356443	9.113230484	1.18E-06	9.57E-06
A_24_P913339	C2orf18	1.313545633	11.05447511	3.73E-09	6.90E-08
A_23_P201816	CEP350	-1.313300728	14.17341713	7.40E-09	1.22E-07
A_24_P386771	PPP1CC	1.313165874	12.45538143	6.49E-07	5.68E-06

A_23_P354175	TMEM129	-1.313017999	13.48891482	7.22E-06	4.57E-05
A_24_P371758	MRPL36	1.31296519	13.56984324	1.34E-08	2.04E-07
A_32_P96641	NDUFA5	1.31288435	13.39001014	1.42E-05	8.15E-05
A_24_P378368	MBTPS1	1.312698933	8.759730987	3.23E-12	2.00E-10
A_23_P384512	ZNF22	1.312513756	9.195396906	4.85E-06	3.24E-05
A_24_P268015	MEAF6	1.312439924	13.03282618	5.38E-05	0.000261626
A_23_P410040	KCNH7	1.31237634	8.440946965	1.60E-09	3.38E-08
A_23_P109420	BMS1	1.312124671	11.85669896	2.90E-09	5.59E-08
A_23_P321485	COX18	1.312101702	11.96732319	1.38E-07	1.50E-06
A_24_P811704	PPFIBP1	-1.312027136	11.0849679	4.59E-06	3.09E-05
A_24_P162412	WDR20	-1.311997486	9.968073732	7.00E-06	4.44E-05
A_23_P26922	HDAC5	1.311704914	9.667243429	5.64E-09	9.69E-08
A_23_P215037	WTAP	1.311633761	11.99531893	2.21E-06	1.64E-05
A_23_P23572	PIP5K1A	1.311520097	8.984323244	1.37E-08	2.07E-07
A_24_P16124	IFITM4P	1.311440444	14.59208079	0.000390741	0.001468075
A_23_P88095	TBC1D4	1.311342482	10.51345968	0.000118628	0.000519735
A_23_P133332	MRPS27	1.311297128	12.14920799	9.19E-10	2.11E-08
A_23_P35148	TAF13	1.311216502	9.649000797	5.89E-07	5.24E-06
A_24_P193027	APOF	1.311141261	8.271006543	0.001868946	0.005666991
A_23_P501183	NSUN5C	-1.310946682	15.565464	3.54E-07	3.37E-06
A_24_P403459	IFNA4	-1.310637538	10.10502925	1.45E-06	1.14E-05
A_23_P167692	MAPK9	1.310589488	9.683761771	8.63E-08	1.00E-06
A_24_P415012	LRP12	-1.310379121	12.3829829	7.00E-05	0.000328943
A_23_P151133	TSPAN9	1.310298233	10.06092754	6.13E-08	7.44E-07
A_23_P44083	GFPT1	1.310205266	11.78181914	0.000203847	0.000832139
A_23_P113777	ITGBL1	1.310194054	10.53714798	0.021709407	0.046357569
A_24_P472455	ARF6	1.310184511	11.39642107	1.74E-06	1.34E-05
A_32_P114866	LOC284570	1.310017162	11.97928804	2.50E-07	2.49E-06
A_23_P90710	DES	-1.309796892	7.720004876	0.000759397	0.002611524
A_32_P155811	CD2AP	-1.309741477	12.82422033	3.04E-08	4.07E-07
A_32_P391586	CCDC46	-1.309656084	8.083321119	7.31E-07	6.30E-06
A_23_P24987	TSPAN31	1.309604355	11.87975488	1.45E-06	1.14E-05
A_23_P29303	RRP7A	1.309396088	9.160948975	1.76E-09	3.64E-08
A_23_P90533	POP4	1.309332339	13.12220878	1.40E-10	4.32E-09
A_23_P3312	ISLR	-1.309196158	11.32469816	0.004239769	0.011434197
A_23_P94030	LAMB1	1.308925255	14.06855288	0.002963514	0.008418846
A_23_P167553	DHFR	1.308778429	9.937740444	1.26E-05	7.35E-05
A_32_P118655	CTBP1	-1.308719514	10.66278662	0.003456957	0.009599351
A_24_P83262	TSPAN6	1.308191201	10.76205627	1.94E-06	1.46E-05
A_23_P11201	GPR34	1.308122665	8.291290178	3.60E-05	0.000184023
A_24_P340428	ATP11B	-1.307685174	9.532197498	0.000620652	0.002190653
A_32_P8251	PHRF1	-1.307619436	14.53730829	1.64E-08	2.41E-07
A_24_P164731	TMED1	1.307477905	10.06965643	5.63E-09	9.68E-08
A_23_P90743	REG1A	1.306971279	8.106135204	0.018884546	0.04117063

A_23_P17420	BCAS1	1.306915782	9.517006175	0.010416986	0.024730676
A_24_P109082	USP37	-1.306896453	12.82236861	1.00E-08	1.59E-07
A_23_P48495	TCL1B	-1.306504827	9.601970918	1.17E-05	6.89E-05
A_24_P132518	IKBKB	-1.306053952	13.17182405	5.44E-05	0.00026431
A_23_P379634	SLFNL1	-1.305875077	8.9669464	0.000507734	0.001841999
A_24_P399362	PSMG2	1.305853869	13.04356025	4.43E-08	5.62E-07
A_23_P343843	CENPBD1	1.305794579	8.88717114	8.25E-10	1.92E-08
A_24_P339201	METRN	1.305576912	12.12394195	0.001224623	0.003939378
A_23_P149707	DLGAP3	1.305479046	12.7978444	1.18E-06	9.55E-06
A_23_P39095	CGB1	-1.30532607	9.168022495	0.005736581	0.014840019
A_24_P912751	CYP4V2	1.305121268	10.75158701	5.43E-10	1.34E-08
A_23_P217688	TSC22D3	1.304887947	9.245576726	6.08E-08	7.39E-07
A_24_P933580	NAV2	-1.304818152	8.419811168	0.009952086	0.023803481
A_23_P216396	EXOSC2	-1.304474364	11.10797591	7.25E-08	8.61E-07
A_24_P34505	NOL10	-1.304309064	10.88209096	4.19E-12	2.47E-10
A_23_P323143	ZNF767	-1.303968791	10.44687315	1.32E-07	1.44E-06
A_24_P355493	LHPP	1.30363651	9.596396973	3.51E-07	3.34E-06
A_23_P66599	VPS25	1.303442335	12.73826793	1.60E-12	1.13E-10
A_32_P399546	ARNTL2	-1.303303724	9.41066823	4.63E-10	1.17E-08
A_23_P74900	ESRRG	-1.303294845	9.860995596	0.011564121	0.027068759
A_23_P207301	PRR11	-1.302894008	15.29204298	2.79E-07	2.74E-06
A_23_P200928	NID1	1.302765153	9.073570248	1.36E-06	1.08E-05
A_23_P67169	IL11	-1.302719538	9.850244344	0.000245166	0.000977202
A_24_P106953	PTGES2	1.302673078	10.08503507	1.47E-09	3.16E-08
A_32_P76811	RP3-377H14.5	1.302302921	15.78718728	7.58E-10	1.80E-08
A_23_P5089	ATP5D	1.302295016	14.00292959	1.54E-07	1.64E-06
A_23_P37484	CHSY1	-1.302094078	15.32651441	2.26E-07	2.28E-06
A_23_P69121	SIAH2	1.302043093	10.86854337	1.76E-07	1.84E-06
A_23_P129075	WDR76	1.30185546	8.251284443	1.88E-06	1.42E-05
A_32_P24939	BMPR1B	1.301852693	9.974922607	0.006961942	0.017512342
A_23_P44734	USP38	1.301836223	8.610241257	1.55E-09	3.29E-08
A_24_P94651	ARIH2	-1.301684968	15.14800125	2.62E-08	3.59E-07
A_32_P194115	UNC84B	1.300687923	8.883425067	4.23E-10	1.09E-08
A_23_P717	TMEM206	1.300622805	10.37454046	2.98E-07	2.90E-06
A_24_P560519	PPARGC1B	-1.300610789	11.13140188	0.00075481	0.002596905
A_23_P109235	RALY	1.300337362	10.8817576	1.08E-13	1.28E-11
A_32_P169574	OTUD5	1.300301513	10.66978214	1.35E-06	1.07E-05
gi|56550088|ref|NM_007157.3|	ZXDB	-1.300261257	11.87508808	9.70E-08	1.11E-06
A_24_P65271	C16orf58	-1.299970624	11.41466038	1.26E-08	1.93E-07
A_23_P401361	PITPNM2	-1.299915957	8.52120915	1.12E-06	9.13E-06
A_32_P532216	QARS	1.299790198	9.987705917	1.34E-07	1.46E-06
A_23_P216291	CPA6	-1.299733247	7.628594496	0.007271379	0.018165809
A_32_P194246	CLEC16A	-1.299724693	13.8251192	9.96E-11	3.27E-09
A_23_P34827	HCN3	-1.299722213	10.07373775	0.000244668	0.000975519

A_23_P202013	ZEB1	1.299227105	8.685193977	3.74E-06	2.59E-05
A_24_P305993	CCDC67	-1.298919081	7.818086006	0.004654693	0.012398256
A_23_P366216	HIST1H2BH	1.2985481	15.39625808	0.000126098	0.000547264
A_23_P79426	CAB39	1.298190374	11.55155555	4.29E-06	2.92E-05
A_23_P413796	HAUS1	1.298105404	11.27647087	2.08E-06	1.56E-05
A_23_P54116	DAAM1	1.297825611	12.00593772	5.93E-06	3.85E-05
A_23_P59700	BET1	1.29781672	10.49124689	1.46E-06	1.14E-05
A_23_P162425	GOLT1B	1.29700947	8.614422405	3.71E-08	4.82E-07
A_23_P12336	PRMT6	1.296971221	11.61949385	5.57E-05	0.000269922
A_24_P239407	LOC100289109	-1.296791293	8.147365165	4.07E-05	0.000204846
A_23_P202637	SAPS3	1.296703477	10.45242105	6.63E-08	7.98E-07
A_24_P200023	IL1R1	1.296672743	10.77705986	0.000804869	0.00274743
A_24_P643041	RNF216L	-1.296464949	11.79794851	2.09E-07	2.13E-06
A_23_P253052	CD99L2	-1.296360278	14.40175807	1.37E-05	7.91E-05
A_32_P93149	DDX10	-1.296235261	8.524835662	9.20E-08	1.06E-06
A_24_P276791	LRRC42	1.295892408	10.11645476	2.08E-08	2.95E-07
A_23_P391238	MCM3APAS	-1.295877331	9.641792712	0.000209419	0.000852281
A_23_P381992	ITGAV	1.295738185	8.837872483	1.73E-06	1.32E-05
A_23_P128650	SLC25A15	1.295689841	10.77929678	6.71E-07	5.85E-06
A_24_P367329	LOC440292	1.295318417	10.19016621	2.57E-09	5.03E-08
A_23_P201079	PRDM2	-1.295200401	11.31608042	9.19E-07	7.69E-06
A_23_P328729	KLHL8	1.295137037	9.713857206	3.87E-07	3.62E-06
A_23_P125771	HCFC1	1.29497106	8.444899273	1.15E-13	1.34E-11
A_23_P409386	SLC25A22	-1.294874201	13.30004154	1.08E-06	8.84E-06
A_23_P502797	WDFY1	1.294696651	10.02336254	3.56E-07	3.38E-06
A_24_P74981	KDM1A	1.2943149	9.610880306	3.06E-07	2.96E-06
A_24_P358381	GTPBP6	1.293958975	9.127386531	1.20E-08	1.85E-07
A_24_P644742	HAUS6	-1.293852227	8.133158194	7.16E-10	1.71E-08
A_23_P213766	ANKH	1.293434634	11.37216324	7.83E-05	0.0003624
A_24_P324449	ABCB10	1.293061445	9.215095906	2.55E-05	0.000135457
A_23_P131383	FANCL	-1.292910246	13.77519548	1.25E-06	1.01E-05
A_32_P96272	PPIAL4A	1.292759981	14.44150903	4.41E-11	1.66E-09
A_23_P369666	ZMYND8	-1.292682189	11.8146415	0.000242185	0.000966917
A_32_P125832	LOC100128893	-1.292673473	6.935978957	2.89E-06	2.07E-05
A_23_P139228	ARFIP2	1.292496862	12.44145009	7.93E-11	2.73E-09
A_23_P43273	EXT1	1.292130637	10.39626794	3.44E-08	4.52E-07
A_24_P245646	TP53RK	1.291897186	11.16096828	2.51E-11	1.06E-09
A_24_P935881	SERF1B	1.291854273	12.58739925	1.25E-07	1.37E-06
A_23_P258493	LMNB1	1.291715702	13.78992033	0.001537801	0.004794942
A_24_P307724	FLJ33360	-1.291563143	8.817970465	7.92E-05	0.000366334
A_23_P152768	TUBG1	1.291421642	11.88874262	0.000120313	0.000525631
A_24_P568190	SMA4	-1.291366448	13.23975308	0.000839561	0.002851723
A_23_P370574	KIR3DL3	-1.291295185	8.862030496	5.59E-06	3.66E-05
A_24_P88565	RAG1AP1	1.291111445	11.49428746	2.49E-08	3.43E-07

A_23_P56140	CSNK1G2	1.290744959	12.57440716	5.49E-07	4.92E-06
A_23_P49842	UNC119	1.290715969	10.13528417	1.64E-11	7.54E-10
A_24_P132950	NAP1L4	1.290614186	10.9984543	4.11E-09	7.46E-08
A_23_P99320	KRT18	1.290353966	13.72704663	2.96E-07	2.88E-06
A_23_P66924	DYM	1.290231549	9.097536581	4.84E-11	1.80E-09
A_24_P328819	C4orf41	1.289914842	9.838425338	6.44E-07	5.64E-06
A_23_P208240	ZNF160	-1.289669058	8.968028927	0.00014578	0.000621594
A_23_P502609	PIGQ	1.289602386	9.899182217	2.08E-11	9.19E-10
A_24_P254084	ZNF69	1.28954462	8.966837275	1.01E-09	2.29E-08
A_24_P191884	PHAX	1.289430757	10.41272003	7.82E-10	1.84E-08
A_24_P124973	NDNL2	1.289190463	9.959528204	1.76E-09	3.64E-08
A_23_P3514	C16orf80	1.288780367	12.46009872	8.36E-09	1.36E-07
A_23_P124733	COQ2	1.288706353	10.15138128	4.45E-07	4.10E-06
A_24_P911179	ASPM	-1.288483149	10.19844834	0.009585076	0.023056976
A_23_P32404	ISG20	1.288413228	13.51092095	0.001119951	0.003644479
A_23_P258570	PSMD10	1.288208384	11.65854028	8.21E-07	6.99E-06
A_24_P677642	FLJ31813	1.288089748	10.16904475	5.14E-08	6.41E-07
A_23_P374315	C13orf31	-1.287888462	8.432464355	0.000251923	0.001000426
A_23_P155765	HMGB2	-1.287825318	15.24131675	0.000315377	0.001218044
A_23_P206822	XPO6	-1.287821913	13.12371768	1.85E-05	0.000102326
A_24_P135167	PCM1	-1.287677019	12.09860088	9.54E-08	1.10E-06
A_23_P379034	BAIAP2L2	-1.287572675	10.18539612	0.009233288	0.022324991
A_23_P2942	NPAS3	-1.287369143	9.259401634	0.006931051	0.017453924
A_23_P100341	ORC6L	-1.286941991	16.38525625	8.29E-08	9.67E-07
A_24_P918500	ITSN1	-1.28687251	8.434071269	2.36E-05	0.00012674
A_23_P324490	KIAA0355	-1.286639877	15.02752099	8.67E-10	2.00E-08
A_24_P172738	F7	1.286068265	10.19892026	9.34E-09	1.50E-07
A_23_P159663	UXT	1.286035385	14.92155371	1.42E-06	1.12E-05
A_23_P101707	PLIN3	1.285834201	11.78739197	1.88E-09	3.86E-08
A_32_P429872	IER3IP1	1.285284665	12.82790745	1.22E-08	1.88E-07
A_23_P76658	N4BP2L1	-1.2852713	10.69237821	0.003581547	0.009900554
A_32_P155416	ERI3	1.285179061	9.841385178	1.45E-08	2.18E-07
A_23_P333484	HIST1H3H	1.284952458	10.90985863	3.18E-08	4.23E-07
A_23_P148150	COBRA1	1.284849541	11.58768539	1.01E-10	3.29E-09
A_23_P171034	NSDHL	1.284780281	9.067094894	6.84E-08	8.18E-07
A_23_P44768	TBK1	-1.28416012	13.81561691	2.08E-06	1.55E-05
A_23_P25194	HRK	-1.284129911	9.712250943	0.00197445	0.005941929
A_32_P98313	NDUFA4	1.284118722	15.61973048	1.52E-06	1.18E-05
A_23_P118353	SRP68	1.284097567	10.1388637	2.75E-11	1.14E-09
A_23_P143274	NRSN2	-1.283790579	9.744469945	2.67E-05	0.000141414
A_32_P209325	LOC100290885	-1.283202628	9.081224133	4.24E-09	7.64E-08
A_32_P70135	C13orf23	-1.283085847	13.49251306	1.87E-08	2.70E-07
A_23_P140792	EEF2K	-1.283037199	9.956222134	3.99E-05	0.000201331
A_23_P64669	ZCCHC8	-1.282913448	13.79931667	3.93E-07	3.67E-06

A_32_P88817	SPDYE7P	-1.282856643	9.387148535	3.81E-08	4.94E-07
A_24_P808100	MCTS1	-1.282471089	10.25228676	2.72E-05	0.000143823
A_23_P394276	RWDD4A	-1.282319277	13.59067954	1.57E-06	1.22E-05
A_24_P399220	HOXB3	-1.282299619	11.08710511	0.007472166	0.018589761
A_23_P70785	AIM1	1.282292358	11.25877794	0.002132553	0.006347627
A_24_P364381	MMAB	1.282207764	13.2675175	2.29E-06	1.69E-05
A_24_P251221	PPP2R5A	1.281909347	11.28330028	6.21E-07	5.48E-06
A_23_P300867	MIPOL1	1.281658363	9.872356608	1.77E-05	9.86E-05
A_23_P20932	PDHB	1.281614322	12.02085006	1.03E-06	8.48E-06
A_24_P156922	SCP2	1.281214721	9.087043461	4.40E-08	5.59E-07
A_23_P5983	PLTP	1.280986128	10.41324128	0.000442952	0.001635967
A_24_P148450	UBE2E3	1.28081096	11.67508716	3.68E-05	0.000187481
A_32_P48842	LOC644538	1.280682475	9.433024654	0.002594782	0.007520418
A_23_P51572	TSNAX	1.280660643	10.59833797	5.87E-07	5.21E-06
A_23_P405754	CACNB1	-1.28048421	10.64105914	0.000379146	0.001429092
A_23_P54556	MKL2	-1.280442784	14.98888656	1.46E-06	1.15E-05
A_24_P128205	PSMD1	1.280276037	12.29782349	3.73E-09	6.90E-08
A_24_P179585	MARK1	-1.280131861	10.88491274	0.003375519	0.009404933
A_24_P147263	USP31	-1.279893259	13.68264293	1.34E-06	1.06E-05
A_24_P377065	ARL8B	-1.279862931	10.87861935	6.77E-06	4.32E-05
A_24_P367812	LOC650392	1.279781391	13.3713607	5.69E-07	5.08E-06
A_23_P126448	APOA1BP	1.279710497	13.59989674	1.07E-10	3.46E-09
A_23_P49279	C16orf87	1.279285244	10.73227681	1.66E-06	1.28E-05
A_23_P34744	CTSK	1.27889941	13.70926968	0.002942131	0.00836822
A_24_P77681	PAIP1	1.278871854	12.79742235	2.96E-08	3.98E-07
A_23_P398836	TXNDC16	1.278592568	12.66931319	0.000210257	0.000855241
A_24_P59494	PARP11	1.27847051	9.013376406	1.19E-08	1.85E-07
A_23_P2884	PIGH	1.277936073	12.18916279	8.42E-06	5.20E-05
A_24_P297680	PDXDC2	1.277769937	10.18421445	3.57E-06	2.49E-05
A_24_P19228	GAMT	1.277600862	11.99764802	1.52E-06	1.18E-05
A_23_P134734	GOLSYN	-1.277268428	13.62871363	0.002672171	0.007716968
A_23_P59616	GTF2IRD2	1.277268047	9.627156842	1.01E-06	8.35E-06
A_23_P329870	RHBDF2	-1.277206681	13.04713136	3.93E-05	0.000198732
A_23_P357104	ANXA6	1.276994818	10.96092915	1.95E-05	0.000107508
A_23_P380839	C11orf30	-1.276992281	9.132490758	8.92E-07	7.50E-06
A_23_P377115	MCC	1.276700203	8.47137666	3.65E-05	0.000186127
A_23_P56578	VIT	1.27662526	8.59377228	0.008052445	0.019821146
A_23_P216915	NR5A1	-1.276499311	7.663618985	2.18E-14	3.51E-12
A_23_P16423	ZBTB7A	-1.27636476	13.48875361	3.23E-06	2.28E-05
A_23_P138105	MED18	1.276154612	8.39256817	5.77E-10	1.41E-08
A_24_P313109	UFM1	1.27591831	10.27783526	1.57E-06	1.22E-05
A_23_P250102	CAND2	-1.275591375	10.14308782	0.010460143	0.02481328
A_23_P74115	RAD54L	-1.27533879	11.3891124	0.002509987	0.007315203
A_32_P212802	LOC442572	-1.275200898	9.298638186	2.62E-08	3.60E-07

A_23_P118435	SUMO2	1.275173079	14.94133292	5.35E-08	6.64E-07
A_32_P99032	LOC100240734	-1.275037465	9.798184069	4.98E-05	0.000244771
A_23_P392126	C17orf108	-1.274783998	12.57223686	0.000185868	0.000767104
A_23_P59798	MKRN1	-1.274629235	15.43885659	1.02E-09	2.30E-08
A_23_P420981	C14orf79	1.274324372	9.266045706	1.43E-06	1.13E-05
A_23_P149529	TACSTD2	1.27415745	15.1815972	0.022782416	0.048286442
A_23_P44586	COX5A	1.274030718	14.3633148	4.15E-07	3.85E-06
A_32_P47566	UBLCP1	1.27398402	8.611735943	4.99E-10	1.25E-08
A_32_P47157	LOC92973	-1.273606609	9.071647765	0.002672895	0.007718482
A_24_P238855	SETD8	1.273508933	10.00117658	7.31E-06	4.61E-05
A_24_P108738	SCARF2	1.273355583	9.741047617	1.46E-06	1.15E-05
A_23_P325501	MORC3	-1.273213284	13.10349735	3.33E-09	6.26E-08
A_32_P24059	AGK	-1.273001732	11.46728274	1.21E-06	9.79E-06
A_24_P65098	TMEM87A	-1.272897855	10.22508655	1.47E-05	8.42E-05
A_24_P25326	ZMYM6	-1.272656204	10.78355933	2.77E-07	2.72E-06
A_32_P18668	RAB3B	1.272547736	11.73181365	6.68E-05	0.000315817
A_23_P63209	HSD11B1	-1.272513824	8.299907316	0.022378664	0.047563572
A_23_P37327	ABCD4	-1.272225978	12.58622687	1.54E-09	3.27E-08
A_23_P18824	POLS	-1.272171989	14.10875679	1.94E-07	2.01E-06
A_32_P140898	FOXN2	-1.272004522	9.272498084	2.36E-06	1.73E-05
A_23_P206041	MAN2A2	-1.271935755	13.69709906	3.91E-08	5.06E-07
A_23_P425066	CRYBB2	1.271662521	9.904215636	0.000995351	0.003297891
A_23_P128060	ZNF26	-1.27154414	12.26377893	0.00030229	0.001173702
A_23_P123010	BCL7B	1.271373622	8.515547931	7.38E-11	2.58E-09
A_24_P307025	RPL23AP7	1.271115703	14.34342482	8.99E-09	1.45E-07
A_23_P126393	SETDB1	-1.270967738	13.70203614	1.47E-06	1.15E-05
A_24_P126282	BTN3A1	-1.270934882	9.262528665	0.007286118	0.018196484
A_23_P100576	PSMB3	1.270826334	13.88379864	3.81E-10	9.98E-09
A_23_P108028	ZNF146	1.270778319	11.27794871	9.51E-08	1.09E-06
A_23_P345591	PSMA2	1.27074017	14.56823336	8.37E-06	5.17E-05
A_23_P321160	ZNF594	-1.270725462	10.85753375	6.85E-06	4.36E-05
A_24_P71280	GPR157	-1.270675737	10.12000104	0.000174763	0.000726754
A_24_P39654	GRB2	1.270621441	11.95757969	1.73E-08	2.53E-07
A_23_P160214	TTC39A	1.270544011	11.62050966	5.34E-05	0.00025985
A_24_P55437	TES	-1.270388147	12.5303938	5.01E-05	0.000245839
A_23_P204484	RAB35	1.270255316	12.72827495	1.40E-08	2.12E-07
A_23_P317657	DDX3X	-1.270194845	15.17890527	2.75E-05	0.000144819
A_23_P137705	TBX19	-1.270015953	11.3539711	6.83E-07	5.93E-06
A_23_P2998	OXA1L	1.269981334	11.83084638	1.10E-10	3.55E-09
A_24_P400355	C7orf20	-1.269924849	13.80931526	6.26E-09	1.06E-07
A_24_P380234	FAM53C	-1.269919067	13.47759369	9.72E-08	1.11E-06
A_24_P157053	ADAM17	-1.269836777	12.46331026	1.41E-08	2.13E-07
A_24_P116700	TMEM220	1.269663214	9.705602731	0.015498145	0.034767274
A_23_P13083	BARX2	1.269490083	7.32493313	1.73E-05	9.66E-05

A_32_P44568	LDHA	1.269349286	11.96747904	0.000131355	0.000567526
A_23_P33119	C8orf33	1.268914792	10.22898657	6.27E-07	5.52E-06
A_23_P7761	C5orf22	1.268464662	11.68730192	3.25E-06	2.29E-05
A_24_P398781	C14orf179	1.268210603	13.49323327	1.18E-07	1.31E-06
A_23_P213093	OCIAD1	1.268208066	14.28204578	2.19E-08	3.09E-07
A_23_P82839	AGPAT6	1.268159304	10.28043326	6.33E-07	5.57E-06
A_23_P141520	C17orf49	1.268086398	12.84463664	6.07E-09	1.03E-07
A_23_P61810	BAIAP2	-1.268033837	13.03988334	1.29E-05	7.49E-05
A_23_P153628	YIF1B	1.268008893	10.82489281	4.89E-10	1.23E-08
A_23_P502047	CHRD	-1.267928857	10.35168799	0.001239554	0.00397782
A_23_P49060	SPINT1	1.267692891	11.08153731	1.09E-08	1.71E-07
A_23_P316812	AFARP1	1.267526681	9.152268944	7.47E-12	3.93E-10
A_24_P54879	SCARB2	1.267502482	12.78205399	2.95E-06	2.11E-05
A_23_P130418	NDUFV2	1.267418337	14.65912624	1.81E-09	3.73E-08
A_24_P79153	SCAMP4	1.267048045	9.463798444	8.37E-09	1.36E-07
A_23_P351695	SYNRG	-1.266946054	9.947647981	3.19E-07	3.06E-06
A_32_P725218	METTL2A	1.266918712	10.56865374	4.66E-07	4.27E-06
A_23_P167509	CYFIP2	1.266797065	10.67544629	9.36E-05	0.000423075
A_24_P932418	AP2A2	-1.266599791	12.83294599	7.23E-06	4.57E-05
A_32_P209989	MRPL46	1.266375774	11.15627015	2.10E-09	4.23E-08
A_23_P72025	SLC25A20	1.26628337	10.77756816	0.000218707	0.000885514
A_23_P111452	AGAP3	-1.266094125	14.69411181	8.29E-08	9.67E-07
A_24_P185986	UPRT	-1.26572455	13.47822844	3.88E-08	5.03E-07
A_23_P208812	ZNF507	-1.265585165	10.87224389	3.08E-09	5.87E-08
A_24_P149124	C5orf13	-1.265492952	13.68824534	0.002231952	0.006608921
A_24_P367602	DUSP5P	-1.265125455	10.49289153	0.00322962	0.00905712
A_24_P382467	SLC39A3	1.264905451	10.54602136	3.12E-09	5.94E-08
A_24_P320526	C6orf106	1.264863345	13.45922488	3.01E-07	2.93E-06
A_23_P34325	LRP8	-1.264783095	11.02965249	0.001264547	0.004047283
A_23_P201676	METTL13	1.264656221	10.042034	1.24E-11	6.01E-10
A_23_P101141	RNF125	-1.264539293	9.198910616	0.000312468	0.001208613
A_23_P86089	VWA1	1.264380494	14.41820923	0.000484924	0.001768034
A_23_P129458	SDR42E1	1.264044939	9.048460888	0.000874847	0.002951626
A_24_P942469	SPAG9	1.263817606	11.09852233	2.39E-08	3.31E-07
A_23_P259632	TMEM120A	1.263656362	12.62975196	1.71E-06	1.31E-05
A_23_P139547	TUBA1A	1.263470083	12.6141571	1.13E-07	1.26E-06
A_24_P166443	HLA-DPB1	1.263432282	11.37491894	0.00015659	0.000660489
A_23_P161595	MARK2	-1.263306817	13.84400518	1.78E-06	1.36E-05
A_24_P260639	HIST1H1D	1.263083455	12.91488411	0.004654913	0.012398256
A_24_P158946	FGD4	-1.263053821	11.76233574	4.29E-05	0.000215002
A_24_P535219	PHF10	-1.262793744	12.50564181	0.000183261	0.000758265
A_32_P26895	FAM160B1	-1.262661908	13.78345966	6.35E-06	4.09E-05
A_23_P121102	HESX1	-1.262602655	8.661168666	0.000132808	0.000573101
A_23_P18276	RBM5	-1.26256126	15.27091665	1.20E-06	9.72E-06

A_23_P416142	DLG1	1.262424512	10.03512539	4.07E-08	5.24E-07
A_32_P30649	ETV5	1.262172048	9.235306747	7.28E-05	0.000340339
A_23_P57819	TATDN2	-1.262073493	13.09825389	2.56E-10	7.18E-09
A_23_P16915	QPCT	1.262048783	9.979295986	0.000567529	0.002027002
A_23_P408376	HSPA12A	-1.262046068	11.16891679	8.23E-05	0.000378334
A_23_P41716	GNB2L1	1.261791455	14.07613229	2.05E-08	2.91E-07
A_32_P453321	C1orf228	-1.261549789	9.785557958	0.000117651	0.0005161
A_24_P122050	PSMF1	1.261373302	10.19570425	1.59E-11	7.37E-10
A_23_P139632	GNS	1.261173707	9.052520798	4.40E-09	7.88E-08
A_23_P63219	POGZ	-1.260940953	15.60184615	1.08E-08	1.70E-07
A_24_P269619	DECR1	1.260909685	12.06831485	0.000800357	0.002733978
